# Recent Advances and Future Perspectives of Proton-Conducting Electrolytes for Reversible Solid Oxide Cells

**DOI:** 10.1021/acs.chemrev.5c00529

**Published:** 2026-08-20

**Authors:** Zheyu Luo, Chenhui Yang, Xueyu Hu, Haoyu Li, Zhijun Liu, Weining Wang, Yuefeng Song, Meilin Liu

**Affiliations:** † School of Materials Science and Engineering, 256693 Georgia Institute of Technology, Atlanta, Georgia 30332-0245, United States; ‡ State Key Laboratory of Catalysis, Dalian National Laboratory for Clean Energy, iChEM (Collaborative Innovation Center of Chemistry for Energy Materials), 543687 Dalian Institute of Chemical Physics, Chinese Academy of Sciences, Dalian 116023, China

## Abstract

Proton-conducting reversible solid oxide cells (P-RSOCs) are emerging as a transformative platform for efficient and flexible conversion between electricity and chemical fuels, including hydrogen and syngas. Their intermediate-temperature operation (400–600 °C) offers a compelling combination of high energy efficiency, rapid reaction kinetics, and strong compatibility with renewable electricity and industrial waste heat, positioning P-RSOCs as a promising technology for a carbon-neutral energy future. At the heart of these devices lies the proton-conducting electrolyte, which governs proton transport, chemical stability, interfacial compatibility, and long-term durability. Despite remarkable advances in electrolyte development, fundamental challenges in understanding and controlling proton transport, chemical stability, and electrode-electrolyte interactions continue to constrain practical deployment. This review presents a comprehensive and critical assessment of the current state of proton-conducting electrolytes for P-RSOCs, with emphasis on proton transport mechanisms, composition–structure–property relationships, stability limitations, and interfacial compatibility. We examine advances in perovskite-based and emerging alternative electrolyte families, while highlighting key strategies—including aliovalent doping, interfacial engineering, microstructure design, and advanced fabrication—for overcoming persistent limitations. Emerging operando characterization and computational approaches are further discussed as powerful tools for uncovering dynamic transport and degradation mechanisms and accelerating materials discovery. By integrating fundamental insights with materials-design strategies, this review identifies critical knowledge gaps and opportunities to guide the development of robust, high-performance electrolytes. Ultimately, we envision that such advances will help unlock the full potential of P-RSOCs as a versatile platform for sustainable energy conversion, storage, and renewable-fuel production.

## Graphical abstract

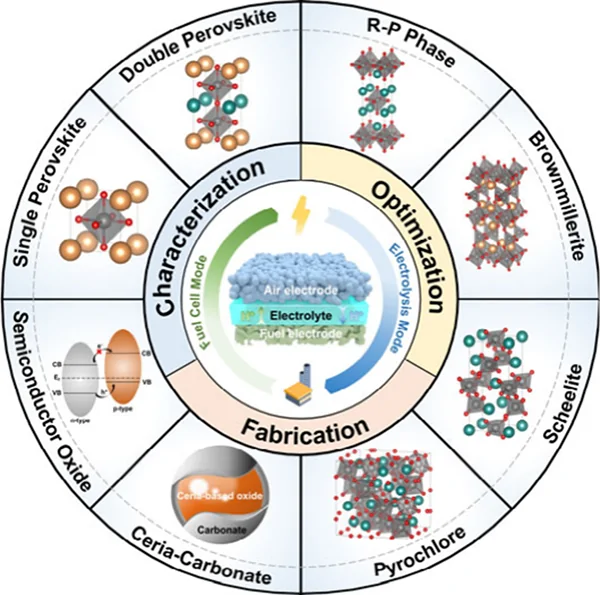

## 1 Introduction

The global transition to a low-carbon energy future is fueling interest in efficient and scalable technologies for energy storage and conversion in clean chemical forms. As the global energy system increasingly integrates renewable electricity sources like wind and solar into the power grid, the demand for energy storage solutions capable of managing fluctuations and balancing supply and demand is becoming increasingly critical.^1,2^ Among emerging technologies, high-temperature electrochemical energy systems—particularly solid oxide cells (SOCs)—stand out for their capability to reversibly interconvert electricity and chemical fuels on a large scale. Owing to their inherent advantages in thermodynamic efficiency, fuel flexibility, and ease of integration with various energy systems, SOCs have garnered significant attention in research and development.^3–6^

Reversible solid oxide cells (RSOCs), capable of operating alternately in fuel cell and electrolysis cell modes, represent a critical enabling platform in the emerging hydrogen economy (Figure 1).^7,8^ In the fuel cell mode, RSOCs generate electricity from hydrogen or other fuels with minimal emissions; in the electrolysis mode, they produce hydrogen or syngas by splitting water or coelectrolyzing H_2_O and CO_2_. This dual functionality allows RSOCs to serve not only as power generators but also as dynamic hubs for energy storage and conversion, effectively stabilizing intermittent renewable energy sources. Their high round-trip efficiency and compatibility with industrial waste heat make them particularly well-suited for deployment in smart grids, chemical production plants, and decentralized energy systems.^8–10^ Moreover, RSOCs offer distinct advantages over low-temperature fuel cell technologies such as alkaline fuel cells, proton exchange membrane fuel cells, and phosphoric acid fuel cells. Operating below 250 °C, these low-temperature systems rely heavily on noble metal catalysts (e.g., Pt) to activate hydrogen. However, these catalysts are highly susceptible to poisoning by common fuel impurities such as CO, necessitating the use of high-purity hydrogen and precluding the direct use of hydrocarbon fuels like natural gas. In contrast, RSOCs, which operate at elevated temperatures, exhibit much greater catalytic activity toward hydrocarbon reforming and enhanced tolerance to contaminants. High operating temperatures enable the use of non-noble metal catalysts and facilitate internal reforming, significantly expanding fuel flexibility.^11^

**Figure 1:**
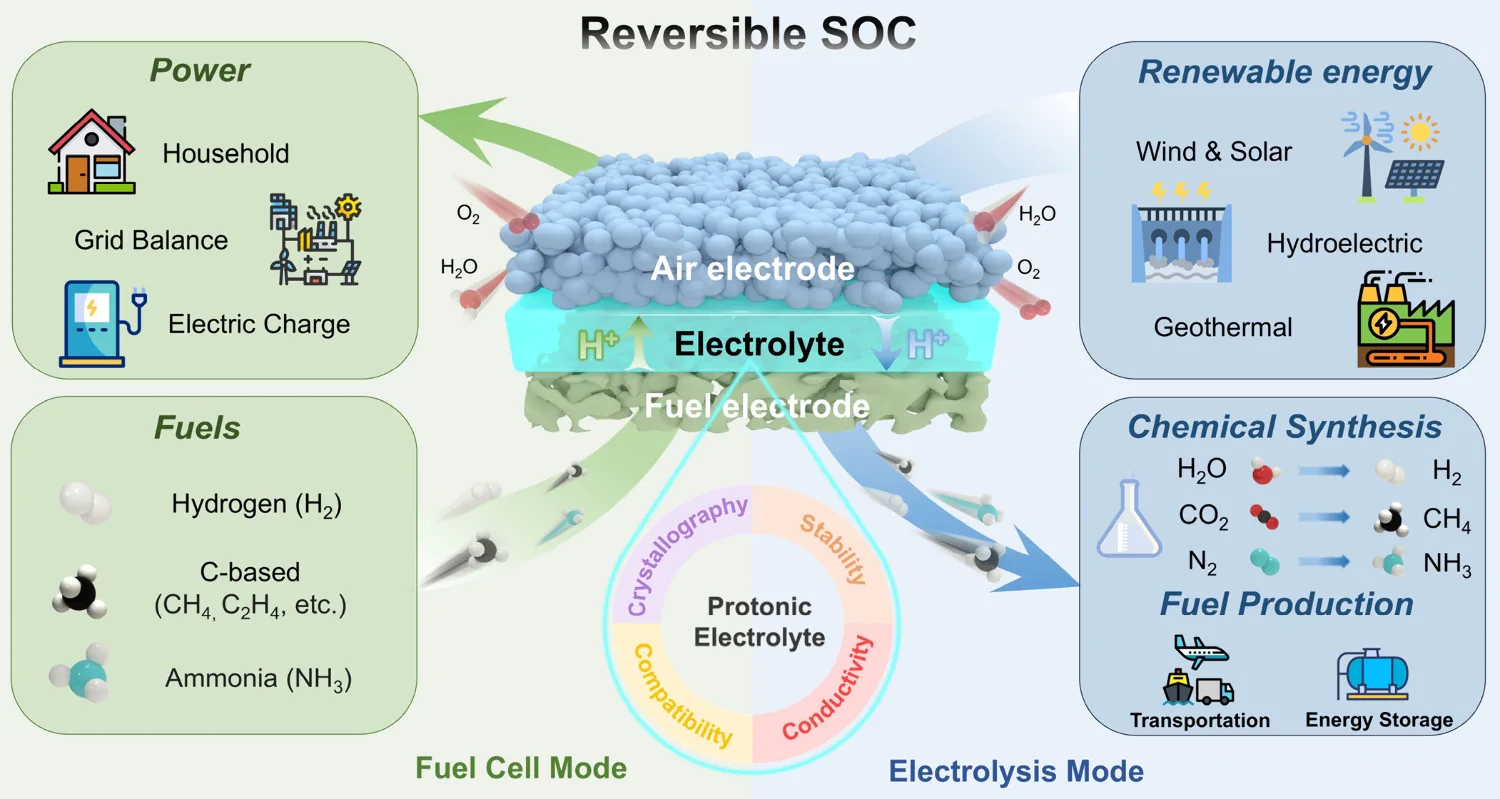
Schematic diagram of a proton-conducting reversible solid oxide cell.

Within this context, P-RSOCs have emerged as a particularly promising variant of SOC technology. In contrast to conventional oxygen-ion conducting SOCs, P-RSOCs employ dense ceramic electrolytes in which proton (H^+^) conduction predominates over oxide ion (O^2–^) conduction. This fundamental difference in charge carriers imparts several system-level advantages. In electrolysis mode, water is supplied to the air electrode, where it dissociates into oxygen gas and protons; the protons then migrate through the electrolyte and recombine at the fuel electrode to generate pure hydrogen. This reaction pathway inherently avoids the formation of hydrogen–steam mixtures, eliminating the need for downstream gas separation and simplifying system design.^12^ In fuel cell mode, hydrogen is oxidized at the fuel electrode to form protons, which are then transported across the electrolyte to the air electrode, where they react with oxygen to form water. Since water is generated at the air electrode side, away from the fuel stream, P-RSOCs facilitate high fuel utilization and offer improved thermal and gas management.^13^

The use of protons as charge carriers offers additional materials and electrochemical advantages. Owing to their small ionic radius and low activation energy for migration, protons exhibit higher mobility than oxide ions, enabling effective operation at intermediate temperatures (400–600 °C).^14–16^ These reduced operating temperatures alleviate thermal stresses and minimize the degradation of cell components, thereby enabling the use of more cost-effective materials and enhancing system longevity. As a result, P-RSOCs are attracting growing interest as viable platforms for intermediate-to-low-temperature (300–500 °C) electrolysis, hydrogen production, carbon-neutral chemical synthesis, and ammonia interconversion.^17,18^

A key component of the P-RSOC system is the proton-conducting electrolyte, which governs key performance metrics such as ionic conductivity, chemical stability, interfacial compatibility, and long-term durability. To ensure efficient operation, the electrolyte must possess high protonic conductivity (>10^–2^ S cm^–1^ at 500 °C), minimal electronic conductivity to maintain high Faradaic efficiency (FE), and robust chemical resistance in steam- or CO_2_-rich environments.^15,19–21^ Additionally, it must be chemically and mechanically compatible with other cell components—including electrodes, interconnects, and sealants—without inducing interfacial reactions that degrade performance. Meeting these stringent requirements poses a formidable materials design challenge and remains a central focus of research and innovation in the P-RSOC field.

Recent years have witnessed substantial progress in the discovery and engineering of proton-conducting electrolyte materials. Perovskite-type oxides—particularly those derived from the BaCeO_3−δ_ (BCO)–BaZrO_3−δ_ (BZO) solid solution system—remain among the most extensively studied due to their high proton conductivity and structural versatility.^22–25^ Doped variants such as BaZr_0.1_Ce_0.7_Y_0.1_Yb_0.1_O_3−δ_ (BZCYYb1711) have demonstrated excellent proton transport properties and electrochemical performance in both fuel cell and electrolysis configurations. However, achieving an optimal balance between proton conductivity and chemical stability remains a persistent challenge. Cerate-rich compositions offer high proton conductivity but are prone to degradation in CO_2_- and H_2_O-rich environments, whereas zirconate-rich materials offer superior chemical resilience at the expense of lower conductivity and poor sinterability.^26^

To overcome these limitations, researchers have investigated advanced doping strategies (e.g., codoping with Y^3+^, Yb^3+^, Gd^3+^, or Sc^3+^), lattice engineering approaches, and the design of bilayer electrolyte architectures that couple a high-conductivity core with a chemically robust outer layer.^27–29^ Concurrently, alternative structural families—such as double perovskites, Ruddlesden–Popper phases, scheelite-type oxides, pyrochlores, ceria-carbonate composites, and semiconductor-based oxides—are being explored to expand the compositional landscape and uncover new structure–property relationships. Many of these materials exhibit favorable hydration behavior and tunable defect chemistry, providing promising pathways for the design of next-generation proton conductors capable of high performance across a wider temperature range.^30^

This surge in materials discovery has been driven by advances in both experimental characterization and computational modeling. Experimental techniques such as impedance spectroscopy, solid-state nuclear magnetic resonance (NMR), neutron scattering, and isotope exchange have yielded detailed insights into proton transport mechanisms, hydration thermodynamics, and dopant–defect interactions. In parallel, first-principles calculations, molecular dynamics (MD) simulations, and machine learning approaches are accelerating the screening and optimization of candidate materials by elucidating structure–property relationships at the atomic scale.

The growing importance of proton-conducting electrolytes extends beyond their roles in fuel cells and electrolysis systems. These materials are increasingly being explored for applications in hydrogen pumps, hydrogen sensors, and membrane reactors for catalytic conversion of light alkanes and ammonia synthesis.^4,31,32^ Their ability to enable efficient hydrogen transport and mediate proton-coupled reactions renders them versatile components in a variety of energy- and chemical-processing technologies.

Given these recent scientific and technological advancements, a comprehensive understanding of proton-conducting electrolyte materials is urgently needed to guide future research and accelerate practical implementation. This review addresses this need by summarizing recent progress in the discovery, characterization, and engineering of proton-conducting electrolytes for RSOCs. Fundamental requirements for high-performance electrolytes, including proton conductivity, chemical and thermal stability, and interfacial compatibility, are first outlined. Major structural families of proton conductors, with an emphasis on perovskites and their derivatives, are then classified and evaluated. Strategies to enhance performance, core characterization methods, and advanced fabrication techniques are then discussed. Finally, key challenges and emerging opportunities are highlighted, including the use of high-throughput computation, machine learning, and integrated experimental–theoretical approaches to accelerate materials innovation. This integrative perspective supports the rational design and scalable application of advanced proton-conducting electrolytes, advancing the deployment of P-RSOCs in next-generation clean energy systems.

## 2 Electrolyte Materials

### 2.1 Basic Requirements

Electrolyte materials form the backbone of P-RSOCs, determining their electrochemical performance, operational longevity, and practical viability. To function effectively, an ideal electrolyte must satisfy several stringent criteria: it should exhibit high proton conductivity with minimal electronic leakage, possess excellent chemical and thermal stability under a diverse range of operating conditions, and maintain a strong interfacial compatibility with both fuel and air electrodes. These stringent requirements have presented significant materials science challenges, driving intensive research over the past three decades, with a notable surge in advancements in the last ten years.

To date, the most extensively studied electrolyte materials for P-RSOCs are perovskite-type oxides with a general formula of ABO_3_.^22,33^ The development of proton-conducting electrolytes has evolved from early BaZrO_3−δ_-based systems toward increasingly complex multication solid solutions. Meanwhile, due to their distinctive proton transport mechanisms and structure–property relationships, several alternative families (Figure 2)—including double perovskites, Ruddlesden–Popper phases, brownmillerites, scheelite-type oxides, pyrochlores, ceria-carbonate composites, and semiconductor-based oxides—have also been widely investigated to broaden the compositional landscape.^30^ This evolution reflects ongoing efforts to balance competing material properties while meeting the practical requirements of SOC fabrication and operation. More recently, research has increasingly focused on tailoring electrolyte materials through defect chemistry and nanostructured interfaces to achieve concurrent improvements in conductivity, stability, and interfacial compatibility.^19^

**Figure 2:**
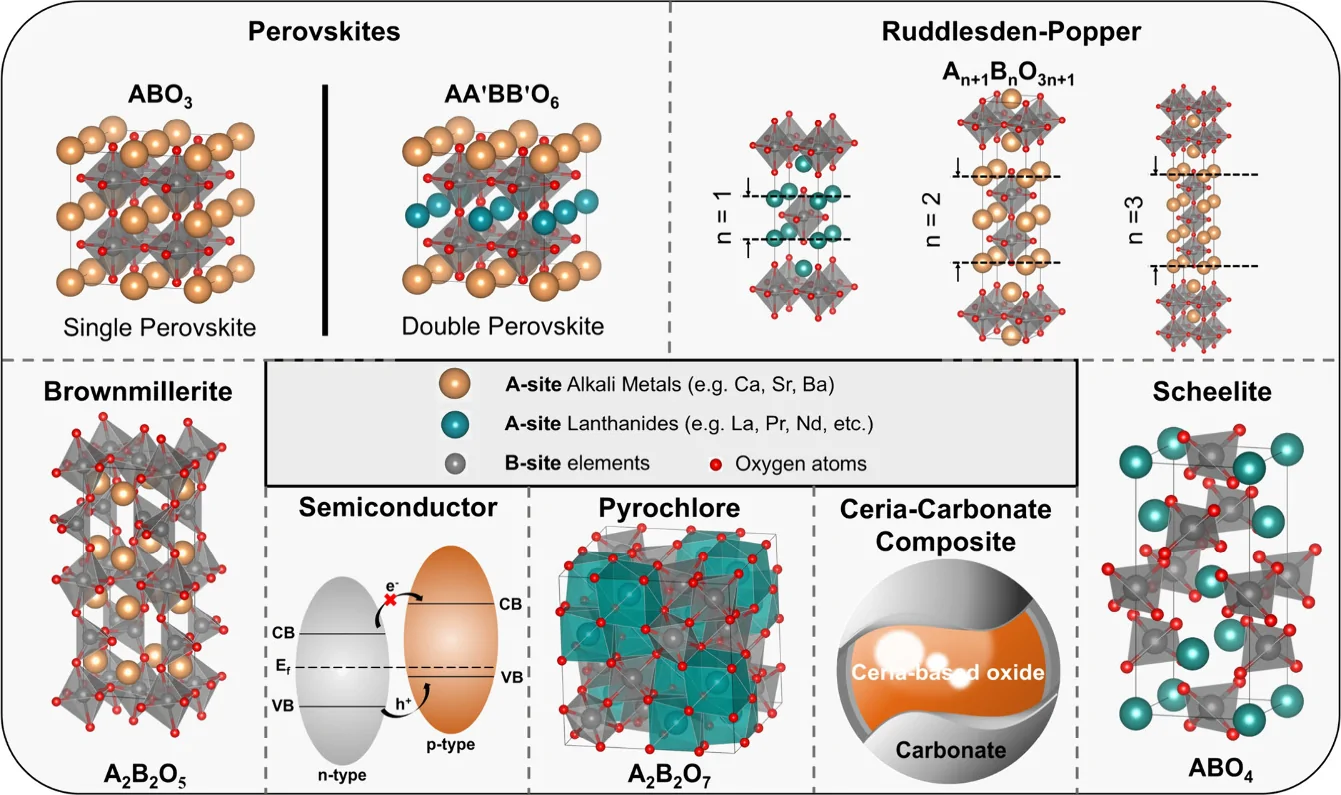
Representative structural motifs of proton-conducting oxide-based materials and illustrations of oxide-related materials.

However, in practical energy conversion systems—including intermediate-temperature solid oxide fuel cells (IT-SOFCs), water electrolysis hydrogen production cells, and electrochemical reactors for conversion of CO_2_ to syngas or ammonia synthesis—the operating conditions (temperature and gas atmosphere) can vary widely.^5,34^ Designing a single electrolyte that performs optimally under all these extreme conditions while simultaneously meeting the three fundamental requirements (proton conductivity, stability, and compatibility) is highly challenging. A more practical approach is to adopt an operating-condition-orientation strategy: select an electrolyte that offers the greatest advantages for the most critical performance parameter in a given scenario (e.g., low-temperature proton conductivity), and then optimize the weaker aspects (e.g., stability).

For instance, proton conductivity may serve as the most critical performance metric for intermediate to low-temperature SOCs. BZCYYb1711, as an excellent proton conductor, is therefore widely adopted as an electrolyte for such devices. However, it is susceptible to degradation in atmospheres with high concentrations of steam or carbon dioxide,^28,35^ limiting its stability in applications such as hydrogen production via electrolysis under high water partial pressure or hydrocarbon–air fuel cells. By ensuring that proton conductivity is not significantly compromised, strategies such as introducing protective layers^28^ and redesigning electrolyte fabrication processes^36^ can enhance the performance of BZCYYb1711-based cells under these complex conditions.

In most application scenarios, ionic conductivity is widely regarded as the primary performance parameter. Table 1 summarizes the key characteristics of proton-conducting electrolytes discussed in the subsequent sections, along with their principal advantages and associated challenges. Variations in intrinsic physicochemical properties of electrolyte materials—such as crystal structure and defect chemistry—can result in markedly different conductive behaviors under diverse operating conditions. By systematically comparing the characteristics and limitations of different electrolyte classes and outlining potential strategies to address these challenges, new opportunities for the development of next-generation proton-conducting electrolytes may be realized.

**Table 1:** Summary of Key Characteristics of the Selected Proton-Conducting Electrolyte^a^

Electrolyte category	Advantage	Disadvantage	Potential application scenarios	Representative materials	Temperature^b^	Conductivity^c^	Atmosphere
Single perovskite	High proton conductivity	★Poor CO_2_ stability	★IT-SOFCs in low-CO_2_ environments	BaZr_0.1_Ce_0.7_Y_0.1_Yb_0.1_O_3−δ_ ^37^	700	5.2 × 10^–2^	Wet O_2_
					600	2.7 × 10^–2^	
					500	1.3 × 10^–2^	
					400	6.2 × 10^–3^	
		★Zr doping reduces sinterability	★Pure H_2_ production electrolyzers	BaZr_0.4_Ce_0.4_Y_0.1_Yb_0.1_O_3−δ_ ^38^	600	2.6 × 10^–2^	N_2_ (3% H_2_O)
					500	2.0 × 10^–2^	
					400	8.2 × 10^–3^	
				La_0.9_Sr_0.1_ScO_3−δ_ ^39^	600	7.4 × 10^–3^	N_2_–H_2_ (2.8% H_2_O)
					500	3.8 × 10^–3^	
					400	1.2 × 10^–3^	
Double perovskite	Excellent CO_2_/H_2_O stability	★Increased electronic leakage at high temperatures	High-humidity and high-CO_2_ electrolyzers	Ba_3_Ca_1.18_Nb_1.52_Y_0.3_O_9−δ_ ^40^	600	5.3 × 10^–3^	Air (3% H_2_O)
		★Moderate proton conductivity at low temperatures			500	2.0 × 10^–3^	
					400	6.0 × 10^–4^	
Ruddlesden–Popper phase	Good hydration capacity and thermal stability	Low conductivity below 700 °C	High-temperature solid oxide fuel cells	BaNd_0.8_Ca_0.2_InO_4−δ_ ^41^	600	5.0 × 10^–4^	Air (3% H_2_O)
					500	1.3 × 10^–4^	
					400	3.0 × 10^–5^	
Brownmillerite	Strong hydration capacity	Structural phase transition-induced mechanical stress	★High-humidity electrolyzers	Sr_2_Sc_0.95_Zn_0.05_GaO_5−δ_ ^42^	630	5.4 × 10^–5^	Wet N_2_
					530	2.0 × 10^–5^	
					430	4.8 × 10^–6^	
			★Oxygen separation membrane coupling systems	Ba_2_LuAlO_5−δ_ ^43^	487	1.0 × 10^–2^	N_2_ (2% H_2_O)
					232	1.5 × 10^–3^	
Scheelite type	Low proton migration energy	Poor long-term stability induced by Li volatilization	Low-temperature micro protonic ceramic cells	LaBaGa_0.7_Li_0.3_O_4−δ_ ^44^	500	5.9 × 10^–3^	N_2_ (3% H_2_O)
					450	4.7 × 10^–3^	
					400	3.2 × 10^–3^	
Pyrochlore type	Excellent CO_2_/H_2_O stability	Obvious electronic leakage	High-humidity and high-CO_2_ electrolyzers	La_1.85_Cs_0.15_Ce_2_O_7−δ_ ^45^	600	6.2 × 10^–4^	H_2_ (3% H_2_O)
					500	7.0 × 10^–5^	
					400	5.3 × 10^–6^	
				La_1.85_Rb_0.15_Ce_2_O_7−δ_ ^45^	600	8.4 × 10^–4^	
					500	1.6 × 10^–4^	
					400	2.9 × 10^–5^	
Ceria-carbonate composite	High conductivity at intermediate-to-low temperatures;	★Poor mechanical strength due to carbonate melting	Low-temperature distributed power systems	CeO_2_–(Li/Na/K)_2_CO_3_ ^46^	550	2.0 × 10^–1^	N_2_ (10% H_2_)
					450	7.0 × 10^–2^	
	Low cost	★Risk of electronic leakage		ZSDC–(K/Na)_2_CO_3_ ^47^	550	8.0 × 10^–2^	H_2_
					500	5.0 × 10^–2^	
					450	3.0 × 10^–2^	
Semiconductor-based oxides	Excellent low-temperature conductivity	★Insufficient long-term stability	Ultralow-temperature protonic ceramic cells	epitaxial H-SmNiO_3_ ^48^	500	7.6 × 10^–3^	Ar (5% H_2_)
					400	4.9 × 10^–3^	
					300	1.5 × 10^–3^	
		★Challenges in cell fabrication		H-SrCoO_2.5_ ^49^	140	3.3 × 10^–1^	H_2_–Ar
					80	8.2 × 10^–2^	
					40	2.8 × 10^–2^	

a Sm_0.1_Zr_0.1_Ce_0.8_O_2−δ_ (ZSDC). (Li/Na/K)_2_CO_3_ and (K/Na)_2_CO_3_ mean a mixing carbonates with Na_2_CO_3_, Li_2_CO_3_, and K_2_CO_3_ in a specific proportion.

b In °C.

c In S cm^–1^.

#### 2.1.1 Conductivity

##### 2.1.1.1 Proton Conductivity

In proton-conducting perovskite oxides, multiple charge carriers can coexist, including the following: 1) protons as lattice hydroxide defects (OH_O_^•^), 2) oxygen vacancies (V_O_^••^), 3) electrons (e′) and electron holes (h^•^) as schematically illustrated in Figure 3a. Under humid atmospheres, water is incorporated through the hydration reaction (eq 1), in which oxygen vacancies are consumed to generate protonic defects.^50^ In parallel, the oxygen sublattice exchanges with the gas phase through redox equilibria (eq 2), where increasing pO_2_ generally depletes oxygen vacancies and promotes p-type electronic defects (eq 3), with holes frequently described as oxygen-site polarons (O_O_). Conversely, strongly reducing conditions (low pO_2_ and/or high pH_2_) shift the defect chemistry toward higher vacancy and electron concentrations (eq 4).^51–53^ The resulting defect populations are governed by mass-action relationships and therefore depend sensitively on temperature and the gas partial pressures of H_2_O, H_2_, and O_2_.

$$H_{2}O_{\left(\mathrm{gas}\right)}+V_{O}^{\cdot \cdot }+O_{O}^{\times }↔2{\mathrm{OH}}_{O}^{\cdot }$$ 1

2 $$O_{2}+2V_{O}^{\cdot \cdot }+2O_{O}^{\times }↔4O_{O}^{\cdot }$$

3 $$O_{2}+2V_{O}^{\cdot \cdot }↔2O_{O}^{\times }+4h^{\cdot }$$

$$H_{2}+2O_{O}^{\times }↔2{\mathrm{OH}}_{O}^{\cdot }+2e^{\prime }$$ 4

**Figure 3:**
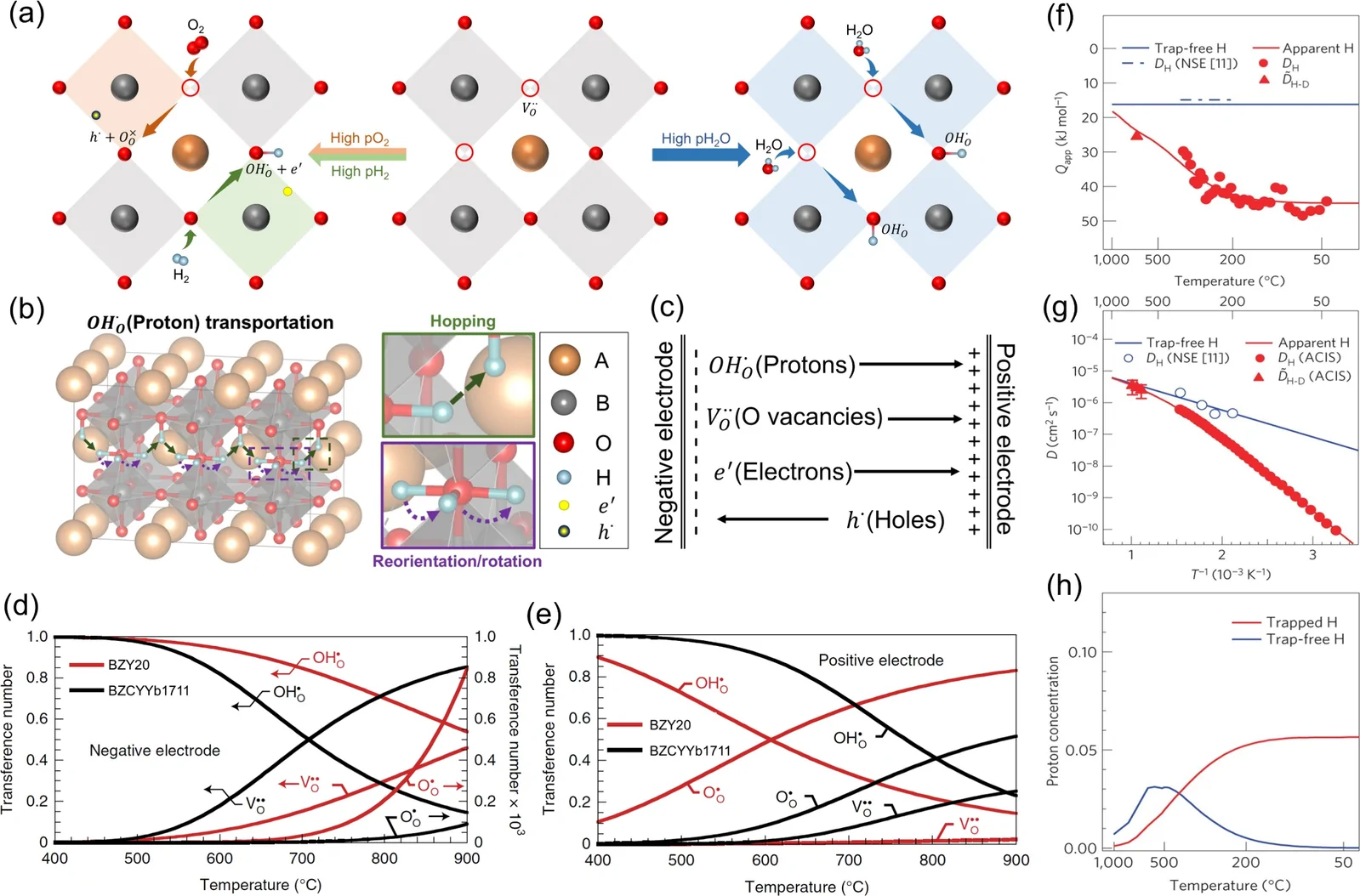
Schematic of (a) charge carrier formation in perovskite oxides, (b) the proton conduction mechanism for acceptor-doped ABO_3_ perovskite oxides, and (c) constituent components of total conductivity. Predicted equilibrium transference numbers as a function of temperature for BZCYYb1711 and BZY20 under (d) negative electrode conditions and (e) positive electrode conditions. Reproduced with permission from ref 61. Copyright 2019 Springer Nature. (f) Long-range proton diffusivity displays a characteristic downward curvature. Red circles and triangles represent the proton diffusivity and the ambipolar diffusivity (proton–deuterium), respectively, as measured by AC impedance spectroscopy. The trap-free proton diffusivity predicted by the proton–dopant association (trapping) model (blue line) is in good agreement with values obtained from neutron spin–echo measurements (blue open circles). (g) Apparent activation energies for proton diffusion are presented alongside the constant activation energy corresponding to trap-free (association-free) proton transport. (h) Trapped and trap-free proton concentrations are calculated using the proton–dopant association model and are compared with the experimentally determined total proton concentration. Reproduced with permission from ref 63. Copyright 2013 Springer Nature.

The protonic conduction mechanism involves two key steps: proton incorporation via hydration reactions at oxygen vacancies, followed by proton migration through the oxide lattice.^54^ Recent studies have provided valuable atomistic insights into these processes, revealing new strategies for enhancing proton mobility and overcoming transport-limiting bottlenecks. Fujii et al.^55^ showed that proton transport in Y-doped BaZrO_3−δ_ is strongly influenced by dopant configuration, with isolated Y dopants—typical of equilibrium distributions—reducing proton trapping and enhancing diffusivity. Their atomistic simulations revealed that Y–H interactions dominate the migration barrier landscape, while H–H interactions and preferential pathways are less significant. These results highlight dopant isolation as a key strategy for improving proton mobility in perovskite electrolytes.^55^

As shown in Figure 3b, the mechanism of proton conduction in perovskite oxides mainly relies on two principal processes: proton hopping between adjacent oxygen ions (the Grotthuss mechanism) and the rotational diffusion of protonic defects.^52,56,57^ This mechanism gives rise to relatively high proton mobility with a typical activation energy of 0.4–0.6 eV in BaZrO_3−δ_/BaCeO_3−δ_ systems.^58^ The transport behavior is fundamentally controlled by the material’s defect chemistry, which is largely dictated by the type and concentration of acceptor dopants. Trivalent cations are commonly employed to substitute for tetravalent B-site cations (Ce^4+^ or Zr^4+^), generating oxygen vacancies that act as incorporation sites for protons.^59,60^ Traversa et al.^60^ showed that doping BaZrO_3−δ_ with trivalent cations like Y^3+^, Yb^3+^, and Gd^3+^ creates oxygen vacancies for proton incorporation, with the dopant ionic radius critically influencing proton mobility. Dopants with radii close to Zr^4+^ minimize proton trapping and enhance conductivity, while size mismatch leads to defect clustering or site misplacement, reducing effective proton transport. In contrast, another model for proton transport, wherein protons migrate in association with O^2–^ was proposed. This model suggests that proton mobility is facilitated through the concerted motion of larger oxide ions, effectively allowing protons to be transported as part of a composite defect species. However, given the relatively large ionic radius and mass of O^2–^, this mechanism is typically associated with a high activation energy barrier, rendering it less favorable compared to the Grotthuss mechanism model of proton conduction.

Because each mobile carrier contributes a partial conductivity, the experimentally measured total conductivity represents the sum of protonic, oxide-defect–mediated, and electronic components. The corresponding transference numbers quantify the fractional contribution of each carrier type under specific operating conditions (Figure 3c). Accordingly, an “ideal” proton conductor is typically characterized by a proton transference number (t_H^+^_) approaching unity across the relevant temperature and atmosphere window. Duan et al.^61^ theoretically evaluated carrier-specific transference numbers for state-of-the-art BaZr_0.8_Y_0.2_O_3−δ_ (BZY20) and BZCYYb1711 under both fuel cell and electrolysis modes operating at ± 1 A cm^–2^ (Figure 3d,e). Their results underscore that both temperature and operating conditions can substantially redistribute the dominant charge carrier.

At the atomistic level, first-principles calculations by Lee et al.^62^ further showed that proton transport in Y-doped BaZrO_3−δ_ is governed by defect formation energies, hydration thermodynamics, and local dopant–defect interactions: Ba-site vacancies can enhance proton incorporation but also introduce trapping configurations that limit proton mobility, while the strong configurational dependence of hydration energetics highlights the critical roles of lattice distortion and defect clustering.

Recent research has highlighted the critical role of the local coordination environment around dopant cations in shaping proton-trapping behavior. As shown in Figure 3f–h, a study by Yamazaki et al.^63^ provided critical insight into the mechanisms governing proton transport in BZY20, emphasizing the role of proton trapping in limiting long-range conductivity. Through a combination of thermogravimetric analysis (TGA), alternating current (AC) impedance spectroscopy, and high-temperature solid-state ^1^H NMR, the study demonstrated that protons in BZY20 do not migrate freely but are partially immobilized due to association with yttrium dopant sites. This trapping behavior was quantitatively described using a two-state defect model, from which the authors extracted a proton–dopant association energy of −29 kJ mol^–1^ and a trap-free proton migration barrier of 16 kJ mol^–1^. These values explain the observed non-Arrhenius behavior in proton diffusivity at elevated temperatures, which deviates from the linear dependence expected in purely trap-free conduction.^63^ Complementary NMR measurements revealed the presence of two distinct proton environments at elevated temperatures, which were assigned to trapped and trap-free protons, respectively. The good agreement between the NMR-derived site populations and the predictions of the trapping model reinforces the interpretation that local chemical environments around Y dopants strongly influence proton mobility. The authors further showed that, while most protons remain associated with Y–O–M (M = Y, Zr) configurations, a fraction can thermally access less strongly bound environments and contribute to long-range conduction. This study established a direct link between dopant chemistry, local defect interactions, and macroscopic transport behavior. It also highlighted the potential for significant improvements in proton conductivity by reducing the association energy through optimized dopant selection. Specifically, a decrease in the proton–dopant binding energy from −29 to −20 kJ mol^–1^ would enable a 2-fold increase in conductivity at 350 °C, with substantial implications for lowering the operating temperature of protonic ceramic fuel cells (PCFCs) while maintaining target area-specific resistance (ASR).

The crystal structure of the electrolyte is equally vital in determining proton transport. Cubic perovskite phases typically exhibit superior proton mobility relative to lower-symmetry phases, owing to their more isotropic oxygen ion environments. The study by Eriksson Andersson et al.^64^ provides a detailed structural analysis of the proton-conducting perovskite BaCe_0.8_Y_0.2_O_3−δ_ (BCY20) using high-resolution neutron diffraction under both dry and humid atmospheres. It reveals that BCY20 undergoes a sequence of temperature-dependent phase transitions—cubic (Pm3m) to rhombohedral (*R*3*c*), orthorhombic (*Imma*), and eventually monoclinic (I2/m)—with the transition temperatures strongly influenced by hydration. Hydration not only stabilizes higher-symmetry structures to higher temperatures but also induces significant chemical expansion and octahedral tilt suppression due to proton incorporation. Notably, deuteron sites were directly identified in both *R*3*c* and *Imma* phases, and their presence correlated with structural modifications supporting enhanced proton mobility. These findings emphasize the strong coupling between protonic defects, structural symmetry, and transport properties in perovskite electrolytes, underlining the importance of *in situ* structural analysis in understanding and optimizing proton conduction mechanisms.^64^

Hydration thermodynamics is another key factor governing proton conductivity. The hydration equilibrium constant, K_W_, for the water incorporation reaction can be written as in eq 5. Since the concentration of regular lattice oxygen [O_O_^×^] is effectively constant and close to unity, this expression is commonly simplified, leading to the Van’t Hoff relation (eq 6). The water incorporation reaction (eq 1) is strongly exothermic, characterized by a negative hydration enthalpy.^65^ As a result, increasing temperature reduces K_W_, shifting the equilibrium toward dehydration and lowering the concentration of protonic defects [OH_O_^•^]. Consequently, the equilibrium proton concentration decreases with increasing temperature, directly impacting proton conductivity.

The thermodynamics of these reactions (eqs 1–4) favor high proton concentrations at lower temperatures. However, this must be balanced against the thermally activated nature of proton mobility, which defines an optimal operating temperature window, typically 400–600 °C, for these electrolytes. Because hydration is exothermic, higher temperatures reduce the equilibrium constant, promoting dehydration. This makes protonic defects thermodynamically unstable and leads to dissociation. Proton-conducting perovskites begin to dehydrate above ∼ 600 °C,^66^ resulting in lower proton concentration and decreased conductivity. In addition, parasitic electronic conduction becomes significant at elevated temperatures, reducing the ionic transference number (t_i_) and overall cell performance.

Conversely, although these materials remain well hydrated at temperatures below ∼ 400 °C, proton mobility is limited by the high activation energy for proton migration, resulting in inadequate conductivity. Therefore, the temperature range of 400–600 °C represents a regime in which both proton concentration and mobility are simultaneously sufficient, while electronic leakage remains minimal. Recent studies on heavily doped systems, such as BaZr_0.4_Sc_0.6_O_3−δ_, demonstrate that appropriate dopant selection can enhance both proton concentration and mobility. Hyodo et al.^67^ reported that 60 at% Sc doping yields a high proton concentration (0.55 mol per formula unit) and a reduced diffusion activation energy (41.4 kJ mol^–1^), thereby broadening the operational window for intermediate-temperature applications.

5 $$K_{W}=\frac{{\left[{\mathrm{OH}}_{O}^{\cdot }\right]}^{2}}{\left[V_{O}^{\cdot \cdot }\right]\cdot \left[O_{O}^{\times }\right]\cdot p_{H_{2}O}}$$

$${\mathrm{lnK}}_{W}=-\frac{\Delta_{H_{w}}}{\mathrm{RT}}+\frac{\Delta_{S_{w}}}{R}$$ 6

Changes in the operating environment lead to significant variations in ionic conduction behavior. Under humid conditions, protons are the dominant charge carriers in perovskite oxides, and the total conductivity is governed by proton transport, with only a minor contribution from oxide ions. In contrast, when the atmosphere is dried (low pH_2_O), or the oxygen partial pressure (pO_2_) is increased, the protonic contribution decreases sharply. Quantitatively, at 600 °C, the proton conductivity of BaZrO_3−δ_-based electrolytes can drop by several orders of magnitude upon switching from humid to dry gas.^68^

Under these conditions, charge transport increasingly relies on oxide-ion conduction and any available electronic carrier. While this might increase the total conductivity, it occurs at the expense of internal short-circuiting currents, which degrade the electrochemical efficiency. In fuel cell operation, any electronic conduction reduces the open-circuit voltage (OCV) below the Nernst potential due to electronic leakage through the electrolyte. Conversely, in the electrolysis mode, electronic leakage reduces FE, as not all electrons contribute to the desired electrochemical reactions. Kim et al.^69^ provided experimental evidence that in BZCYYb1711, proton conduction dominates under humid, reducing conditions, whereas mixed conduction arises at higher oxygen partial pressure (pO_2_) or elevated temperatures. Their quantitative analysis of partial conductivities and equilibrium constants demonstrated that ionic transport is governed by the interplay among hydration thermodynamics, defect equilibria, and ambient conditions, underscoring the coupled structural and chemical factors that control the conductivity in proton-conducting perovskites.^69^

##### 2.1.1.2 Ionic Transference Number

The t_i_ is a key performance metric for electrolytes, representing the fraction of the total current through the electrolyte carried by ionic species. For optimal operation, electrolyte materials should exhibit transference number values approaching unity to minimize electronic leakage currents, which can diminish the FE of electrolysis and energy efficiency of fuel cells. Choi et al.^20^ showed that in BaZr_0.4_Ce_0.4_Y_0.1_Yb_0.1_O_3−δ_ (BZCYYb4411), the t_H^+^_ decreases from 0.985 at 500 °C to 0.936 at 650 °C, leading to increased electronic leakage and reduced FE during electrolysis. Their findings highlight the importance of developing electrolytes with proton transference numbers close to one to ensure efficient current utilization.

In proton-conducting perovskites, electronic conductivity primarily arises from two competing defect reactions: the hydration reaction (eq 1) and the oxidation of oxygen vacancies (eq 3). Under oxidizing conditions at elevated temperatures (>600 °C), oxygen vacancy oxidation becomes dominant, resulting in substantial p-type electronic conduction.

Advances in understanding these competing processes have enabled the development of several strategies to enhance t_i_. Lowering the operating temperature (<500 °C) favors the hydration reaction while suppressing oxygen vacancy oxidation. Additionally, maintaining high steam partial pressures in electrolysis cells can effectively shift the defect equilibrium toward protonic defects, thereby reducing electronic leakage.

Significant progress has also been made in developing accurate characterization methods for determining t_i_. Although conventional direct current (DC) polarization techniques are still widely employed, they can substantially underestimate electronic conductivity in mixed conductors. More advanced methods, such as combining electromotive force (EMF) measurements with isotopic labeling, have enabled more precise quantification of charge transport behavior.^70,71^ As shown in Figure 4, Han et al.^66^ systematically investigated the impact of cerium content on the transport properties of BaZr_0.8–x_Ce_x_Y_0.2_O_3−δ_, with particular emphasis on the t_i_ and its role in electrochemical performance. Their results revealed that increasing the Ce content not only enhances proton concentration and oxide ion mobility but also significantly suppresses hole conduction under oxidizing conditions. This suppression of electronic (hole) transport leads to a marked increase in the t_i_ across the BaZr_0.8–x_Ce_x_Y_0.2_O_3−δ_ series, particularly in compositions with higher Ce content. The improved ionic selectivity arises from both thermodynamic and kinetic factors. Density functional theory (DFT) calculations indicated that Ce substitution reduces the binding strength between Y dopants and oxygen vacancies, facilitating oxide ion mobility while minimizing defect trapping.^66^ At the same time, the increased hydration enthalpy associated with cerium-rich compositions increases the concentration of mobile protons. Together, these effects yield a higher proportion of current carried by ionic species (both H^+^ and O^2–^), thereby increasing the t_i_ and improving electrochemical efficiency in device operation. This work highlights the critical role of controlling mixed conduction in protonic ceramic electrolytes and demonstrates that tuning the B-site cation ratio offers a viable strategy for maximizing ionic transport while minimizing electronic leakage—an essential criterion for achieving high FE in both fuel cell and electrolysis modes.^66^

**Figure 4:**
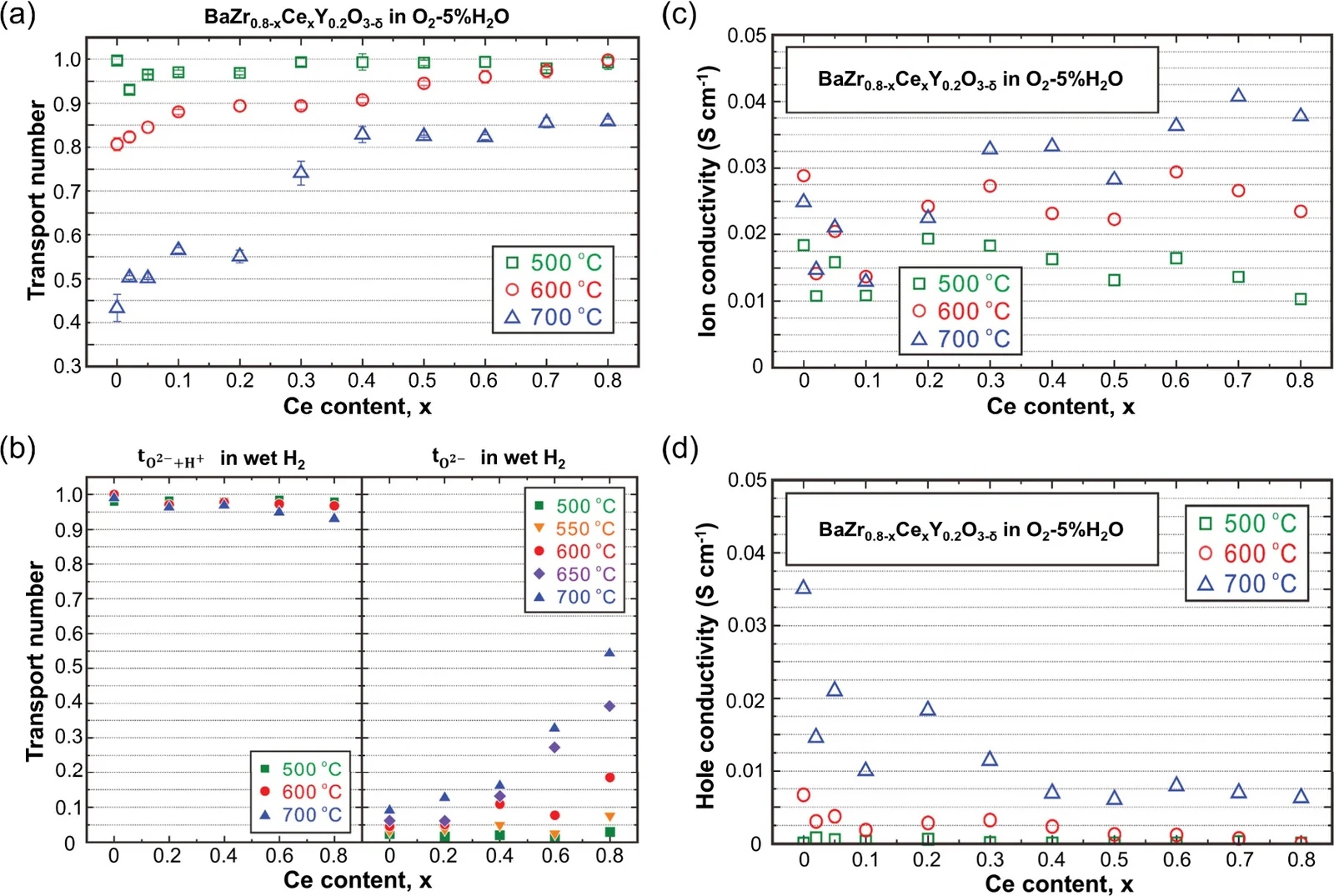
(a) Ionic transport numbers (combined O^2–^ and H^+^ contributions) for BaZr_0.8–x_Ce_x_Y_0.2_O_3−δ_, determined by fitting the total conductivity as a function of oxygen partial pressure (pO_2_) at a fixed water vapor pressure (pH_2_O = 0.05 atm). (b) Ionic transport numbers, including total ionic conduction (O^2–^ + H^+^) and H^+^ conduction, measured under wet H_2_ conditions using the EMF method. (c) Ionic conductivity and (d) hole conductivity of BaZr_0.8–x_Ce_x_Y_0.2_O_3−δ_ with varying Ce content, extracted by simulating the dependence of total conductivity on pO_2_ in wet O_2_ (pH_2_O = 0.05 atm). Reproduced with permission from ref 66. Copyright 2021 Wiley.

#### 2.1.2 Stability

The long-term stability of proton-conducting electrolytes under operational conditions remains one of the most critical challenges for the commercial deployment of SOC systems. Stability encompasses chemical resistance to reactive gas species, tolerance to thermal cycling, and mechanical integrity under operational stress. Recent research has focused on developing comprehensive stability assessment protocols and unraveling degradation mechanisms at the atomic scale, providing essential insights into materials optimization.

##### 2.1.2.1 Chemical Stability

Inherently, proton conductors with alternative crystal structures often exhibit excellent chemical stability against contaminants such as CO_2_ and H_2_O, making them attractive from a durability standpoint. However, their low conductivity remains a major bottleneck. As a result, extensive efforts have been directed toward improving the chemical stability of barium-based perovskite oxides—particularly BaCeO_3−δ_-based systems—without incurring significant trade-offs in proton conductivity. Chemical stability against CO_2_ and steam is perhaps the most extensively investigated aspect of proton-conducting electrolyte durability.^72^ Each reactive species triggers distinct degradation pathways, posing unique challenges for materials design. Advances in *in situ* characterization techniques have recently enabled unprecedented resolution in tracking these degradation processes. In CO_2_-containing environments, barium-cerate-based materials decompose via the reaction (eq 7) as follows.

7 $${\mathrm{BaCeO}}_{3}+{\mathrm{CO}}_{2}\to {\mathrm{BaCO}}_{3}+{\mathrm{CeO}}_{2}$$

Thermodynamic analyses show that this reaction proceeds spontaneously below ∼ 700 °C for undoped BaCeO_3−δ_, with the exact stability threshold influenced by dopant composition and CO_2_ partial pressure. Kim et al.^73^ showed that Y-doped BaCeO_3−δ_ decomposes into BaCO_3_ and CeO_2_ upon CO_2_ exposure at ∼ 800 °C, while BaZrO_3−δ_-based compositions remain stable. Thermogravimetric and structural analyses confirmed that Zr substitution significantly enhances CO_2_ resistance, consistent with thermodynamic predictions across BaCe_1–x_Zr_x_O_3−δ_ systems.

In steam environments, degradation follows a distinct pathway (eq 8) through the formation of barium hydroxide species. This process is particularly problematic at intermediate temperatures, where steam partial pressures are high, yet temperatures are insufficient for rapid hydroxide decomposition. Saz̆inas et al.^74^ provided clear evidence that secondary phase formation in BaZr_1–x_Y_x_O_3−δ_ begins at grain boundary sites upon CO_2_ exposure. Scanning electron microscopy (SEM) and energy-dispersive X-ray spectroscopy (EDS) mapping revealed the emergence of Ba-deficient and Y-enriched regions localized at grain boundary sites, coinciding with the gradual formation of surface BaCO_3_. These microstructural changes were accompanied by cation redistribution, where Y was observed to migrate from Zr to Ba sites, destabilizing the perovskite lattice locally. This supports that grain boundaries act as preferential sites for chemical degradation, highlighting the need for microstructural control—such as grain boundary passivation or dopant segregation management—to mitigate long-term instability in proton-conducting perovskites.^65,74,75^

8 $${\mathrm{BaCeO}}_{3}+H_{2}O\to \mathrm{Ba}{\left(\mathrm{OH}\right)}_{2}+{\mathrm{CeO}}_{2}$$

Recent materials development efforts have also explored alternative B-site substitutions to improve chemical stability. Hafnium-containing compositions, such as BaHf_x_Ce_0.8–x_Y_0.1_Yb_0.1_O_3−δ_, have shown particular promise, delivering both enhanced CO_2_ stability and preserved proton conductivity.^21^ Computational studies attribute this improvement to the stronger Hf–O bonds relative to Zr–O or Ce–O, which raise the activation barrier for decomposition reactions (Figure 5).

**Figure 5:**
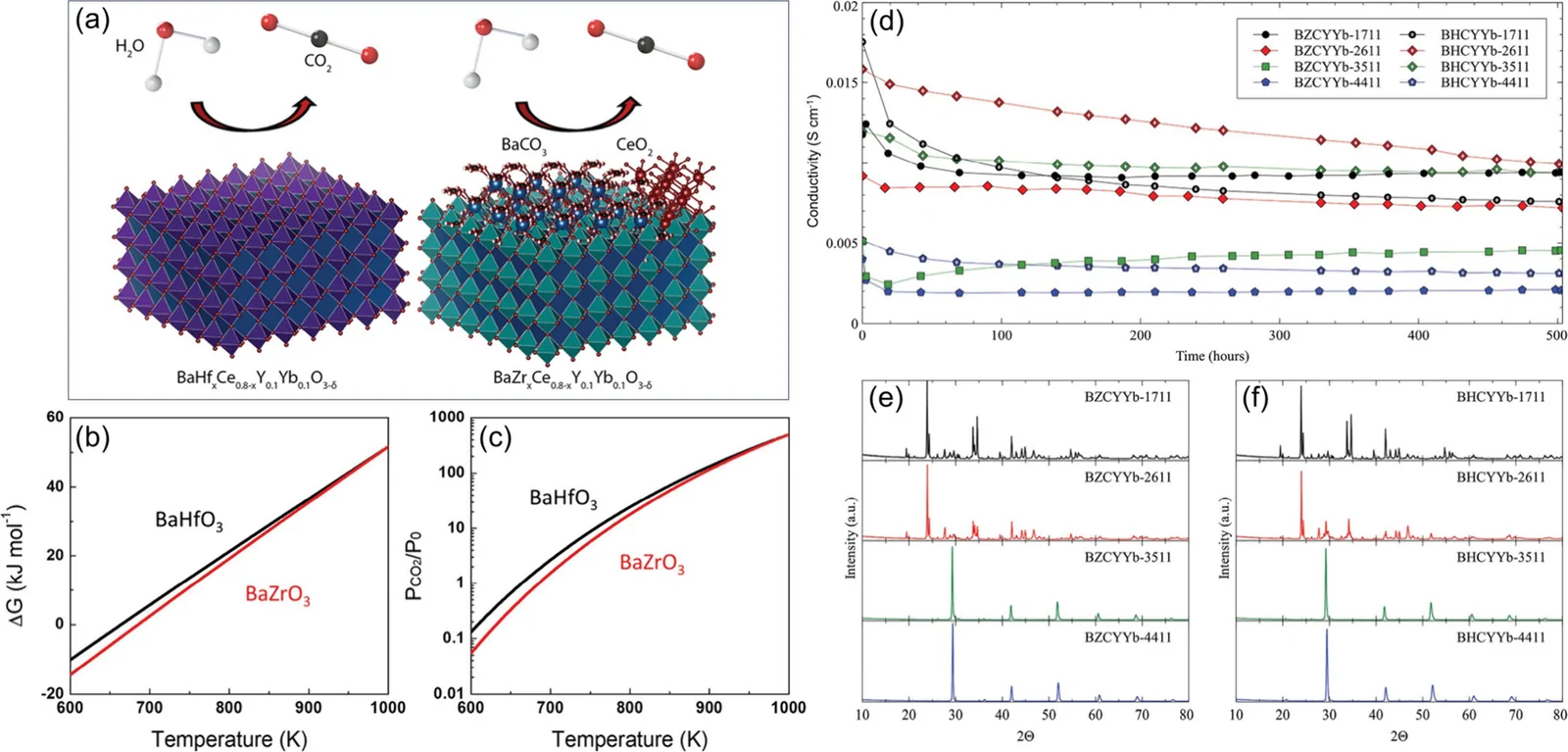
(a) Schematic illustration of degradation pathways involving reactions with CO_2_ and H_2_O. (b) Gibbs free energy change for the reaction between BaMO_3_ and CO_2_, forming BaCO_3_ and MO_2_ (M = Zr, Hf), indicating the relative thermodynamic stability. (c) Van’t Hoff plot for the reaction of BaMO_3_ with CO_2_, highlighting temperature dependence. (d) Time-dependent conductivity of BaZr_x_Ce_0.8–x_Y_0.1_Yb_0.1_O_3−δ_ (BZCYYb) and BaHf_x_Ce_0.8–x_Y_0.1_Yb_0.1_O_3−δ_ (BHCYYb) measured over 500 h in a gas atmosphere containing 25% CO_2_, 25% H_2_O, and 50% H_2_ at 700 °C. X-ray diffraction (XRD) patterns of (e) BZCYYb and (f) BHCYYb, respectively, after 500 h exposure to the same CO_2_/H_2_O/H_2_ atmosphere at 700 °C, revealing phase evolution and chemical stability. Reproduced with permission from ref 21. Copyright 2020 Wiley.

Microstructural engineering is another critical strategy in enhancing chemical resilience. Dense, fine-grained microstructures reduce the effective surface area susceptible to degradation, while the elimination of open grain boundaries mitigates pathways for CO_2_ or H_2_O infiltration. Control over sintering profiles, powder morphology, and dopant homogeneity is essential to achieve the desired microstructural attributes.^76^ Moreover, recent innovations in bilayer electrolyte architectures have shown great promise. These designs feature a thin, chemically stable outer layer—such as BaHf_0.8_Yb_0.2_O_3−δ_ (BHYb82)—deposited atop a high-conductivity core like BZCYYb1711. The chemically robust top layer acts as a barrier to gas infiltration, effectively passivating the underlying proton conductor while preserving its superior transport properties. These bilayers, fabricated using cosputtering, chemical vapor deposition (CVD), or other scalable thin-film techniques, have demonstrated minimal degradation over hundreds of hours under wet CO_2_ environments and exhibit outstanding electrochemical performance.^28,77,78^ Qian et al.^78^ developed a BZY20/BCY20 bilayer electrolyte using pulsed laser deposition (PLD) to enhance CO_2_ stability in an anode-supported solid oxide fuel cell (SOFC). They showed that increasing the BZY20 layer thickness improved resistance to CO_2_-induced degradation, with a 3.6 μm BZY20 layer maintaining structural integrity after exposure to pure CO_2_ at 900 °C. Although thicker BZY20 layers reduced power density due to lower conductivity and increased interfacial resistance, the bilayer maintained high OCV and adequate performance. These results highlight the effectiveness of protective layers in improving chemical stability while balancing electrochemical performance.

##### 2.1.2.2 Thermal Stability

Thermal stability in proton-conducting electrolytes encompasses both phase stability across operational temperature ranges and the sintering behavior during cell fabrication. Recent advances have focused on elucidating phase transformation mechanisms, optimizing sintering processes, and improving thermal cycling resilience to enhance the overall durability of the solid oxide cells.

Phase stability is a key concern, particularly for cerate-based materials that undergo multiple structural transitions with temperature. For example, BCY20 exhibits three distinct polymorphs: monoclinic (room temperature–400 °C), orthorhombic (400–600 °C), and cubic (>600 °C). These phase transitions can induce volume changes and associated mechanical stresses during thermal cycling, potentially compromising mechanical integrity. Recent research has explored chemical substitution strategies, notably with Zr dopant, to stabilize the cubic phase at lower temperatures.^64^

Sintering behavior presents another critical challenge, particularly for BZO-based materials, which typically require sintering temperatures exceeding 1600 °C to achieve adequate densification. Such high temperatures can cause barium evaporation and lead to stoichiometric deviations, undermining material performance. VahidMohammadi et al.^79^ showed that BZCYYb1711 requires sintering temperatures above 1500 °C to achieve high density, during which substantial barium loss occurs via solid-state reaction (SSR) and vapor-phase evaporation. This results in stoichiometric imbalance, phase decomposition, and poor densification unless sintering is carefully controlled with protective layering or sintering aids such as NiO. Similarly, Choi et al.^80^ confirmed that sintering above 1400 °C leads to significant Ba evaporation, which disrupts stoichiometry, promotes secondary phase formation, and severely degrades conductivity and grain growth. They demonstrated that controlling the local Ba chemical potential during sintering preserves stoichiometry and enables larger grain sizes and higher proton conductivity. These findings underscore that the high-temperature sintering required for BaZrO_3−δ_-based materials induces barium volatility and structural degradation and that controlling Ba loss is essential for achieving stable, high-performance proton-conducting electrolytes.^80^ To address the high sintering temperatures typically required for densification, Liu et al.^81^ systematically evaluated the effect of adding ∼ 1 wt % NiO as a sintering aid. The introduction of NiO substantially improved sinterability, enabling >96% theoretical density after sintering at 1350 °C for 3 h, compared to 1550 °C for 10 h without additives. X-ray diffraction confirmed the preservation of the perovskite phase, with the formation of a minor BaY_2_NiO_5_ secondary phase at the grain boundaries. SEM analyses revealed enhanced densification and grain growth, attributed to accelerated mass transport and possible liquid-phase-assisted sintering. Importantly, electrical characterization showed that the total conductivity of NiO-modified BZCYYb remained comparable to that of the unmodified sample, despite the presence of secondary phases. Open-circuit voltage measurements under fuel cell conditions yielded high t_i_ (∼0.96 at 650 °C), indicating minimal electronic leakage. These results demonstrate that NiO is an effective sintering aid for BZCYYb, enabling lower processing temperatures without compromising electrolyte performance in protonic ceramic fuel cells (Figure 6).^81^ More recent work has explored alternative sintering aids such as CuO and ZnO.^82^ Advanced sintering techniques, including cold sintering and field-assisted sintering, have further reduced processing temperatures while maintaining high densification. Kindelmann et al.^83^ demonstrated that cold sintering enables significant densification of BaZr_0.7_Ce_0.2_Y_0.1_O_3−δ_ (BZCY721) at temperatures as low as 250–350 °C by tailoring the phase composition of the starting powders. By optimizing the presence of a Ce-rich phase, they achieved high density and conductivity (∼10^–3^ S cm^–1^ at 500 °C) after a moderate post-thermal treatment. This confirms that advanced sintering methods, such as cold sintering, can effectively reduce processing temperatures while maintaining high electrolyte performance.

**Figure 6:**
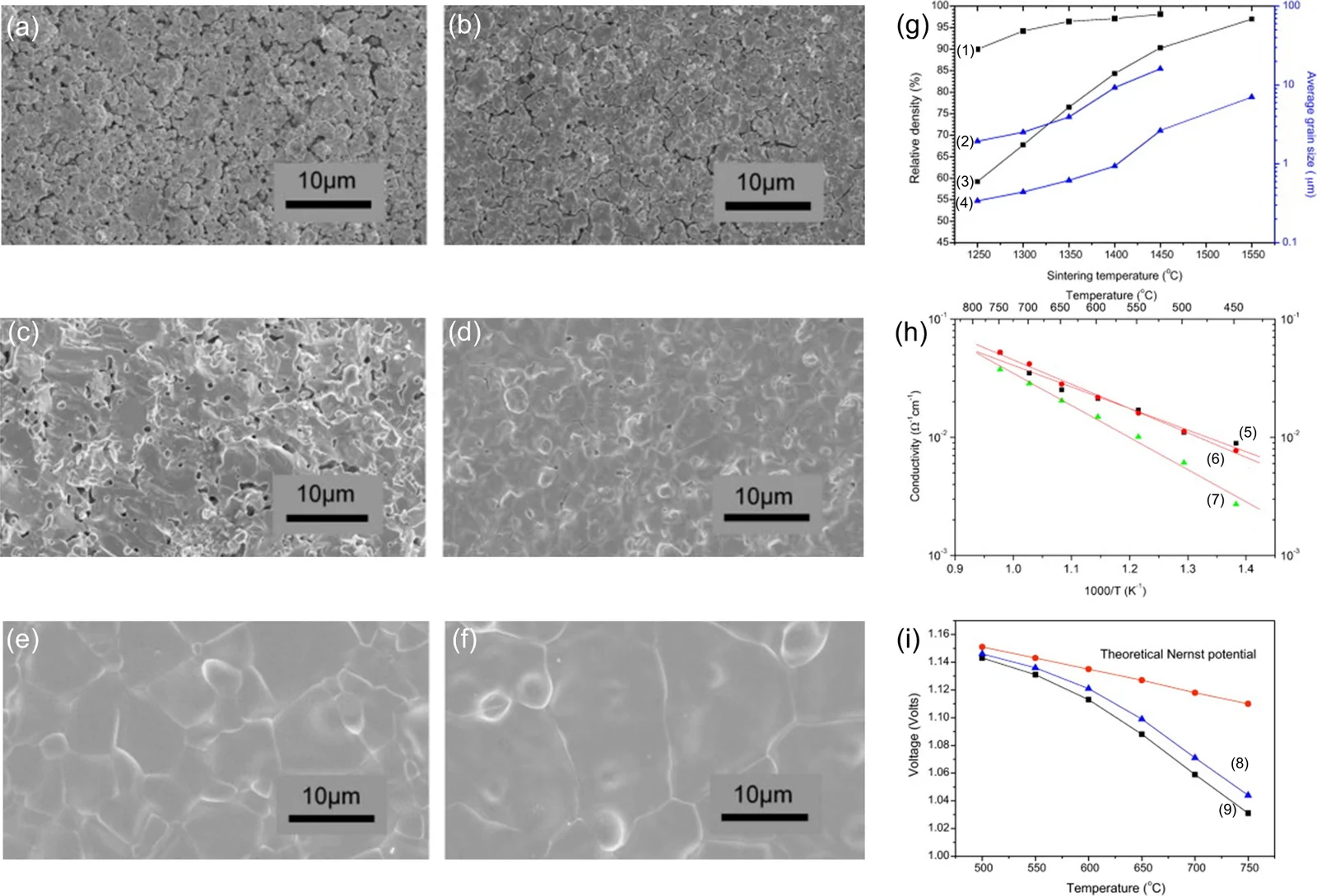
SEM images of BZCYYb1711 and 1 wt % NiO-modified BZCYYb1711 pellets sintered under various conditions: (a) pristine BZCYYb1711 sintered at 1350 °C for 3 h, (b) 1% NiO–BZCYYb1711 at 1250 °C for 3 h, (c) pristine BZCYYb1711 at 1450 °C for 3 h, (d) 1% NiO–BZCYYb1711 at 1350 °C for 3 h, (e) pristine BZCYYb1711 at 1550 °C for 10 h, and (f) 1% NiO–BZCYYb1711 at 1450 °C for 3 h. (g) Relative densities and average grain sizes of NiO-modified (1 and 2) and unmodified (3 and 4) BZCYYb1711 pellets sintered at various temperatures for 3 h. (h) Electrical conductivities of BZCYYb1711 and 1% NiO–BZCYYb1711 pellets sintered under different conditions: (5) pristine BZCYYb1711 at 1550 °C for 10 h, (6) 1% NiO–BZCYYb1711 at 1450 °C for 3 h, and (7) 1% NiO–BZCYYb1711 at 1350 °C for 3 h. (i) Open-circuit voltages of Pt|electrolyte|Pt cells operated with wet H_2_ (∼3 vol % H_2_O) as the fuel and air as the oxidant, using different electrolyte membranes: (8) pristine BZCYYb1711 sintered at 1550 °C for 10 h and (9) 1% NiO–BZCYYb1711 sintered at 1450 °C for 3 h, measured across a range of temperatures. Reproduced with permission from ref 81. Copyright 2011 Elsevier.

##### 2.1.2.3 Mechanical Stability

Mechanical stability in proton-conducting electrolytes encompasses both the bulk mechanical properties of the electrolyte material and the interfacial behavior with adjacent cell components. The mechanical properties of proton-conducting perovskites are closely tied to their composition and microstructure. Generally, zirconate-rich compositions exhibit higher mechanical strength but also greater brittleness compared to cerate-rich counterparts. Yamanaka et al.^84^ systematically compared the mechanical and thermophysical properties of BaZrO_3−δ_ and BaCeO_3−δ_, showing that BaZrO_3−δ_ exhibits significantly higher mechanical strength—with Young’s modulus of 243 GPa and Vickers hardness of 4.95 GPa—compared to BaCeO_3−δ_, which has values of 154 and 2.34 GPa, respectively. This reflects the inherently stiffer and stronger bonding environment in zirconate lattices. However, these same characteristics also result in lower ductility and greater brittleness in BaZrO_3−δ_, making it more prone to mechanical failure under stress. In contrast, the lower modulus and hardness of BaCeO_3−δ_ indicate a more compliant and less brittle nature. A recent study by Zhou et al.^85^ demonstrated through Vickers indentation and micropillar splitting that fracture behavior in BaCe_0.65_Zr_0.2_Y_0.1_O_3−δ_ is strongly influenced by local microstructure, particularly the presence of pores and grain boundary chemistry. Irregular crack paths, bifurcation, and deflection observed near grain boundaries led to variations in measured fracture toughness across scales, emphasizing that grain boundary characteristics critically govern crack propagation and mechanical reliability.

Hydration-induced strain presents a unique mechanical challenge for proton-conducting electrolytes. Saz̆inas et al.^86^ demonstrated that hydration of BaZr_1–x_Y_x_O_3−δ_ and BaZr_0.7_Ce_0.2_Y_0.1_O_3−δ_ leads to measurable lattice expansion due to water incorporation, quantified as chemical strain. This expansion generates internal compressive stress, particularly at intermediate temperatures, with cerate-containing compositions like BZCY721 exhibiting the largest volumetric changes. These internal stresses can influence mechanical performance and must be considered in device design, especially for materials operating under cyclic hydration and dehydration conditions. Sereda et al.^87^ demonstrated that hydration-induced chemical expansion in proton-conducting perovskites, such as BCN, BZY20, and BCY20, can be quantitatively tracked using *in situ* XRD and dilatometry. These methods revealed that water uptake leads to measurable lattice expansion, and the authors proposed a chemical strain model that accurately predicts this expansion based on ionic radius changes and coordination number adjustments. Their results confirm that advanced characterization techniques provide critical insights into the hydration behavior of these materials, enabling the rational design of strain-tolerant electrolytes.

Interfacial delamination remains one of the most prevalent failure modes in SOCs, driven by thermal expansion mismatches and chemical interactions between the electrolyte and surrounding components. Recent research has focused on engineering interfaces with compliant layers or graded compositions to mitigate these issues. For example, the incorporation of thin film interlayers has been shown to significantly improve interfacial adhesion while suppressing cation interdiffusion.^88^ As Figure 7 illustrates, Le et al.^89^ investigated the long-term electrochemical stability of proton-conducting ceramic electrochemical cells using BZCYYb4411 as the electrolyte, with a particular focus on degradation mechanisms at the electrolyte–electrode interface. The study demonstrated that the primary degradation in both fuel cell and electrolysis modes arises not from bulk electrolyte decomposition but from interfacial instabilities—especially at the air-side electrode. These include sluggish surface exchange kinetics, gas adsorption limitations, and possible local phase evolution at the triple-phase boundary (TPB). Crucially, the introduction of a 10 mol % gadolinium-doped ceria (GDC) functional layer between the BZCYYb4411 electrolyte and the air–steam electrode was shown to dramatically suppress degradation.^89^ The GDC interlayer acted as a chemically and structurally compatible buffer, mitigating interfacial reactions and enhancing oxygen surface exchange, thereby reducing the degradation rate to less than 1% per 1000 h. In the absence of this interlayer, degradation was more pronounced, with electrochemical impedance measurements revealing significant increases in polarization resistance (R_p_) over time.^89^ These findings underscore the pivotal role of functional layers in stabilizing electrolyte–electrode interfaces in protonic ceramic cells. By serving as a barrier to unwanted chemical interactions and facilitating interfacial transport processes, such interlayers are essential for maintaining long-term durability under demanding operational conditions.

**Figure 7:**
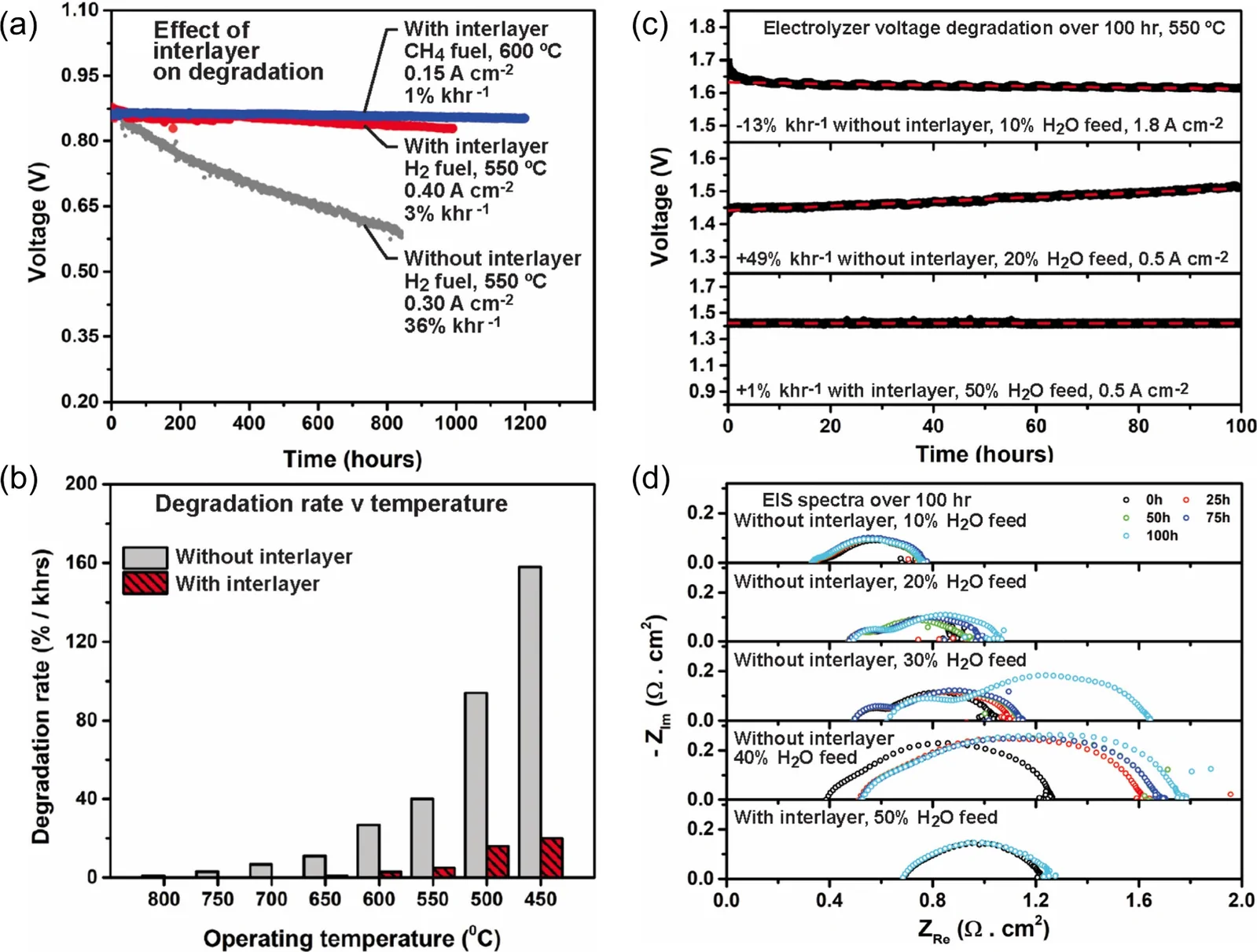
(a) Stability of PCFCs operated with H_2_ and CH_4_ fuels, highlighting the effect of a GDC interlayer and 600 °C operation in reducing the degradation rate to ∼ 1% per 1000 h. (b) Dependence of degradation rate on operating temperature, showing enhanced stability with the incorporation of the GDC interlayer. (c) Long-term stability of protonic ceramic electrolysis cells (PCECs) under various operating conditions. (d) Electrochemical impedance spectra of the PCEC measured under open-circuit conditions during the stability tests illustrate the evolution of interfacial resistance over time. Reproduced with permission from ref 89. Copyright 2022 Elsevier.

#### 2.1.3 Compatibility

The successful integration of proton-conducting electrolytes into functional SOC devices requires careful attention to their compatibility with both fuel and air electrodes. Compatibility challenges include chemical reactivity, thermal expansion mismatch, and electrochemical properties at the electrolyte-electrode interfaces. Recent efforts have focused on advancing interface engineering strategies and deepening the mechanistic understanding of interfacial reactions, thereby laying the foundation for more-durable and efficient SOC architectures.

##### 2.1.3.1 Air Electrode Compatibility

The electrolyte–air electrode interface presents several critical compatibility challenges, including chemical reactivity, thermal expansion mismatch, and stringent electrochemical requirements. Recent advances in interface characterization have enabled the development of targeted strategies to mitigate these issues and improve the long-term device performance.

Chemical reactivity between electrolyte and cathode materials is a primary concern, particularly under high-temperature fabrication conditions. Common cathode materials such as (Ba, Sr)­(Co, Fe)­O_3_ can react with barium-containing electrolytes, forming insulating secondary phases like BaCoO_2_. Yoo and Lim systematically examined the chemical compatibility between BaCe_0.6_Zr_0.2_Y_0.2_O_3−δ_ (BCZY62) proton-conducting electrolytes and Ba_0.5_Sr_0.5_Co_0.8_Fe_0.2_O_3−δ_ (BSCF) cathodes under high-temperature processing conditions. Their study revealed that interfacial reactions between BCZY62 and BSCF begin to occur at temperatures above 900 °C, with XRD analysis identifying the formation of secondary phases such as BaCoO_2.23_ and Ba-rich perovskite-type compounds. These phases result from the diffusion of Ba from the electrolyte and Co from the cathode, indicating a thermodynamic driving force for phase decomposition at the BCZY62–BSCF interface. The emergence of these reaction products poses a risk to the structural and electrochemical stability of the cell, as they are likely to be electronically conductive or insulating, depending on their stoichiometry, and can degrade the electrode–electrolyte interface. SEM-EDS mapping analyses further confirmed elemental interdiffusion at the interface after cosintering, suggesting that direct contact and high-temperature fabrication may compromise long-term performance. This study underscores the importance of cathode–electrolyte compatibility in P-RSOCs and suggests that, although BSCF is a high-performance cathode material, its use with BCZY62 electrolytes requires either lower-temperature processing or the introduction of a chemically stable barrier layer to prevent interfacial degradation.^90^

To address interfacial reactivity, two principal material strategies have been explored: the use of chemically inert cathodes and the incorporation of protective interlayers. Pr_2_NiO_4+δ_ has emerged as a promising cathode candidate due to its excellent oxygen reduction activity and minimal reactivity with proton-conducting electrolytes.^91^ Recent studies demonstrated that PrNiO_3−δ_-based cathodes exhibit exceptional stability when paired with BZCYYb electrolytes.^92^ Ultrathin interlayers deposited by PLD have shown particular promise in minimizing interfacial resistance and enhancing durability.^38^

##### 2.1.3.2 Fuel Electrode Compatibility

The fuel electrode–electrolyte interface presents a distinct set of compatibility challenges, primarily due to the reducing atmosphere during operation and the widespread use of nickel-based cermets as anode materials. Recent advances have centered on developing alternative anode materials, optimizing processing conditions, and improving our understanding of interfacial reactions under realistic operating environments.

Chemical interactions between nickel and electrolyte materials have been extensively studied, revealing complex phase equilibria that can impact long-term device stability.^93^ For example, in Y-doped BZO systems, the solubility limit of Y in the presence of NiO is approximately 12%; exceeding this threshold can lead to the formation of secondary phases such as BaY_2_NiO_5−δ_.^25^ These phases may persist upon cooling, causing local stoichiometric imbalances and potentially introducing electronic leakage pathways.^94–96^ Recent studies by Han et al.^97–99^ have mapped phase stability diagrams across varying oxygen partial pressures, providing critical insights for optimizing processing conditions.

In addition, Luo et al.^100^ reported that the chemical compatibility between proton-conducting electrolytes and NiO-based fuel electrodes is a critical factor in ensuring reliable cell fabrication and long-term operation. Figure 8 shows that cofiring processes at high temperatures (up to 1400 °C) can trigger reactions between NiO and electrolyte components, particularly when large trivalent dopants (e.g., Er^3+^, Y^3+^, Gd^3+^, Sm^3+^) are present. For example, while BaHf_0.1_Ce_0.7_Yb_0.2_O_3−δ_ (BHCYb172) shows excellent phase stability with NiO, avoiding secondary phase formation, electrolytes doped with larger rare-earth cations exhibit the formation of BaR_2_NiO_5−δ_ secondary phases, which correlate strongly with the dopant’s ionic radius. Studies show that even at lower cofiring temperatures (1100 °C), the diffusion of NiO can penetrate electrolyte layers up to ∼ 50 μm, making it difficult to suppress interfacial reactions purely through temperature control. Optimal doping strategies, such as limiting Y^3+^ content below its solubility limit (∼12%) or using smaller trivalent dopants like Yb^3+^ (optimally 20% B-site doping), help maintain both high conductivity and NiO compatibility. In contrast, excessive dopant loading or the use of dopants with low NiO solubility can lead to chemical reactivity, phase instability, and compromised performance.^100^

**Figure 8:**
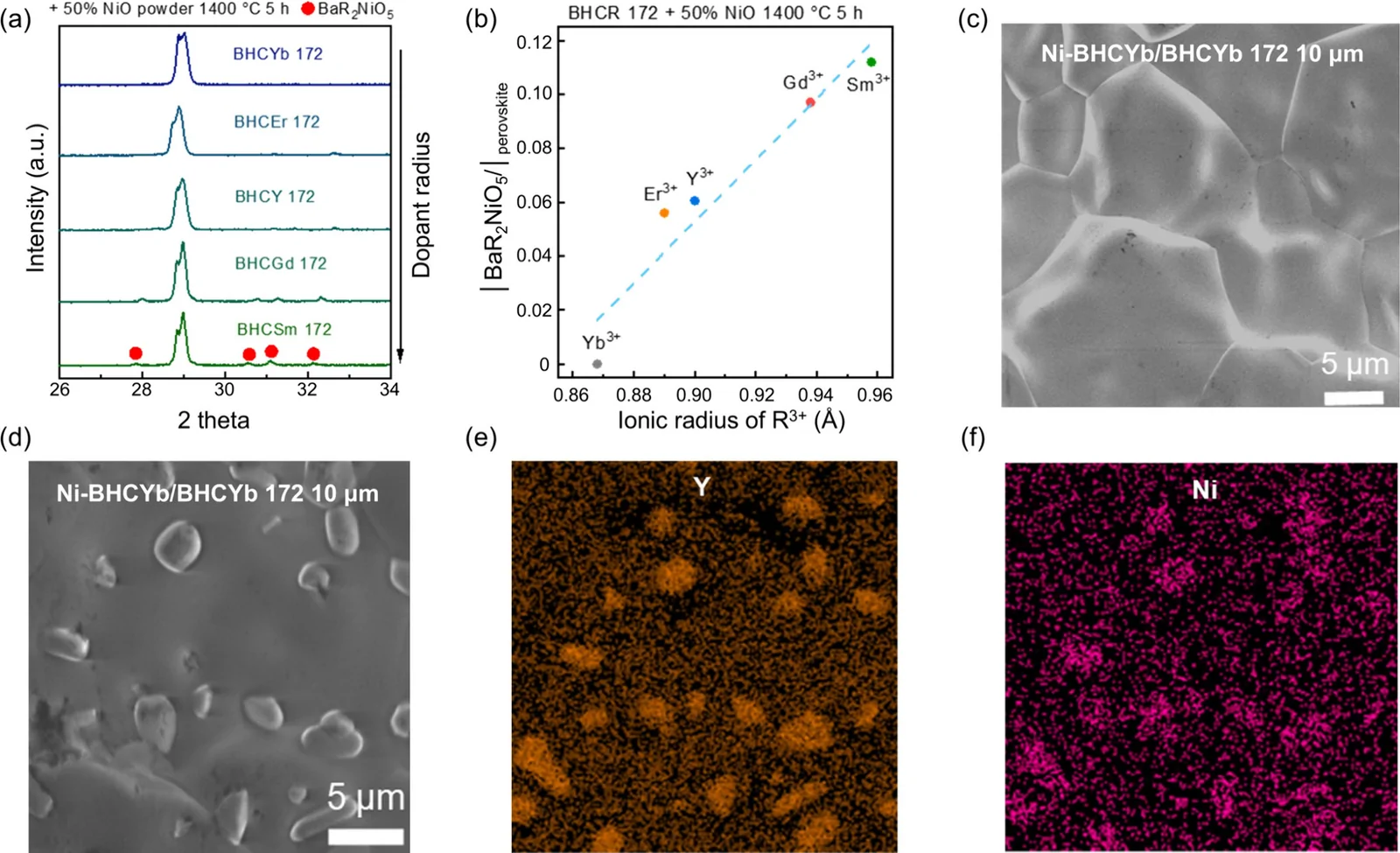
(a) Magnified XRD patterns of BaHf_0.1_Ce_0.7_R_0.2_O_3−δ_ (R = Yb, Er, Y, Gd, Sm) after cofiring with NiO at 1400 °C for 5 h. (b) The intensity ratio of the BaR_2_NiO_5_ phase relative to the perovskite (220) peak in BaHf_0.1_Ce_0.7_R_0.2_O_3−δ_ (R = Er, Y, Gd, Sm) as a function of the R^3+^ ionic radius. (c) SEM image of the electrolyte surface of a Ni–BHCYb172|BHCYb172 half-cell with a 10 μm electrolyte thickness after firing at 1400 °C for 5 h. (d–f) SEM image and corresponding EDS elemental mapping of the electrolyte surface of a Ni–BHCYb172|BHCY172 half-cell with a 10 μm-thick electrolyte after firing under the same conditions. Reproduced with permission from ref 100. Copyright 2022 RSC.

Furthermore, due to a general lack of cell-level validation data, it remains unclear whether many novel proton conductors are compatible with commonly used electrode materials such as NiO. This compatibility issue poses a significant hurdle for practical application, as interfacial reactions during cosintering or operation can degrade performance. For example, Ba_7_Nb_4_MoO_20−δ_ (BNM), which will be discussed in Section 2.4, has demonstrated reasonable bulk conductivity (σ_bulk_) and chemical stability; however, it reacts with NiO at typical cell processing temperatures, making it difficult to realize its full performance potential at the system level. Such challenges underscore the importance of holistic evaluation—including chemical compatibility with electrodes—when assessing new electrolyte candidates for RSOCs.

To mitigate these challenges, two primary strategies have emerged: optimization of sintering conditions and development of alternative anode materials. Controlled atmosphere sintering under reducing conditions has shown promise for suppressing unwanted secondary phase formation. Alternatively, proton-conducting ceramic anodes, such as doped strontium titanates, offer a potential path forward, although they generally exhibit lower catalytic activity compared to nickel cermets.^101^

Microstructural engineering at the anode–electrolyte interface has also emerged as a promising research direction.^102^ Nanostructured interfaces fabricated using infiltration techniques have shown improved electrode compatibility and electrochemical performance due to reduced processing temperature. Notably, the development of 3D-printed anode scaffolds has enabled precise control over the interface morphology, resulting in improved current collection efficiency and reduced polarization losses.^103^

##### 2.1.3.3 Thermal Expansion Coefficient Mismatch

Mismatch in thermal expansion coefficients (TECs) between solid oxide cell components remains a persistent challenge in SOC design and operation, often leading to interfacial stresses, delamination, or cracking during thermal cycling. Recent efforts have focused on developing materials with tailored thermal expansion behavior and implementing advanced interface design to address this issue.

The thermal expansion behavior of proton-conducting perovskites is influenced by several factors, including composition, defect chemistry, and hydration state. In general, zirconate-rich compositions exhibit lower TEC values (∼7–8 × 10^–6^ K^–1^) compared to cerate-rich materials (∼9–10 × 10^–6^ K^–1^).^104^ Table 2 presents a summary of TEC values for common electrolyte and air electrode materials.

**Table 2:** Thermal Expansion Coefficients of Representative Electrolytes and Air Electrodes

Material	TEC (×10^–6^ K^–1^)	Measurement Temperature Range (°C)	Atmosphere
SrCeO_3−δ_ ^108^	11.1	25–800	
SrZrO_3−δ_ ^109^	9.7	25–627	-
BaCeO_3−δ_ ^84^	11.2	25–800	Reducing
BaZrO_3−δ_ ^84^	7.1	25–800	Reducing
BaZr_0.8_Y_0.2_O_3−δ_ ^110^	8.2	100–900	Air
BaCe_0.8_Y_0.2_O_3−δ_ ^110,111^	11.6	100–620	Air
BaZr_0.1_Ce_0.7_Y_0.2_O_3−δ_ ^110^	11.3	100–630	Air
BaZr_0.1_Ce_0.7_Y_0.1_Yb_0.1_O_3−δ_ ^104^	9.1	25–1200	Air
BaZr_0.4_Ce_0.4_Y_0.1_Yb_0.1_O_3−δ_ ^112^	10.1	700–900	N_2_
BaSnO_3−δ_ ^113^	9.3	27–1500	-
BaHfO_3−δ_ ^114^	6.9	27–1227	-
BaIn_0.8_Ti_0.2_O_3−δ_ ^115^	12.5	400–850	Air (2.3% H_2_O)
LaNbO_4−δ_ ^116,117^	8.6	500–1000	Air (2.3% H_2_O)
La_6_WO_12−δ_ ^118^	11	27–800	-
La_2_Ce_2_O_7−δ_ ^119,120^	14.5	500–1000	-
La_2_Zr_2_O_7−δ_ ^121^	9.7	25–1200	Air
BaCo_0.4_Fe_0.4_Zr_0.1_Y_0.1_O_3−δ_ ^122^	21.6	300–700	Air
Ba_0.5_Sr_0.5_Co_0.8_Fe_0.2_O_3−δ_ ^123,124^	19.0–20.8	600–900	O_2_–N_2_
La_0.6_Sr_0.4_Co_0.2_Fe_0.8_O_3−δ_ ^125,126^	16.5	25–900	-
La_0.8_Sr_0.2_MnO_3−δ_ ^127^	12.4	-	-
Pr_2_NiO_4+δ_ ^128^	13.4	400–1200	Air
PrBaCo_2_O_5+δ_ ^129^	21.5	50–800	Air
PrBa_0.5_Sr_0.5_Co_1.5_Fe_0.5_O_5+δ_ ^130^	21.3	250–900	-

To mitigate TEC mismatch, three primary strategies have emerged: development of tailored composite electrodes, implementation of graded interfaces, and optimization of operational protocols. Composite electrodes that incorporate electrolyte material have proven especially effective, improving TEC matching while maintaining adequate electronic conductivity. For example, Ni–BZY20 cermet containing 40–60 vol % electrolyte phase has demonstrated excellent thermo-mechanical compatibility with BZY20 electrolytes.^105^ Graded interfaces, where the composition transitions gradually from pure electrode to pure electrolyte over several microns, represent another promising approach. Advanced fabrication methods such as PLD and aerosol deposition have enabled precise control over these graded architectures, greatly enhancing interfacial durability. Notably, Umer et al.^106^ developed a high-performance PCFC enabled by a fibrous anode scaffold, pore-filling via electrophoretic deposition, and PLD of functional layers. The vertically aligned pores improve gas transport, while electrophoretic deposition fills surface defects, allowing the deposition of dense, crack-free electrolytes and interlayers. A graded anode interlayer mitigates thermal stress and prevents delamination. This work offers a scalable route to durable, high-efficiency PCFCs. In parallel, operational strategies such as controlled heating and cooling rates, as well as optimized thermal cycling protocols, have contributed to improving long-term reliability. Recent modeling studies have provided valuable insights into stress evolution during thermal cycling, informing the development of more robust cell designs. Finite element modeling approaches developed by Yang et al.^107^ can predict delamination risks under a range of operating conditions, enabling proactive design modifications.

### 2.2 Single Perovskite Oxides

Proton-conducting electrolytes can be broadly classified on the basis of their structural frameworks and chemical compositions. Among these, perovskite-type oxides have attracted the most attention because of their favorable proton transport properties, tunable structures, and relatively high chemical and thermal stability. This section provides a systematic overview of the major classes of proton-conducting materials, with particular emphasis on perovskite-based electrolytes, which remain the most extensively studied and technologically significant groups.

Proton-conducting oxides have become essential materials for intermediate-temperature solid oxide electrochemical devices. Over the past four decades, the field has advanced through key discoveries that addressed fundamental challenges in conductivity, chemical stability, and processability.^7,50,56^ As shown in Figure 9, pioneering work by Iwahara^131^ in 1981 first identified proton conduction in SrCeO_3−δ_, establishing the potential of perovskite oxides as proton conductors. This was followed in 1988 by the demonstration of superior conductivity in BaCeO_3−δ_, though its chemical instability under CO_2_-containing atmospheres remained a major limitation.^132^

**Figure 9:**
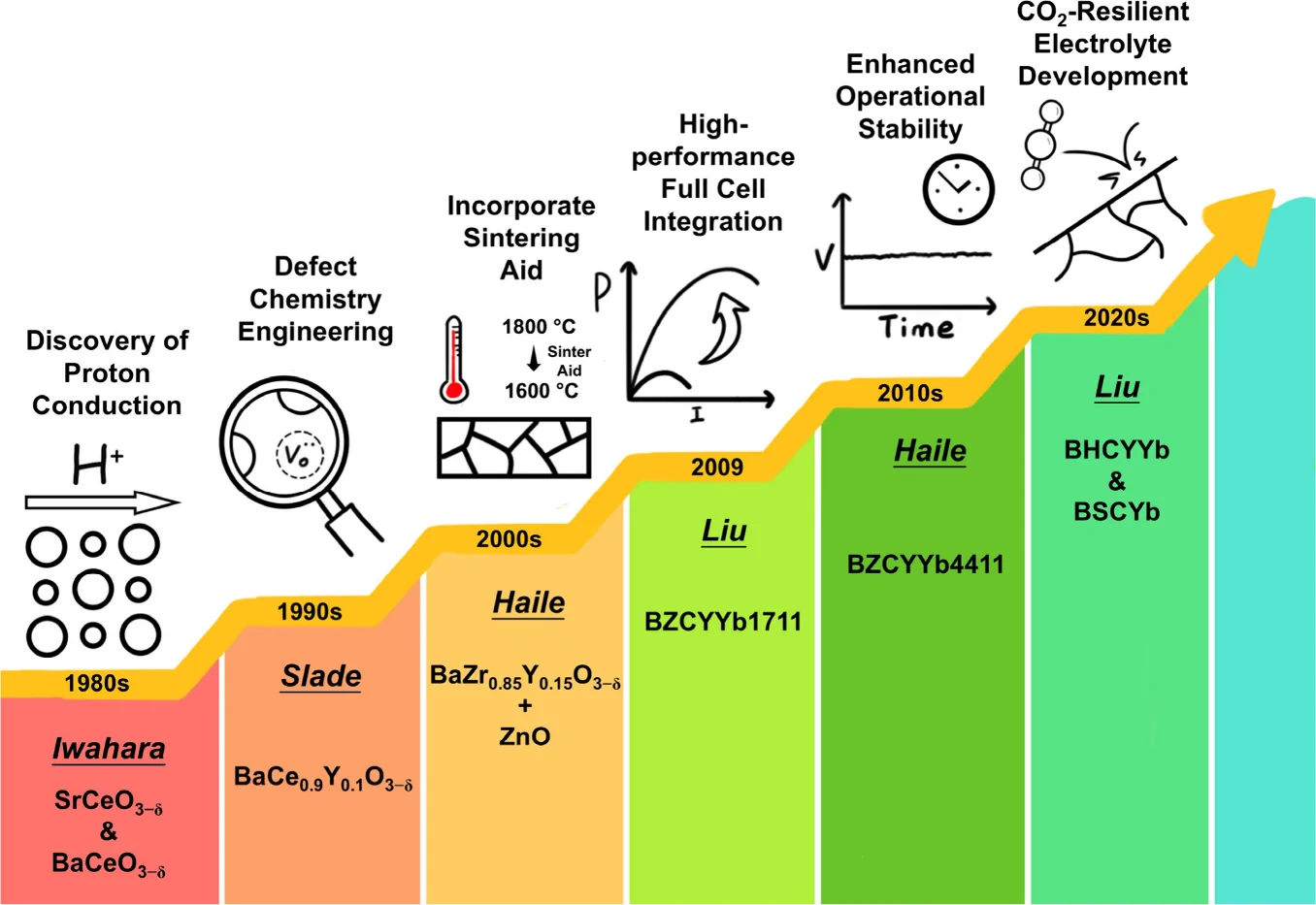
Evolution of perovskite-type proton-conducting electrolyte.

Significant progress was made in the 1990s and 2000s with the development of doped compositions. For example, Slade’s study in 1993 on BCY20 optimized proton conductivity through controlled oxygen vacancy creation.^133^ A breakthrough came in 2005 when Haile^26^ introduced ZnO sintering aids for BZY, addressing the longstanding challenge of poor sinterability in zirconate systems. The following decade saw the emergence of optimized solid solutions: Liu’s 2006 BaZr_0.1_Ce_0.7_Y_0.2_O_3−δ_ (BZCY172) composition successfully balanced the high conductivity of cerates with the superior chemical stability of zirconates,^134^ while the 2009 BZCYYb1711 formulation further improved performance through Yb codoping.^37^ More recently, the BZCYYb4411 composition reported by Haile^38^ in 2018 demonstrated exceptional CO_2_ stability, the BHCYYb system with Hf incorporation reported by Liu in 2020 achieved improved chemical robustness,^21^ and the Sn-containing BaSn_x_Ce_0.8–x_Yb_0.2_O_3−δ_ (BSCYb) formulation reported by Liu in 2024 showed enhanced durability under both CO_2_ and steam environments.^135^

This historical progression highlights an ongoing effort to optimize three interrelated electrolyte properties: proton conductivity, chemical stability, and sinterability—each addressed through systematic materials design. Among the various structural families, ABO_3_-type perovskites based on BaCeO_3−δ_ and BaZrO_3−δ_ have emerged as the most widely studied and technologically relevant proton conductors, owing to their favorable combination of conductivity, stability, and electrode compatibility.^15,136^

The perovskite framework offers an excellent matrix for proton transport, with its flexible crystal structure enabling precise tuning of proton concentration and mobility through controlled doping strategies. While this review primarily focuses on perovskite systems due to their dominant role in the field, alternative structural families showing promising proton-conducting behavior will also be discussed in later sections.^30^ Perovskite oxides, with the general formula ABO_3_, form the cornerstone of proton-conducting electrolyte research due to their combination of high proton mobility, structural flexibility, and doping versatility. Proton conduction in these materials generally relies on the introduction of oxygen vacancies via acceptor doping, which facilitates water incorporation and subsequent proton transport. Perovskite-type electrolytes can be categorized into three main groups: single perovskite oxides, double perovskites, and more complex perovskite-derived structures. Among these, single perovskites have been the most thoroughly investigated due to their simpler crystal chemistry and more predictable defect behavior. Single perovskite oxides adopt a relatively simple crystal structure, making them an ideal platform for systematically tuning conductivity, stability, and mechanical properties through targeted doping and cation substitution. Within this family, two major compositional subclasses—A^2+^B^4+^O_3_ and A^3+^B^3+^O_3_ perovskites—exhibit distinct proton conduction behaviors and stability profiles, largely determined by their differing cationic arrangements and defect chemistries.

#### 2.2.1 A^2+^B^4+^O_3_-Type Perovskite

A^2+^B^4+^O_3_-type perovskites, characterized by alkaline-earth cations (such as Sr^2+^, Ba^2+^) on the A-site and tetravalent cations (such as Ce^4+^, Zr^4+^) on the B-site, represent one of the most extensively studied classes of proton-conducting materials. Systems such as SrCeO_3−δ_, BaCeO_3−δ_, and Ba­(Zr, Ce)­O_3−δ_ have drawn particular attention due to their high intrinsic proton conductivity and well-documented proton incorporation mechanisms. Their defect chemistry, phase stability, and transport properties continue to be actively investigated, making them central to ongoing efforts to improve SOC electrolyte performance.

##### 2.2.1.1 SrCeO_3−δ_-Based Oxide

SrCeO_3−δ_ is one of the earliest reported perovskite oxides exhibiting protonic conductivity at elevated temperatures and continues to serve as an important model system for understanding defect-mediated transport in proton-conducting ceramics. Structurally, SrCeO_3−δ_ crystallizes in an orthorhombic perovskite structure at room temperature, with a Goldschmidt tolerance factor of ∼0.88,^137,138^ indicating a significant deviation from cubic symmetry. This deviation results in octahedral tilting and distortion of the CeO_6_ network, which can profoundly influence both the energetics and connectivity of proton hopping pathways.^138,139^

The introduction of oxygen vacancies—essential for hydration and proton incorporation—is typically achieved through acceptor doping with trivalent rare-earth cations such as Gd^3+^, Y^3+^, or Sm^3+^, which substitute for Ce^4+^ at the B-site. Among these, Gd-doped SrCeO_3−δ_ (e.g., SrCe_0.95_Gd_0.05_O_3−δ_) has been studied extensively due to its relatively high protonic conductivity, reasonable chemical compatibility, and moderate sinterability. Zaja̧c et al.^140^ have shown that the proton conductivity of such Gd-doped systems can approach 10^–3^ S cm^–1^ at 700 °C under humidified atmospheres, placing it in a similar performance range as undoped BaCeO_3−δ_ or lightly doped BaZrO_3−δ_ systems. The mechanism of proton transport in SrCeO_3−δ_ follows the conventional hydration-migration pathway, where water molecules dissociatively adsorb at oxygen vacancy sites to form mobile protonic defects (OH_O_^•^). These defects migrate via thermally activated Grotthuss-type hopping between adjacent lattice oxygen atoms. However, due to the relatively low tolerance factor and resulting anisotropic lattice environment, the energy landscape for proton migration is rugged and spatially heterogeneous. This leads to strong defect–dopant associations, particularly at higher dopant concentrations, which can trap protons and reduce the effective diffusion coefficient.^140^

In addition to structural constraints, chemical instability remains a significant limitation of SrCeO_3−δ_-based materials. Under CO_2_-rich environments, SrCeO_3−δ_ readily reacts with carbon dioxide to form SrCO_3_ and CeO_2_. Thermodynamic studies by Shirsat et al.^141^ indicate that this reaction is favorable even at modest CO_2_ partial pressures, and decomposition onset temperatures are often below 800 °C. The formation of SrCO_3_ not only compromises ionic conductivity but also leads to severe microstructural degradation and loss of mechanical integrity. Exposure to humid environments induces an additional degradation mechanism via hydroxylation. This reaction is particularly problematic at intermediate temperatures, where steam partial pressures are high, and the kinetics of Sr­(OH)_2_ formation are favorable.^141^ Experimental investigations using TGA and *in situ* XRD have revealed that degradation often initiates at grain boundaries, eventually leading to bulk decomposition and mechanical failure. To address these challenges, extensive efforts have been devoted to modifying the B-site chemistry of SrCeO_3−δ_ by partially substituting Ce^4+^ with Zr^4+^. The rationale is that Zr^4+^ forms stronger Zr–O bonds and introduces greater lattice rigidity, thereby enhancing chemical stability without compromising perovskite phase formation. For example, compositions such as SrCe_0.2_Zr_0.6_Y_0.2_O_3−δ_ and SrCe_0.4_Zr_0.4_Y_0.2_O_3−δ_ have been shown to resist decomposition in both CO_2_ and steam atmospheres, achieving stable operation over hundreds of hours.^142^

Another advantage of SrCeO_3−δ_-based materials is their relatively favorable sintering behavior compared to zirconate analogs such as BaZrO_3−δ_ or SrZrO_3−δ_. While BaZrO_3−δ_ typically requires sintering temperatures exceeding 1600 °C, dense SrCeO_3−δ_ ceramics can be achieved at 1400–1450 °C. However, achieving phase purity remains challenging due to the sensitivity of SrCeO_3−δ_ to the processing atmosphere and dopant volatility. Careful control of stoichiometry, particle size, and calcination atmosphere is necessary to prevent secondary phase formation and ensure reproducible proton conductivity.^142,143^

Overall, SrCeO_3−δ_ represents a chemically and structurally distinct class of proton-conducting oxides with a complex balance of performance attributes. Its intrinsic limitations—moderate conductivity, poor environmental stability, and strong proton–defect association—have prevented it from achieving widespread adoption in standalone form. However, when combined with more stable or conductive systems (e.g., as part of Sr­(Ba, Ce, Zr)­O_3−δ_ solid solutions), SrCeO_3−δ_ contributes to tuning phase behavior, sintering kinetics, and thermal expansion properties. These attributes position it as a valuable component in the broader design space of mixed perovskite proton conductors for next-generation fuel cells and electrochemical devices.

##### 2.2.1.2 BaCeO_3−δ_-Based Oxide

BaCeO_3−δ_-based electrolytes represent one of the most extensively studied classes of single perovskite proton conductors, largely due to their outstanding proton conductivity, favorable defect chemistry, and relatively accessible fabrication processes. Proton conduction in BaCeO_3−δ_ arises from the formation of oxygen vacancies via acceptor doping at the B-site, typically achieved by substituting Ce^4+^ ions with trivalent rare-earth cations. These oxygen vacancies facilitate the incorporation of water molecules through hydration reactions, resulting in the formation of mobile protonic defects that migrate through the oxide lattice.^144^ Doped BaCeO_3−δ_ compositions, including BaCe_0.9_Y_0.1_O_3−δ_ and BCY20, have demonstrated high proton conductivities on the order of ∼ 10^–3^–10^–2^ S cm^–1^ in the intermediate temperature range (∼400–500 °C), making them promising candidates for IT-SOFCs and electrolysis applications.^145,146^

The excellent transport properties of BaCeO_3−δ_ are closely linked to its relatively large unit cell volume and the high polarizability of its oxygen sublattice, which together lower the activation energy for proton hopping. Furthermore, BaCeO_3−δ_ exhibits strong hydration thermodynamics, favoring proton incorporation even under moderate steam partial pressures, which contributes to its high conductivity in the 400–700 °C temperature window.

However, BaCeO_3−δ_-based electrolytes are significantly limited by their poor chemical stability in CO_2_- and H_2_O-containing environments, which are unavoidable in practical operating conditions.^147,148^ In CO_2_ atmospheres, BaCeO_3−δ_ readily decomposes via eq 7. While in the presence of steam, it reacts as shown in eq 8.

Both reactions produce insulating secondary phases such as BaCO_3_ and Ba­(OH)_2_, which compromise ionic pathways, weaken mechanical integrity, and degrade electrochemical performance over time. The decomposition tendency is especially severe at lower operating temperatures (<700 °C), where the thermodynamic driving forces for carbonate and hydroxide formation are strongest. Zakowsky et al.^149^ demonstrated that BaCe_0.9_Y_0.1_O_3−δ_ begins to carbonate at temperatures as low as 500 °C under pure CO_2_, with complete decomposition occurring below 700 °C. Their thermogravimetric and XRD analyses confirmed that at these lower temperatures, the thermodynamic driving force for BaCO_3_ and CeO_2_ formation is strong, making BaCe_0.9_Y_0.1_O_3−δ_ particularly vulnerable to carbonation during operation in CO_2_-rich environments.

To improve the chemical resilience of BaCeO_3−δ_, several strategies have been pursued. Early work focused on dopant optimization. However, excessive doping can lead to defect clustering and reduced proton mobility, underscoring the need for careful dopant engineering.

Microstructural optimization has also been critical. Dense, low-porosity microstructures can slow the ingress of CO_2_ and H_2_O, delaying degradation. Advanced sintering techniques—such as the use of transient liquid-phase sintering aids and field-assisted methods like spark plasma sintering (SPS)—have been effective in achieving dense BaCeO_3−δ_ ceramics at reduced processing temperatures, minimizing barium volatility and compositional deviations. Yang et al.^150^ demonstrated that incorporating Cu^2+^ into the lattice of BaCeO_3−δ_-based electrolyte significantly enhances sinterability, enabling high-density ceramics at just 1200 °C—much lower than the typical >1600 °C required for BaCeO_3−δ_-based electrolytes. The Cu^2+^ ions, occupying interstitial sites, promote grain growth and reduce grain boundary resistance (R_gb_) without compromising phase stability. Also, Kim et al.^151^ demonstrated that grain boundaries in BaCeO_3−δ_ polycrystals often contain nanoscale Ba-rich amorphous intergranular phases that severely impede proton conduction and promote chemical degradation. Using STEM and EDS analysis, they identified these secondary phases as structurally disordered and reactive to H_2_O and CO_2_. By eliminating them through Ba-deficient composition control or postannealing, they achieved significantly reduced grain boundary impedance and enhanced chemical stability. These findings confirm that secondary phases at grain boundaries act as both proton transport barriers and degradation pathways, underscoring the importance of microstructural control in optimizing proton-conducting perovskites.

Surface modification strategies have been explored as well, including the application of thin, chemically inert coatings, such as doped ceria, to act as diffusion barriers against CO_2_ and H_2_O. While these approaches show promise, challenges remain in maintaining long-term adhesion and compatibility under thermal cycles.

Despite these efforts, the intrinsic chemical reactivity of BaCeO_3−δ_ with CO_2_ and H_2_O remains a fundamental limitation. Partial substitution of Ce^4+^ with more chemically stable tetravalent cations (e.g., Zr^4+^), which will be discussed in the following section, has been widely recognized as an effective strategy for enhancing stability. Nonetheless, BaCeO_3−δ_-based systems continue to attract attention, particularly for applications in controlled, low-CO_2_, and dry environments. Moreover, they provide valuable model systems for studying fundamental proton transport mechanisms, defect interactions, and electrode–electrolyte interfaces.^152,153^

Recent studies have also renewed interest in BaCeO_3−δ_ thin films.^154^ Advances in PLD and atomic layer deposition (ALD) have enabled the fabrication of highly textured BaCeO_3−δ_ films with improved chemical stability and mechanical properties compared to bulk ceramics. These thin-film approaches open exciting opportunities to exploit BaCeO_3−δ_’s excellent proton conductivity while mitigating degradation risks through microstructural engineering and protective surface treatments.

In summary, BaCeO_3−δ_-based electrolytes exhibit some of the highest proton conductivities reported among oxide-based materials, making them benchmark systems for proton-conducting ceramics. While their chemical instability in CO_2_- and H_2_O-rich environments constrains widespread application, continued advances in dopant design, microstructural control, and surface protection strategies hold promise for enabling their use in targeted next-generation protonic devices operating under carefully controlled conditions.

##### 2.2.1.3 Ba­(Ce, Zr)­O_3−δ_-Based Oxide

Ba­(Ce, Zr)­O_3−δ_-based electrolytes currently represent one of the most promising families of proton-conducting perovskite oxides, as they combine high proton conductivity with dramatically improved chemical and mechanical stability compared to pure BaCeO_3−δ_ systems. The key innovation lies in the partial substitution of Ce^4+^ with Zr^4+^ in the perovskite lattice, fundamentally altering the lattice stability and thermodynamic properties, thereby suppressing degradation reactions under operational conditions. Fabbri et al.^155^ demonstrated that partial substitution of Ce^4+^ with Zr^4+^ in BaCe_0.8–x_Zr_x_Y_0.2_O_3−δ_ significantly improves the chemical stability of the material under CO_2_ exposure by altering both the lattice structure and thermodynamic behavior. XRD analysis after exposure to pure CO_2_ at 900 °C showed that compositions with higher Zr content (x ≥ 0.5) maintained the perovskite phase, while Ce-rich compositions decomposed into BaCO_3_ and CeO_2_. This substitution reduced the unit cell volume and increased the material’s tolerance factor, thereby stabilizing the lattice and suppressing degradation reactions under fuel cell operating conditions.^155^

The introduction of Zr^4+^—a smaller, more chemically inert cation than Ce^4+^—strengthens the metal–oxygen bonding framework within the perovskite structure. This reinforcement significantly enhances the resistance of Ba­(Ce, Zr)­O_3−δ_-based materials to reactions with CO_2_ and H_2_O, the primary degradation agents for cerate-based electrolytes. Thermodynamic studies have shown that increasing Zr content elevates decomposition temperatures and reduces the extent of carbonate and hydroxide formation, even under high CO_2_ and steam partial pressures. As a result, Ba­(Ce, Zr)­O_3−δ_ compositions have emerged as leading candidates for IT-SOFCs and electrolysis cells operating under realistic conditions.^156^

To combat these issues, material development has shifted toward BaZrO_3−δ_-based and Zr-rich Ba­(Ce, Zr)­O_3−δ_ solid solutions. Zirconate-based electrolytes, although inherently less conductive than their cerate counterparts, exhibit superior chemical stability in CO_2_- and H_2_O-rich environments due to the stronger Zr–O bonds and higher decomposition energies. As illustrated in Figure 10a–d, compositions such as BZCYYb4411 represent a practical balance between conductivity and durability, achieving improved phase stability while maintaining acceptable ionic transport properties.^38^

**Figure 10:**
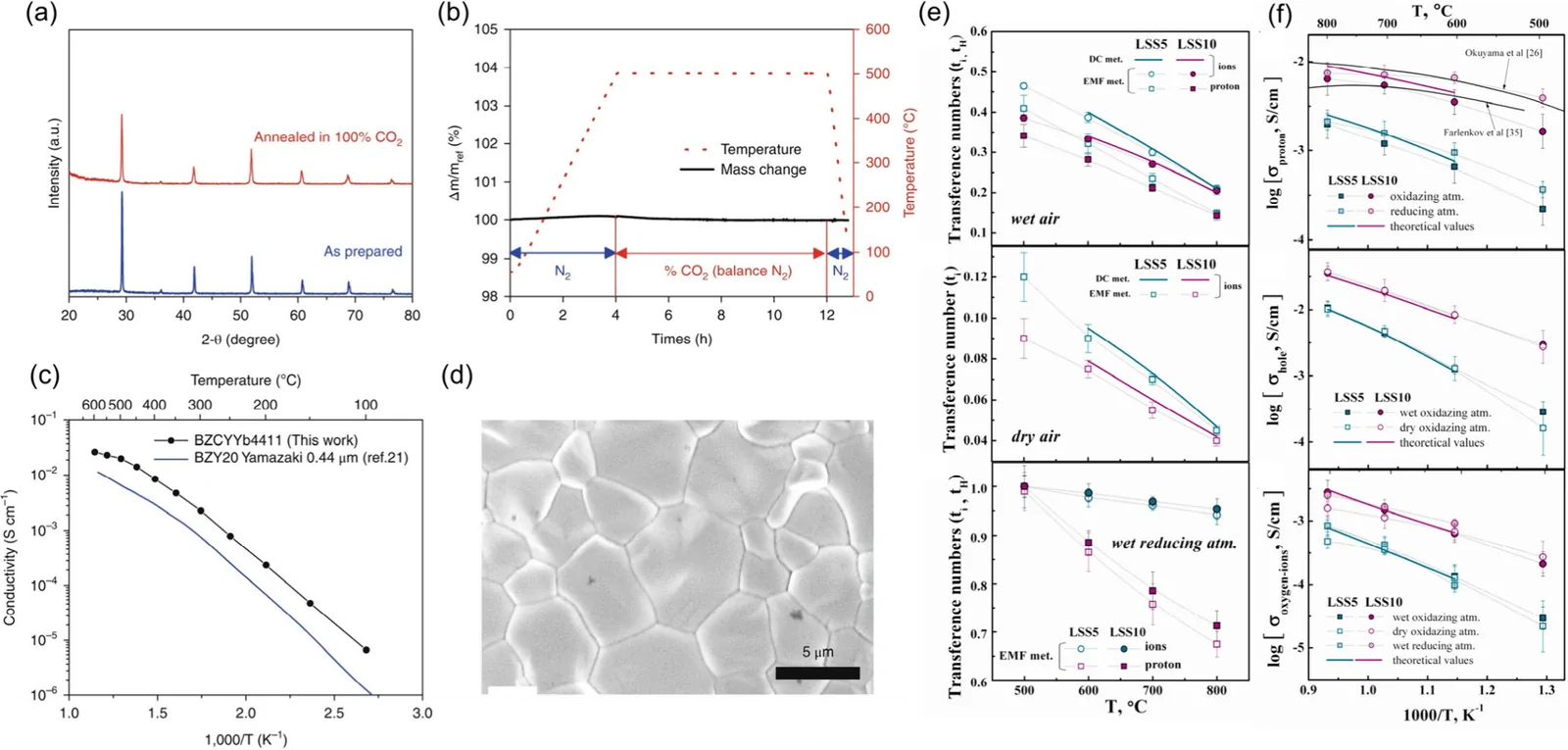
(a) XRD patterns of the sample before and after exposure to 100% CO_2_ at 500 °C, illustrating phase stability. (b) TGA profile under 60% CO_2_ (balance N_2_) at 500 °C, indicating mass changes associated with carbonation. (c) Proton conductivity measured under humidified N_2_ (pH_2_O = 0.031 atm) compared to that of BZY20 sintered under similar conditions. (d) SEM image of the as-sintered surface, showing microstructural features. Reproduced with permission from ref 38. Copyright 2018 Springer Nature. (e) Temperature dependence of the proton transference number (t_H^+^_) and the total t_i_ for La_0.95_Sr_0.05_ScO_3−δ_ and La_0.9_Sr_0.1_ScO_3−δ_ under three conditions: humid air (pH_2_O = 2.8 kPa), dry air (pH_2_O ≤ 0.1 kPa), and humid reducing atmosphere (pH_2_O = 2.8 kPa). (f) Temperature dependence of the proton, hole, and oxygen-ion conductivities of La_0.95_Sr_0.05_ScO_3−δ_ and La_0.9_Sr_0.1_ScO_3−δ_ measured in an oxidizing atmosphere at pH_2_O = 2.8 kPa (closed symbols) and pH_2_O ≤ 0.1 kPa (open symbols), as well as in a humid reducing atmosphere at pH_2_O = 2.8 kPa (shaded symbols). Reproduced with permission from ref 39. Copyright 2021 Elsevier.

From a proton transport perspective, Ba­(Ce, Zr)­O_3−δ_ solid solutions retain many of the favorable characteristics of pure BaCeO_3−δ_, such as an open lattice structure and strong hydration thermodynamics, while introducing new complexities. The hydration reaction remains highly exothermic over a broad temperature range, allowing substantial proton incorporation at ∼ 400–600 °C.^157^ However, Zr substitution also alters lattice parameters, cation–oxygen bond strength, and defect distributions, which can influence proton mobility. Notably, excessive Zr incorporation tends to shrink the unit cell, increase lattice distortion, and elevate the activation energy for proton hopping—resulting in a trade-off between chemical stability and ionic conductivity.^158^ To optimize performance, achieving a balance between maintaining sufficient proton transport pathways and enhancing structural robustness is essential.

One of the landmark studies in this area by Yang et al.^37^ highlighted the importance of codoping strategies using multiple acceptor cations in Ba­(Ce, Zr)­O_3−δ_-based perovskites. In particular, they focused on BZCYYb1711, where the combination of Y^3+^ and Yb^3+^ as codopants led to a material that simultaneously exhibited high proton and oxide-ion conductivities, excellent catalytic activity at the anode, and superior tolerance to coking and sulfur poisoning during hydrocarbon reforming. Mechanistically, this codoping approach balances lattice distortion and free volume effects: Y^3+^, with a larger ionic radius, introduces favorable lattice expansion and high oxygen vacancy formation, whereas Yb^3+^, being smaller, refines the local lattice strain and suppresses excessive lattice softening. This balance optimizes both the hydration thermodynamics (as quantified by negative hydration enthalpies) and the long-range protonic diffusion coefficient, positioning BZCYYb1711 as one of the most successful electrolyte materials for PCFCs.^37^ RSOCs based on proton-conducting electrolytes exhibit exceptional performance across multiple configurations. The NiO–BZCYYb1711|BZCYYb1711|BaCoO_3−δ_–La_0.6_Sr_0.4_Co_0.2_Fe_0.8_O_3−δ_ (LSCF) cell, with a dense ∼ 10 μm BZCYYb1711 electrolyte, delivers outstanding reversible performance, achieving peak fuel cell power densities of 2.04, 1.60, 1.16, 0.78, and 0.41 W cm^–2^ at 700, 650, 600, 550, and 500 °C, respectively. In electrolysis mode at 1.3 V, it achieves current densities of 2.3, 1.8, 1.0, and 0.5 A cm^–2^ at 650–500 °C.^159^ High-performance PCECs fabricated using tape casting and ultrasonic spray-coating to form an ultrathin bamboo-structured electrolyte with *in situ* BSC + PBSCF electrodes achieve open-circuit voltages above 1.05 V under fuel cell operation, and a peak power density (PPD) of 1.64 W cm^–2^ at 600 °C.^160^ The Ni–BZCYYb1711|BZCYYb1711|Ba_0.9_Pr_0.1_Hf_0.1_Y_0.1_Co_0.8_O_3−δ_ cell achieves peak fuel cell power densities of 1.37, 0.96, and 0.62 W cm^–2^ at 600, 550, and 500 °C, respectively, and delivers 2.40 A cm^–2^ at 600 °C in electrolysis mode, demonstrating excellent dual-mode performance and stability.^161^ Finally, the 5 × 5 cm^2^ NiO–BaCe_0.55_Zr_0.3_Y_0.15_O_3‑δ_|BaCe_0.55_Zr_0.3_Y_0.15_O_3‑δ_|BSCF cell, fed with humidified H_2_ (anode) and air (cathode), achieves open-circuit voltages of 1.056 V at 600 °C and 1.12 V at 500 °C, confirming gastight, dense electrolyte performance. It delivers peak power densities of 1.302 W cm^–2^ at 600 °C and 0.535 W cm^–2^ at 500 °C, with low area-specific ohmic resistance (R_o_), highlighting the low overall resistance and potential for further performance gains through optimized electrode microstructures.^162^ These results demonstrate the remarkable performance and versatility of P-RSOCs based on Ba­(Ce, Zr)­O_3−δ_-based proton-conducting electrolytes across a range of air-electrode materials and operating conditions.

Further improvements have been achieved through advanced doping strategies. Multidopant systems incorporating Y^3+^, Yb^3+^, Gd^3+^, and Sc^3+^ have been used to fine-tune defect chemistry and lattice strain. Kim et al.^163^ demonstrated that the smaller ionic radii of Yb^3+^ and Sc^3+^ dopants effectively reduce local lattice distortions in BaZrO_3−δ_ and BaCeO_3−δ_, promoting more uniform proton migration pathways and mitigating proton trapping. Their DFT analysis revealed that these dopants induce favorable electron density redistribution and bond angle modifications upon oxygen vacancy formation, which weakens proton-acceptor binding. This effect enhances long-range proton conductivity by suppressing localized trapping.

Microstructural engineering has also been critical in realizing the full potential of Ba­(Ce, Zr)­O_3−δ_-based electrolytes. Achieving high-density ceramics through optimized sintering protocols and the use of sintering aids such as NiO, ZnO, or CuO is vital to minimize R_gb_ and maximize σ_bulk_.^26^ Grain boundary characteristics play a decisive role in performance. Grain boundaries can act as barriers to proton transport if decorated with secondary phases or impurities. Haile et al.^72^ demonstrated that in proton-conducting perovskites like BaCe_0.85_Gd_0.15_O_3−δ_, grain boundary resistivity is significantly higher than bulk resistivity, often by several orders of magnitude. They attributed this to the presence of space-charge layers and possible segregation of secondary phases, such as amorphous BaO or Ba­(OH)_2_, which can locally block proton conduction. Similarly, Cervera et al.^164^ showed that in nanostructured Y-doped BaZrO_3−δ_, structurally disordered grain boundaries and trace hydroxide phases resulted in poor interfacial contact and low proton conductivity in as-pressed or low-temperature-annealed samples. Only after high-temperature annealing, which improved grain boundary quality and dopant distribution, did the proton conductivity increase significantly.

Similarly, strategies such as controlling dopant segregation and applying thin protective interlayers have proven effective in mitigating grain boundary effects. Lindman et al.^165^ showed through first-principles free energy calculations that dopant segregation to grain boundaries in BaZrO_3−δ_ is driven by both energetic and entropic factors, with ionic radius playing a key role. Their results revealed that larger dopants such as Y^3+^ and Gd^3+^ tend to segregate to grain boundaries more readily than smaller ones like Sc^3+^, contributing to positively charged cores and proton depletion. The study highlighted that such segregation increases R_gb_ but also suggested that controlling dopant distributions—especially at high processing temperatures—can mitigate this effect.

Recent work has also explored Ba­(Ce, Zr)­O_3−δ_ thin-film architectures for micro-SOFCs and miniaturized protonic devices. Epitaxially grown thin films can stabilize the cubic perovskite phase at lower temperatures, improving proton mobility and reducing the impact of grain boundaries. Thin films of Ba­(Ce, Zr)­O_3−δ_-based electrolytes have been fabricated and have shown exceptional proton conductivities and chemical stability, offering a promising pathway for next-generation low-temperature solid oxide cells.^90,166^

In parallel, research on atomic-scale defect chemistry has accelerated. Computational methods such as DFT and MD simulations have provided insights into proton migration mechanisms, dopant–defect interactions, and hydration energetics in Ba­(Ce, Zr)­O_3−δ_ systems.^167,168^ Kang et al.^167^ employed first-principles DFT calculations to systematically investigate doped BaZrO_3−δ_ systems, providing detailed insights into proton migration mechanisms, dopant–defect interactions, and hydration energetics. Their work assessed activation energies for proton hopping, proton formation energies, and carbonate formation thermodynamics across a wide range of trivalent dopants. By combining DFT and kinetic Monte Carlo simulations, they predicted relative proton conductivities and stabilities, revealing trade-offs between mobility and chemical resilience. These theoretical efforts are increasingly complemented by advanced experimental techniques, including neutron scattering, solid-state NMR, and *in situ* X-ray absorption spectroscopy (XAS), which together offer a comprehensive picture of the interplay between structure, defects, and transport.^144,169^ For example, Noferini et al.^169^ employed high-resolution neutron backscattering spectroscopy to study proton dynamics in hydrated BaZr_1–x_M_x_O_3−δ_ (M = Sc, Y, In), revealing detailed insights into jump diffusion mechanisms, activation energies, and dopant-dependent mobility. Their findings complement computational work by confirming temperature-activated long-range proton transport and showing how local structural features influence relaxation processes. This study underscores the critical role of advanced experimental techniques like neutron scattering in resolving the interplay between structure, defects, and transport in proton-conducting perovskites.

Despite significant progress, challenges remain for practical deployment. These include improving sinterability without compromising phase purity, minimizing electronic leakage at elevated temperatures, ensuring mechanical compatibility with electrode materials, and scaling up fabrication processes while maintaining a consistent material quality. Ongoing efforts in combinatorial doping, interface engineering, and process optimization are expected to address these challenges and further advance the performance boundaries of these materials.

In summary, Ba­(Ce, Zr)­O_3−δ_-based proton conductors have successfully bridged the gap between high conductivity and chemical stability, positioning them at the forefront of protonic solid oxide cell development. Future innovations in materials design, processing techniques, and device integration hold great promise for unlocking their full potential in practical energy conversion applications.

#### 2.2.2 A^3+^B^3+^O_3_-Type Perovskite

In contrast to the A^2+^B^4+^O_3_ perovskites, the A^3+^B^3+^O_3_-type perovskites feature trivalent rare-earth cations (e.g., La^3+^) at the A site and trivalent transition-metal or rare-earth cations (e.g., Sc^3+^) at the B site. This compositional family has attracted increasing interest due to its distinct defect chemistry and improved chemical stability under harsh operating conditions compared with its alkaline-earth counterparts.

##### 2.2.2.1 LaScO_3−δ_-Based Oxides

LaScO_3−δ_ and its Sr-doped derivatives (La_1–x_Sr_x_ScO_3−δ_) have recently emerged as promising proton-conducting perovskites, offering a combination of high proton mobility, good chemical stability, and compatibility with PCFC environments. Structurally, these materials crystallize in an orthorhombic perovskite framework (*Pnma* space group), where Sr^2+^ substitution at the La^3+^ site introduces oxygen vacancies that act as key sites for water incorporation and subsequent protonic defect formation.

Experimental studies have demonstrated that La_0.9_Sr_0.1_ScO_3−δ_ achieves proton conductivities of ∼ 3 × 10^–2^ S cm^–1^ at 800 °C under humidified conditions, placing it among the highest-performing La-based proton conductors. The introduction of Sr not only increases the unit cell volume but also enhances proton defect concentration, boosting both the conductivity and the proton mobility, which reaches ∼ 2.5 × 10^–4^ cm^2^ V^–1^ s^–1^ at 800 °C for the x = 0.1 composition. Importantly, under reducing atmospheres, the protonic transport numbers approach unity below 500 °C, indicating nearly pure protonic conduction at intermediate temperatures.^39^

Electrochemical impedance and EMF measurements reveal that the conductivity in La_1–x_Sr_x_ScO_3−δ_ arises from a mixed contribution of protons, oxide ions, and holes, with the dominant carrier type strongly dependent on the atmospheric conditions. As shown in Figure 10e,f, under oxidizing conditions, hole conductivity dominates at high temperatures but diminishes below ∼ 500 °C, while under humid reducing conditions, the system transitions to nearly exclusive protonic transport. The activation energies for proton conduction decrease with increasing Sr content, falling from ∼ 0.69 eV for x = 0.01 to ∼ 0.45 eV for x = 0.15, underscoring the role of dopant-induced vacancies in facilitating proton migration.

Thermogravimetric and hydration analyses further indicate that only ∼ 55–60% of the nominal oxygen vacancies participate in hydration, suggesting the presence of nonequivalent crystallographic oxygen sites and surface heterogeneity effects that partially limit water uptake. Ongoing research is focusing on optimizing the dopant concentration, microstructure, and sintering conditions to improve the effective utilization of oxygen vacancies, enhance grain boundary conductivity (σ_gb_), and further boost the performance of LaScO_3−δ_-based electrolytes in practical electrochemical applications.

#### 2.2.3 Perovskite-Derived Oxides

Perovskite-derived oxides encompass a broad range of materials structurally related to traditional ABO_3_ perovskites but featuring modified stoichiometries, cation ordering, and oxygen deficient frameworks. These structural modifications introduce unique proton-conduction pathways and often improve chemical stability under harsh conditions. Recent advances have highlighted their potential as viable proton-conducting electrolytes with tunable properties accessible through precise compositional and structural adjustments.

##### 2.2.3.1 BaSc_0.8_Mo_0.2_O_3−δ_

BaSc_0.8_Mo_0.2_O_3−δ_ (BSM20) represents a novel class of donor-doped perovskite proton conductors, engineered to overcome the limitations of conventional acceptor-doped oxides. Unlike traditional systems where proton trapping near acceptor dopants limits conductivity, BSM20 stabilizes a cubic perovskite framework through Mo^6+^ donor doping into BaScO_3−δ_, creating a highly disordered lattice with intrinsic oxygen vacancies. This combination enables high proton concentrations, reduced proton trapping, and enhanced three-dimensional proton diffusion, leading to exceptional proton conductivity at intermediate and low temperatures.^170^

Experimental studies show that BSM20 achieves proton conductivities exceeding 0.01 S cm^–1^ at 320 °C, placing it among the top-performing proton conductors and surpassing materials like BaZr_0.8_Y_0.2_O_3−δ_ and BaCe_0.9_Y_0.1_O_3−δ_. Importantly, the high conductivity is maintained within the so-called “Norby gap” (200–500 °C), a temperature range where most oxide conductors fail to combine high conductivity with chemical stability. Neutron diffraction and DFT analyses reveal that protons in BSM20 diffuse primarily around ScO_6_ octahedra, avoiding MoO_6_ regions due to electrostatic repulsion, and migrate through a well-connected three-dimensional network of proton diffusion pathways.^170^

In addition to its remarkable conductivity, BSM20 exhibits outstanding chemical and electrical stability under oxidizing, reducing, and CO_2_-rich atmospheres, making it a robust candidate for use in PCFCs and electrolyzers. Thermogravimetric studies confirm the ability to incorporate substantial water (∼0.32 H per formula unit at 100 °C), with its high proton concentration attributed to the intrinsic oxygen vacancy content (δ ≈ 0.2), which exceeds that of many benchmark materials. Furthermore, ab initio molecular dynamics (AIMD) simulations and isotope exchange measurements confirm that BSM20’s proton diffusion coefficient is significantly higher than that of conventional acceptor-doped systems, with low apparent activation energy (∼0.41 eV), reflecting minimal proton trapping effects.^170^

Current research focuses on refining the synthesis, optimizing Sc/Mo site distributions, and exploring microstructural control strategies to further harness the exceptional protonic properties of the BSM20. The successful demonstration of donor-doped perovskites such as BSM20 opens a promising new pathway for designing highly conductive and chemically stable proton conductors for next-generation intermediate-temperature electrochemical devices.

##### 2.2.3.2 Ba_5_Er_2_Al_2_ZrO_13−δ_

Ba_5_Er_2_Al_2_ZrO_13−δ_ is an emerging hexagonal perovskite-related oxide distinguished by its intrinsically oxygen-deficient layered structure, which enables it to function as a high-performance proton conductor. As Figure 11a shows, the material consists of alternating perovskite-like octahedral layers and oxygen-deficient tetrahedral layers stacked along the *c*-axis, where the oxygen vacancies play a critical role in facilitating water incorporation and generating mobile protonic defects. Remarkably, Ba_5_Er_2_Al_2_ZrO_13−δ_ exhibits proton conductivities exceeding ∼ 10^–3^ S cm^–1^ between 300–600 °C, rivaling those of conventional cubic perovskite proton conductors but without requiring extrinsic acceptor doping (Figure 11b).

**Figure 11:**
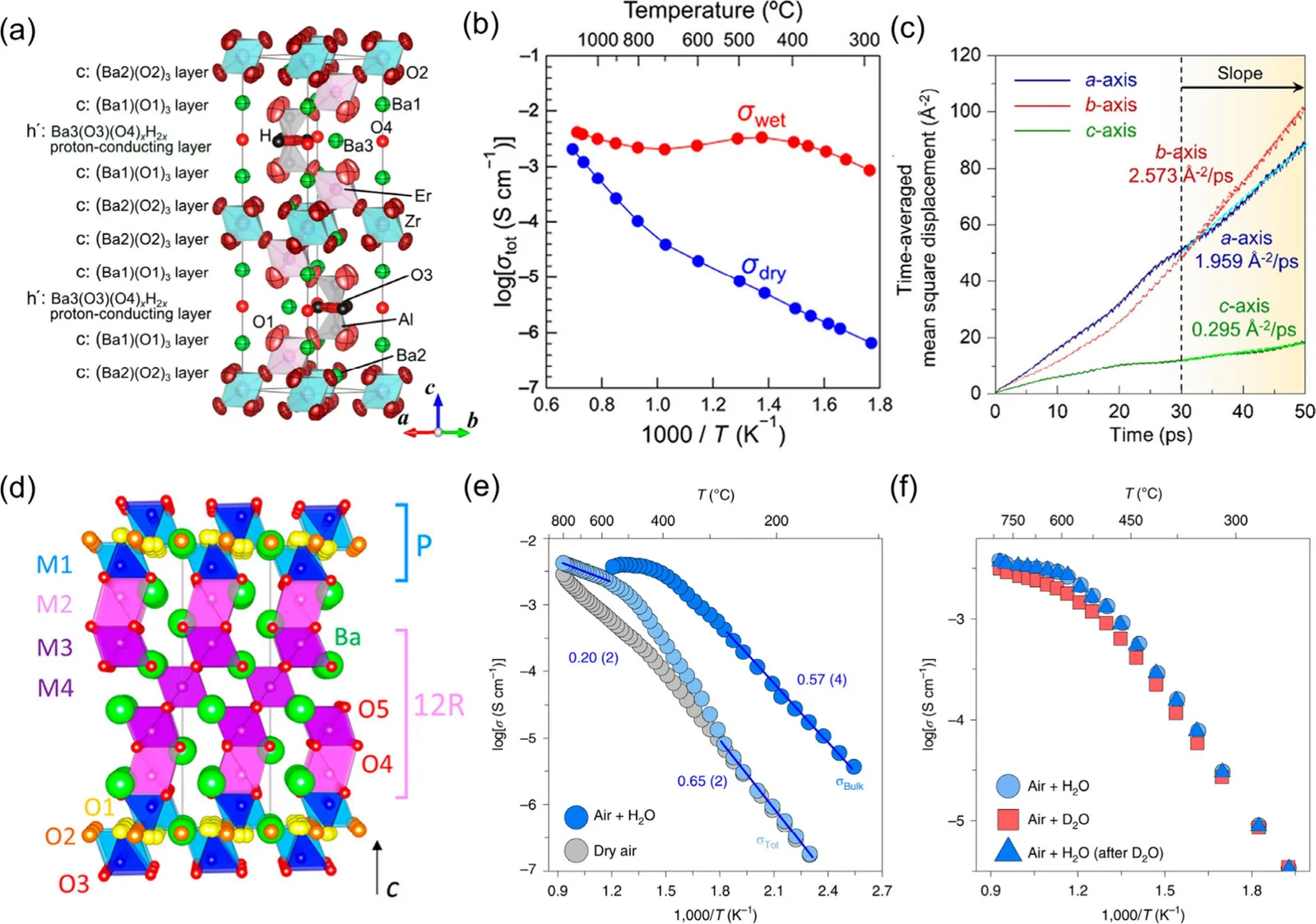
(a) The refined crystal structure of Ba_5_Er_2_Al_2_ZrO_13.23_H_0.46_ obtained from neutron diffraction data collected at 18 °C. Green, pink, blue, gray, light red, dark red, and red ellipsoids represent Ba, Er, Zr, Al, O1, O2, and O3 atoms, respectively; red and black spheres indicate O4 and H atoms. Thermal ellipsoids are shown at the 95% probability level. (b) Arrhenius plots of the total electrical conductivity of Ba_5_Er_2_Al_2_ZrO_13−δ_·xH_2_O measured during cooling in dry and humidified air (pH_2_O = 0.017 atm). Reproduced from ref 171. Copyright 2020 American Chemical Society. (c) Time-averaged mean squared displacement of protons along the a, b, and c crystallographic axes, with slopes estimated over the 30–50 ps interval. Reproduced from ref 172. Copyright 2023 American Chemical Society. (d) Average crystal structure of BNM, comprising an intergrowth of palmierite-like (P) layers and 12R-type hexagonal perovskite blocks. Blue and light blue polyhedra represent M1O_x_ units with mixed tetrahedral and octahedral coordination, arising from partial occupancy of the O1 and O2 oxygen sites. Partial occupancy at M1 and M2 cation sites leads to hybrid trimer stacking sequences consisting of cation vacancies and isolated or face-sharing polyhedral units. (e–f) Arrhenius plots of conductivity for BNM. (e) Comparison of bulk and total conductivities, along with activation energies (eV), in humidified air versus total conductivity in dry air. (f) Demonstration of the isotope effect: total conductivity measured in air + D_2_O shows a reduction relative to that in air + H_2_O. Reproduced with permission from ref 173. Copyright 2020 Springer Nature.

A key feature driving its performance is the selective hydration of the oxygen-deficient layers, which results in high proton concentrations and promotes two-dimensional proton diffusion. Neutron diffraction and bond-valence calculations reveal that protons preferentially localize near O3 sites within these layers, enabling efficient proton hopping mechanisms. Notably, the activation energy for proton conduction is as low as ∼ 0.40 eV in the 300–450 °C range, and the proton transport number approaches unity, indicating nearly pure proton conduction under wet atmospheres.^171^

Recent studies have further revealed pronounced anisotropy in the proton-transport behavior of Ba_5_Er_2_Al_2_ZrO_13−δ_. Advanced thin-film fabrication combined with orientation-controlled grain alignment has demonstrated that proton migration is significantly more favorable in the lateral (ab-plane) direction compared to that in the vertical (*c*-axis) direction (Figure 11c). This anisotropic conduction is attributed to preferential proton migration through the electrostatically neutral perovskite-like layers rather than the tetrahedral layers, as supported by DFT and AIMD simulations. Importantly, this insight suggests that optimizing the texturing and orientation of Ba_5_Er_2_Al_2_ZrO_13−δ_ microstructures may unlock further improvements in its proton conduction performance.^172^

Overall, Ba_5_Er_2_Al_2_ZrO_13−δ_ represents a promising member of the hexagonal perovskite-derived family, offering a rare combination of intrinsic oxygen deficiency, high hydration capacity, anisotropic proton transport, and excellent stability—all without the need for chemical doping. Ongoing research is actively exploring microstructural engineering, surface energy-driven orientation control, and doping strategies to further elevate its potential as a next-generation electrolyte material for protonic ceramic devices.

##### 2.2.3.3 Ba_7_Nb_4_MoO_20−δ_

BNM is a disordered hexagonal perovskite-derived oxide that has emerged as a highly promising dual-ion conductor, exhibiting significant proton and oxide ion conductivity at intermediate temperatures. As shown in Figure 11d, its structure, characterized by an intergrowth of palmierite-like layers and 12R perovskite blocks, accommodates abundant intrinsic cation and anion vacancies, which serve as active sites for both water incorporation and ionic migration. Under humidified conditions, BNM demonstrates bulk proton conductivities reaching ∼ 4.0 mS cm^–1^ at ∼ 510 °C, comparable to those of well-established cubic barium cerates and zirconates (Figure 11e,f).^173^

A key feature of BNM lies in its remarkable structural flexibility, which enables it to accommodate large amounts of water—up to ∼ 0.8 H_2_O molecules per formula unit—through the hydration of oxygen vacancies along the palmierite-like layers. Neutron diffraction and DFT studies have shown that water absorption leads to a reorganization of the local oxygen environments, notably increasing positional disorder and promoting proton delocalization. This gives rise to a frustrated proton sublattice with low-energy diffusion pathways, enabling fast proton transport even in a lower-symmetry hexagonal framework where proton mobility is typically suppressed.^174^

In dry conditions, BNM functions predominantly as an oxide ion conductor, with an oxide ion transport number exceeding 0.99 across a wide pO_2_ window (∼10^–18^–1 atm at 600 °C). Upon exposure to humidified atmospheres, the material undergoes a transition in transport mechanism, with proton conduction becoming dominant above ∼ 300 °C, coinciding with structural rearrangements that reduce activation barriers for proton hopping. Computational studies highlight that the material’s unique combination of tetrahedral and octahedral MO_x_ units, coupled with cationic vacancy disorder, facilitates dynamic reconfigurations that enhance both proton and oxide ion conductivity.

Crucially, BNM also displays excellent chemical and electrical stability under CO_2_-containing and reducing environments, properties that distinguish it from many conventional perovskite electrolytes prone to degradation under such conditions. Current research is actively exploring chemical doping, defect engineering, and microstructural optimization to further boost its performance and unlock its potential as a next-generation dual-ion electrolyte for intermediate-temperature solid oxide and protonic ceramic fuel cells.

##### 2.2.3.4 A_6_B_2_O_11_

A_6_B_2_O_11_-type oxides, including Sr_6_Ta_2_O_11–δ_, Sr_6_Nb_2_O_11–δ_, and Ba_4_Ca_2_Nb_2_O_11–δ_, represent a unique family of perovskite-derived materials characterized by highly oxygen-deficient frameworks and disordered oxygen sublattices. These compounds feature alternating polyhedral units and incomplete oxygen site occupancy (approximately 1/12 vacant), providing abundant sites for hydration and facilitating proton incorporation via dissociative water adsorption. This structural characteristic enables efficient proton conduction, particularly under humidified conditions and positions these oxides as promising candidates for intermediate-temperature protonic ceramic devices.

A key feature of A_6_B_2_O_11_ oxides is their ability to incorporate significant amounts of water, leading to the formation of hydroxyl species and protonic defects that dominate charge transport at moderate temperatures (∼300–600 °C). For instance, Sr_6_Ta_2_O_11–δ_ undergoes a phase transition from cubic (Fm3m) to orthorhombic (*Fmmm*) symmetry upon hydration, typically observed near 450–475 °C at pH_2_O ≈ 0.02 atm, which marks the onset of a new hydrate-like phase with enhanced proton mobility. Similar behavior has been reported in Ba_4_Ca_2_Nb_2_O_11–δ_, where hydration-induced phase changes are accompanied by significant increases in proton conductivity.

Experimental investigations using thermogravimetry, dilatometry, and XRD have shown that these materials can accommodate up to ∼ 1 mol H_2_O per formula unit, approaching the theoretical hydration limit, and maintaining stable proton conduction pathways under operational conditions. Importantly, the proton conductivity increases monotonically with rising oxygen vacancy concentration, and the activation energy for proton transport typically falls within the range of ∼ 0.5–0.6 eV, consistent with other oxygen-deficient perovskites.^175^

Recent research has emphasized the critical role of cation ordering and lattice dynamics in tuning the hydration behavior and transport properties of A_6_B_2_O_11_ oxides. For example, in (Ba_1–y_Ca_y_)_6_Nb_2_O_11_ solid solutions, increasing the Ba content leads to lattice expansion, which reduces the oxygen migration energy and concomitantly boosts both oxygen-ion and proton conductivity. Ongoing studies are exploring compositional tuning strategies, such as partial B-site substitutions and defect engineering, to further enhance the protonic performance and chemical durability of these materials under CO_2_- and H_2_O-containing atmospheres.^176^

Overall, A_6_B_2_O_11_-type oxides offer a compelling platform for next-generation proton-conducting electrolytes, combining structural flexibility, high hydration capacity, and tunable defect chemistry to meet the demanding requirements of solid oxide electrochemical devices.

### 2.3 Double Perovskite Oxides

Double perovskites, with the general formula AA′B_2_O_6_ or A_2_B′B″O_6_, represent a structurally expanded class of perovskite-related proton conductors characterized by the ordered arrangement of distinct B-site cations. Compared to single perovskites, double perovskites typically offer enhanced chemical stability and unique proton transport properties due to their ordered cation frameworks and tailored defect landscapes. The deliberate cation ordering creates distinct crystallographic environments, facilitating controlled doping and optimizing hydration behavior—both critical for effective proton incorporation and transport.

#### 2.3.1 Ca­(Ba)_2_B’Nb_2–x_O_6−δ_-Based Oxides

Ca­(Ba)_2_B′Nb_2–x_O_6_-type double perovskites represent a promising family of proton-conducting materials, characterized by their A_3_B′B″_2_O_9−δ_ stoichiometry and distinctive B-site ordering. Among these, Ba_3_Ca_1.18_Nb_1.82_O_9−δ_ (BCN18) and related compositions have attracted significant attention due to their combination of high proton conductivity, chemical robustness, and structural tunability.

The crystal structure of these materials features a complex perovskite framework where Ca^2+^ and Nb^5+^ occupy B′ and B″ sites, respectively, often in an ordered or partially disordered fashion, depending on stoichiometry and doping. Oxygen vacancies, introduced through cation off-stoichiometry (e.g., Ca excess) or acceptor doping (e.g., Y^3+^), play a central role in enabling proton incorporation via dissociative water adsorption. Notably, Y-doped BCN18 achieves proton conductivities up to ∼ 5.3 × 10^–3^ S cm^–1^ at 600 °C, approximately 2.4 times higher than undoped BCN18, due to enhanced oxygen vacancy content and disrupted B-site ordering, which facilitates improved defect mobility and proton transport (Figure 12a). Meanwhile, BCN-based materials exhibit excellent chemical stability in CO_2_-containing environments and maintain a high t_i_ (>0.9) at temperatures below 650 °C (Figure 12b,c).^40^

**Figure 12:**
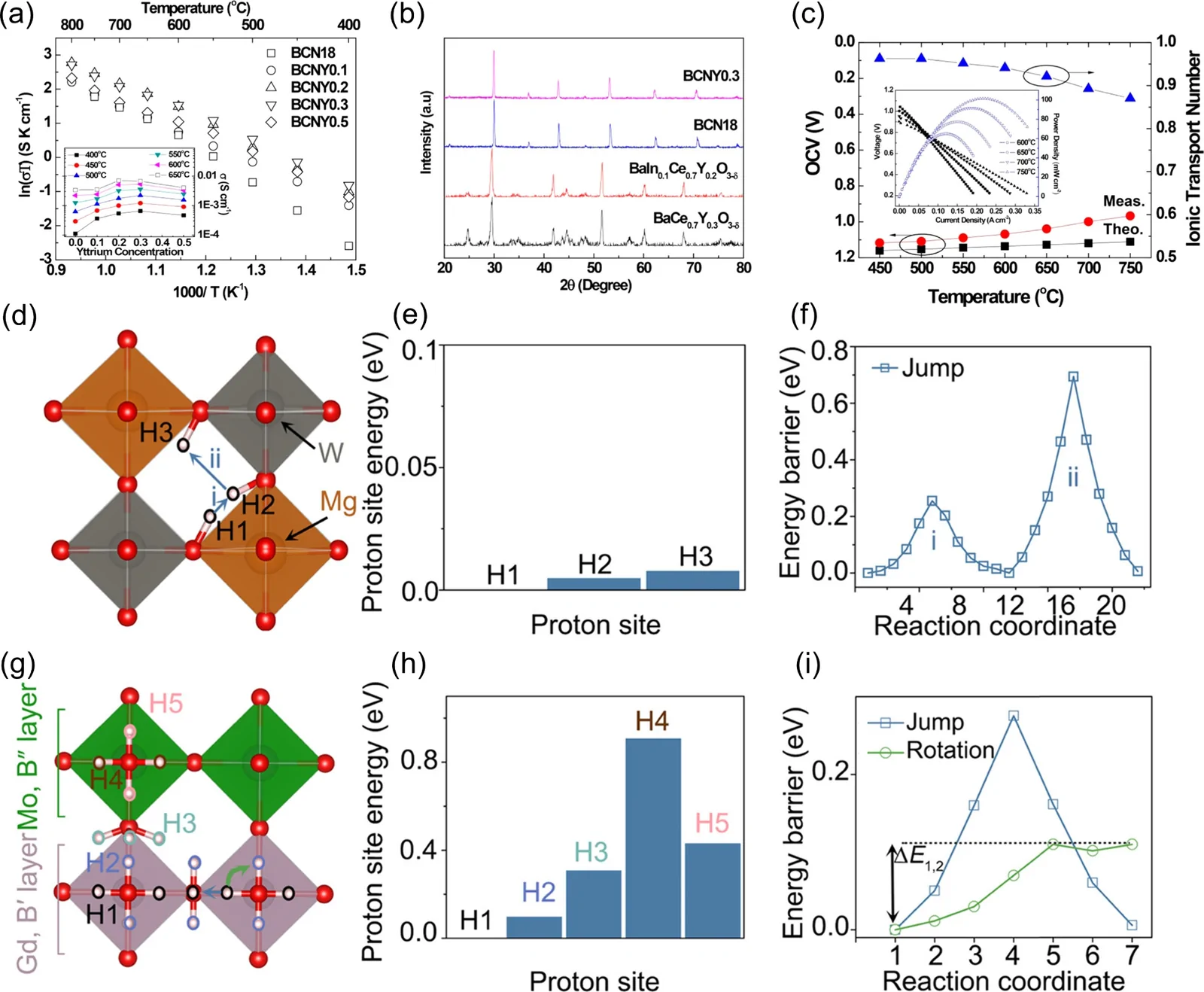
(a) Arrhenius plots of the total conductivity of the samples measured in humidified air. The inset shows the conductivity as a function of yttrium concentration. (b) XRD patterns of sintered pellets after exposure to a gas mixture containing 3% CO_2_ and 3% H_2_O in air at 700 °C for 24 h. For comparison, XRD patterns of BaCe_0.7_Y_0.3_O_3−δ_ and BaIn_0.1_Ce_0.7_Y_0.2_O_3−δ_ powders treated under identical conditions are also shown. (c) Theoretical and experimentally measured OCV values for Ba_3_Ca_1.18_Nb_1.52_Y_0.3_O_9−δ_ in a 3 vol % H_2_O–H_2_/Pt|Ba_3_Ca_1.18_Nb_1.52_Y_0.3_O_9−δ_|Pt/air configuration, along with the corresponding ionic transport number. The inset presents the power density as a function of current density for a Ni–BZCYYb1711|Ba_3_Ca_1.18_Nb_1.52_Y_0.3_O_9−δ_|Ba_0.9_Co_0.7_Fe_0.2_Nb_0.1_O_3−δ_ single cell. Reproduced from ref 40. Copyright 2014 American Chemical Society. (d–f) Proton conduction in the double-perovskite Ba_2_MgWO_6−δ_ with B-site rock-salt ordering: (d) schematic of proton sites (white spheres) and migration pathway (arrow), (e) proton site energies, and (f) the corresponding migration energy profile. (g–i) Proton conduction in the double-perovskite Ba_2_GdMoO_6_ with B-site cation layered ordering: (g) crystal structure highlighting the GdO_6_ octahedral layer, (h) proton site energies, and (i) migration energy profile within the GdO_6_ layer. Reproduced from ref 178. Copyright 2021 American Chemical Society.

Experimental investigations confirm that materials like Ba_4.3_Ta_1.7_O_8.55_, an analog within the same family, exhibit total conductivities on the order of ∼ 10^–5^–10^–4^ S cm^–1^ across 400–800 °C under humid atmospheres, with protonic transport numbers exceeding 0.6 at 700 °C. These values, coupled with a favorable hydration enthalpy (−79.9 kJ mol^–1^), highlight the practical potential of these double perovskites for SOFCs, hydrogen separation membranes, and electrochemical sensors.^177^

Recent computational studies using first-principles methods have provided valuable design insights into this material class. It was found that double perovskites with layered B-site ordering, such as Ca­(Ba)_2_B′NbO_6−δ_, benefit from reduced proton migration barriers compared to rock-salt-type ordered systems. This advantage arises because the B′-site cations (typically with lower oxidation states) preferentially host proton sites, while the layered architecture offers continuous low-energy migration pathways within the B′O_6_ layers (Figure 12d–i). Computational screening has identified several candidate compositions in this family with both strong hydrogen incorporation capability and fast proton diffusion, underscoring the importance of balancing cation size, oxidation state, and site ordering to minimize proton trapping effects.^178^

Ongoing research is focused on further enhancing their performance through targeted cation substitutions, precise control of oxygen vacancy distributions, and optimization of microstructural features, including grain size and densification. Together, these strategies are poised to unlock the full potential of Ca­(Ba)_2_B′NbO_6−δ_-type double perovskites as versatile, high-performance proton conductors for next-generation electrochemical devices.

### 2.4 Ruddlesden–Popper Phase Oxides

Ruddlesden–Popper phase oxides, characterized by a layered structure typically denoted as A_n+1_B_n_O_3n+1_, represent another prominent family of proton conductors distinct from conventional perovskites. Their architectures consist of alternating ABO_3_ perovskite layers and AO rock-salt layers, creating intrinsic two-dimensional pathways for proton conduction. The layered nature of these materials not only enhances chemical stability under CO_2_- and humidified conditions but also provides tunable proton conductivity through targeted doping strategies.

#### 2.4.1 BaNd­(Sc)­InO_4−δ_-Based Oxides

BaNdInO_4−δ_ and BaNdScO_4−δ_ represent a family of modular layered perovskites that have recently emerged as promising proton-conducting materials. These compounds adopt a monoclinic (P21/c, BaNdInO_4−δ_) or orthorhombic (*Cmcm*, BaNdScO_4−δ_) structure, featuring alternating perovskite-like slabs and rare-earth oxide-like layers. Their unique layered architectures provide abundant oxygen vacancies and facilitate water incorporation, making them attractive hosts for proton transport upon acceptor doping.

In BaNdInO_4−δ_, calcium substitution at the Nd site introduces oxygen vacancies that dramatically enhance the material’s conductivity. Experimental studies report that 20% Ca-doped samples (BaNd_0.8_Ca_0.2_InO_4−δ_) achieve total conductivities up to 2.6 × 10^–3^ S cm^–1^ at 750 °C, with significantly higher protonic contributions under humid conditions (e.g., ∼ 1.3 × 10^–4^ S cm^–1^ at 500 °C in ∼ 3% H_2_O atmosphere) compared to dry conditions. Impedance spectroscopy, DC conductivity measurements, and thermogravimetric analyses confirm that water incorporation decreases the activation energy for bulk conduction (from ∼ 0.76 eV to ∼ 0.68 eV), with hydrated samples exhibiting measurable lattice expansion.^41^

BaNdScO_4−δ_, part of the orthorhombic BaNdInO_4−δ_-type structural family, also demonstrates excellent proton-conducting capabilities when acceptor-doped. Neutron diffraction and DFT calculations reveal that protons preferentially coordinate with oxygen sites near the interface between perovskite-like and rare-earth oxide-like layers, where both inter- and intraoctahedral hopping mechanisms contribute to the overall proton conduction (Figure 13a,b). Interestingly, DFT simulations suggest slightly lower energy barriers for proton migration along the *c*-axis, indicating a directional preference at low temperatures and a potential for three-dimensional conduction at elevated temperatures. Notably, as shown in Figure 13c, BaNd_0.8_Ca_0.2_ScO_4−δ_ shows a 39-fold increase in total conductivity compared to the undoped parent compound at 800 °C, reaching ∼ 1.2 × 10^–2^ S cm^–1^, with a clear enhancement under wet atmospheres attributable to proton transport.^179^

**Figure 13:**
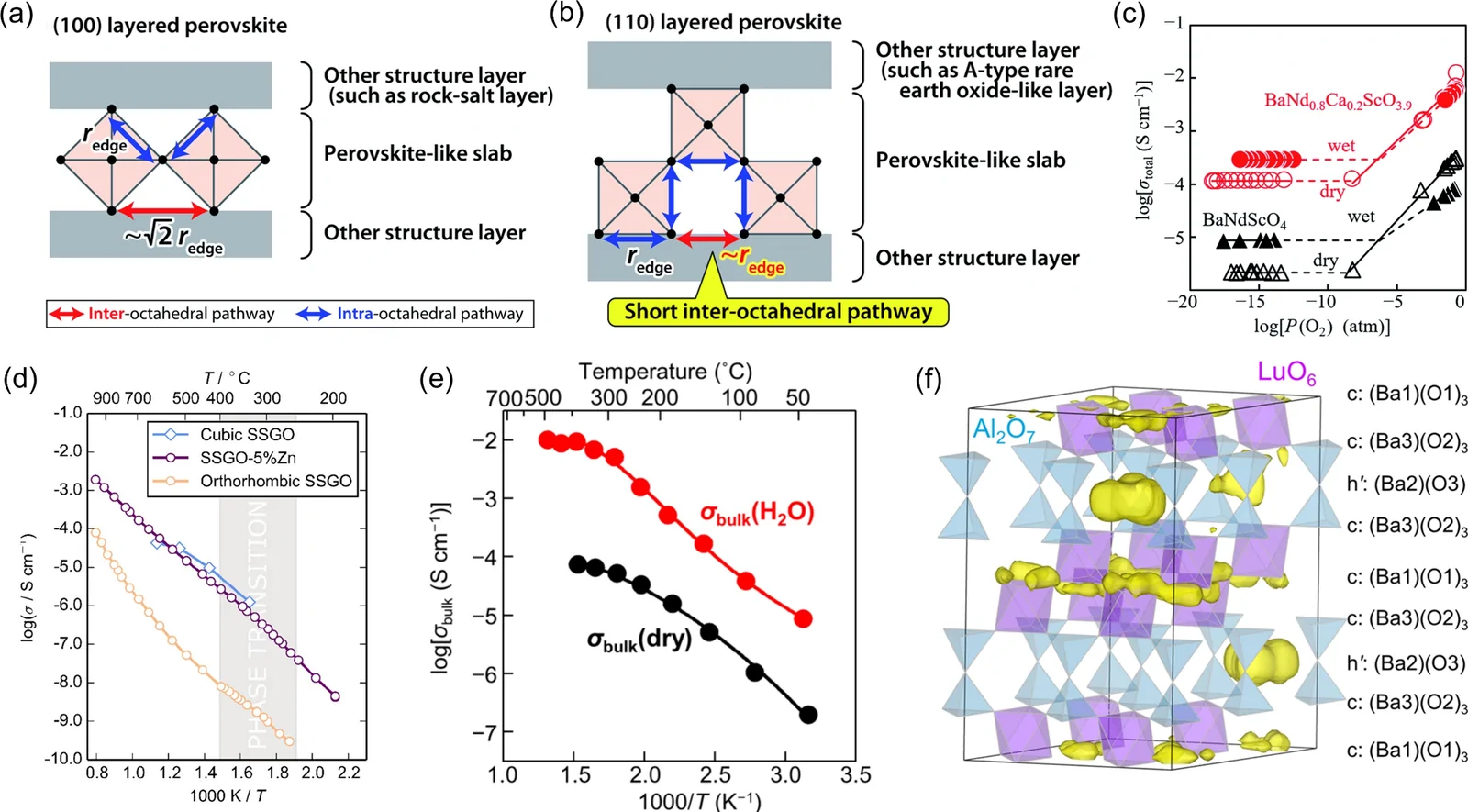
(a,b) Schematic illustrations of interoctahedral and intraoctahedral proton hopping pathways in layered perovskites: (a) a (100)-oriented layered perovskite without octahedral tilting and (b) a (110)-oriented layered perovskite. Black spheres represent oxygen atoms, and orange squares represent the MX_6_ octahedra in the AMX_3_ perovskite structure. In this notation, A and M are relatively large and small cations, respectively, and X denotes an anion. The O–O edge distance of the octahedron (r_edge_) corresponds to the intraoctahedral hopping distance. A-site cations are omitted for clarity. (c) Dependence of total conductivity on oxygen partial pressure for BaNd_0.8_Ca_0.2_ScO_4−δ_ (red) and BaNdScO_4−δ_ (black) measured at 800 °C. Open and closed symbols represent measurements in dry and humid atmospheres, respectively. Reproduced with permission from ref 179. Copyright 2021 RSC. (d) Arrhenius plots of the conductivity in air for brownmillerite-type orthorhombic Sr_2_ScGaO_5−δ_, cubic Sr_2_ScGaO_5−δ_, and orthorhombic Sr_2_ScGaO_5−δ_ doped with 5% Zn. Reproduced from ref 42. Copyright 2019 American Chemical Society. (e) Arrhenius plots of the σ_bulk_ of Ba_2_LuAlO_5−δ_ under dry and humidified N_2_ gas flow. (f) Proton probability density distribution in Ba_2_LuAlO_5−δ_·0.125H_2_O. The yellow isosurface (2.0 × 10^–4^ Å^–3^) shows the proton probability density obtained from AIMD simulations at 1473 K. Reproduced from ref 43. Available under a CC-BY 4.0 license. Copyright 2023 Springer Nature Riho Morikawa et al.

Collectively, these BaNd­(Sc)­InO_4−δ_-type layered perovskites present a compelling platform for developing next-generation proton-conducting oxides, combining high hydration capacity, tunable defect chemistry, and strong chemical and thermal stability. Current research efforts focus on optimizing dopant content, microstructure, and hydration strategies to further enhance their conductivity and operational durability in practical electrochemical devices.

### 2.5 Brownmillerite

Brownmillerite oxides, with the general formula A_2_B_2_O_5_, feature a unique oxygen-deficient structure derived from the parent perovskite through ordered oxygen-vacancy layers. These intrinsic vacancy channels provide direct pathways for proton conduction, enabling distinct transport mechanisms compared with conventional perovskites. Brownmillerites often exhibit high ionic conductivity due to their built-in vacancy networks, although achieving optimal proton conductivity and structural stability remains an ongoing research challenge.

#### 2.5.1 Ba_2_In_2_O_5−δ_-Based Oxides

Ba_2_In_2_O_5−δ_ is a brownmillerite-type oxide that has emerged as a highly promising proton- and oxide-ion-conducting material due to its rich structural flexibility and defect chemistry. The A_2_B_2_O_5_ stoichiometry corresponds to one-sixth of the oxygen sites in the ideal perovskite being vacant, with these vacancies ordering into parallel layers to minimize lattice strain and electrostatic energy.^42^ Consequently, the BO_6_ octahedra in one layer maintain full coordination, whereas the adjacent layer presents BO_4_ tetrahedral coordination hosting ordered oxygen vacancies. The structure is often termed “pseudocubic”: although octahedral and tetrahedral layers alternate with long-range ordering, the average lattice parameters remain nearly cubic, and the framework preserves key topological characteristics of the parent perovskite. Local departures from ideal cubic symmetry originate from rotational, tilting, and distortional effects induced by the accommodation of tetrahedral units.^180^ In its dehydrated state, Ba_2_In_2_O_5−δ_ features alternating layers of InO_6_ octahedra and InO_4_ tetrahedra, with intrinsic oxygen vacancies aligned along the tetrahedral layers. Upon hydration under wet conditions, Ba_2_In_2_O_5−δ_ transforms into a pseudocubic BaInO_3_H phase, where water dissociates into hydroxyl (OH^–^) groups and protons, effectively filling the vacant tetrahedral layers and enabling significant proton conduction.

Experimental and computational studies using infrared (IR) spectroscopy, inelastic neutron scattering (INS), and AIMD have revealed two dominant proton sites in the hydrated structure: H(1), characterized by intraoctahedral hydrogen bonding, and H(2), associated with interoctahedral hydrogen bridges. These dual proton sites enable a complex and anisotropic proton transport mechanism, particularly in partially hydrated phases, where protons tend to segregate into oxygen-rich domains.^180^

Recent advances have focused on enhancing the total conductivity of Ba_2_In_2_O_5−δ_ through strategic doping. For example, codoping with Zr^4+^ and Zn^2+^ has been shown to create Frenkel-type defect clusters, which promote local disordering of oxygen vacancies even at relatively low temperatures (∼923 K) and significantly boost the oxide-ion conductivity without inducing large lattice volume changes. These doped systems maintain structural integrity across wide temperature ranges, avoiding the ∼ 3–4% volume expansion typically observed in undoped Ba_2_In_2_O_5−δ_ during the order–disorder transition of oxygen vacancies. Remarkably, electrical conductivities as high as ∼ 3 × 10^–2^ S cm^–1^ at 700 °C have been reported, placing Ba_2_(In_0.7_(Zn_0.5_Zr_0.5_)_0.3_)_2_O_5−δ_ among the leading brownmillerite-type proton conductors for intermediate-temperature applications.^181^

The combination of hydration-driven proton transport and defect-enhanced oxide-ion conduction positions Ba_2_In_2_O_5−δ_ as a versatile dual-ion conductor, suitable for PCFCs, oxygen separation membranes, and catalytic applications. Ongoing research is exploring microstructural engineering, doping strategies, and defect modeling to further refine its performance and durability under real-world operating conditions.

#### 2.5.2 Sr_2_ScGaO_5−δ_-Based Oxides

Sr_2_ScGaO_5−δ_ is a brownmillerite-type oxide that has recently attracted attention as a promising mixed ionic conductor, primarily for its oxide ion conductivity but also for its capacity to support proton conduction under certain conditions. Structurally, it features alternating ScO_6_ octahedral layers and GaO_4_ tetrahedral chains, arranged within an oxygen-deficient perovskite framework. This architecture provides a network of ordered and disordered oxygen vacancies, which are central to its ionic transport behavior.^42^

A key characteristic of Sr_2_ScGaO_5−δ_ is its temperature-dependent structural dynamics. Below ∼ 250–300 °C, the material adopts a noncentrosymmetric polar orthorhombic phase (space group I2mb), where the (GaO_4_/_2_)_∞_ chains are fully ordered. As the temperature increases, the system undergoes a second-order phase transition to a centrosymmetric orthorhombic phase (space group *Imma*), during which the tetrahedral chains become orientationally disordered. Neutron scattering and pair distribution function analyses confirm that GaO_4_ tetrahedra remain largely rigid across this transition, while ScO_6_ octahedra accommodate local distortions, reflecting the system’s capacity for dynamic rearrangement under thermal excitation.

Impedance spectroscopy measurements show that Sr_2_ScGaO_5−δ_ has modest σ_bulk_, primarily due to oxide ion transport. The activation energy for ionic conduction decreases slightly (from ∼ 0.9 eV to ∼ 0.7 eV) across the phase transition, suggesting that the reorientation of tetrahedral chains assists oxide ion mobility by lowering migration barriers. Interestingly, while previously classified as a pure oxide ion conductor, recent measurements under wet atmospheres reveal a detectable protonic contribution at lower temperatures, particularly below ∼ 500 °C, as evidenced by small increases in conductivity under humidified conditions and confirmed by TGA showing minor water uptake (∼0.03–0.04 H_2_O per formula unit).

Efforts to enhance the conductivity of Sr_2_ScGaO_5−δ_ have included partial Zn^2+^ substitution for Sc^3+^, creating additional oxygen vacancies within the octahedral layers. As shown in Figure 13d, Zn-doped Sr_2_ScGaO_5−δ_ (e.g., Sr_2_Sc_0.95_Zn_0.05_GaO_5−δ_) exhibits conductivity improvements of up to 2 orders of magnitude across the measured temperature range, on the order of ∼ 10^–5^ S cm^–1^ at 400 °C, with the activation energy for oxide ion conduction decreasing correspondingly. These enhancements highlight the critical role of extrinsic defect engineering in tuning the ionic transport properties of brownmillerite-type electrolytes.

Overall, Sr_2_ScGaO_5−δ_ exemplifies the complex interplay between structural order–disorder transitions, lattice dynamics, and ionic transport in layered oxygen-deficient materials. Ongoing research aims to exploit these features by fine-tuning the defect chemistry and microstructure to develop highly efficient oxide ion or mixed ionic conductors for advanced electrochemical applications.

#### 2.5.3 Ba_2_LuAlO_5−δ_-Based Oxides

Ba_2_LuAlO_5−δ_ is a newly discovered hexagonal perovskite-related oxide that has garnered significant attention due to its exceptionally high proton conductivity and chemical stability, achieved without any chemical doping. Structurally, it features a distinctive combination of cubic close-packed (c) layers and highly oxygen-deficient hexagonal close-packed (h′) BaO layers, forming a (ccch′)_2_ stacking sequence. This framework provides an intrinsic reservoir of oxygen vacancies, enabling substantial water uptake (x ≈ 0.50 in Ba_2_LuAlO_5−δ_·xH_2_O) and efficient generation of mobile protonic defects upon hydration.

As shown in Figure 13e, experimental studies report bulk proton conductivities as high as 10^–2^ S cm^–1^ at 487 °C and 1.5 × 10^–3^ S cm^–1^ at 232 °C, placing Ba_2_LuAlO_5−δ_ among the most conductive known protonic oxides. Notably, the activation energy for proton transport is exceptionally low (∼0.36 eV), outperforming conventional doped perovskites such as BaZr_0.8_Y_0.2_O_2.9_. Impedance and isotope substitution measurements confirm the dominance of proton conduction under wet conditions, with proton transport numbers approaching unity over a wide temperature range (300–800 °C). Importantly, AIMD simulations reveal that protons preferentially migrate near the cubic close-packed c layers at the interface of LuO_6_ octahedra, while a portion of protons remains trapped within the h′ layers, supporting a mixed dimensionality in the conduction pathways (Figure 13f).^43^

Beyond its impressive conductivity, Ba_2_LuAlO_5–δ_ exhibits remarkable chemical stability under oxidizing, reducing, CO_2_-containing, and humidified atmospheres, with no significant phase changes observed after prolonged annealing at 400–500 °C. This robustness makes it an attractive electrolyte candidate for intermediate-temperature PCFCs and other electrochemical devices. Moreover, its intrinsic oxygen vacancy architecture avoids the need for extrinsic acceptor doping, thereby mitigating common issues such as proton-dopant association, compositional inhomogeneity, and interfacial reactivity. This emerging material highlights the importance of exploring new material chemistries and alternative crystal structures as a potential route to overcome the long-standing conductivity-stability trade-off of conventional acceptor-doped BaCeO_3−δ_ and BaZrO_3−δ_ electrolytes. However, Ba_2_LuAlO_5–δ_ remains at an early stage of investigation. Further fundamental studies are needed to elucidate its defect chemistry, hydration behavior, and proton transport mechanisms, and its practical feasibility as an electrolyte has yet to be demonstrated in single-cell device configurations.

### 2.6 Scheelite-Type Oxides

Scheelite-type oxides, with the general formula ABO_4_, have emerged as attractive alternative proton-conducting electrolytes owing to their excellent chemical stability, moderate proton conductivity, and intrinsic structural versatility. Their characteristic tetragonal scheelite structure (space group I41/a), composed of isolated BO_4_ tetrahedral units, provides pathways for proton conduction primarily through oxygen vacancies introduced via targeted doping strategies. Compared to perovskite-based electrolytes, scheelite oxides typically exhibit superior resistance to carbonation and hydration, making them promising candidates for intermediate-temperature proton-conducting applications.

#### 2.6.1 (La, Ba)­GaO_4−δ_-Based Oxides

(La, Ba)­GaO_4−δ_ belongs to a family of layered oxides with orthorhombic symmetry (space group *P*2_1_2_1_2_1_) that have recently drawn attention as promising proton-conducting materials. Traditionally, La_1–x_Ba_1+x_GaO_4–0.5x_ compounds exhibited modest proton conductivities (∼4 × 10^–4^ S cm^–1^ at 600 °C), significantly lower than those of benchmark perovskite-based proton conductors such as BaCeO_3−δ_- or BaZrO_3−δ_-based systems. This limited conductivity hindered their practical deployment despite their structural stability and the detailed understanding of their oxygen vacancy accommodation and conduction mechanisms.

Recent advancements have dramatically improved the performance of these materials through lithium doping on the Ga site, forming LaBa_1–x_Li_x_O_4–x_ compositions. Li^+^ substitution introduces a substantial increase in oxygen vacancy concentration, coupled with a structural phase transition from the original noncentrosymmetric *P*2_1_2_21_2_1_ phase to a centrosymmetric *Pcca* symmetry, which significantly enhances proton mobility. As a result, Li-doped LaBaGaO_4_ samples (e.g., x = 0.3) achieve proton conductivities of 6.38 × 10^–3^ S cm^–1^ at 600 °C—more than an order of magnitude higher than their undoped counterparts and comparable to the best BaCeO_3−δ_-based proton conductors (Figure 14a).^44^

**Figure 14:**
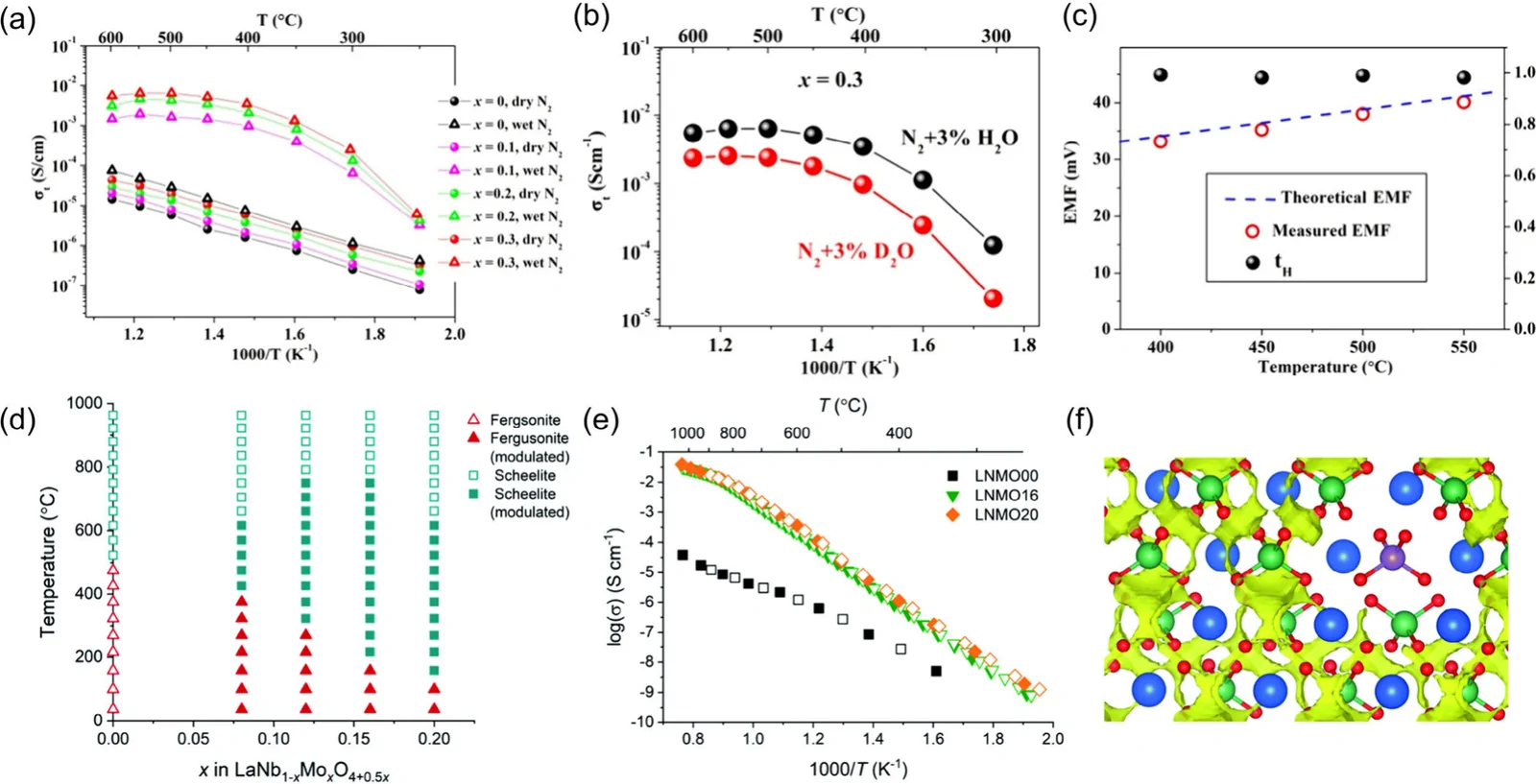
(a) Total conductivities of LaBaGa_1–x_Li_x_O_4–x_ (0 ≤ x ≤ 0.3) measured under dry and humidified N_2_ atmospheres. (b) Conductivity of the x = 0.3 composition measured under N_2_ + H_2_O and N_2_ + D_2_O conditions, highlighting the isotope effect. (c) Comparison of theoretical and experimentally measured EMF values and the corresponding proton transference number at various temperatures. Reproduced from ref 44. Copyright 2024 American Chemical Society. (d) Phase diagram of LaNb_1–x_Mo_x_O_4+0.5x_ constructed from variable-temperature X-ray diffraction data. (e) Total conductivity of selected LaNb_1–x_Mo_x_O_4+0.5x_ samples measured in air. Data obtained during heating and cooling cycles are represented by filled and open symbols, respectively. (f) Section of a bond valence energy landscape map calculated for a lightly doped supercell of scheelite-type LaNb_1–x_Mo_x_O_4+0.5x_ at 705 °C, shown in the vicinity of a Mo^6+^ dopant. Atomic species are represented as blue (La), green (Nb), purple (Mo), and red (O) spheres. The yellow isosurface corresponds to an energy level of 0.90 eV above the global minimum, delineating a continuous low-energy migration pathway. Reproduced with permission from ref 182. Copyright 2021 RSC.

As shown in Figure 14b,c, detailed impedance and EMF measurements confirm that the dominant charge carriers under humidified conditions are protons, as evidenced by a strong hydrogen–deuterium (H/D) isotope effect and negligible electronic conductivity across varied oxygen partial pressures. Furthermore, TGA indicates that these materials can incorporate significant amounts of water (up to ∼ 0.3 H_2_O per formula unit), contributing to the expanded lattice volume and facilitating robust proton transport pathways. Notably, the activation energy for proton conduction in Li-doped LaBaGaO_4−δ_ is remarkably low (0.26–0.32 eV between 400–550 °C), positioning these materials among the most efficient oxide proton conductors reported to date.

Ongoing research is focusing on optimizing the lithium content, microstructural control, and hydration behavior to further improve conductivity and long-term stability under operating conditions. The Li doping strategy for LaBaGaO_4_ not only establishes these materials as promising candidates for PCFC electrolytes but also provides a valuable blueprint for enhancing proton conduction in other gallium-containing oxide systems.

#### 2.6.2 LaNbO_4−δ_-Based Oxides

LaNbO_4−δ_ is an emerging proton-conducting oxide belonging to the family of orthoniobates, offering a distinctive combination of protonic conductivity and excellent chemical stability under CO_2_- and H_2_O-containing environments. Structurally, LaNbO_4−δ_ adopts a monoclinic fergusonite-type configuration at low temperatures, transitioning to a tetragonal scheelite-type phase above ∼ 500–530 °C. This fergusonite-scheelite phase transition plays a critical role in determining its transport and mechanical properties, with implications for both protonic and oxide-ion conduction.^183^

Acceptor-doped LaNbO_4−δ_ (e.g., with Ca^2+^ or Sr^2+^) exhibits notable proton conductivity under wet conditions, achieving maximum protonic conductivities of ∼ 0.001 S cm^–1^ at ∼ 950 °C, comparable to other intermediate-temperature protonic ceramics but with superior CO_2_ stability. Proton transport in LaNbO_4−δ_ proceeds via the incorporation of protons as hydroxide defects (OH_O_^•^) into pre-existing oxygen vacancies, with the conductivity strongly dependent on water vapor pressure. Impedance spectroscopy and EMF measurements reveal that the protonic contribution dominates under reducing and humidified conditions up to ∼ 1100 °C, while electronic conduction becomes significant under highly oxidizing conditions at elevated temperatures.^182^

As shown in Figure 14d,e, recent studies have shown that Mo-doped LaNb_1–x_Mo_x_O_4+0.5x_ significantly enhances its oxide-ion conductivity, raising total conductivities by several orders of magnitude (up to ∼ 2.6 × 10^–2^ S cm^–1^ at 900 °C for 20% Mo) while maintaining good redox stability and compatibility with common SOFC electrodes.^182^ Bond valence energy landscape analyses and DFT simulations highlight that the fergusonite-to-scheelite transition is driven by Nb atom displacive modes and has a quasi-first-order character, with potential impacts on ionic mobility pathways (Figure 14f).^182^

Importantly, LaNbO_4_ demonstrates excellent chemical compatibility with various perovskite-type cathode materials (LaMnO_3−δ_, LaFeO_3−δ_, LaCoO_3−δ_) and Ni-based electrodes, making it an attractive candidate for integration into SOFC architectures. Its chemical robustness against CO_2_ attack and reduced reactivity compared to barium- or strontium-based perovskites make LaNbO_4−δ_ a valuable alternative electrolyte for long-term, stable electrochemical operation. Ongoing research is focused on improving sinterability, understanding interfacial properties, and optimizing microstructures to fully harness its protonic and oxide-ionic transport capabilities.^184^

#### 2.6.3 SrWO_4−δ_-Based Oxides

Scheelite-type SrWO_4−δ_ represents a promising class of proton-conducting materials explored for SOFC electrolyte applications. These materials crystallize in a tetragonal scheelite structure (space group I41/a) and feature fully occupied A-sites with Sr^2+^ or Ba^2+^ and B-sites with W^6+^, creating a robust and dense oxide framework. The substitution of larger Ba^2+^ cations into the SrWO_4−δ_ lattice expands the unit cell volume and improves sinterability, yielding highly dense ceramic electrolytes with relative densities up to ∼ 96%, as confirmed by XRD and SEM analyses.

TGA has shown that SrWO_4−δ_ compounds exhibit significant proton uptake in the intermediate-temperature range, with Ba doping enhancing the water absorption and proton incorporation. This hydration leads to the formation of mobile hydroxyl species that facilitate proton conduction, particularly under wet atmospheres. The Fourier transform infrared (FTIR) spectra further confirm the presence of surface −OH groups, which play a key role in mediating proton transport. Notably, impedance spectroscopy measurements indicate that the total conductivity improves with increasing Ba content, with Sr_0.7_Ba_0.3_WO_4−δ_ achieving the highest protonic conductivity (∼1.9 × 10^–6^ S cm^–1^) at 1000 °C in wet argon atmospheres. While these conductivities are lower than those of state-of-the-art perovskite electrolytes, the materials’ high density, phase stability, and chemical robustness make them attractive candidates for further development.^185^

Importantly, the activation energy for proton conduction decreases with Ba doping, dropping from 1.4 eV (x = 0.1) to ∼ 1.03 eV (x = 0.3), suggesting enhanced proton mobility and reduced migration barriers. Moreover, these scheelite-type materials demonstrate stable phase retention under hydrogen and hydrated environments, indicating good chemical compatibility and durability under SOFC-relevant conditions. Ongoing research focuses on improving the intrinsic conductivity of SrWO_4−δ_ by exploring A- or B-site doping strategies, optimizing grain boundary characteristics, and engineering microstructures to minimize R_gb_ and maximize bulk transport.

Overall, SrWO_4−δ_ scheelite-type oxides offer an intriguing alternative platform for intermediate-temperature proton-conducting applications, combining mechanical and chemical stability with tunable protonic transport properties that warrant further exploration.

### 2.7 Pyrochlore Type Oxides

Pyrochlore oxides are a class of complex materials characterized by the general formula A_2_B_2_O_7_, in which the A-site is typically occupied by 8-fold coordinated rare-earth elements, including La, Pr, Nd, Sm, Eu, Gd, Tb, Dy, Ho, Er, Y, Yb, Lu, and alkali-earth metal, alkaline metal elements. In contrast, the B-site hosts smaller 6-fold coordinated cations like Ce, Zr, Sn, Ti, Hf, Nb, Ta, and Ru.^186^ An alternative representation of the chemical formula, B_2_O_6_·A_2_O′, reflects the presence of two distinct types of oxygen ions residing in chemically different environments.^187^ The B_2_O_6_ sublattice includes six oxygen atoms occupying 48f positions, which form a network of corner-sharing BO_6_ octahedra. Meanwhile, a single 8b-positioned oxygen atom in the A_2_O′ sublattice is part of a framework resembling corner-sharing A_4_O′ tetrahedra. Detailed structural analysis has shown that each 48f oxygen ion is linked to two A-site and two B-site cations, whereas the 8b oxygen is coordinated exclusively to four A-site cations.^188^ Compared to the ideal fluorite lattice, the absence of an oxygen atom at the 8a site introduces intrinsic vacancies, giving rise to the characteristic formula A_2_B_2_O_7_.^189^

The crystallographic phase adopted by a given A_2_B_2_O_7_ compound is largely governed by the ionic radius ratio (r_A_/r_B_):^190–197^

#### 2.7.1 Monoclinic Layered Perovskite Phase (r_A_/r_B_ > 1.78)

When this ratio exceeds 1.78, the material tends to crystallize in a monoclinic-layered perovskite structure, associated with the P2_1_ space group. This type of structure corresponds to a member of the A_n_B_n_O_3n+2_ (n = 4) homologous series and is made up of layers of four corner-connected, distorted BO_6_ octahedra. As depicted in Figure 15a, a cleavage along the *c*-axis reveals two distinct A-site cation environments: one located beneath the octahedral layer and the other situated between two such layers in the interstitial region.

**Figure 15:**
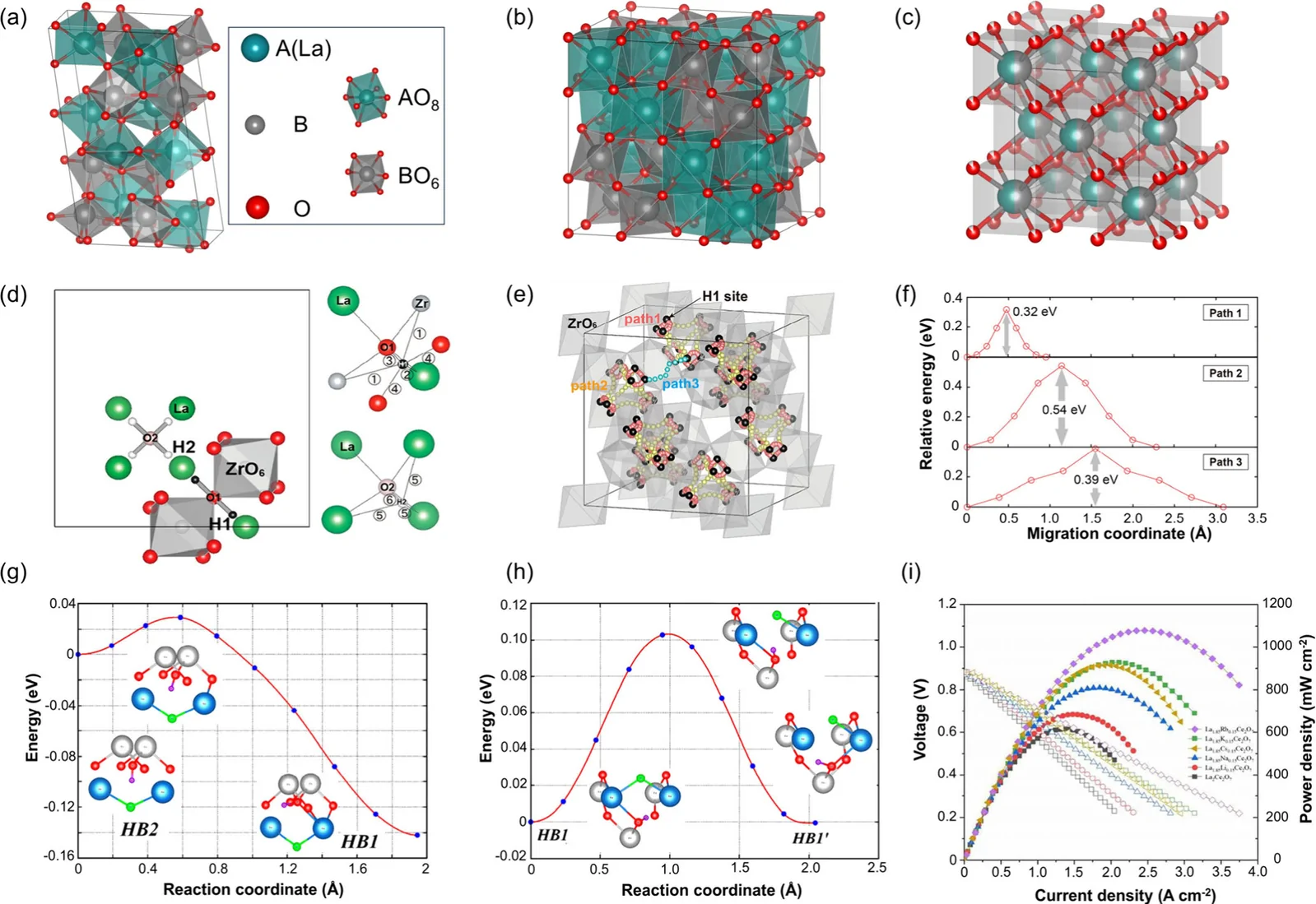
Schematics of the crystalline phase structure of A_2_B_2_O_7−δ_: (a) monoclinic layered perovskite phase, (b) ordered pyrochlore phase, and (c) disordered defective fluorite phase. (d) Proton sites around O1 and O2 ions in La_2_Zr_2_O_7−δ_. (e) Three migration paths found by the nudged elastic band (NEB) method in La_2_Zr_2_O_7−δ_ and (f) their energy profiles. The red, yellow, and light blue spheres denote paths 1, 2, and 3, respectively. Reproduced from ref 199. Copyright 2015 American Chemical Society. Potential energy profiles for proton transfer reaction pathways in La_2_Ce_2_O_7−δ_: (g) HB1-HB2, (h) HB1-HB1′. Reproduced from ref 200. Copyright 2013 American Chemical Society. (i) I–V and I–P curves of the single cells with the La_2_Ce_2_O_7−δ_ and alkaline metal-doped La_2_Ce_2_O_7−δ_ electrolyte measured at 700 °C in a wet H_2_. Reproduced with permission from ref 45. Copyright 2021 Elsevier.

#### 2.7.2 Cubic Ordered Pyrochlore Phase (1.46 ≤ r_A_/r_B_ ≤ 1.78)

A radius ratio within this intermediate range favors the formation of an ordered cubic pyrochlore phase (Figure 15b), classified under the *Fd*3̅*m* space group. This structure can be understood as a partially vacant derivative of the fluorite lattice, where one-eighth of the oxygen positions remain unoccupied. The general formula is often rewritten as A_2_B_2_O_6_O′, with A and B cations positioned at 16d (1/2, 1/2, 1/2) and 16c (0, 0, 0) Wyckoff sites, respectively. The oxygen anions occupy 48f (x, 1/8, 1/8) and 8b (3/8, 3/8, 3/8) sites, while the 8a (1/8, 1/8, 1/8) sites remain vacant, contributing to enhanced lattice oxygen mobility. To preserve electrical neutrality and maintain the ordered structure, the unit cell expands to twice the length of the standard fluorite. The structure is composed of four interrelated sublattices:

1) AO_6_O_2_′ sublattice: Each A-site ion is surrounded by a distorted cube of six O (48f) atoms and two O′ (8b) atoms, with A–O′ bonds being slightly shorter than A–O bonds.
2) BO_6_ sublattice: The B-site ion is located in a distorted octahedral environment coordinated by six oxygen atoms and flanked by two vacant 8a sites.
3) OA_2_B_2_ sublattice: Each oxygen ion in this group bridges two A-site cations and two B-site cations.
4) O′A_4_ sublattice: Each O′ anion is coordinated by four A-site cations. Overall, the structure may also be viewed as a three-dimensional framework of corner-sharing BO_6_ octahedra with A-site ions filling the intervening voids.

#### 2.7.3 Disordered Defect Fluorite Phase (r_A_/r_B_ < 1.46)

For radius ratios below 1.46, a transition occurs to a disordered defect fluorite structure described by the *Fm*3̅*m* space group, as illustrated in Figure 15c. In this configuration, both the A-site and B-site cations are randomly distributed and the oxygen vacancies are also randomly dispersed throughout the lattice. Structural studies indicate that, in such a disordered arrangement, the average coordination number for both A and B cations is approximately 7. Nevertheless, the A-site cations often retain a slightly higher coordination number, suggesting that B-site cations are more commonly situated near intrinsic 8a oxygen vacancies. Because of the simultaneous disorder in both the cation and anion sublattices, this structure offers superior oxygen ion mobility when compared to the ordered pyrochlore phase.

Although the ionic-radius ratio between the A-site and B-site cations is widely used as an empirical guideline for predicting whether an A_2_B_2_O_7_ compound adopts a pyrochlore or defect-fluorite structure, this descriptor ultimately reflects deeper thermodynamic and structural constraints. The stability of the pyrochlore phase arises from its ability to sustain long-range ordering of both cations and oxygen vacancies while maintaining well-defined coordination environments—typically 8-fold coordination for the A-site cations and 6-fold coordination for the B-site cations—with near-ideal A-O and B–O bond lengths and minimal polyhedral distortion.^198^ This ordered arrangement minimizes local bond-strain energy and electrostatic repulsion by preserving distinct cation sublattices and a regular pattern of oxygen vacancies. However, as the size mismatch between A- and B-site cations increases, maintaining these ordered coordination environments becomes increasingly energetically unfavorable. Large size disparities introduce elastic strain, distort BO_6_ and AO_8_ polyhedra, and increase the energetic penalty associated with enforcing long-range vacancy ordering.^190,196^ Under such conditions, the system can lower its Gibbs free energy by adopting the defect-fluorite structure, in which cations and oxygen vacancies are statistically disordered. This disorder relaxes local strain by allowing flexible coordination environments and is further stabilized by a significant gain in configurational entropy, which becomes increasingly important at elevated temperatures. Consequently, the transition from pyrochlore to defect-fluorite should be understood as the outcome of a competition between enthalpic stabilization from ordered bonding and coordination preferences, and the combined elastic- and entropy-driven stabilization associated with structural disorder, rather than as a direct consequence of ionic size ratio alone.

In 1995, Huang et al.^201^ first demonstrated that the pyrochlore-structured oxide Y_3+x_Ta_1–x_O_7−δ_ possesses proton conductivity, primarily facilitated by its disordered oxygen vacancies. This material exhibited dominant proton transport behavior below 400 °C, achieving a conductivity on the order of 10^–7^ S cm^–1^. A year later, in 1996, Shimura et al.^202^ confirmed the occurrence of protonic conduction in a range of pyrochlore-type compounds with the general formula Ln_2_Zr_1.8_Y_0.2_O_7−δ_ (where Ln = La, Nd, Sm, Gd, Er), under hydrogen-rich environments at high temperatures. However, in humid conditions, these materials showed a decline in proton transport number with rising temperature, mainly due to the enhanced electronic conduction at elevated temperatures. In 2001, Omata et al.^203^ investigated how calcium incorporation affects the proton conductivity of La_2_Zr_2_O_7−δ_. Their findings showed that doping with Ca^2+^ significantly boosted the conductivity: at 873 K in a humidified hydrogen atmosphere, the conductivity of (La_1.95_Ca_0.05_)­Zr_2_O_7−δ_ and La_2_(Zr_1.985_Ca_0.015_)­O_7−δ_ reached 6.8 × 10^–2^ S m^–1^ and 1.0 × 10^–2^ S m^–1^, respectively. A positive correlation was noted between the Ca^2+^ content and proton transport performance. In 2005, Liu et al.^204^ reported that La_1.95_Ca_0.05_Ce_2_O_7−δ_ (LCCO), a Ce-based pyrochlore, also displayed protonic conduction in hydrogen-containing atmospheres at elevated temperatures, and outperformed the analogous Zr-based material, La_1.95_Ca_0.05_Zr_2_O_7−δ_, in terms of conductivity. This prompted further studies on La_2_Ce_2_O_7−δ_, revealing that its total conductivity increases significantly with water vapor partial pressure, suggesting a strong protonic transport contribution under moist conditions—especially prominent at lower temperatures. Notably, in wet air (3% H_2_O) at 550 °C, La_2_Ce_2_O_7−δ_ reached a protonic conductivity of 6.68 × 10^–5^ S cm^–1^.

In the pyrochlore La_2_Zr_2_O_7−δ_, two crystallographically inequivalent oxygen sites are present: O1 (48f) and O2 (8b). As shown in Figure 15d, the O1 site is coordinated by two Zr ions and two La ions, whereas the O2 site is surrounded exclusively by four La ions. Correspondingly, two proton sites are identified: H1 and H2, associated with O1 and O2, respectively. First-principles calculations reveal that the H2 site exhibits higher site energy compared to the H1 site, indicating that protons preferentially bind to O1 rather than the more isolated O2 sites. This suggests that the proton-conducting network in La_2_Zr_2_O_7−δ_ primarily follows the three-dimensional ZrO_6_ octahedral framework.^199,205^

Proton migration in the bulk involves numerous sequential proton transfer events, including hopping between adjacent oxygen atoms and rotational reorientation around host oxygen. As illustrated in Figure 15e,f, three representative migration pathways (Paths 1–3) are considered:^199,206^

- Path 1 involves short-range hopping (0.95 Å) along the edges of the equilateral triangle formed by three adjacent H1 sites, with the lowest energy barrier of 0.32 eV.
- Path 2 corresponds to partial rotation around an O1 ion, with a longer migration distance (2.28 Å) and a higher energy barrier of 0.54 eV.
- Path 3, the rate-determining step, bridges otherwise isolated migration domains by enabling internetwork transport. It features an intermediate hopping distance of 3.09 Å and an energy barrier of 0.39 eV.

In the pyrochlore La_2_Ce_2_O_7−δ_, four energetically favorable proton sites have been identified (Figure 15g,h): two near O8b (HA1 and HA2) and two adjacent to O48f (HB1 and HB2). Among these, the HB sites (associated with O48f) are energetically more stable than the HA sites (near O8b). Geometrically, the HA1, HB1, and HB2 sites are located nearly along the lines connecting adjacent oxygen atoms (O8b–O48f, O48f–O48f, and O48f–O8b, respectively), while the HA2 site lies approximately on the plane bisecting the O48f–O8b–O48f angle. Five representative migration paths are considered in La_2_Ce_2_O_7−δ_, including three rotational (HA1–HA2, HB1–HB1′, HB1–HB2) and two jumping processes (HA1–HB2, HB1–HB1′). Among them, the HA1–HA2 and HB1–HB1′ paths exhibit the lowest energy barriers, suggesting they dominate the overall proton transport mechanism in this system.^200,206^

Thanks to these favorable characteristics, La_2_Ce_2_O_7−δ_ has attracted considerable attention as a proton-conducting electrolyte for RSOCs. Its chemical stability in H_2_O and CO_2_-rich environments, coupled with its moderate proton conductivity, makes it a strong candidate for energy conversion applications. Moreover, doping of A-site elements, including rare earth elements, alkaline earth metals, and alkali metals, has proven to be a straightforward yet effective strategy to further enhance key material properties like proton transport capability, sintering behavior, and chemical robustness.

#### 2.7.4 Rare Earth Elements Doped La_2_Ce_2_O_7−δ_

Given the comparable electronic configurations across rare-earth elements, substituting various rare-earth elements for La has been extensively explored to modify material performance.

Anwar et al.^207^ synthesized a series of La_2–x_Sm_x_Ce_2_O_7−δ_ (x = 0–0.20) powders to investigate how Sm substitution at the A-site influences phase composition and electrochemical behavior. All samples maintained a disordered cubic fluorite structure. Notably, the undoped material (x = 0) exhibited a biphasic structure containing both fluorite (F-type) and pyrochlore (C-type) phases, with the C-type fraction progressively declining as Sm content increased. The La_1.95_Sm_0.05_Ce_2_O_7−δ_ exhibited the highest ionic conductivity, reaching 3.7 × 10^–3^ S cm^–1^ at 700 °C under humidified conditions. Correspondingly, fuel cells based on this composition delivered peak power densities of approximately 474 mW cm^–2^ at 700 °C and 397 mW cm^–2^ at 650 °C.

Subsequently, the team extended their approach to Yb-doped La_2_Ce_2_O_7−δ_, preparing La_2–x_Yb_x_Ce_2_O_7−δ_ with x values ranging from 0 to 0.20. These materials also exhibited coexisting F-type and C-type phases, depending on dopant concentration. Among them, La_1.95_Yb_0.05_Zr_2_O_7−δ_ demonstrated the highest ionic conductivity, measuring 3.3 × 10^–3^ S cm^–1^ at 700 °C. Fuel cells employing this composition achieved a maximum power output of 589 mW·cm^–2^ in wet hydrogen and 420 mW·cm^–2^ when operated with methane at the same temperature.^208^

In another study, Han et al.^209^ examined the effects of doping rare earth ions, including Y, Ho, Er, Yb, and Tm, on both the proton concentration and conductivity of La_2_Ce_2_O_7−δ_. All doped variants preserved a single-phase fluorite structure up to a doping level of x = 0.2. Among the dopants tested, Y^3+^ yielded the highest proton content, with La_2_(Ce_0.95_Y_0.05_)_2_O_7−δ_ achieving the peak conductivity at 600 °C. This behavior was attributed to a balance between enhanced hydration ability and reduced proton mobility at elevated Y levels. However, Choudhary et al.^210^ reported that a higher Y content of x = 0.15 in the A site of La_1.85_Y_0.15_Ce_2_O_7−δ_ produced superior ionic conductivity of 3.29 × 10^–3^ S cm^–1^ in a humid atmosphere at 700 °C. The corresponding cell demonstrated maximum power densities of approximately 500, 353.6, and 197 mW cm^–2^ at 700 °C, 650 °C, and 600 °C, respectively. The discrepancies between these two reports may arise from variations in synthesis protocols or microstructural differences, which influence hydration and defect chemistry.

#### 2.7.5 Alkaline Earth Metals Doped La_2_Ce_2_O_7−δ_

The incorporation of divalent alkaline-earth cations (A^2+^ = Ca, Sr, Ba) in place of La^3+^ in La_2_Ce_2_O_7−δ_ has been investigated for its influence on hydration characteristics and ionic conductivity. This substitution enhances hydration behavior, likely due to a greater concentration of oxygen vacancies that can incorporate protons and to the altered local coordination environment introduced by A^2+^ ions alongside La^3+^.

Liu et al.^211^ reported that doping with Mg notably improves the sinterability of La_2_Ce_2_O_7−δ_, enabling the successful fabrication of La_1.85_Mg_0.15_Ce_2_O_7−δ_ (LMCO) dense ceramic membranes via cofiring at a relatively low temperature of 1300 °C. LMCO exhibited superior electrical performance, reaching 7.41 × 10^–3^ S cm^–1^ in dry air and 1.55 × 10^–2^ S cm^–1^ in humidified hydrogen (∼3% H_2_O) at 700 °C. The electrolyte also showed remarkable resistance to degradation in the presence of CO_2_ and H_2_O. In electrochemical performance tests, LMCO-based fuel cells achieved a maximum power output of 897 mW cm^–2^ at 700 °C, indicating their potential as a proton-conducting electrolyte for chemically robust, high-performance SOFCs with lower sintering requirements.

In another study, Tao et al.^212^ demonstrated that La_1.95_Ca_0.05_Ce_2_O_7−δ_ remains chemically stable after exposure to 3% CO_2_ and 3% H_2_O at 700 °C for 100 h, confirming its promise as an SOFC electrolyte. A single cell was constructed using LCCO as the electrolyte and a cathode comprising a blend of LSCF and LCCO via a wet suspension method. The assembled cell yielded a PPD of 259 mW cm^–2^ at 700 °C, with a total resistance of 0.44 Ω cm^2^ and an electrode polarization resistance of 0.23 Ω cm^2^. The OCV reached 0.832 V at 700 °C. These characteristics, coupled with the possibility of improving the OCV using a bilayer electrolyte design, underscore LCCO’s viability as a candidate for commercial SOFC applications.

#### 2.7.6 Alkali Metals Doped La_2_Ce_2_O_7−δ_

Due to their comparable ionic radii to La^3+^ and their potential to enhance hydration, alkali metals (Li, Na, K, Rb, Cs) have been investigated as dopants in La_2_Ce_2_O_7−δ_.^213^

Peng et al.^214^ explored the influence of sodium incorporation at the A-site, synthesizing a series of La_2–x_Na_x_Ce_2_O_7−δ_ (x = 0–0.20) compounds. Na-doping not only improved total conductivity but also enhanced sintering characteristics. The composition La_1.85_Na_0.15_Ce_2_O_7−δ_ achieved the highest conductivity of 1.66 × 10^–2^ S cm^–1^ in dry air at 800 °C and 1.38 × 10^–2^ S cm^–1^ in a 5% H_2_–95% Ar environment at 700 °C, performance comparable to BaCeO_3−δ_-based electrolytes. Moreover, this material displayed excellent chemical stability under harsh operating conditions. A single cell based on La_1.85_Na_0.15_Ce_2_O_7−δ_ delivered a maximum power density of 501 mW cm^–2^ and an OCV of 0.933 V at 700 °C.

In a broader study, Yang et al.^213^ discovered that replacing part of the La^3+^ with alkaline metal ions not only generated more oxygen vacancies for ion transport but also improved the sinterability of La_2_Ce_2_O_7−δ_. Liu et al.^45^ systematically investigated the impact of various alkali dopants (Li, Na, K, Rb, Cs) on the electrical properties of La_2_Ce_2_O_7−δ_. The conductivity increased with the dopant’s ionic radius up to Rb, but decreased when Cs was used. La_1.85_Rb_0.15_Ce_2_O_7−δ_ showed excellent conductivity of 3 × 10^–3^ S cm^–1^ in dry air and 6.21 × 10^–3^ S cm^–1^ in humidified H_2_ at 800 °C. Impressively, single cells employing this material achieved peak power densities of 1031 mW cm^–2^ at 700 °C (Figure 15i), maintaining stable operation for over 200 h. These results identify Rb-doped La_2_Ce_2_O_7−δ_ as a highly promising electrolyte for next-generation SOFCs.

#### 2.7.7 Other Doped La_2_Ce_2_O_7−δ_

In addition to the aforementioned dopants, transition metal molybdenum (Mo) was introduced into La_2_Ce_2_O_7−δ_ by Peng et al.^215^ to further enhance protonic conductivity. XRD analysis confirmed that all La_2–x_Mo_x_Ce_2_O_7−δ_ samples maintained a stable cubic fluorite structure. The ionic conductivity of Mo-doped La_2_Ce_2_O_7−δ_ electrolytes was investigated under both air and 5% H_2_–95% Ar conditions. Among the compositions studied, La_1.85_Mo_0.15_Ce_2_O_7−δ_ exhibited the highest conductivity, reaching 7.36 × 10^–3^ S cm^–1^ in air and 1.65 × 10^–2^ S cm^–1^ in wet hydrogen atmosphere at 700 °C. Moreover, single cells employing this electrolyte achieved a PPD of approximately 0.643 W cm^–2^ at 700 °C, indicating the potential of Mo doping in significantly boosting the electrochemical performance of IT-SOFCs.

In addition, the beneficial effect of indium (In) doping was also demonstrated.^216^ The La_1.9_In_0.1_Ce_2_O_7−δ_ sample delivered a maximum total conductivity of 0.82 × 10^–2^ S cm^–1^ in dry air and 2.03 × 10^–2^ S cm^–1^ in wet 5% H_2_–Ar atmosphere at 700 °C. A fuel cell constructed with a Ni–BaCe_0.5_Zr_0.3_Dy_0.2_O_3−δ_ anode and the La_1.9_In_0.1_Ce_2_O_7−δ_ electrolyte exhibited a significantly improved open circuit voltage of 1.005 V and a maximum power density of 546 mW cm^–2^ at 700 °C. These results confirm the synergistic role of In doping in promoting both protonic transport and overall fuel cell performance.

### 2.8 Other Electrolytes

The preceding sections have primarily focused on the development of proton-conducting electrolytes based on the specific crystal structures. Over the past three decades, advances in nanomaterials and semiconductor technologies have enabled the emergence of ceria-carbonate composite electrolytes and semiconductor-based oxide electrolytes, which exhibit excellent conductivity at intermediate to low temperatures.^217,218^ Notably, these two classes of electrolytes often function as dual-ion conductors, transporting both protons and oxygen ions. Because oxygen-ion conduction faces a higher activation energy barrier than proton conduction at low temperatures, most studies have emphasized enhancing proton transport in these materials within the intermediate-to-low-temperature range. This research direction has provided new insights into the design of proton-conducting electrolytes and the protonation-based modification of mixed ionic conductor systems.

#### 2.8.1 Ceria-Carbonate Composite

As typical oxygen ion conductors, doped ceria such as samarium-doped ceria (SDC) and GDC are commonly used as electrolytes in high-temperature SOFCs (>700 °C).^219–221^ However, the performance of doped ceria below 600 °C is limited by low ionic conductivity and significant electronic conduction in reducing environments.^218,222^ In 2003, Zhu investigated composites of doped ceria with various salts, including chlorides, hydroxides, and carbonates, to enhance the ionic conductivity and chemical stability.^223^ The SDC–(Li/Na)_2_CO_3_ composites show optimal performance, with conductivity boosted by amorphous carbonate layers that create continuous ion transport pathways.^223^ Initially, Zhu identified that oxygen ion conduction in the ceria phase is governed by oxygen vacancies, while proton conduction occurs in the carbonate through a temporary bonding mechanism,^224^ specifically the H^+^–CO_3_^2–^ pathway. Subsequent studies revealed that interfacial ion conduction plays a key role in enabling hybrid H^+^ and O^2–^ conduction, thereby demonstrating a distinct composite effect.^225^ Since the first report of ceria-carbonate composite electrolytes, research on these materials has proliferated, driven by their unique dual-conduction properties.^218,223,226–228^ This kind of composite has been developed by integrating an oxygen-ion conducting framework with alkali carbonates, creating a new class of hybrid proton and oxygen ion conductive materials with transformative properties for low-temperature solid oxide fuel cells (LT-SOFCs).

The typical microstructure of ceria-carbonate composite exhibits a well-defined core–shell architecture in which doped ceria oxides (e.g., SDC, GDC) form the core, and the surrounding shell is primarily composed of Li_2_CO_3_, Na_2_CO_3_, and K_2_CO_3_ (Figure 16a).^229^ Compared to doped ceria, ceria-carbonate composites exhibit enhanced ionic conductivity at low temperatures. This enhancement is attributed to their nanostructured core–shell architecture, where amorphous carbonate shells coat ceria-based nanoparticles, creating extensive interfacial areas (Figure 16b) that act as “superionic highways” for ion transport.^226^ The SDC–Na_2_CO_3_ exemplifies this behavior, reaching an ionic conductivity of 0.1 S cm^–1^ at approximately 300 °C, a nearly two-order-of-magnitude increase compared to that of single-phase SDC (Figure 16c).^226,230^ An empirical “Swing Model” was proposed by Wang et al.^226^ to explain the proton conduction mechanism. As shown in Figure 16d, when protons from the anode reach the composite electrolyte, they form metastable hydrogen bonds with oxygen ions on the surface of SDC and the surrounding carbonate groups. At temperatures above the glass transition temperature of the amorphous carbonate phase, bending/stretching vibrations of C–O bonds and mobility/rotation of CO_3_^2–^ groups become pronounced. The interface between SDC and carbonate serves as a highly conductive pathway, facilitating dynamic breaking and reforming of hydrogen bonds with carbonate ions acting as a “bridge” to enable efficient long-range proton transport.^226^ Based on this mechanism, a novel core–shell SDC–Na_2_CO_3_ nanocomposite demonstrated ion conductivities exceeding 0.1 S cm^–1^ between 300 and 600 °C-orders of magnitude higher than individual SDC. It also achieved an excellent PPD of 0.8 W cm^–2^ at 550 °C.^231^

**Figure 16:**
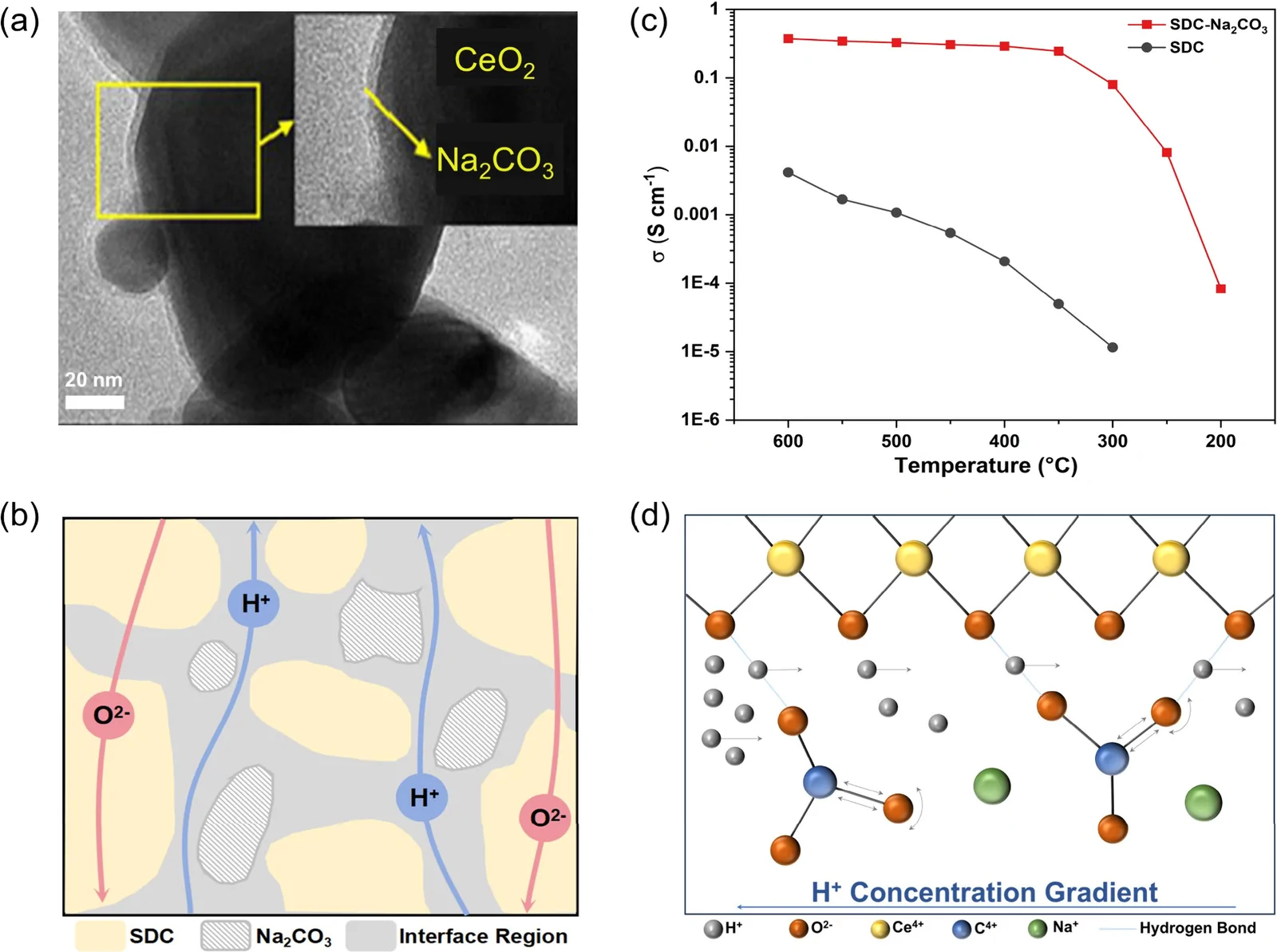
(a) High-resolution transmission electron microscopy image of CeO_2_–Na_2_CO_3_ with a zoomed-in view of the CeO_2_ particle coated with Na_2_CO_3_ shell. Reproduced with permission from ref 229. Copyright 2021 Elsevier. (b) A schematic illustration of dual H^+^/O^2–^ conduction pathways: the protons are transported by the interface in the SDC–Na_2_CO_3_ nanocomposite, while oxygen ion conduction is through the grains of SDC. (c) Conductivity of SDC–Na_2_CO_3_ nanocomposite^226^ compared to that of pure SDC^230^ at various temperatures. (d) A schematic illustration of the “Swing model” pathway for proton conduction in SDC–Na_2_CO_3_ nanocomposite. Reproduced with permission from ref 226, 230 . Copyright 2011 and 2009 Elsevier.

The composition and content of the carbonate phase strongly affect the structural and electrochemical properties of ceria-carbonate composites. Huang et al.^225^ studied SDC–binary carbonate (Li_2_CO_3_–Na_2_CO_3_) composite electrolytes and found that the carbonate content affects the superionic phase transition behavior and the underlying conduction mechanism. They assumed that both oxygen ion and proton conduction are significant at low carbonate content, while proton conduction dominates at high carbonate content. The composite electrolytes achieved a total ionic conductivity of 0.1 S cm^–1^ in air at 500 °C (with 20 wt % carbonate). Ali et al.^232^ systematically investigated the synthesis and electrochemical performance of SDC composite electrolytes incorporated with single, binary, and ternary alkali carbonates (Li_2_CO_3_, Na_2_CO_3_, K_2_CO_3_). Among the composites, the binary Li/Na carbonate–SDC exhibits the highest ionic conductivity of 0.31 S cm^–1^ in a H_2_ atmosphere at 600 °C, attributed to its low eutectic melting point and formation of a continuous amorphous carbonate layer that facilitates fast ion transport.^232^ Moreover, the morphology of ceria also decides the thermal and ionic transport behavior of ceria-carbonate electrolytes.^233^ A composite electrolyte comprising rod-like SDC particles—synthesized via a coprecipitation method—and a (Li/Na)_2_CO_3_ eutectic salt favored the formation of consecutive interface and dual-phase pathways for the ionic conduction. The corresponding single cell exhibited an excellent performance with a PPD of 1700 mW cm^–2^ at 650 °C.^233^ Furthermore, a single chamber reaction for electrochemical ammonia synthesis using a GDC–(Li, Na, K)_2_CO_3_ composite electrolyte has been demonstrated, achieving an ammonia formation rate of 1.83 × 10^–6^ mol s^–1^ m^–2^ at 1.4 V and 400 °C.^234^ This further demonstrates the significant proton conduction in the ceria-carbonates composite electrolyte, which holds promise for realizing more functional electrochemical applications.

#### 2.8.2 Semiconductor-Based Oxides

In the pursuit of novel proton-conducting materials, extensive efforts have adopted semiconductor heterostructure composites as a viable strategy to develop superionic conductors.^235–237^ In 2011, Zhu reported an electrolyte-free fuel cell constructed from a single homogeneous layer composed of a mixed-conductivity composite, combining LiNiZn-based oxide and SDC, which revealed high mixed conductivity of 1 S cm^–1^ above 500 °C.^238^ In the same year, they proposed double-layer fuel cell (DLFC) devices that eliminate the need for a physically separated electrolyte layer.^239^ These devices are commonly referred to as semiconductor-based fuel cells (SBFCs) based on the operational phenomenon they exhibit. SBFCs represent a novel approach to LT-SOFCs, leveraging semiconductor heterostructures and interface engineering to overcome the limitations of traditional electrolyte-based systems. Unlike conventional SOFCs, which rely on pure ionic conductors and operate at high temperatures (800–1000 °C), SBFCs utilize semiconductor membranes (SM) or semiconductor-ionic membranes (SIM) materials to enable efficient proton/oxygen ion conduction and electron blocking at 300–600 °C.^235^ Their functionality hinges on energy band alignment and a built-in electric field (BIEF) formed at heterojunctions, which facilitates ionic transport while suppressing electronic short-circuiting.^235,236^

A heterojunction refers to an interaction region formed by the contact of two materials (e.g., a metal and a semiconductor, or a semiconductor and another semiconductor) with different major charge carriers. This principle is exemplified by the prototypical p–n junction, as illustrated in Figure 17.^236^ When n-type and p-type semiconductors come into contact, electrons diffuse from the conduction band of the n-type semiconductor to the p-type, while holes migrate from the valence band of the p-type semiconductor to the n-type due to the concentration gradient. This diffusion continues until the Fermi levels of the two semiconductors align, resulting in energy band bending that progresses from the p-type to the n-type regions. Consequently, a BIEF barrier is established, which hinders further electron movement from the n-type to the p-type layer and hole migration from the p-type to the n-type layer. This interaction region, formed by the concentration diffusion of majority carriers and the drift motion of minority carriers, is also termed the space charge region, where the BIEF exists.^236,240^ The synergistic effect of the BIEF and the ion concentration potential within the heterojunction enables SBFCs to have a specific proton and oxygen ion transport mode, thereby effectively promoting the hydrogen oxidation reaction and the oxygen reduction reaction processes.

**Figure 17:**
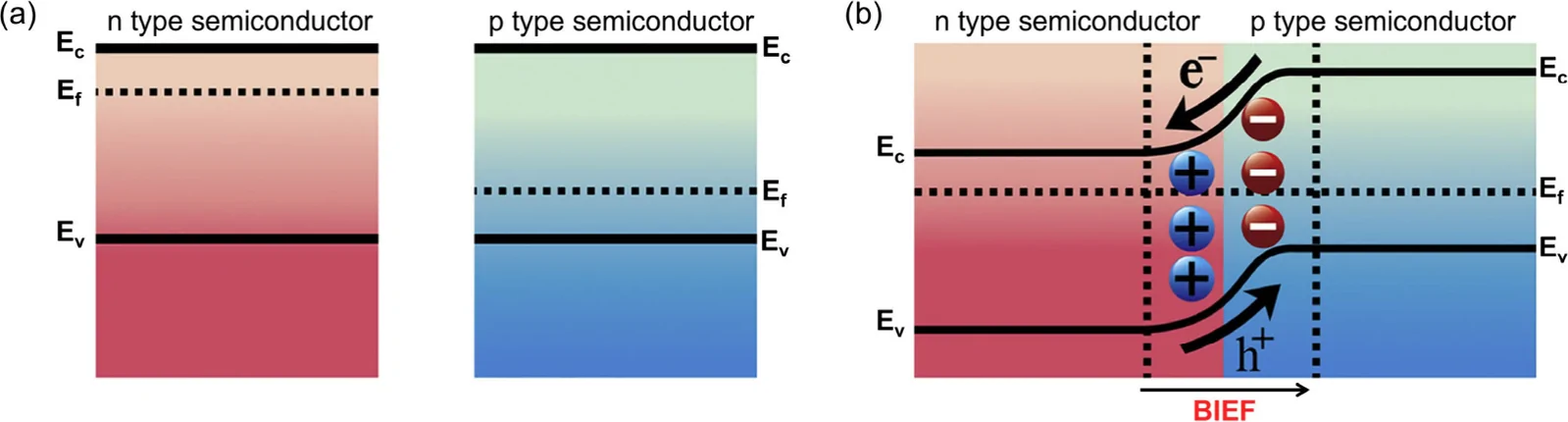
Schematic of (a) energy band of n- and p-type semiconductor before contact and (b) energy band bending when p-n heterojunction is formed after contact; E_v_, the energy of maximum valence band; E_f_, Fermi energy; E_c_, the energy of minimum conduction band. Reproduced with permission from ref 236. Copyright 2021 Elsevier.

##### 2.8.2.1 SM Material

In traditional SOFCs, a partial leakage current in the ionic conductor electrolyte can lead to a notable reduction in fuel cell efficiency and open-circuit voltage.^166,241,242^ To address this issue, semiconductor oxide materials have been successfully applied to replace conventional ionic conductor electrolytes. This new device integrates the functions of the cathode, anode, and electrolyte in a fuel cell into a single-layer membrane composed of semiconductor oxides or semiconductor-ionic heterostructured composites, which has been given the name of single-layer fuel cells (SLFCs).^236^ On this basis, many semiconductor membrane materials have been prepared and exhibited remarkable electron, proton, and oxide ion conductivity.^48,243–245^

Shah et al.^243^ synthesized SrFe_x_Ti_1–x_O_3−δ_ p-type semiconductor and applied it as an electrolyte with symmetrical Ni_0.8_Co_0.15_Al_0.05_LiO_2_ (NCAL) electrodes. Ultraphotoelectron spectroscopy revealed conduction band energy levels of −4.66 eV for SrFe_x_Ti_1–x_O_3−δ_ and −5.27 eV for NCAL, indicating a conduction band offset and the formation of a p–n junction at the interface. The resulting band bending facilitates directional ionic transport while effectively suppressing cross-layer electron conduction, thereby improving the charge separation and overall ionic conductivity. The activation energy for ionic charge carrier transport in SrFe_0.3_Ti_0.7_O_3−δ_ is 0.65 eV, and the total conductivity of the electrolyte reaches 0.17 S cm^–1^ in a Ni–NCAL|SrFe_0.3_Ti_0.7_O_3−δ_|Ni–NCAL configuration fuel cell with H_2_/O_2_ atmosphere at 520 °C.^243^

In perovskite semiconductor oxides, protons can diffuse through a Grotthuss mechanism involving the fast rotational diffusion of protonic defects and the rate-limiting proton transfer to neighboring oxygen ions, while forming ionic defects by bonding with oxygen.^52,57,246^ Zhou et al.^48^ studied the use of perovskite semiconductor oxide membrane SmNiO_3_ (SNO) with high electronic conductivity as an electrolyte to build the PCFC and found that hydrogen-induced filling-controlled Mott transition can turn SNO into an electrically insulating H-SNO on the anode side, which suppresses electronic conduction. As shown in Figure 18a,b, protons are spontaneously incorporated into the lattice, where they undergo rotational diffusion within an octahedron and transfer to a neighboring oxygen by hydrogen bonding.^48^ In the microfabricated PCFC employing the SNO electrolyte, the OCV increases under continuous hydrogen flow until the H-SNO phase transition reaches a steady state. The maximum OCV attains 1.03 V at 500 °C, which is very close to the Nernst potential. Based on estimates using the standard EMF method, the t_i_ of H-SNO is 0.96.^48^ A similar transformation also occurred in the brownmillerite oxide SrCoO_2.5_ (SCO).^49^ It can be realized through ionic liquid gating (ILG) and noble metal Pt catalysis. For the latter one, during the annealing process in a 10% H_2_–Ar atmosphere at temperatures below 150 °C, protons formed by Pt-catalyzed conversion on the SCO surface intercalated into the SCO lattice, forming the H-SCO phase (Figure 18c).^49^ Figure 18e shows the total energies of optimized crystalline configurations calculated along a series of snapshots during the proton transportation process. First-principles calculations and proton diffusion coefficient tests show that the ordered oxygen vacancy channels, elevated proton concentration, and significantly suppressed electron conductivity within H-SCO collectively enable low-temperature proton conduction, with superior proton conductivities of 0.028 S cm^–1^ and 0.33 S cm^–1^ at 40 and 140 °C, respectively. The electronic conductivity is 2 orders of magnitude lower than the intrinsic proton conductivity, as determined from electrochemical impedance spectroscopy (EIS) and DC polarization measurements, confirming that protons are the dominant charge carriers in H-SCO. Correspondingly, a Pt|H-SCO|Pt fuel cell delivers a PPD of 170 mW cm^–2^ at an ultralow operating temperature of 120 °C, demonstrating the feasibility of H-SCO as an electrolyte for low-temperature fuel cells (Figure 18d).^49^

**Figure 18:**
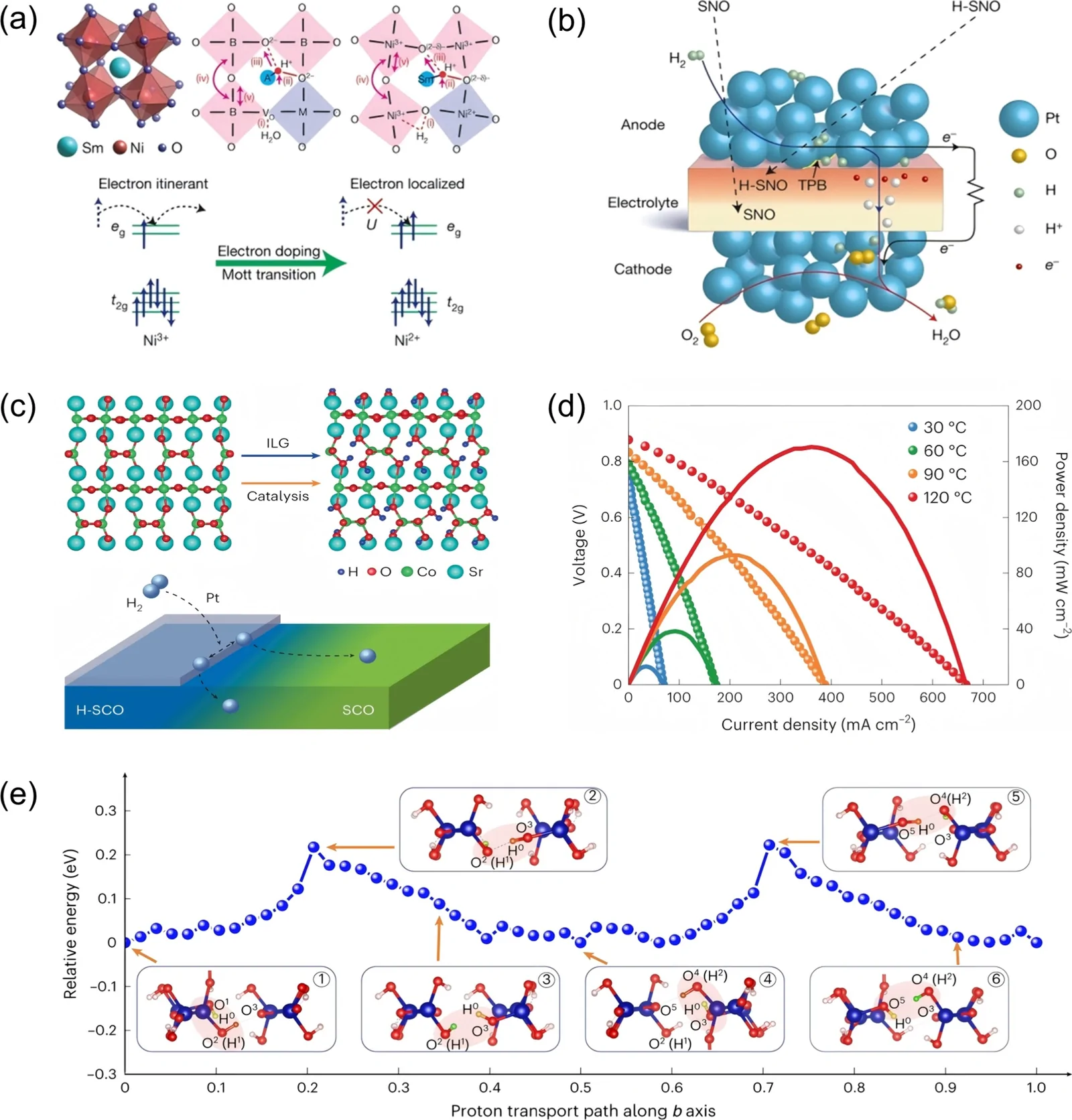
(a) Diagram of a filling-controlled Mott transition from SNO to H-SNO. (b) A schematic of an SNO-electrolyte PCFC and its operation mechanism. Reproduced with permission from ref 48. Copyright 2016 Springer Nature. (c) Structural demonstration of the phase transformations realized by ILG and noble metal Pt catalysis, and the schematic illustration of the latter. (d) Measured current density (dotted lines, *x*-axis and left *y*-axis) and power density (solid lines, right *y*-axis) for the fabricated fuel cell with the electrolyte of H-SCO. (e) Calculated energy profile during proton transportation within the H-SCO. Reproduced with permission from ref 49. Copyright 2022 Springer Nature.

##### 2.8.2.2 SIM Material

In 2011, a planar p-n heterojunction fuel cell device without an electrolyte layer was fabricated by directly pressing the LiNiCuZn­(Fe)-oxide/(Na/K)_2_CO_3_–Ce_0.8_Sm_0.2_O_2−δ_ mixture as the anode and BSCF–SDC as the cathode layer by layer under a pressure of 100–300 MPa.^239^ A BIEF is formed, directed from the anode to the cathode at the applied fuel cell atmosphere, thus suppressing the electronic short-circuiting phenomenon and simultaneously promoting ionic conduction. The DLFC device displayed slightly lower but comparable performances to conventional electrolyte-based fuel cells, achieving a PPD near 600 mW cm^–2^ at 550 °C.^239^ Alternatively, a functional single-component p-n heterojunction layer can be formed by intimately mixing semiconductor and ion-conducting particles at the micro- or nanoscale, creating a bulk heterogeneous structure.^239,247,248^ This semiconductor-ionic membrane (SIM) configuration can also be used to fabricate SLFCs, which further reduce the polarization loss at the interface between the p-type layer and the n-type layer in the DLFCs.^236,248^

H^+^/O^2–^/e^–^ triple-conducting material BaCo_0.4_­Fe_0.4_­Zr_0.1_­Y_0.1_O_3−δ_ (BCFZY) was applied for electrolyte owing to its high ionic conduction, and a bulk p-n heterojunction was developed by composing BCFZY and ZnO, as shown in Figure 19a.^249^ Through band bending alignment for BCFZY–ZnO, this heterostructure can form a BIEF, sweeping electrons into ZnO and holes into BCFZY, effectively suppressing electronic leakage while simultaneously lowering ionic migration barriers across the depletion region. Meanwhile, the built-in electrostatic force also facilitates the migration of charged species H^+^ and O^2–^, accelerating transport and reducing the activation energy to 0.62 eV.^249^ Figure 19b,c shows the electrochemical performance of BCFZY–ZnO electrolyte. The composite exhibited extraordinary total conductivity of 0.11–0.26 S cm^–1^ at 400–500 °C and a comparable fuel cell performance of 643 mW cm^–2^ power density at 500 °C.^249^ However, these conductivity values reflect the mixed ionic conduction behavior of BCFZY–ZnO. To further verify the proton-conducting properties, an O^2–^ blocking fuel cell was fabricated in the configuration: Ni–NCAL|BaCe_0.7_Zr_0.1_Y_0.2_O_3−δ_|BCFZY–ZnO|BaCe_0.7_­Zr_0.1_­Y_0.2_­O_3−δ_|Ni–NCAL. In this setup, the proton conductivity of BCFZY–ZnO contributed roughly half of the total ionic conductivity, reaching appreciable values of 0.039–0.098 S cm^–1^ over the temperature range of 400–500 °C.^249^ Most SIM materials consist of a simple physical mixture of semiconductor and ionic conductor phases. Following this principle, other reported SIM materials such as SrFe_0.75_Ti_0.25_O_3−δ_–SDC and LaFe_0.65_Ti_0.35_O_3−δ_–SCDC also have the same heterostructure and have demonstrated superior ionic conduction properties.^250,251^

**Figure 19:**
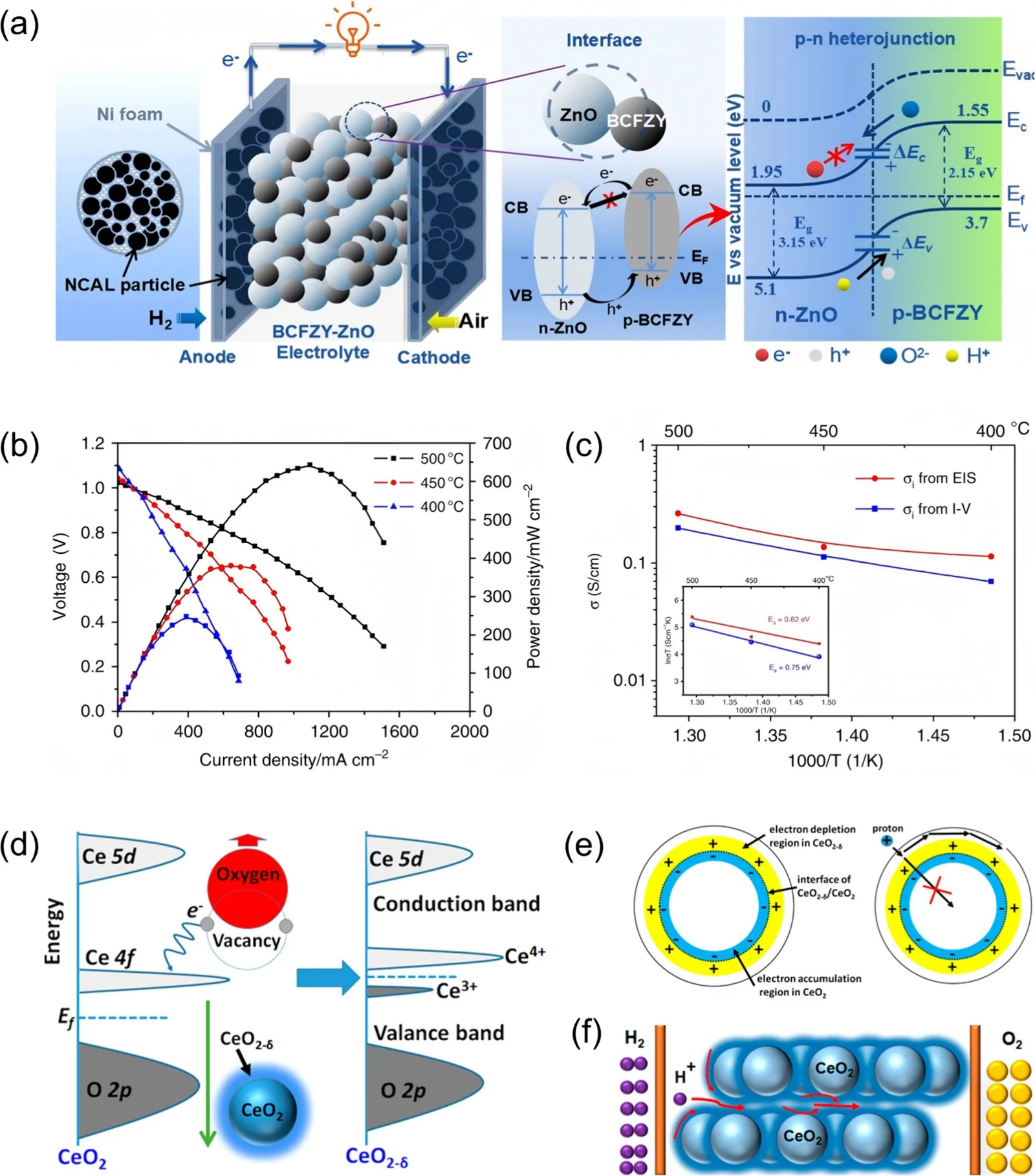
(a) Schematic diagram of a typical p-n heterojunction formed at the heterointerface of the BCFZY–ZnO electrolyte layer and the corresponding energy band alignment mechanism proposed for interpreting the charge separation and ionic transportation process. (b) Electrochemical performance of the BCFZY–ZnO electrolyte-based SOFC at 400–500 °C. (c) Ionic conductivity and activation energy plots of BCFZY–ZnO as a function of 1000/T at 400–500 °C. Reproduced from ref 249. Available under a CC-BY 4.0 license. Copyright 2019 Springer Nature Chen Xia et al. (d) Schematic of electronic structure changes with oxygen vacancy formation in CeO_2−δ_. (e) Diagram of the electron separation at the interface of CeO_2_/CeO_2−δ_ core–shell structure and proton transportation near the surface region of CeO_2−δ_. (f) Diagram of the “proton shuttles” transport in the high-conducting region of the electrolyte membrane constituted by the surface region of the CeO_2−δ_ shell. Reproduced from ref 253. Copyright 2019 American Chemical Society.

Beyond the bulk p-n heterojunction, the Schottky junction (SJ) effect has also been widely reported in the SLFCs. It is generally believed that an SJ is formed at the contact interface between a metal and a semiconductor. Through designing the structure of the semiconductor-based electrolyte, the metal reduced in the hydrogen atmosphere on the anode side of the fuel cell will form an SJ with the semiconductor at the interface, thus effectively suppressing the conduction of electrons in the electrolyte.^235,236^ The innovative research on an SJ-based fuel cell with a configuration of Ni|LiNi_0.85_Co_0.15_O_2−δ_ (LCN)–(Ce_0.8_Sm_0.2_O_2−δ_–Na_2_CO_3_)|NCAL was reported by Zhu et al.^240^ In this configuration, LCN was used for heterostructure as an intrinsic p-type semiconductor with hybrid proton-oxygen ion conducting material Ce_0.8_Sm_0.2_O_2−δ_–Na_2_CO_3_. The SJ was *in situ* formed due to the reduction of Ni and Co from the LCN on the anode side, promoting the ions’ transport and blocking the electron leakage. In this case, the device performance has been significantly enhanced, exhibiting a maximum power density of about 1000 mW cm^–2^ at 550 °C.^240^ Another SIM material, Co_0.2_Zn_0.8_O–SDC, which utilizes the SJ effect to achieve good electron–ionic transport properties, has also been used in designing SLFCs.^252^ By applying different bias voltages, the unique rectification phenomenon of the SJ was observed in a Ni–NCAL|Co_0.2_Zn_0.8_O–SDC asymmetric structure, further demonstrating that the BIEF generated in the SJ serves as a barrier to electron transport through the electrolyte.^252^ Moreover, the proton conductivity of the Co_0.2_Zn_0.8_O–SDC composite electrolyte is significantly improved compared to either Co_0.2_Zn_0.8_O or SDC, achieving 0.01–0.088 S cm^–1^ at 450–550 °C. This heterostructure expands the TPB for reactions and reduces the electrode R_p_ from 0.54 Ω cm^2^ to 0.254 Ω cm^2^, further enhancing the efficiencies of the hydrogen oxidation reaction and oxygen reduction reaction, enabling the fuel cell to achieve a maximum power density of 928 mW cm^–2^ at 550 °C.^252^

The application of ceria-based electrolytes in LT-SOFCs is hindered by electronic leakage and microcrack formation, resulting from the partial reduction of Ce^4+^ to Ce^3+^ under the reducing atmosphere at the anode. In addition to the method discussed in the previous 2.8.1 section for protecting inner ceria-based components by introducing a carbonate shell secondary phase, increasing attention has been directed toward the n-i type heterointerface spontaneously formed on the ceria surface via reduction pretreatment or heat treatment. Xing et al.^253^ further advanced the core–shell concept by engineering CeO_2_/CeO_2−δ_ nanoheterojunctions, where oxygen vacancy-rich CeO_2−δ_ shells encapsulate intrinsic CeO_2_ cores. As depicted in Figure 19d,e, in CeO_2−δ_, electron localization in the unoccupied 4f orbitals of the two neighboring Ce^4+^ atoms accompanies the formation of an oxygen vacancy. CeO_2−δ_ is an intrinsic n-type semiconductor, while CeO_2_ primarily functions as an ionic conductor. Consequently, electron transfer from the CeO_2−δ_ shell to the CeO_2_ core builds an electron depletion region in the shell and an accumulation region in the core.^253^ The positively charged layer in the shell would prevent protons from migrating deep inside the shell to cross the interface. As a result, proton transportation is confined to the surface and shallow layers near the surface region of CeO_2−δ_. This special core–shell architecture effectively reduces electron density in the shell, suppressing electron conduction while enhancing proton transport through a continuous, high-conducting “proton shuttle” pathway (Figure 19f).^253^ The proton isotopic conduction effect was measured to further verify the proton transport properties. The conductivity of CeO_2_ with core–shell heterojunctions in D_2_O increased by more than 3-fold compared to that in air, and it was further enhanced by approximately 2-fold from D_2_O to H_2_O. Thermal stability studies emphasized the crucial role of the amorphous shell, as elevated heat treatments diminished vacancies, raised activation energy, and drastically reduced performance. A fuel cell device incorporating this heterostructure achieved a PPD of 697 mW cm^–2^ at 520 °C and maintained stable operation for 200 h.

Although ceria-carbonate composite electrolytes and semiconductor-based oxide electrolytes have exhibited promising electrochemical performance in intermediate-to-low-temperature fuel cell devices, experimental verification of key proton conduction parameters—such as proton conductivity, proton diffusion coefficient—remains limited. Besides, electrolytes are often not fully densified during fabrication, which can lead to gas leakage and electrical leakage in single cells. This issue also poses challenges to their application in electrochemical synthesis under electrolysis conditions.

## 3 Optimization Strategy

### 3.1 Element Doping

Elemental doping is a central strategy for tuning the performance of Ba-based proton-conducting perovskites by modifying defect chemistry, lattice energetics, and transport properties. A-site doping, through partial substitution of Ba^2+^ with smaller or monovalent cations, introduces oxygen vacancies and adjusts lattice geometry, improving hydration, mobility, and chemical stability. B-site doping, using trivalent, tetravalent, or donor cations, offers fine control over vacancy populations, proton migration barriers, and chemical robustness, often employing codoping to balance conductivity and durability. Recently, O-site (anion) doping has emerged as a complementary approach, where substituting oxygen with electronegative anions like F^–^ improves lattice stability and proton transport despite reduced vacancy content. Together, these doping strategies provide a versatile design framework for developing next-generation proton-conducting materials for energy applications (Figure 20).

**Figure 20:**
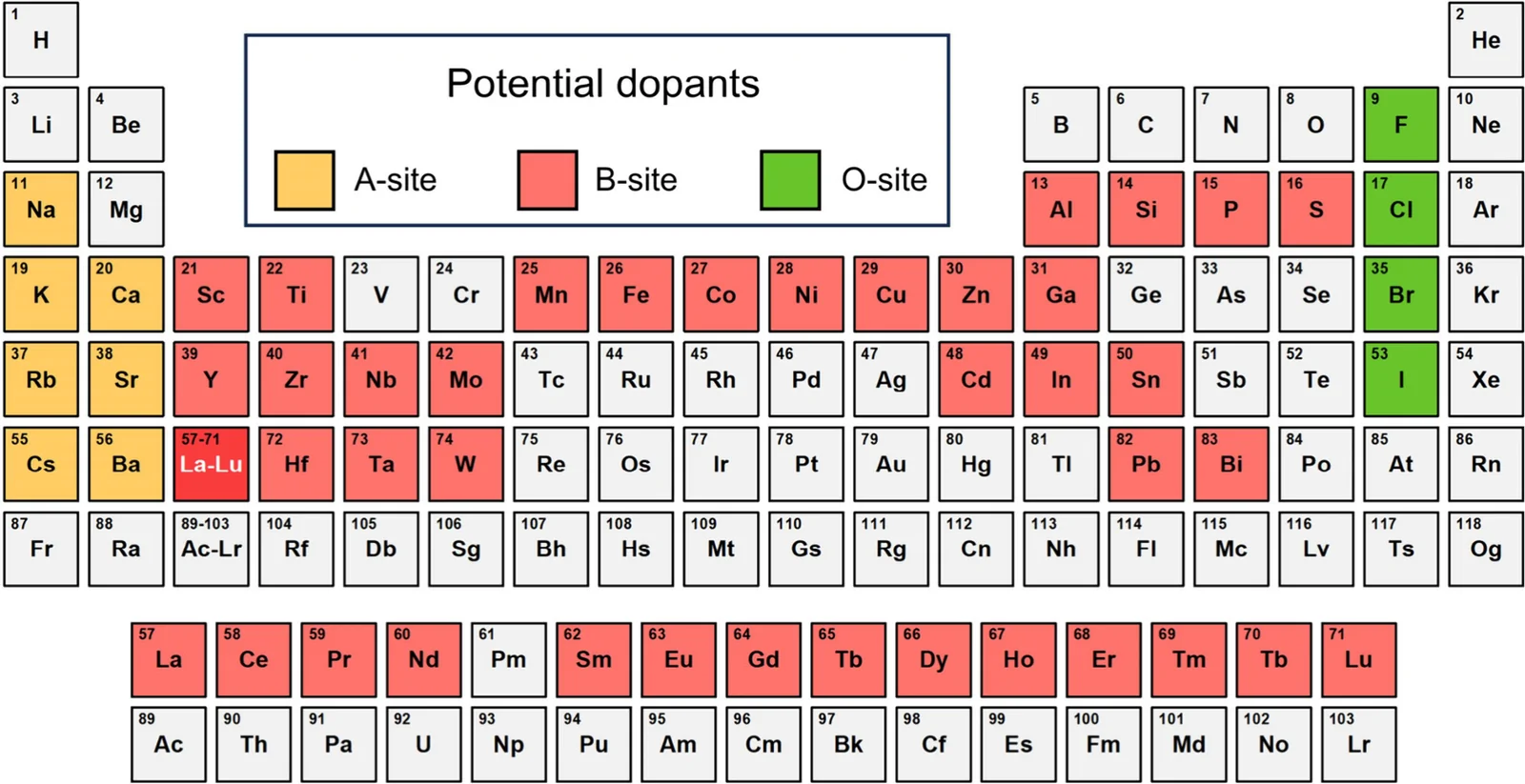
Potential dopants for different sites in perovskite oxides. Orange: A-site, red: B-site, and green: O-site.

#### 3.1.1 A-Site Doping

Proton-conducting perovskite oxides, such as BaCeO_3−δ_ and BaZrO_3−δ_, have garnered sustained research interest for applications in intermediate-temperature PCFCs, solid oxide electrolysis cells (SOECs), hydrogen separation membranes, and catalytic membranes. In these materials, proton transport relies on the incorporation of water into oxygen vacancies, forming mobile protonic defects (OH_O_^•^) that migrate through the oxide lattice predominantly via Grotthuss-type hopping mechanisms. Historically, B-site acceptor doping, where trivalent cations (e.g., Y^3+^, Gd^3+^, Sm^3+^, In^3+^) replace tetravalent B-site cations (Ce^4+^ or Zr^4+^), has been the standard approach for generating oxygen vacancies and enhancing proton conductivity. However, this doping strategy is not without drawbacks: the strong Coulombic interaction between the acceptor dopant and protons can lead to the formation of deep protonic trap states, elevating activation barriers and limiting long-range proton diffusion. Moreover, B-site doping can destabilize the perovskite lattice under certain chemical environments, such as CO_2_- or H_2_O-rich atmospheres, especially in BaCeO_3−δ_-based systems. B-site substitution modulates the acidity/basicity of the perovskite lattice primarily by altering the electronic structure and covalency of the B–O bonds within the BO_6_ octahedral framework, which constitutes the structural and chemical backbone of the perovskite. Because lattice oxygen atoms are directly coordinated to B-site cations, changes in B-site electronegativity, valence state, and orbital hybridization strongly influence the electron density on oxygen and, consequently, its effective Lewis basicity.^20,21,135^ Dopants that increase B–O bond covalency or withdraw electron density from oxygen reduce the electron-donating ability of lattice oxygen, rendering it more Lewis acidic and chemically reactive toward acidic species.

When B-site doping increases the lattice acidity, the weakened basic character of oxygen enhances its susceptibility to external chemical interactions, particularly with CO_2_ and H_2_O. In CO_2_-containing atmospheres, acidic lattice oxygen facilitates carbonate formation through reactions involving Ba–O and surface oxygen species, leading to the formation of BaCO_3_ and concomitant lattice decomposition. Similarly, under humid conditions, increased lattice acidity lowers the energetic barrier for hydroxylation and hydrothermal degradation, accelerating structural destabilization.^254^ These effects are often amplified by the concurrent increase in oxygen vacancy concentration introduced by aliovalent B-site doping as oxygen vacancies serve as highly reactive adsorption and incorporation sites for both CO_2_ and H_2_O. Conversely, B-site dopants that decrease lattice acidity, either by increasing electron density on oxygen or by strengthening ionic B–O bonding, enhance the Lewis basicity of lattice oxygen and suppress chemical reactivity toward acidic gases. Such dopants raise the thermodynamic penalty for carbonate and hydroxide formation, thereby improving tolerance to CO_2_- and H_2_O-rich environments.^21^ This sensitivity of chemical stability to B-site chemistry highlights that B-site substitution affects degradation behavior not only through defect introduction but also through fundamental modifications of lattice electronic states, oxygen bonding characteristics, and vacancy energetics, which together govern both surface and bulk reactivity.

To address these challenges, A-site doping has emerged as an increasingly attractive alternative or complementary strategy. A-site modification involves the partial substitution of Ba^2+^ on the perovskite A-site with smaller divalent cations (e.g., Ca^2+^ and Sr^2+^) or monovalent alkali cations (e.g., Na^+^, K^+^, Rb^+^, and Cs^+^). This approach simultaneously introduces charge-compensating oxygen vacancies and modifies the global lattice geometry and local defect environment, thereby influencing not only protonic defect formation but also defect transport, hydration thermodynamics, chemical stability, and sinterability (Table 3).

**Table 3:** Key Features of A/B/O Sites Doping Strategy on Ba­(Ce, Zr)­O_3_-Based Electrolytes

Doping Site		Representative Composition	Dopant(s)	Key Effects	Performance Highlights	Ref.
A-site		Ba_1–x_A_x_ZrO_3−δ_ (A = Na,K,Rb,Cs)	Na^+^, K^+^, Rb^+^, Cs^+^	Create oxygen vacancy; reduce proton-dopant association	High hydration enthalpy; reduced trapping	^255^
		Ba_1‑x_Ca_x_Ce_0.5_Zr_0.3_Y_0.2_O_3‑d_	Ca^2+^	Lattice shrink	Improved CO_2_ tolerance and sinterability; Low conductivity	^258^
B-site	Acceptor doping	BaZr_0.1_Ce_0.7_Y_0.1_Yb_0.1_O_3−δ_	Optimize oxygen vacancy concentration	High conductivity and improved Ni compatibility; instability against high concentrations of H_2_O	10^–2^ S/cm at 500 °C, instability against high concentrations of H_2_O	^37^
		BaHf_0.1_Ce_0.7_Yb_0.2_O_3−δ_				^100^
	Tetravalent doping	BaZr_0.4_Ce_0.4_Y_0.1_Yb_0.1_O_3−δ_	Increase the acidity of the perovskite lattice	High stability against CO_2_	High stability against CO_2_	^20^
		BaHf_0.3_Ce_0.5_Y_0.1_Yb_0.1_O_3−δ_				^21^
		BaSn_0.3_Ce_0.5_Yb_0.2_O_3−δ_				^135^
	Donor doping	BaNb_0.05_Ce_0.7_Yb_0.25_O_3−δ_	Stronger B–O bond; need excess acceptor codoping for vacancies	Sufficient tolerance against high concentrations of H_2_O and similar conductivity to BZCYYb1711	Sufficient tolerance against high concentrations of H_2_O and similar conductivity to BZCYYb1711	^65^
		BaTa_0.05_Ce_0.7_Yb_0.25_O_3−δ_				^264^
		BaMo_0.03_Ce_0.71_Yb_0.26_O_3−δ_				
		BaW_0.03_Ce_0.71_Yb_0.26_O_3−δ_				
O-site		BaZr_0.8_Y_0.2_O_2.9−δ_F_0.1_	F^–^	Lower basicity; modified vacancy distribution	Improved chemical durability	^267^
		BaCe_0.8_Sm_0.2_O_2.9−δ_F_0.1_				^268^

Recent first-principles DFT investigations by Kang and Sholl have provided key mechanistic insights into the effects of A-site alkali metal doping on BaZrO_3−δ_, revealing systematic trends in proton–dopant binding energies, hydration energetics, and carbonate formation tendencies. Notably, their work demonstrated that proton–dopant binding energies decrease progressively with increasing alkali metal ionic radius, ranging from ∼ −0.33 eV for Na^+^, −0.24 eV for K^+^, −0.17 eV for Rb^+^, and as low as −0.10 eV for Cs^+^. These values are markedly lower than the −0.5 eV or higher typically associated with Y^3+^ or other B-site dopants, indicating a reduced propensity for proton trapping and therefore higher effective proton mobility. Moreover, K^+^- and Rb^+^-doped systems were predicted to exhibit optimal combinations of low migration barriers and favorable hydration enthalpies, making them particularly promising for achieving superior bulk proton conductivity. Interestingly, while Cs^+^ doping offers the lowest calculated migration barrier, its relatively low solubility in the BaZrO_3−δ_ lattice limits its practical use at high doping levels.^255,256^

Complementary experimental and simulation work by Løken et al.^255^ further validated these theoretical predictions, emphasizing that A-site-doped BaZrO_3−δ_ systems benefit from enhanced hydration capacity, as evidenced by highly negative hydration enthalpies (e.g., −131 kJ mol^–1^ for Na^+^ and −90 to −83 kJ mol^–1^ for K^+^ and Cs^+^, respectively). These energetics indicate a strong thermodynamic driving force for water incorporation, ensuring a robust protonic defect population under humidified operating conditions. Crucially, the relatively low dopant–proton interaction strengths in A-site-doped systems ensure that these hydrated protons remain highly mobile, avoiding the deep trapping phenomena frequently observed in B-site-doped materials such as BaZr_0.9_Y_0.1_O_3−δ_. Together, these studies highlight the importance of simultaneously optimizing defect chemistry, solubility, and lattice dynamics to engineer high-performance proton-conducting electrolytes.

In addition to transport enhancements, A-site doping confers significant improvements in chemical stability, particularly under CO_2_- and H_2_O-rich environments that would otherwise degrade BaCeO_3−δ_-based perovskites. Kang and Sholl’s DFT work showed that K-doped BaZrO_3−δ_ exhibits the best balance of high proton conductivity and minimal carbonate formation tendency among the alkali metal dopants, addressing one of the key challenges associated with perovskite electrolytes operating in realistic gas atmospheres. This enhanced stability arises not only from the inherent chemical resilience of BaZrO_3−δ_ but also from the modified lattice energetics introduced by A-site doping, which reduces the reactivity of surface sites toward CO_2_ adsorption and incorporation.

Atomistic simulations by Wu et al.^257^ on BaCeO_3−δ_-based materials revealed additional subtleties regarding cation site preference and partitioning. Under Ba-deficient or high-temperature synthesis conditions, large trivalent dopants such as Nd^3+^ can occupy either the A- or B-site, but the consequences for defect chemistry are profoundly different. Specifically, when Nd^3+^ occupies the A-site, it does not generate oxygen vacancies, as its +3 charge does not require the same level of charge compensation as when substituted onto the B-site. This leads to a reduction in hydration capacity and overall proton conductivity, underscoring the critical importance of controlling dopant site selectivity and synthesis conditions to achieve the desired defect and transport profiles.^257^

Experimental studies by Zhou et al.^258^ on Ca^2+^-doped BaCe_0.5_Zr_0.3_Y_0.2_O_3−δ_ have further elucidated the dual role of A-site doping in enhancing both chemical durability and sintering behavior. Partial substitution of Ba^2+^ by Ca^2+^ leads to a reduction in lattice volume, which can slightly reduce total conductivity due to restricted lattice-free volume but significantly improves CO_2_ resistance and mechanical robustness. Moreover, Ca doping promotes densification and lowers the sintering temperature, facilitating the fabrication of dense, crack-free membranes that are crucial for long-term operational reliability. This balance between chemical and mechanical performance is essential for practical device integration and highlights the multifaceted benefits of A-site modification beyond mere conductivity optimization.^258^

Furthermore, A-site doping has also been applied to pyrochlore oxides, as mentioned in Section 2.7. In an additional study, Eurenius et al.^259^ systematically investigated the proton conductivity of Ca-doped Sm_2_Ti_2_O_7−δ_. Impedance measurements revealed enhanced σ_bulk_ below 500 °C under wet conditions, indicative of proton conduction. The Sm_1.92_Ca_0.08_Ti_2_O_7−δ_ sample showed higher conductivity of 1.6 × 10^–5^ S cm^–1^ in Ar (3% H_2_O) atmosphere at 300 °C. Isotope experiments and thermogravimetric results indicate that the Ca-doped samples exhibit excellent proton uptake capacity under humid conditions, demonstrating that A-site doping can serve as a simple and efficient regulatory approach.

Looking ahead, the integration of A-site and B-site codoping strategies represents a fertile design space for next-generation proton-conducting oxides. By leveraging the distinct advantages conferred by each doping site—such as A-site lattice tuning, hydration enhancement, and stability improvements, combined with B-site-driven oxygen vacancy generation and compositional flexibility—researchers aim to develop materials with unprecedented levels of conductivity, chemical durability, and processability. Advanced computational methods, including high-throughput DFT screening, machine learning-guided materials discovery, and kinetic Monte Carlo modeling, are increasingly being used to navigate the vast compositional landscape of A/B codoped perovskites. Experimental validation through controlled synthesis, detailed microstructural characterization, and comprehensive electrochemical testing will be essential to translate these computational predictions into functional materials. Together, these integrated approaches are poised to unlock the full potential of A-site doping strategies, driving innovation in proton-conducting ceramics and accelerating their deployment in commercial solid oxide electrochemical devices.

#### 3.1.2 B-Site Doping

##### 3.1.2.1 Acceptor Doping

Acceptor doping on the B-site of perovskite oxides, especially in systems such as BaCeO_3−δ_ and BaHfO_3−δ_, has been a cornerstone strategy for achieving high protonic conductivity under intermediate-temperature conditions (400–700 °C). In these materials, the substitution of trivalent acceptor cations (Y^3+^, Yb^3+^, Gd^3+^, Sm^3+^, Er^3+^) for tetravalent B-site cations (Ce^4+^ or Hf^4+^) introduces charge-compensating oxygen vacancies (V_O_^••^) in the lattice (eq 9). Under humidified atmospheres, these vacancies readily incorporate water through dissociative hydration, generating mobile protonic defects (OH_O_^•^) that migrate across the perovskite lattice via Grotthuss-type hopping mechanisms. The extent to which these dopants improve conductivity, however, depends on multiple intertwined factors, including dopant size, defect association, hydration thermodynamics, lattice distortions, chemical compatibility, and microstructural stability.

9 $${\mathrm{Yb}}_{2}O_{3}+2{\mathrm{Ce}}_{\mathrm{Ce}}^{\times }+O_{O}^{\times }\to 2{\mathrm{Yb}}_{\mathrm{Ce}}^{’}+V_{O}^{¨}+2{\mathrm{CeO}}_{2}$$

A representative composition, BZCYYb1711, has been the subject of extensive investigation. As shown in Figure 21a–c, it offers several key advantages over earlier BaCeO_3−δ_- or BaZrO_3−δ_-based electrolytes.^37^ First, by codoping with both Y^3+^ (large ionic radius) and Yb^3+^ (small ionic radius), the B-site lattice can be finely tuned to balance lattice expansion, oxygen vacancy concentration, and free volume, thereby optimizing protonic defect mobility.^37^ This codoping approach mitigates excessive lattice distortion, improves hydration thermodynamics, and enhances long-range proton diffusion. Experimental studies have shown that BZCYYb1711 compositions achieve proton conductivities on the order of 10^–2^ S cm^–1^ at 500 °C, outperforming many earlier perovskite-based proton conductors.^37^

**Figure 21:**
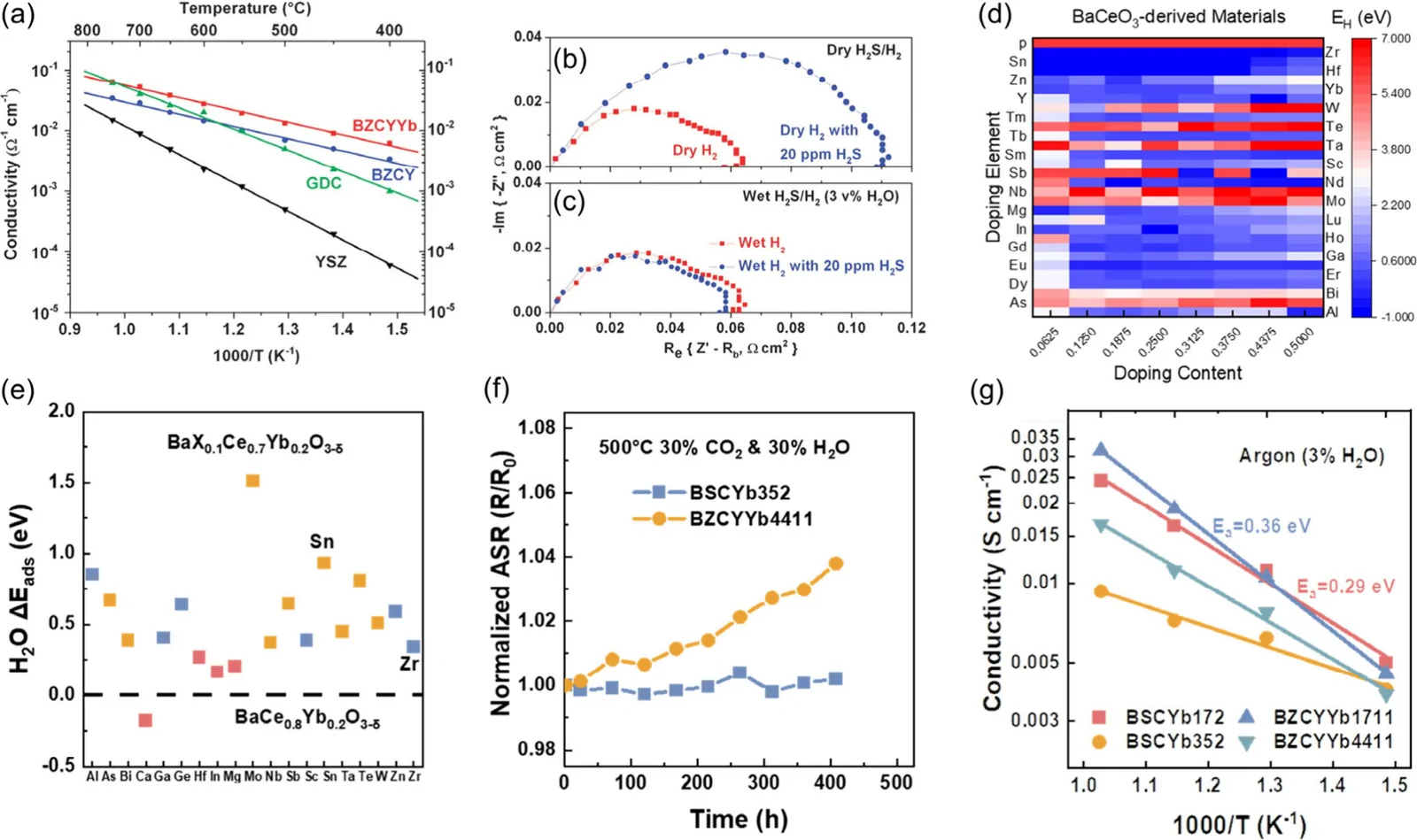
(a) Ionic conductivities of BZCYYb1711, BZCY, GDC, and YSZ measured between 400 and 750 °C in wet oxygen (∼3 vol % H_2_O). Impedance spectra recorded under OCV conditions at 750 °C for a Ni–BZCYYb1711|SDC|LSCF cell in (b) dry H_2_ with 20 ppm of H_2_S and (c) wet H_2_ with 20 ppm of H_2_S (∼3 vol % H_2_O). Reproduced with permission from ref 37. Copyright 2009 AAAS. (d) High-throughput calculation results of E_H_ of BaCeO_3−δ_-derived materials. (e) H_2_O adsorption energies at 0 K. (f) BaSn_0.3_Ce_0.5_Yb_0.2_O_3−δ_ (BSCYb352) and BZCYYb4411 when exposed to 30% CO_2_ and 30% H_2_O in Ar at 500 °C for over 400 h. (g) Conductivity of BaSn_0.1_Ce_0.7_Yb_0.2_O_3−δ_ (BSCYb172), BSCYb352, BZCYYb1711, and BZCYYb4411 as a function of temperature. Reproduced from ref 135. Available under a CC-BY-NC-ND 4.0 license. Copyright 2024 Wiley Zheyu Luo et al.

Beyond its intrinsic conductivity, Ba­(Ce, Zr)­O_3_-derived materials exhibit remarkable chemical and catalytic durability under fuel cell operating conditions. A critical challenge for conventional Ni–yttria-stabilized zirconia (YSZ) anodes is their susceptibility to sulfur poisoning and carbon coking when exposed to hydrocarbon fuels or sulfur-containing gas streams. BZCYYb1711-based systems effectively suppress these degradation mechanisms due to their mixed conducting nature and catalytic surface properties. Under exposure to 20–50 ppm of H_2_S, BZCYYb1711-supported cells maintain stable power outputs, facilitated by the material’s ability to oxidize adsorbed sulfur species (e.g., converting H_2_S to SO_2_), which desorbs readily, thereby minimizing sulfur accumulation. In addition, the material’s strong hydration behavior supports *in situ* steam reforming and partial oxidation reactions, promoting hydrocarbon cracking and reducing carbon deposition on the anode surface.^37^

It is important to recognize that, while acceptor dopants such as Y^3+^ are typically considered B-site substituents in perovskite oxides, their relatively large ionic radius allows for partial occupation of the A-site under certain conditions. Liu et al.^260^ demonstrated that, during high-temperature sintering of BZCYYb1711, Y^3+^ dopants can dynamically migrate from the B-site to the A-site, leading to pronounced lattice shrinkage, a reduction in oxygen vacancy concentration, and poor densification. These structural changes negatively affect both proton conductivity and membrane integrity. To address this issue, a molten salt synthesis method was employed to preincorporate Y^3+^ into the A-site at lower temperatures, thereby suppressing cation redistribution during sintering.^260^ This approach resulted in improved densification, enhanced proton conductivity, and greater long-term structural stability. The findings highlight the critical role of controlled cation site occupancy in optimizing the processing and performance of proton-conducting perovskite electrolytes.

More recently, Luo et al.^100^ performed a comprehensive investigation of acceptor-doped BaHf_0.1_Ce_0.7_R_0.2_O_3−δ_ (R = Yb, Er, Y, Gd, Sm), combining systematic experimental evaluation with DFT-based thermodynamic analysis. Their work uncovered clear correlations between the identity of the acceptor dopant and the resulting conductivity, chemical durability, and electrode compatibility. Smaller-radius dopants such as Yb^3+^ were shown to significantly improve chemical compatibility with NiO during cosintering, reducing the formation of deleterious secondary phases such as BaR_2_NiO_5_, and thus enhancing the mechanical and chemical stability of the electrolyte–electrode interface. Furthermore, Yb-doped systems exhibited higher Gibbs free energy barriers for CO_2_ and H_2_O reactions, confirming their superior resistance to carbonation and hydrothermal degradation compared to Er^3+^- and Y^3+^-doped analogs. Optimally doped BHCYb172 materials achieved protonic conductivities of ∼ 2 × 10^–2^ S cm^–1^ at 600 °C.^100^

From a mechanistic standpoint, acceptor B-site doping exerts a complex interplay of effects. While oxygen vacancy creation is necessary for hydration and proton incorporation, excessive acceptor doping can lead to local dopant–proton association, effectively trapping protons and increasing the activation energy for long-range migration.^261^ Moreover, the size mismatch between the dopant and the host B-site cation plays a crucial role in tuning the lattice distortion and free volume, which in turn modulates the migration pathways and diffusion coefficients of protonic defects.^262^ Computational studies have shown that moderate lattice distortions can facilitate proton transport by opening wider diffusion channels, whereas excessive distortions or local strain accumulation can disrupt the percolation network and thereby reduce macroscopic conductivity.^263^ Hence, optimal doping levels and dopant combinations are essential to maximize both hydration capacity and protonic mobility without inducing mechanical or chemical instability.

Another critical aspect is the chemical compatibility of acceptor-doped electrolytes with adjacent electrode materials, especially Ni-based fuel electrodes commonly used in SOFC and SOEC architectures. Luo et al.^100^ showed that rare-earth dopants with smaller ionic radii, such as Yb^3+^, reduce interfacial reactivity and suppress the formation of barium–nickel secondary phases, leading to clean and stable electrolyte–electrode interfaces that are crucial for long-term device operation. This finding underscores the importance of not only optimizing the bulk electrolyte properties but also considering interfacial thermodynamics and processing compatibility when designing acceptor-doped perovskite electrolytes for practical applications.

##### 3.1.2.2 Tetravalent Doping

Tetravalent B-site doping has emerged as a transformative strategy in the development of chemically robust and high-conductivity Ba-based perovskite proton-conducting electrolytes, addressing the long-standing challenge of balancing ionic transport performance with thermochemical stability. In classical systems such as BaCeO_3−δ_- and BaZrO_3−δ_-based materials, proton conduction relies on the incorporation of oxygen vacancies, typically introduced via trivalent B-site (acceptor) doping, followed by hydration to generate mobile protonic defects. However, these systems, particularly Ce-rich compositions, suffer from severe chemical degradation under CO_2_- and H_2_O-rich conditions, limiting their operational durability in fuel cell and electrolysis environments. Tetravalent doping—substituting the B-site Ce^4+^ or Zr^4+^ with alternative tetravalent cations such as Hf^4+^ or Sn^4+^—offers a compelling approach to decouple chemical stability improvements from defect generation mechanisms, enabling independent control over lattice energetics, defect dynamics, and phase robustness.

Murphy et al.^21^ provided one of the most comprehensive experimental demonstrations of tetravalent B-site doping in the BHCYYb system. Their work systematically evaluated the electrochemical performance, chemical durability, and thermodynamic stability of Hf-doped compositions, revealing that moderate Hf substitution (x ≈ 0.2–0.3) leads to substantial improvements in CO_2_ and H_2_O tolerance compared to conventional BZCYYb. Thermodynamic analyses showed that BaHfO_3−δ_ possesses a higher Gibbs free energy for CO_2_ reaction compared to BaCeO_3−δ_ and BaZrO_3−δ_, reducing the formation of secondary BaCO_3_ and CeO_2_ phases under CO_2_ exposure. Long-term testing under 700-h CO_2_–H_2_O coelectrolysis conditions confirmed the material’s chemical durability, with no significant performance degradation or phase decomposition observed. Importantly, the conductivity trends identified a nontrivial optimum: while low-to-moderate Hf doping improved conductivity by enhancing lattice stability and suppressing Ce^4+^ reduction, excessive doping (x > 0.3) resulted in decreased protonic conductivity due to vacancy oversuppression and lattice over-rigidity, demonstrating the delicate balance required between defect concentration and structural fortification.^21^

Building on these experimental findings, Luo et al.^135^ employed a high-throughput DFT screening framework to systematically assess the effects of various tetravalent B-site dopants, including Zr^4+^, Hf^4+^, and Sn^4+^, on the structural, chemical, and transport properties of BaCeO_3−δ_- and BaZrO_3−δ_-derived perovskites. As shown in Figure 21d,e, their computational study encompassed over 900 candidate compositions, evaluating key parameters such as oxygen vacancy formation energy (E_V_), hydration energy (E_H_), proton migration barriers, and CO_2_/H_2_O adsorption energies. The simulations revealed that all three dopants substantially reduced E_V_ and E_H_ relative to undoped BaCeO_3−δ_, promoting easier hydration and proton incorporation even without additional acceptor dopants.^135^ Notably, Sn^4+^ stood out as particularly promising: Sn-doped BaCeO_3−δ_ exhibited the lowest E_V_ (approximately −5.85 eV) and E_H_ (approximately −6.3 eV), translating into high intrinsic hydration capacity. Experimental validation confirmed these predictions, with BaSn_x_Ce_0.8–x_Yb_0.2_O_3−δ_ outperforming Zr-doped counterparts in conductivity and maintaining exceptional stability under continuous carbon dioxide and steam exposure for over 400 h (Figure 21f,g). Moreover, DFT-calculated proton migration energy landscapes showed that Sn doping reduced the proton hopping barrier to ∼ 0.30 eV, significantly lower than the ∼ 0.47 eV observed in Zr-doped systems, enabling faster long-range proton diffusion.^135^

Mechanistically, tetravalent doping reshapes the perovskite’s BO_6_ octahedral framework, modifying tilt angles, unit cell dimensions, and local strain fields, which in turn alter the connectivity and percolation of proton migration pathways. Sn^4+^, in particular, was shown to smooth the activation energy landscape for proton migration, reducing the tendency for proton trapping and enhancing effective mobility. Furthermore, tetravalent dopants strengthen the B–O bond network, reducing the susceptibility of the lattice to chemical attack, electronic defect formation, and interfacial degradation. Murphy et al.^21^ experimentally observed that Hf-doped BaCeO_3−δ_ exhibited reduced BaO surface enrichment, suppressed CO_2_ adsorption, and enhanced grain boundary cohesion, as confirmed by XRD, TGA, and Raman spectroscopy, demonstrating the bulk and interfacial stability gains achievable through tetravalent doping.

However, the implementation of tetravalent B-site doping requires careful compositional optimization. Excessive doping levels can reduce the oxygen vacancy population to the point where hydration becomes kinetically hindered, diminishing protonic conductivity despite improved chemical resilience. Additionally, dopant solubility limits, phase stability boundaries, and sintering compatibility with adjacent electrodes—particularly Ni-based fuel electrodes—must be considered to avoid secondary phase formation or interfacial degradation during cell fabrication and operation.

##### 3.1.2.3 Donor Doping

Donor B-site doping has emerged as an innovative and powerful strategy for overcoming the intrinsic chemical instability of Ba-based proton-conducting perovskites, especially under harsh CO_2_- and H_2_O-rich conditions. While traditional approaches rely on B-site acceptor doping (e.g., Y^3+^, Yb^3+^) to generate oxygen vacancies for proton incorporation, donor doping introduces higher-valence cations—typically pentavalent (Nb^5+^, Ta^5+^) or hexavalent (Mo^6+^, W^6+^)—into the B-site lattice, enhancing the material’s acidity, bond strength, and lattice stability without necessarily increasing the oxygen vacancy population. Although according to eq 10 and eq 11, donor doping alone reduces vacancy concentration (and thus protonic defect density), carefully designed acceptor–donor codoping strategies can balance this effect, maintaining high proton conductivity while dramatically improving chemical durability. This dual optimization is crucial for advancing protonic-conducting RSOCs.

10 $${\mathrm{Nb}}_{2}O_{5}+2{\mathrm{Ce}}_{\mathrm{Ce}}^{\times }+V_{O}^{\cdot \cdot }\to 2{\mathrm{Nb}}_{\mathrm{Ce}}^{\cdot }+O_{O}^{\times }+2{\mathrm{CeO}}_{2}$$

11 $${\mathrm{MoO}}_{3}+{\mathrm{Ce}}_{\mathrm{Ce}}^{\times }+V_{O}^{\cdot \cdot }\to {\mathrm{Mo}}_{\mathrm{Ce}}^{\cdot \cdot }+O_{O}^{\times }+{\mathrm{CeO}}_{2}$$

Luo et al.^65^ systematically investigated the incorporation of Nb^5+^ and Ta^5+^ dopants into BaCeO_3−δ_-based perovskites, yielding compositions such as BaNb_0.05_Ce_0.7_Yb_0.25_O_3−δ_. These donor-doped materials exhibited substantially enhanced chemical stability relative to the benchmark acceptor-doped BZCYYb1711, especially under humid CO_2_ atmospheres. This improvement was attributed to the higher electronegativity and Lewis acidity of Nb^5+^ and Ta^5+^, which strengthen the B–O bond framework, reduce lattice polarizability, and suppress surface carbonation and hydrothermal degradation.

Luo et al.^65^ further showed that donor-doped systems without additional acceptor dopants suffer from insufficient oxygen vacancy concentrations and limited hydration. By incorporating excess Yb^3+^ as an acceptor dopant, the authors restored hydration capacity and achieved proton conductivities comparable to those of BZCYYb1711. Optimized BaNb_0.05_Ce_0.7_Yb_0.25_O_3‑δ_ and BaTa_0.05_Ce_0.7_Yb_0.25_O_3‑δ_ electrolytes exhibited bulk conductivities of ∼ 0.012 S cm^–1^ at 500 °C.

Critically, these codoped materials exhibited exceptional durability, showing almost no increase in R_o_ over 500 h of exposure to air containing 30% H_2_O. This behavior contrasts sharply with BZCYYb1711, which displays a normalized R_o_ increase of ∼ 6% under identical conditions. High-resolution structural analysis confirmed the stabilization of the perovskite phase, with suppressed Ba segregation and carbonate formation, thereby validating the chemical stabilization effects of donor doping.^65^

In addition, the donor–acceptor codoped electrolytes exhibited excellent compatibility with Ni-based fuel electrodes. Yb^3+^, used as the acceptor dopant, shows minimal reactivity with Ni-based materials during sintering when maintained below a critical concentration.^25,100^ As demonstrated by Luo et al.,^65^ both BaNb_0.05_Ce_0.7_Yb_0.25_O_3‑δ_ and BaTa_0.05_Ce_0.7_Yb_0.25_O_3‑δ_ show no evidence of secondary phase formation after sintering at a B-site Yb content of 25%, while maintaining proton conductivity comparable to BZCYYb1711.

Recent advances have expanded donor doping strategies in Ba-based perovskite electrolytes beyond the well-established pentavalent dopants (Nb^5+^ and Ta^5+^) to include hexavalent cations such as Mo^6+^ and W^6+^. As shown in Figure 22, DFT predictions suggest that these hexavalent donor dopants offer even greater improvements in chemical stability compared with Nb^5+^ and Ta^5+^.^65,264^

**Figure 22:**
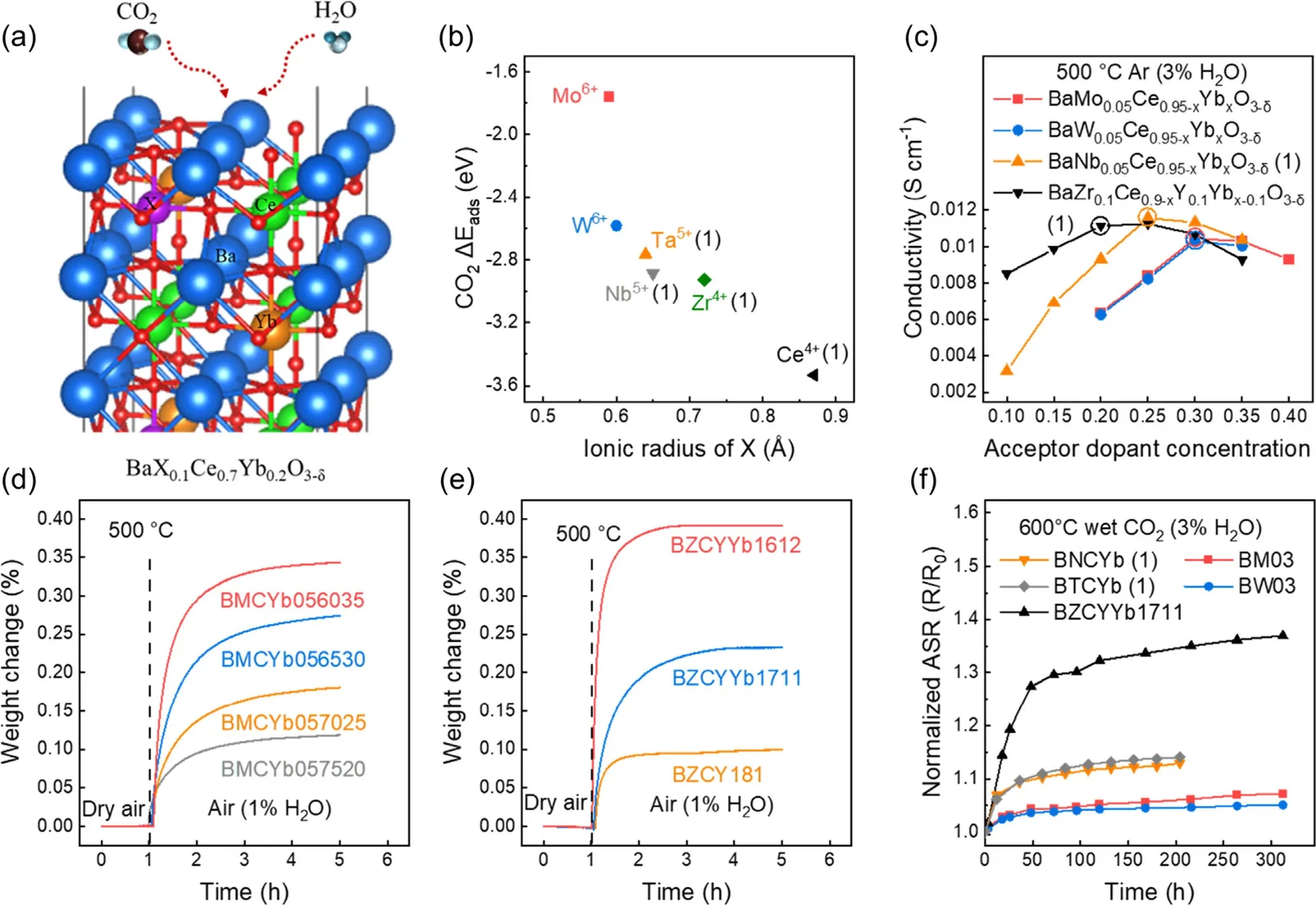
(a) Model of BaX_0.1_Ce_0.7_Yb_0.2_O_3−δ_ (X = Ce, Zr, Nb, Ta, Mo, W) for DFT-based calculations. (b) CO_2_ reaction energy at 0 K as a function of the ionic radius. (c) Conductivity of BaMo_0.05_Ce_0.95–x_Yb_x_O_3−δ_, BaW_0.05_Ce_0.95–x_Yb_x_O_3−δ_, BaNb_0.05_Ce_0.95–x_Yb_x_O_3−δ_, and BaZr_0.1_Ce_0.9–x_Y_0.1_Yb_x–0.1_ at 500 °C as a function of doping concentration on the B-site. Electrolyte protonation profile measured by TGA for: (d) BaMo_0.05_Ce_0.65_Yb_0.3_O_3−δ_ and (e) BZCYYb. (f) Change in resistance of BaMo_0.03_Ce_0.71_Yb_0.26_O_3−δ_, BaW_0.03_Ce_0.71_Yb_0.26_O_3−δ_, and BZCYYb1711 when exposed to wet CO_2_ (3% H_2_O) at 600 °C for over 300 h. Reproduced with permission from ref 264. Copyright 2023 Wiley.

Experimental evaluation showed that introducing 5% Mo^6+^ or W^6+^ into BaCeO_3−δ_-based systems raises the optimal B-site acceptor doping level (Yb^3+^) to 30–35%, compared to ∼ 25–30% in Nb^5+^-doped systems, due to the need to balance the additional positive charge and compensate for partially filled oxygen vacancies. TGA revealed that hexavalent donor–acceptor codoped compositions, such as BaMo_0.05_Ce_0.65_Yb_0.3_O_3−δ_, exhibited enhanced hydration capacity, even surpassing that of benchmark BZCYYb1711. This confirms that while donor doping blocks some oxygen vacancy sites, increasing the acceptor content can regenerate vacancies, rejuvenating hydration capability and enhancing proton conductivity. However, a delicate balance is required: although increasing acceptor doping boosts hydration and proton concentration, excessive doping can lower proton mobility due to dopant–vacancy association and trapping effects, ultimately reducing overall conductivity.

Crucially, chemical stability tests under harsh conditions (3% H_2_O in CO_2_ at 600 °C) demonstrated that Mo^6+^- and W^6+^-doped materials show minimal resistance changes (∼4–6% over 200 h), far outperforming both Nb^5+^/Ta^5+^-doped systems (∼11%) and standard BZCYYb1711 (∼32%). These findings highlight the superior chemical durability conferred by hexavalent donor doping, marking it as a highly promising approach for designing next-generation proton-conducting electrolytes capable of long-term operation in CO_2_- and H_2_O-rich environments.^264^

In summary, B-site doping has proven to be a versatile and powerful strategy for tailoring the performance of Ba-based proton-conducting perovskites (Table 3). Acceptor doping with trivalent cations like Y^3+^ and Yb^3+^ introduces oxygen vacancies essential for hydration and proton transport but requires careful optimization to avoid excessive lattice distortion and proton trapping. Tetravalent doping, using cations such as Hf^4+^ and Sn^4+^, enhances the lattice’s chemical stability, decoupling durability from defect generation and enabling robust operation under CO_2_- and H_2_O-rich conditions. Donor doping with pentavalent and hexavalent cations (Nb^5+^, Ta^5+^, Mo^6+^, W^6+^) further strengthens the B–O framework, dramatically improving chemical resistance while, when paired with acceptor codoping, maintaining high protonic conductivity. Together, these doping strategies offer a finely tunable toolbox for engineering electrolytes that balance conductivity, hydration capacity, chemical durability, and electrode compatibility—key factors for advancing solid oxide fuel cells, reversible solid oxide cells, and protonic ceramic electrolysis technologies.

Looking ahead, future research on B-site doping in proton-conducting oxides is poised to benefit from the integration of high-throughput computational screening, machine-learning-assisted materials discovery, and advanced *in situ* characterization techniques. Emerging trends include the design of synergistic multidopant systems that combine the advantages of acceptor, tetravalent, and donor doping within a unified lattice framework, aiming to simultaneously optimize conductivity, chemical stability, mechanical robustness, and processing compatibility. Furthermore, understanding the interplay between bulk defect chemistry and interfacial phenomena at electrolyte–electrode boundaries will be crucial for improving device-level performance and lifetime. As the field moves toward commercialization, addressing dopant solubility limits, secondary phase suppression, and scalable fabrication will be key challenges. Ultimately, B-site doping, combined with complementary strategies such as A-site engineering, nanostructured architectures, and composite material designs, offers a promising path toward the next generation of high-performance, durable protonic ceramic devices for clean energy applications.

#### 3.1.3 O-Site Doping

O-site (anion) doping, involving the partial substitution of O^2–^ with alternative electronegative anions such as fluoride (F^–^), has emerged as a promising and novel strategy to advance Ba-based proton-conducting perovskite materials (Table 3). While much of the research in protonic ceramic electrolytes has traditionally focused on cationic (A- and B-site) doping to tune defect chemistry and ionic transport, engineering the anion sublattice offers distinct opportunities to modify lattice energetics, electronic structure, and chemical resilience in ways that directly enhance proton incorporation, migration, and long-term stability. This strategy holds particular promise for addressing long-standing limitations of BaCeO_3−δ_- and BaZrO_3−δ_-based electrolytes, including susceptibility to CO_2_ degradation, poor phase stability under humid conditions, and the delicate trade-off between maintaining sufficient oxygen vacancy populations and achieving efficient proton transport.

Su and colleagues systematically investigated the fluoride-doping approach to BaCe_0.8_Sm_0.2_O_3−δ_ (BCS), one of the most conductive Ce-based perovskite electrolytes but historically plagued by poor chemical stability under CO_2_-rich conditions. By partially substituting O^2–^ with F^–^, they synthesized F-doped BaCe_0.8_Sm_0.2_O_3−δ_ (BCSF), a composition that retained high proton conductivity while dramatically improving CO_2_ tolerance. Long-term exposure experiments revealed that BCSF maintained its perovskite phase integrity and microstructural cohesion even after 72 h at elevated temperatures under CO_2_-rich atmospheres, whereas undoped BCS rapidly developed insulating BaCO_3_ and CeO_2_ secondary phases that degraded performance. Electrochemical fuel cell tests showed a 20–60% improvement over undoped BCS, while open-circuit voltages remained stable over 146-h durability testing.

From an electronic-structure perspective, fluoride (electronegativity 3.90) is significantly more electronegative than oxide (3.44). When F^–^ substitutes for O^2–^ at anion sites of BaCeO_3−δ_, Su et al.^265^ showed by XRD and X-ray photoelectron spectroscopy (XPS) that F^–^ is indeed incorporated into the perovskite lattice, forming stronger and more ionic Ce–F (and Sm–F) bonds. The increased bond ionicity and stronger B–F bonding withdraw electron density from the surrounding anion framework, thereby reducing the electron-donating ability of neighboring O^2–^ ions. This decrease in lattice electron density at oxygen sites weakens the basicity of surface hydroxyl and oxide species, directly lowering Brønsted basicity and markedly improving resistance to reaction with acidic gases such as CO_2_.^266^ Beyond electronic effects, fluoride substitution also alters the defect chemistry and associated defect energetics.^267^ In Kröger–Vink notation, codoping with Sm_2_O_3_ and BaF_2_ introduces acceptor dopants on the B-site and F^–^ species on the anion sublattice, along with Ba and O vacancies that can mutually annihilate. This charge-compensation mechanism modifies both the concentration and spatial distribution of oxygen vacancies relative to the purely Sm-doped system. Su et al.^265^ further proposed that anion-site F^–^ species can interact with B-site acceptor dopants and hydratable vacancies, thereby weakening strong proton-trapping associations. This “proton detrapping” effect enhances the effective proton mobility and contributes to the improved transport performance.

Gao et al.^267^ extended fluoride doping on the oxygen site of BZY20, forming BaZr_0.8_Y_0.2_O_3−δ−σ_F_σ_ compositions with σ ≈ 0.1. Through a combination of DFT simulations and experimental characterization, they demonstrated that F^–^, due to its higher electronegativity compared to O^2–^, reshapes the local charge distribution and weakens the metal–oxygen bond strength, particularly around the ZrO_6_ octahedra. This modification enhances lattice polarizability, lowers the migration barriers for protonic and oxide-ion defects, and effectively improves total ionic conductivity, even though F^–^ substitution slightly reduces the oxygen vacancy concentration. EIS confirmed that F-doped BZY20 achieved significantly higher conductivities than its undoped counterpart over a broad intermediate-temperature range, with particularly pronounced performance under reducing conditions where the mixed proton and oxide-ion transport is dominant. Single-cell fuel cell tests further validated these materials, showing increased peak power densities, reduced ohmic and polarization losses, and enhanced durability during thermal cycling, underscoring the practical impact of O-site doping as a lattice engineering strategy.^267^

Mechanistically, O-site doping modifies the perovskite system through several interconnected effects. First, the introduction of highly electronegative anions like F^–^ lowers the lattice’s basicity, reducing its chemical reactivity toward acidic species such as CO_2_ and H_2_O, which in turn suppresses decomposition pathways and improves long-term chemical durability. Second, anion substitution alters the Coulombic interactions between cations and anions, weakening B–O bond strengths, decreasing proton and oxygen migration barriers, and improving defect mobility throughout the lattice. Third, although F^–^ doping may nominally reduce the oxygen vacancy population, the combination of enhanced defect dynamics and stabilized lattice structure often leads to a net improvement in effective protonic conductivity, as shown by both computational models and experimental validation.

Looking forward, O-site doping opens up a largely underexplored frontier for the rational design of advanced proton-conducting electrolytes. Beyond fluoride, future research should systematically explore alternative anionic dopants, including chloride (Cl^–^), bromide (Br^–^), nitride (N^3–^), and sulfide (S^2–^), which could introduce novel modifications to lattice dynamics, defect chemistry, and hydration behavior. Combining anion doping with advanced cation codoping strategies offers the potential to synergistically optimize multiple properties, including conductivity, hydration thermodynamics, chemical durability, mechanical strength, and electrode compatibility. Realizing these opportunities will require the integration of cutting-edge experimental techniques—such as neutron scattering, synchrotron-based XAS, and atom probe tomography (APT)—with high-throughput computational screening and machine-learning-guided materials design. Addressing practical challenges related to dopant solubility, phase stability, long-term operational performance, and scalable synthesis will be essential for translating these promising materials into commercial protonic ceramic devices, enabling next-generation solid oxide fuel cells, reversible solid oxide cells, proton-conducting electrolyzers, and hydrogen separation technologies that meet the growing demands of the clean energy transition.

#### 3.1.4 High-Entropy Doping

High-entropy proton-conducting oxides have recently emerged as a new class of electrolyte materials for protonic ceramic electrochemical cells, offering an alternative design paradigm beyond conventional dilute-doping strategies. In contrast to traditional perovskite proton conductors, which rely on the substitution of one or two dopant species to tailor defect chemistry, high-entropy oxides incorporate five or more cations in near-equimolar or deliberately engineered nonequimolar ratios within a single crystallographic sublattice. This compositional complexity leads to high configurational entropy, which plays a central role in stabilizing single-phase solid solutions and modifying the thermodynamic and transport properties of the material.^269^

From a thermodynamic perspective, the incorporation of multiple cation species increases the entropy contribution to the Gibbs free energy, thereby stabilizing the perovskite structure against decomposition at elevated temperatures. This entropy-driven stabilization is particularly beneficial for proton-conducting electrolytes, as it can suppress the formation of carbonate and hydroxide phases that typically limit the durability of BaCeO_3−δ_-based materials in CO_2_- and H_2_O-containing environments. At the same time, the presence of multiple cations introduces local lattice distortions and complex potential energy landscapes, which can influence oxygen vacancy formation, hydration behavior, and proton migration pathways. These competing effects give rise to the so-called “cocktail effect,” whereby the collective interactions of multiple elements produce emergent properties unattainable in conventional simple-component systems.

Early investigations into high-entropy perovskite oxides demonstrated the feasibility of incorporating multiple cations into a single-phase structure; however, their proton transport properties were initially limited. For example, early systems exhibited proton conductivities on the order of 10^–4^ S cm^–1^ at 600 °C, significantly lower than those of conventional Ba­(Ce, Zr)­O_3−δ_-based electrolytes.^270^ These relatively poor transport properties were primarily attributed to microstructural limitations, including insufficient densification, small grain sizes, and high R_gb_, as well as excessive lattice disorder that hindered long-range proton diffusion. These findings highlighted the critical importance of simultaneously optimizing composition and processing in high-entropy systems to achieve competitive performance.

More recent studies have demonstrated that rational compositional design and advanced synthesis strategies can substantially improve the electrochemical properties. Guo et al.^271^ reported a nonequimolar high-entropy perovskite BaSn_0.16_Zr_0.24_Ce_0.35_Y_0.1_Yb_0.1_Dy_0.05_O_3−δ_, which exhibited a proton conductivity of 8.3 mS cm^–1^ at 600 °C, representing a significant improvement over earlier high entropy systems. The enhanced performance was attributed to improved densification achieved through wet-chemical synthesis and to optimized defect chemistry enabled by tailored nonequimolar cation distributions. Similarly, Yang et al.^270^ developed high-entropy compositions based on BaCeO_3−δ_-BaZrO_3−δ_ solid solutions, such as BaSn_0.15_Ce_0.35_Zr_0.25_Y_0.1_Yb_0.1_Gd_0.05_O_3−δ_, which demonstrated high proton transport numbers and improved proton mobility at intermediate temperatures. The incorporation of multiple trivalent dopants was shown to increase oxygen vacancy concentration while balancing lattice distortion, thereby facilitating proton transport without severely compromising structural stability.

In parallel, equimolar high-entropy systems have also shown promising results in terms of both conductivity and chemical stability. Oh et al.^272^ reported a six-component high-entropy perovskite BaHf_1/6_Sn_1/6_Zr_1/6_Ce_1/6_Y_1/6_Yb_1/6_O_3−δ_, which exhibited improved total conductivity compared to previously reported high entropy electrolytes and demonstrated excellent resistance to CO_2_-induced degradation. When implemented in reversible protonic ceramic electrochemical cells, this material enabled high power densities and current densities in both fuel cell and electrolysis modes, highlighting the potential of high entropy proton-conducting electrolytes for practical device applications.

Despite these advances, several fundamental challenges remain. It should be noted that the performance improvements reported for high-entropy proton-conducting electrolytes are largely attributable to enhanced densification and reduced R_gb_, rather than fundamentally new transport mechanisms. In most cases, the effects of multication substitution on conductivity and stability remain predictable based on conventional dopant chemistry, and no system has yet fully overcome the intrinsic trade-off between high proton conductivity and chemical robustness. Given the vast compositional space enabled by high-entropy design, reliance on empirical approaches is insufficient, highlighting the urgent need for high-throughput computational screening and data-driven strategies to guide future material discovery. The complex interplay between configurational disorder and proton transport is not yet fully understood, particularly concerning proton trapping and the distribution of local migration barriers. In addition, achieving high-density ceramics without compositional segregation remains a key processing challenge because of the multicomponent nature of these materials. Furthermore, the optimization of electronic transport properties and compatibility with electrode materials must be carefully considered for practical device integration.

Overall, high-entropy proton-conducting oxides represent a promising and rapidly evolving class of electrolyte materials. By enabling simultaneous tuning of defect chemistry, lattice structure, and thermodynamic stability, high-entropy design provides a powerful framework for overcoming the intrinsic limitations of conventional BaCeO_3−δ_- and BaZrO_3−δ_-based systems. Continued efforts in compositional optimization, advanced synthesis techniques, and mechanistic understanding are expected to further enhance the performance of these materials and facilitate their integration into next-generation protonic electrochemical devices.

### 3.2 Deficiency Engineering

Most perovskite materials exhibit a high tolerance for cation nonstoichiometry without disrupting their fundamental crystal structures.^52,237^ Such tolerance permits the formation of A- and B-site cation deficiencies, thereby endowing the materials with properties distinct from those of stoichiometric crystals. From a theoretical perspective, the absence of extrinsic elemental doping ensures that all observed property changes originate solely from intrinsic variations at the A- or B-sites.This reduction in extrinsic variables simplifies the mechanistic analysis, enabling more targeted conclusions and offering a streamlined model system for theoretical investigation.

#### 3.2.1 A-Site Deficiency

It is well recognized that barium tends to evaporate during high-temperature sintering (>1350 °C) in most barium-based electrolytes, such as BZY20 and BZCYYb1711, which adversely affects both the density and ionic conductivity.^52,260^ Interestingly, introducing a slight Ba deficiency into the epitaxial 20% Y-doped BaZrO_3−δ_ (20Y–BZO) thin film prepared by PLD has been reported to enhance proton conductivity.^260^ As shown in the high-angle annular dark field scanning transmission electron microscopy (HAADF-STEM) images and APT maps (Figure 23a–e), high-temperature annealing treatment generates a nonstoichiometric Ba-deficient Y-rich secondary phase and results in the major phase having a slight Ba deficiency. Through ab initio modeling analysis of the stoichiometric phase, the major phase, and the secondary phase, it was found that the major phase with a slight Ba deficiency exhibits the lowest lattice distortion. This reduces the proton trapping effect, thereby lowering the activation energy for proton transport. Meanwhile, the slight Ba loss in the annealed sample is accompanied by the generation of oxygen vacancies, thereby increasing the carrier concentration.^273^ These factors enable the annealed 20Y–BZO film to have higher protonic conductivity than the stoichiometric sample. In addition, Mather et al.^274^ have reported that introducing 5% Ba deficiency into BCY20 can effectively increase the proton transport. Ba_0.95_Ce_0.8_Y_0.2_O_3−δ_ exhibited greater cell volume and monoclinic distortion than BCY20 due to the A-site vacancy mechanism, suggesting the formation of positive charge carriers.

**Figure 23:**
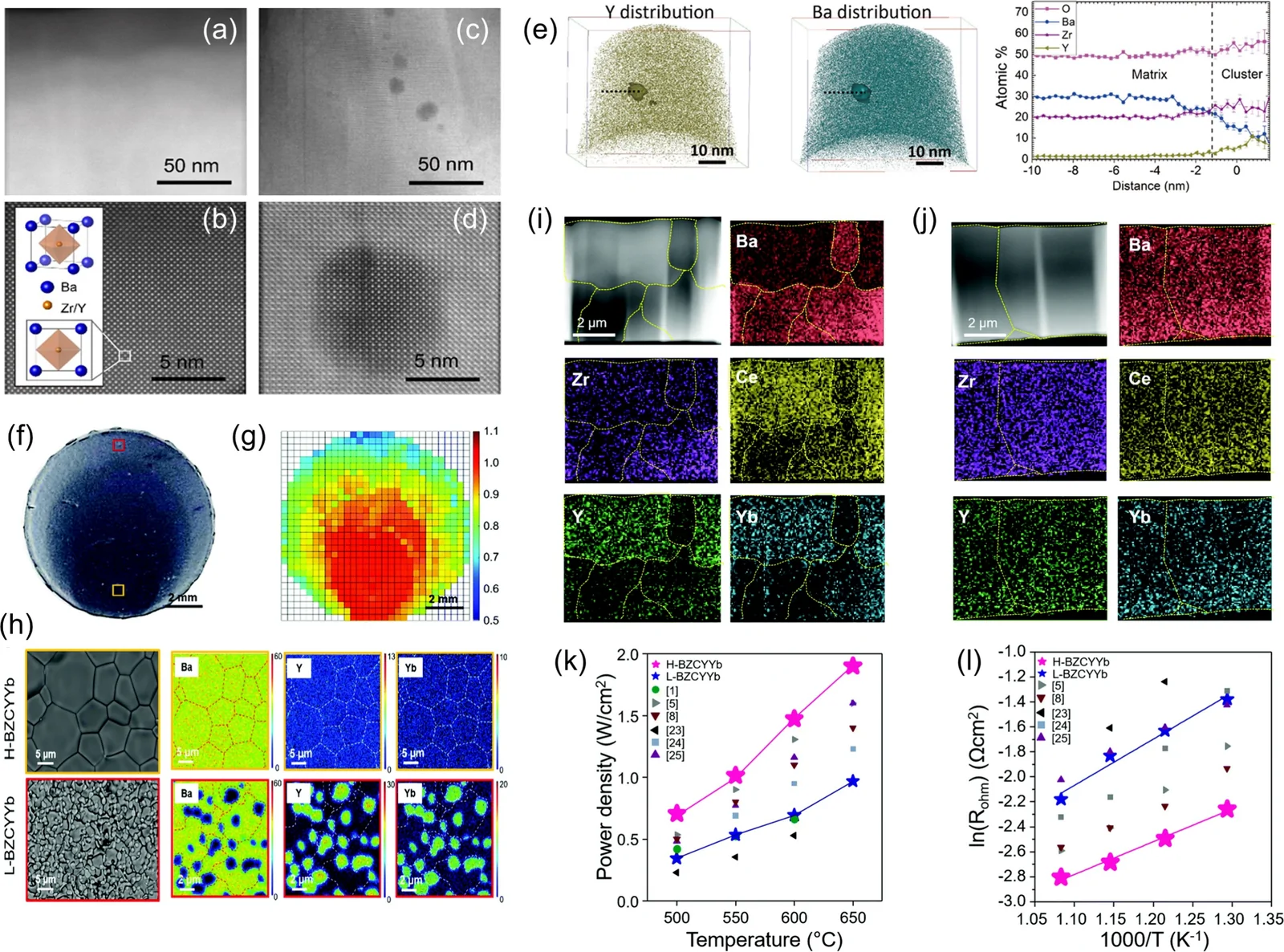
HAADF-STEM images of the (a, b) virgin and (c, d) annealed 20Y-BZO films. (e) APT analysis of the annealed 20Y–BZO sample. Reproduced from ref 273. Copyright 2018 American Chemical Society. (f) An optical image of the BZCYYb4411 pellet sintered at 1500 °C for 5 h. (g) Ba element distribution of the pellet in (f) from the rich to deficient region, indicated by red and blue colors, respectively. (h) SEM and FE-EPMA mapping results at the point with the highest concentration of Ba (H-BZCYYb, the yellow square in (f)) and the lowest concentration of Ba (L-BZCYYb, the red square in (f)). STEM-EDS maps of cation distribution in the thickness direction for (i) L-BZCYYb and (j) H-BZCYYb. Electrochemical performance of two BZCYYb4411 electrolyte-based single cells. Comparison of (k) the peak power densities and (l) the area-specific ohmic resistances with the previously reported PCFCs. Reproduced with permission from ref 80. Copyright 2021 RSC.

However, excessive barium loss leads to a decrease in ionic conductivity, because the substitution of B-site ions reduces oxygen vacancies in the lattice and then hinders the proton migration.^273^ To compensate for Ba evaporation in BaZr_0.4_Ce_0.4_Y_0.1_Yb_0.1_O_3−δ_ electrolyte (noted by L-BZCYYb), Choi et al.^80^ introduced an external Ba source, resulting in a denser electrolyte (noted by H-BZCYYb) with enlarged grains and improved elemental homogeneity. The optical image (Figure 23f,g) shows the different densification of different locations on the electrolyte surface after sintering at 1500 °C for 5 h. The supplementary Ba sources were able to suppress Ba evaporation during the sintering process, leading to reduced surface coverage of secondary clusters caused by the accumulation of B-site cations and the formation of larger grains, as confirmed by the SEM images and field emission electron probe microanalysis (FE-EPMA) in Figure 23h. The STEM-EDS image (Figure 23i,j) further shows the obvious elements distribution difference of L-BZCYYb and H-BZCYYb electrolyte in the thickness direction, indicating that A-site Ba deficiency is accompanied by B-site dopants (Ce, Y, Yb) segregation in perovskite oxides. The increased R_gb_, resulting from small grain size and secondary clusters caused by element segregation, deteriorates the physical and chemical properties of the electrolyte, thereby reducing proton conductivity.^80^ In this case, the electrolyte with an added external Ba source exhibited higher proton conductivity and lower impedance. A single cell composed of this electrolyte demonstrates excellent electrochemical performance (Figure 23k,l), achieving a PPD of 1.90 W cm^–2^ and 1.01 W cm^–2^ at 650 and 550 °C, respectively.

#### 3.2.2 B-Site Deficiency

Due to the diverse nature of B-site cations in proton-conducting electrolytes, the study of B-site cation deficiency is generally carried out by examining the nonstoichiometric across all B-site elements. In a specific example, a 5% B-site cation deficiency in BZCYYb1711 reduces the sintering temperature to 1350 °C, whereas a stoichiometric electrolyte requires a sintering temperature of 1500 °C.^275^ This deficiency enables the material to achieve a relative density of 96.5% and larger grain sizes, thereby significantly decreasing R_gb_. Electrochemical measurements reveal that the nonstoichiometric electrolyte exhibits a 2.5-fold higher proton conductivity (0.042 S cm^–1^ at 700 °C) compared to the pristine electrolyte. The single cell with a nonstoichiometric electrolyte achieves a PPD of 794 mW cm^–2^ at 650 °C, a 23.5% improvement over the stoichiometric cell, and maintains stable performance for 300 h at 550 °C.^275^ Based on the previous discussion, for deficiency regulation, further exploration is needed to quantitatively control deficiency concentration and conduct precise quantitative mechanism studies.

### 3.3 Interfacial Engineering

In P-RSOCs, the electrochemical–mechanical coupling effect at the electrode–electrolyte interface directly affects the microstructural stability, mechanical integrity, electrochemical performance, and overall durability of ceramic electrochemical cells. Charge-transfer processes generally involve oxygen species, protons, and electrons, requiring reactions at the TPB between the electrolyte and the air electrodes.^52,276,277^ For example, at the electrolyte–air electrode interface in a fuel cell mode, oxygen molecules adsorb, dissociate, and transport on the surface of the air electrode and react with electrons from the external circuit to form partially reduced oxygen species and ultimately oxide ions. Each of these surface processes is governed by a combination of vacancy-induced bulk diffusion and surface exchange.^276^ The pore structure of the electrode primarily determines gas transport efficiency, while the quality of the electrolyte-electrode contact affects the formation and conduction of charge carriers at the TPB.^278,279^ As a result, the interfacial resistance remains a critical bottleneck, significantly limiting the single-cell performance. Numerous studies report that the measured ohmic area-specific resistances in high-temperature cofired, anode-supported half-cells exceed theoretical predictions.^38,280^

Beyond the inherent properties of the electrolyte, interfacial characteristics strongly impact the P-RSOC performance. For example, high-temperature sintering can produce secondary phases on the electrolyte surface that are detrimental to proton conductivity.^36,281^ Furthermore, delamination and cracks caused by an interfacial bonding mismatch severely compromise long-term stability.^162^

To mitigate these challenges, various interfacial engineering strategies have been developed to tailor the electrolyte-electrode interface. Two primary approaches have emerged for modifying proton-conducting electrolytes: (1) morphological control of the interface and (2) the introduction of functional interlayers.^52,246,282^ The former approach removes surface impurities on the electrolyte to enhance interfacial bonding through physical or chemical treatments, whereas the latter facilitates interfacial proton conduction by incorporating a functional interlayer.

#### 3.3.1 Interface Morphology Manipulation

Interface morphology regulation eliminates the surface secondary phase—primarily arising from cation segregation during high-temperature sintering—through physical or chemical methods, thereby increasing the interface contact area and enhancing the bonding between the electrolyte and electrodes. Li et al.^283^ removed the defective layer (approximately 1–2 μm) on the BaZr_0.1_Ce_0.7_Y_0.2_O_3−δ_ surface through mechanical polishing, effectively improving interfacial contact and proton conduction capability. After polishing, the performance of the single fuel cell increased by approximately 50%, and that of the electrolysis cell increased by approximately 120% at 650 °C.^283^ However, this physical polishing method may cause damage to the electrolyte layer, resulting in gas leakage. A chemical method through acid etching has also been used to reconstruct the surface morphology of the BZCYYb1711 electrolyte.^284^ As shown in Figure 24a–d, the etching treatment removes surface segregation and creates a rough, reactive interface that promotes strong chemical bonding with the air electrode. Peeling strength tests (Figure 24e) show that the acid-treated cell (10 min treatment) achieves a maximum peeling strength of 23.5 N, compared with 18.6 N for the untreated sample, consistent with the observed surface roughness results. The enhanced mechanical integrity of the electrolyte-electrode interface is further validated by stability tests. Under constant operation at 1.4 V and 600 °C, the acid-treated cell exhibits a current decay of less than 1% over 0–100 h, with no interfacial delamination observed after testing. In contrast, the untreated cell shows a current decay of 10.2% over the same period, accompanied by severe electrolyte-electrode delamination.

**Figure 24:**
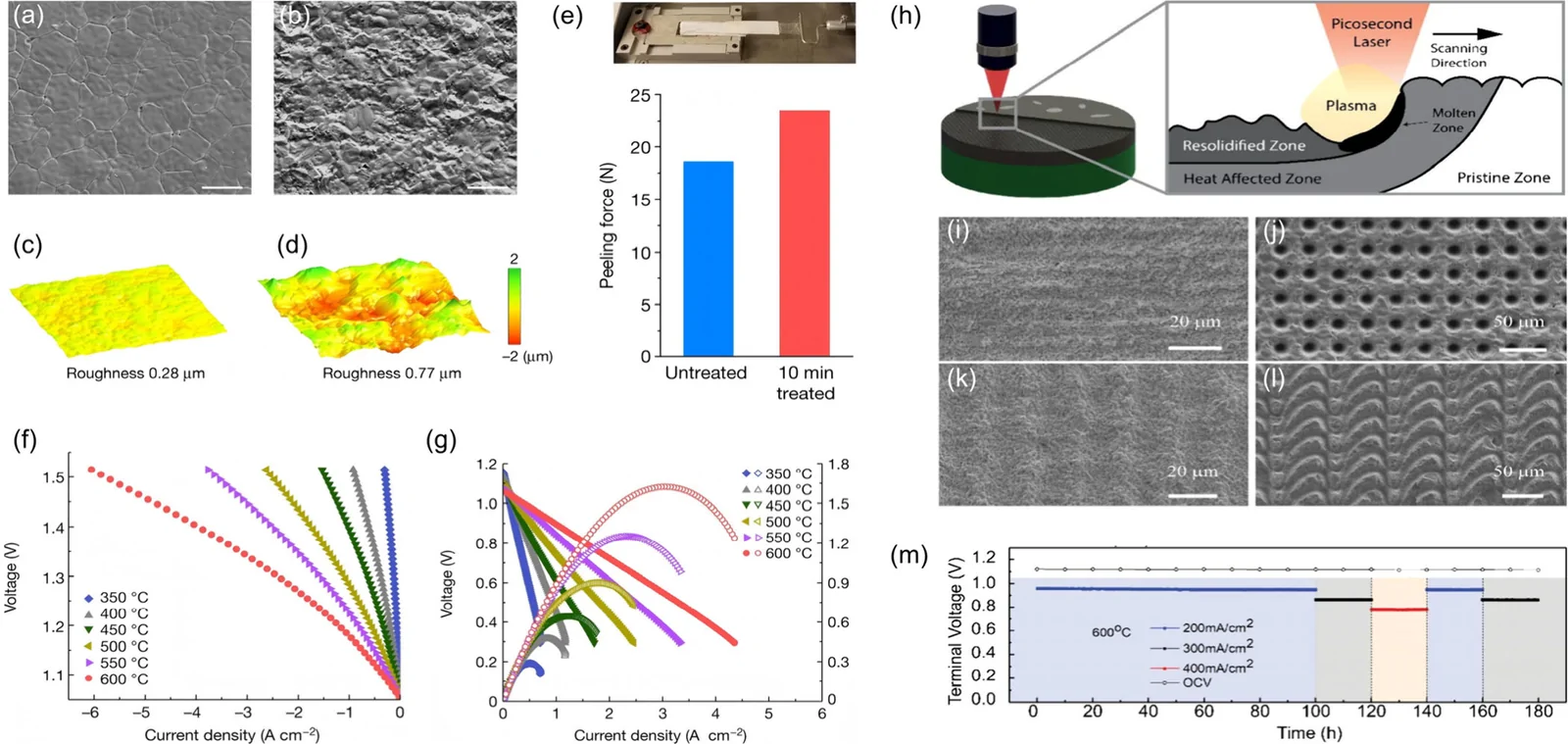
Microstructure for (a) untreated and (b) 10 min acid-treated electrolyte surface. Scale bars, 20 μm. Atomic Force Microscope images for the (c) untreated and (d) 10 min acid-treated electrolyte surface. (e) Peeling strength of cathode-electrolyte interface (lower panel). The upper panel shows a photograph of the peeling strength measurement. I–V curves in (f) PCEC operations, and I–V curves and power density curves in (g) PCFC operations of cells made with the 10 min acid-treatment electrolyte. Reproduced with permission from ref 284. Copyright 2022 Springer Nature. (h) Schematic illustration of the laser ablating process and mechanism. (i) SEM micrograph of the uniformly laser-polished surface. (j–l) SEM micrographs of (j) holes, (k) cross, and (l) S-shape pattern surfaces fabricated by laser ablating process. (m) Stability test of the cross-patterned single cell at various current densities and 600 °C. Reproduced from ref 285. Copyright 2024 American Chemical Society.

Moreover, acid treatment increases the effective contact area between the electrolyte and the oxygen electrode without altering the electrode reaction mechanism or the proton conduction mechanism. This is evident by the unchanged activation energy before and after the treatment. Correspondingly, the proton conductivity—derived from the R_o_ of the cell determined from EIS measurements—increases from 0.0057 S cm^–1^ to 0.013 S cm^–1^ at 500 °C, fully recovering the intrinsic conductivity of the BZCYYb1711 electrolyte.^284^ These results indicate an increased density of active reaction sites at the electrolyte-electrode interface, enabling the intrinsic bulk proton conductivity to be fully utilized. The acid-treated cells exhibit remarkable performance improvements shown in Figure 24f,g. At 600 °C, the PPD in PCFC mode reaches 1.6 W cm^–2^, and in PCEC mode, the current density exceeds −3.9 A cm^–2^ at 1.4 V. Furthermore, this interfacial engineering strategy achieves superior performance at low temperatures of 350 °C with 290 mW cm^–2^, which highlights the critical role of interfacial engineering in all-solid-state electrochemical devices.^284^

Despite these advances, traditional physical or chemical methods face issues such as poor reproducibility, complicated processes, and material applicability, which limit their large-scale application. Zhou et al.^285^ developed a precise laser ablating technique capable of regulating the interface of electrolytes and cathodes. By controlling the laser scanning line spacing and movement path, electrolyte surfaces with different microrough morphologies can be fabricated (Figure 24h–l). The increased microroughness significantly enhances the peeling strength of the cathode on the electrolyte surface, indicating a more stable TPB, which is expected to facilitate charge transfer. Correspondingly, the apparent electrolyte conductivity of the cross-patterned sample at 550 °C is approximately 2.5 times higher than that of the pristine sample. Higher performance in the laser cross-patterned cell was achieved with a PPD of 1401 mW cm^–2^ at 650 °C, and it operated steadily at varying current densities (200 mA cm^–2^ to 400 mA cm^–2^) for over 180 h at 600 °C, as shown in Figure 24m.^285^

#### 3.3.2 Functional Layer

Efficient proton transport from the fuel electrode to the air electrode via the electrolyte is critical for enhancing interfacial reactivity. Inserting a functional layer at the electrolyte-fuel electrode interface to improve proton conduction pathways at the fuel electrode can accelerate proton transfer, thereby reducing overall resistance and enhancing the electrochemical performance of electrochemical cells.^280^ Chen et al.^286^ systematically studied the effect of anode functional layer (AFL) prepared by spin-coating on the electrode–electrolyte interface. Spin-coated AFL reduces the surface porosity of the electrolyte layer, enhances interfacial adhesion, and decreases R_o_ by improving proton conduction pathways. The optimized cell achieves a PPD of 2345 mW cm^–2^ at 700 °C in fuel cell mode and a high electrolysis current density of −3.0 A cm^–2^ at 1.3 V in electrolysis mode.^286^ On the other hand, to achieve long-term stability in cell operation, the conventional single electrolyte layer can be replaced with a double electrolyte layer, thereby reducing the degradation of high-proton-conductive electrolyte materials. Kane et al.^28^ introduce a bilayer electrolyte design for P-RSOCs, combining a highly conductive BZCYYb1711 backbone with a thin, protective BaHf_0.8_Yb_0.2_O_3−δ_ layer deposited via cosputtering. Importantly, the thin BHYb82 layer adds negligible R_o_ while maintaining high proton conductivity. The bilayer electrolyte demonstrates excellent chemical stability when exposed to humid CO_2_. It degrades at a rate of 1.1% per 1000 h, significantly lower than unmodified BZCYYb1711, with a degradation rate of 7.0% per 1000 h in a symmetrical cell test. Despite the addition of a protective layer, the electrochemical performance of a single cell remains outstanding, achieving a PPD of 1.22 W cm^–2^ in fuel cell mode and −1.86 A cm^–2^ at 1.3 V in electrolysis mode at 600 °C. Additionally, excellent reversibility is demonstrated for 200 h at a current density of 0.5 A cm^–2^, switching between modes every 2 h.^28^

### 3.4 Microstructural Engineering

Microstructural engineering has emerged as a pivotal strategy for translating the intrinsic transport properties of proton-conducting electrolytes into practical electrochemical performance. While many proton-conducting materials exhibit high bulk proton mobility, their macroscopic conductivity and device-level performance are often governed by microstructural features introduced during powder synthesis, densification, and cell fabrication. These features—including grain size, grain connectivity, interfacial chemistry, secondary phases, and residual porosity—define the actual transport pathways available to protons and frequently dominate the overall resistance.

Among these factors, interfaces within polycrystalline electrolytes play a particularly critical role, as they are prone to structural and chemical heterogeneity, defect redistribution, impurity segregation, and unintended electronic leakage. As a result, materials with comparable bulk defect chemistry may display proton conductivities that differ by more than an order of magnitude, largely reflecting differences in the processing history rather than intrinsic lattice properties.

Using Y-doped barium zirconate as a representative example, conventionally sintered pellets typically deliver total proton conductivities of only ∼ 10^–3^ S cm^–1^ at 600 °C, despite favorable bulk proton transport.^287^ This limitation arises from the combined effects of poor sinterability, residual porosity, and microstructure-controlled transport bottlenecks that develop during high-temperature processing. Attempts to improve densification by increasing firing temperature are often counterproductive, as Ba volatilization and secondary-phase formation introduce additional microstructural and chemical heterogeneities that further suppress proton transport.^288^

To overcome these constraints, a range of microstructural engineering strategies have been developed to deliberately tailor grain morphology, interfacial chemistry, and transport pathways. These approaches broadly encompass (i) the use of functional sintering additives to promote densification and grain growth,^289^ (ii) dopant-assisted strategies that modify both lattice and interfacial defect chemistry,^81^ (iii) control over particle size and size distribution to regulate grain boundary density,^290^ and (iv) system-level process engineering routes that reshape microstructure at the device scale.^291^ Collectively, these strategies provide complementary and often synergistic pathways to minimize microstructure-induced resistance and unlock the intrinsic proton conductivity of state-of-the-art electrolytes.

#### 3.4.1 Functional Sintering Additives

NiO serves as an exceptionally effective sintering aid for acceptor-doped BZCYYb1711. With the incorporation of merely ∼ 1 wt % NiO, the pellets can achieve ∼ 96.5% of their theoretical density after sintering at 1350 °C for 3 h—representing a reduction of approximately 200 °C in sintering temperature and 7 h in holding time compared to the temperature–time conditions required for comparable densification without NiO.^292^ This pronounced improvement is rooted in grain boundary engineering. During heating, NiO reacts with the perovskite matrix to generate a thin BaY_2_NiO_5_ layer along grain boundaries; the latter creates a transient liquid film that accelerates ion diffusion and thus promotes rapid grain growth and pore elimination (average grain size 3.9 μm at 1350 °C with NiO versus 0.6–0.7 μm for the undoped sample). On cooling, the BaY_2_NiO_5_ partially decomposes, leaving only traces at the interfaces and preserving the bulk perovskite chemistry; as a result, the final microstructure combines large, tightly packed grains with a markedly reduced grain boundary volume fraction. Importantly, this grain boundary modification does not compromise the electrolyte’s functional properties: the NiO-assisted specimens maintain the high protonic conductivity characteristic of dense BZCYYb while offering a processing window more than 200 °C lower than the conventional route.

Beyond Y-doping, the formation—and subsequent decomposition—of the liquid BaLn_2_NiO_5_ sintering phase is common to virtually all lanthanide additives. Wang et al.,^293^ therefore, compared Nd and Y in BaCe_0.8_Y_0.2–x_Nd_x_O_3−δ_ to clarify their relative merits. After sintering at 1400 °C, the average grain size increased sharply from 1.2 μm (x = 0) to 3.7 μm (x = 0.15). Although this coarser microstructure suggests enhanced mass transport, the electrochemical data reveal that conductivity does not rise monotonically with grain growth. Once the Nd content exceeds x = 0.05 (BCYN10, BCYN15), σ_gb_ falls, and R_gb_ climbs, signaling an adverse shift in grain boundary chemistry. To reconcile these effects, the authors invoked an Nd–Y synergistic codoping strategy: at x = 0.05 (BCYN5), the σ_gb_ peaks, while both bulk resistance (R_b_) and R_gb_ reach their minima, yielding the best overall performance. This analysis underscores that, for BaCeO_3−δ_-based proton conductors, judicious control of grain boundary composition—not merely grain size—is decisive for optimizing transport properties.

Moreover, Belyakov et al.^294^ showed that adding 2 or 4 mol % ZnO to SrHf_0.8_Sc_0.2_O_3−δ_ successfully contributes to densification at 1450 °C, allowing the sintering temperature to be lowered by 200 °C while still delivering a high relative density (92% for Zn2 and 94% for Zn4, versus 95% for the Zn-free specimen). Critically, impedance spectroscopy fitting confirmed that the ZnO sintering additive hardly affects σ_bulk_ and σ_gb_. The effective activation energies were 0.64–0.67 eV for σ_bulk_ and 0.72–0.77 eV for σ_gb_, which remained nearly identical to those of pristine SrHf_0.8_Sc_0.2_O_3−δ_. Consistent with this, the total conductivities measured in dry or wet atmospheres—as well as the pO_2_-dependent proton, oxide-ion, and hole contributions—were almost unchanged after ZnO addition, confirming that grain boundaries are not electrically penalized by the aid. Microstructural and compositional analyses reinforce the electrochemical results: XRD and EDS revealed that ZnO is partially (≤0.3 mol %) incorporated into the perovskite lattice and partially accumulates at grain boundaries and surface, from where it gradually evaporates during annealing, leaving no secondary phases. The transient SrO–ZnO liquid that forms at boundaries therefore enhances mass transport during sintering yet volatilizes on cooling, preserving the original grain boundary chemistry and conduction pathways.

Another common sintering aid is CuO. Starostin and co-workers systematically added CuO (0–2 wt %) to BaSn_0.8_Y_0.2_O_3−δ_ and fired the pellets at 1500 °C.^295^ X-ray diffraction showed neither peak shifts nor secondary phases, confirming that the perovskite lattice remained intact after CuO addition. ICP-MS revealed that ≥ 90% of the nominal CuO volatilized during sintering, leaving only trace amounts in the ceramic. Despite this loss, a pronounced liquid-phase-sintering behavior emerged: the average grain size first increased and then fell with CuO content, reaching a maximum of ∼ 4 μm at x = 0.5 wt %—the composition that also gave the most compact bulk microstructure. This trend, together with the unusually low solidus temperature of the BaO–CuO system (∼850–920 °C), confirms that a low-melting Ba–Cu–O liquid forms early in the firing cycle, accelerates mass transport, and promotes densification. After cooling, most of the residual Cu segregates to energetically favorable sites—predominantly grain boundaries—because its solubility in the BaSnO_3−δ_ lattice is negligible. SEM and electron backscatter diffraction mapping detect nanoscale CuO/Cu_2_O particles decorating selected grain boundaries, whereas others remain clean, giving two distinct boundary populations.^296^ EIS analyzed via distribution-of-relaxation-times (DRT) separates these responses: boundaries covered by a continuous CuO layer exhibit suppressed proton transport, manifesting as a new midfrequency, rate-limiting process with higher activation energy, while clean boundaries remain highly conductive. Consequently, although trace CuO is sufficient to facilitate liquid-phase sintering, any uncontrolled enrichment at grain boundaries degrades σ_gb_ and raises the overall activation energy. Further optimization, therefore, hinges on minimizing CuO retention or ensuring its homogeneous, discontinuous distribution to preserve the beneficial densification while avoiding a continuous blocking phase at the grain boundaries.

Using BaCe_0.3_Zr_0.55_Y_0.15_O_3−δ_ as a model electrolyte, Nasani et al.^289^ systematically compared 1 wt % NiO, ZnO, and CuO additives. All three aids produced highly dense pellets (>95% of theoretical density) after a single 8 h dwell at 1400 °C, whereas the pristine powder required 1600 °C. ZnO was particularly effective, lowering the peak shrinkage temperature to ∼ 1300 °C and generating the largest linear shrinkage (∼14%). Although the σ_bulk_ of every doped sample fell by roughly half an order of magnitude—an effect attributed to limited solid solubility and proton trapping —their grain boundary responses diverged sharply. Brick-layer analysis revealed that only the ZnO-doped specimen displayed an enhanced specific σ_gb_, exceeding the undoped value across N_2_, H_2,_ and O_2_ atmospheres. Under humid N_2_, the activation energy for grain boundary transport fell to 0.52–0.53 eV with ZnO, compared with 0.70–0.80 eV for the NiO- and CuO-containing ceramics. Space-charge modeling showed that ZnO lowers the Schottky barrier height at 300 °C far more effectively than NiO or CuO, thereby minimizing carrier depletion in the space-charge layer and accounting for the superior σ_gb_. This behavior is consistent with reports that Zn^2+^ segregates preferentially to grain boundaries—rather than dissolving into the perovskite lattice—where it compensates the positively charged core and mitigates proton repulsion. Density-functional calculations by Lindman et al.^297^ provide a complementary picture. For structurally analogous Σ5 tilt boundaries (a low-Σ coincidence site lattice grain boundary), the proton segregation energy in BaZrO_3−δ_ is ∼ −0.8 eV, whereas in BaCeO_3−δ_ it is only −0.4 eV. The deeper well in BaZrO_3−δ_ drives stronger proton accumulation at the core, generating a larger space-charge potential that severely blocks cross-boundary migration. Because the local hydrogen-bond geometry inside the boundary is comparable in both oxides, the difference stems from the higher intrinsic stability of protons in orthorhombic BaCeO_3−δ_ bulk, not from the boundary structure itself.

Kindelmann et al.^298^ creatively developed a water-mediated cold-sintering route for BaZr_0.7_Ce_0.2_Y_0.1_O_3−δ_. The powder is first compacted at 350 °C under 400 MPa for 5 min using only deionized water as the transient sintering aid; a subsequent 10 h anneal at 1300 °C fully reconstructs the perovskite phase and achieves ∼ 96% relative density. By avoiding the high temperatures (>1500 °C) required for solid-state reactive sintering (SSRS), this protocol suppresses the formation of the detrimental BaY_2_NiO_5_ liquid and yields nanocrystalline microstructures (average grain size <400 nm) with “clean,” dopant-rich grain boundaries. High-resolution STEM-EDS and three-dimensional APT maps show that these boundaries are razor-thin (∼0.6 nm) and display a pronounced segregation of Y and Ni within a ∼ 3 nm zone, accompanied by slight Zr/Ce depletion; in the SSRS sample, by contrast, a ∼ 5 nm Ni-rich interlayer and broad depletion of all other cations are observed (boundary thickness ∼ 2.1 nm). The enriched acceptor dopants neutralize the positive grain boundary core, diminishing the space-charge layer barrier and lowering the activation energy for proton transport. Despite its finer grains, the cold-sintered material exhibits grain boundary conductivities that exceed those of the coarse-grained SSRS reference in every tested atmosphere (wet air, Ar, and H_2_), so the total conductivity is no longer grain-boundary-limited. An H_2_/D_2_ isotope shift confirms that conduction remains proton-dominated; at 600 °C, the total conductivity sits at the top end of all literature values for Zr-rich BZCY721 processed below 1500 °C. Collectively, these results demonstrate that water-assisted cold sintering, followed by moderate annealing, provides an effective grain-boundary-engineering strategy that both lowers the processing window by ∼ 300 °C and enhances the proton conductivity of the grain boundary.

Systematic studies have already established a clear understanding of the sintering behavior of various elemental additives. Recent comparative studies have clarified how NiO-assisted sintering and A-site chemistry shape the grain boundary landscape—and thereby the transport properties—of Ba-based proton conductors. Chen et al.^292^ densified BZY20 and BaZr_0.1_Ce_0.7_Y_0.2_O_3−δ_ with 2 mol % NiO at 1400 °C. The microstructures differ strikingly: BZY20–2%NiO retains fine grains (∼1.2 μm) embedded in a network of thick grain boundaries, whereas BZCY172–2%NiO develops larger grains (∼3.5 μm) with far fewer interfaces. R_gb_ therefore dominates in BZY20–2%NiO, raising its activation energy to 0.58–0.63 eV and limiting the total conductivity in wet air at 700 °C to 0.014 S cm^–1^; BZCY172–2%NiO, in contrast, shows 0.46–0.47 eV and 0.025 S cm^–1^ under identical conditions. The same trend controls device performance: protonic ceramic fuel cells give 360 mW cm^–2^ for BZY20 and 855 mW cm^–2^ for BZCY172 at 700 °C, widening to 30 mW cm^–2^ versus 150 mW cm^–2^ at 450 °C. Impedance fitting confirms that the difference in conductivity is mainly caused by the σ_gb_. Leonard et al.^299^ reached a complementary conclusion for A­(Zr_5/9_Ce_4/9_)_1–x_Y_x_O_3−δ_ (A = Ba, Sr) electrolytes, that the grain boundary response is the main source of resistance in the Ba­(Zr_5/9_Ce_4/9_)_1–x_Y_x_O_3−δ_ specimens, whereas R_b_ dominates in the strontium analog. Their deconvoluted spectra show that exchanging Sr^2+^ for the more basic Ba^2+^ suppresses boundary resistance and lifts the proton conductivity of Ba­(Zr_5/9_Ce_4/9_)_0.8_Y_0.2_O_3−δ_ to 1.44 × 10^–2^ S cm^–1^ at 600 °C (wet H_2_), almost an order of magnitude above Sr­(Zr_5/9_Ce_4/9_)_0.8_Y_0.2_O_3−δ_. Collectively, these results demonstrate that (i) coarse grains with clean, Ni-assisted boundaries in Ce-doped BZY20 facilitate proton transport, while the finer BZY20 matrix is bottlenecked by resistive interfaces; (ii) grain boundary, not bulk, activation energy governs the overall conductivity in BZY20; and (iii) combining Ba-rich A-site chemistry with judicious sintering-aid selection is essential for mitigating boundary resistance and unlocking high power density in protonic ceramic devices.

#### 3.4.2 Dopant-Assisted Strategy

Introducing the sintering-aid element directly into the B-site lattice is emerging as a more practical route than the conventional “case-by-case” external addition strategy. Wang et al.^300^ compared these two approaches for BaZr_0.4_Ce_0.4_Y_0.2_O_3−δ_ (BZCY442). After sintering at 1350 °C for 5 h, 5 mol % Zn-doped BZCY442 (BZCYZn) crystallized as a single-phase perovskite with a relative density of 98%, whereas the sample produced by externally adding 1 wt % ZnO (BZCY–ZnO) showed Y_2_O_3_ segregation and reached only 94% density. Internal Zn^2+^ substitution (partly for Y^3+^ at the B-site) introduces additional oxygen vacancies owing to its larger ionic radius (74 pm) and lower valence, thereby enhancing ionic transport—particularly across grain boundaries. Consistently, BZCYZn displays a larger average grain size (∼3.84 μm vs 1.29 μm for BZCY–ZnO) ZnO) and a lower grain boundary induced space-charge potential barrier. In contrast, externally added ZnO reacts with BaO to form the low-melting BaO–ZnO eutectic (1098 °C), which preferentially forms a transient liquid phase along the grain boundaries, markedly accelerating atomic diffusion and neck growth, yet even 1 wt % ZnO noticeably disturbs the Ba stoichiometry and triggers Y_2_O_3_ precipitation at the boundaries. A parallel study by Wang et al.^301^ on BaZr_0.1_Ce_0.7_Y_0.2_O_3−δ_ confirms the advantage of internal doping. Replacing 4 mol % Ce^4+^ with Ni^2+^ yields a dense electrolyte at 1400 °C—200 °C lower than the 1600 °C needed for pristine BZCY172—and produces larger grains, fewer boundaries, and a more uniform elemental distribution than a companion specimen obtained by mixing 4 mol % NiO as an external sintering aid. The BaZr_0.1_Ce_0.66_Ni_0.04_Y_0.2_O_3−δ_ sample shows a high density and large grain size after sintering at a relatively low temperature (1400 °C), but higher conductivity than BaZr_0.1_Ce_0.7_Y_0.2_O_3−δ_ sintered at 1600 °C. Under fuel cell conditions, the internally doped membrane attains σ = 6.3 × 10^–3^ S cm^–1^ at 600 °C, outperforming both the externally aided counterpart and the high-temperature-sintered pristine sample. It should be mentioned that Ni^2+^ is a transition-metal ion with finite electronic conductivity, and excessive substitution may promote mixed proton–electron transport and thus diminish the electrolyte’s electronic blocking capability. Moreover, in reducing atmospheres (e.g., the anode side under H_2_), Ni can catalyze side reactions or be reduced to Ni^0^, migrate, and precipitate, posing long-term stability risks. These considerations underscore the need to balance densification benefits with potential electronic leakage and catalytic side effects when selecting transition-metal dopants for grain boundary engineering.

Wang et al.^302^ explored an extreme case in which the electrolyte is left entirely unsintered. In their study, the unsintered BCY20 was tested between 600 and 800 °C. AC-impedance spectroscopy showed a significantly higher GB impedance for unsintered BCY20— ∼ 8.9 Ω cm^2^ at 600 °C—more than five times that of a fully sintered BCY20 pellet (∼1.5 Ω cm^2^). SEM micrographs confirmed a high density of grain boundaries together with nonuniform particle packing, in stark contrast to the dense microstructure of the sintered sample. Despite those microstructural drawbacks, unsintered BCY20 retains a remarkably high proton-defect concentration (up to 1.99 × 10^–1^ mol mol^–1^) and an outstanding hydration equilibrium constant, K_W_ = 1.00 × 10^–3^ Pa^–1^ at 600 °C, both of which surpass most reported proton conductors. Nevertheless, its total conductivity is roughly a factor of 5 lower than that of sintered BCY20: 8.90 × 10^–3^ S cm^–1^ versus ∼ 4.06 × 10^–2^ S cm^–1^ at 800 °C (pH_2_O = 618 Pa; pO_2_ ≈ 2.0 × 10^4^ Pa). Taken together, these results underscore that high-temperature sintering—which eliminates porosity, reduces grain boundary density, and thereby suppresses R_gb_—is indispensable for unlocking the full grain boundary contribution to proton transport and maximizing the overall electrochemical performance of BaCeO_3−δ_-based electrolytes.

#### 3.4.3 Particle Size and Distribution Control

Attempts to tailor precursor powders have concentrated on shrinking the primary particle size before sintering so that rapid densification and exaggerated grain growth are triggered later, diluting the population of grain boundaries and, with it, the resistive barrier they impose. In place of the classical SSR, powder routes such as spray-drying, spray pyrolysis, and—most successfully—sol–gel, wet-chemical, or microwave-hydrothermal syntheses have been adopted. Yamazaki et al.^287^ prepared nanocrystalline precursor powders by a polymeric sol–gel route. When the sintered grain size was enlarged from 0.44 to 1.00 μm, the total conductivity at 450 °C increased by a factor of 2.5–3.2, whereas the grain-interior conductivity was essentially unchanged, confirming that the gain originated entirely from reduced R_gb_. A total conductivity of ∼ 1 × 10^–2^ S cm^–1^ was already achieved at 450 °C. Calcining at only 950 °C deliberately left a small amount of BaCO_3_ in the precursor; during the subsequent reactive sinter, this carbonate reacts with the Ba-deficient perovskite, drives abnormal grain growth, and yields a dense, coarse-grained microstructure. Using a hybrid wet-chemical/SSR route, Zhong et al.^303^ obtained phase-pure BZCYYb powders at 950 °C in 3 h (vs 1200 °C, 24–36 h for conventional SSR). Electrolytes derived from these powders reached 96.3% relative density after sintering at 1450 °C, an SSR control required 1550 °C to attain comparable densification and still exhibited finer grains (∼2 μm vs ∼ 3 μm). The coarser-grained modified wet-chemical/SSR pellet delivered 2.6 × 10^–2^ S cm^–1^ at 700 °C in wet air, together with a lower space-charge potential barrier and a higher grain-to-grain contact factor w/d_g_—(defined as the grain-to-grain contact width w normalized by the average grain size d_g_ of the sintered pellets), unequivocally evidencing superior grain boundary conduction. Because no foreign sintering aid is added, the process avoids second-phase formation and preserves the intrinsic transport properties of the electrolyte. Balasundari et al.^304^ synthesized single-phase monoclinic LaNbO_4_ at only 800 °C—nearly 200 °C below the temperature needed for coprecipitated powders. The microwave hydrothermal powders, composed of uniform nanocrystals, sintered to 97% theoretical density at 1300 °C while retaining relatively large grains (∼0.7–1.5 μm). The reduced GB area yielded a proton conductivity of 5.23 × 10^–4^ S cm^–1^ at 700 °C, more than 5-fold higher than pristine LaNbO_4_ (9.5 × 10^–5^ S cm^–1^), underscoring the critical role of grain boundary engineering.

Recent advances in nonconventional sintering have delivered substantial progress in tailoring grain-boundary structures and, in turn, proton transport in perovskite electrolytes. Singh et al.^305^ produced BaZr_0.85_Ho_0.1_Y_0.025_Nd_0.025_O_3−δ_ pellets that reached 99.8% of the theoretical density after only 20 min at 1500 °C by SPS. Impedance spectroscopy resolved a σ_gb_ of 4.22 mS cm^–1^ at 600 °C, markedly higher than that of conventionally sintered BZY20, confirming that the low-defect, high-contact-strength interfaces formed under the pulsed DC field effectively suppress Ba volatilization and secondary-phase precipitation, thereby minimizing grain boundary resistive barriers. Choi et al.^306^ eliminated Ba_5_Nb_4_O_15_ and Ba_3_NbMoO_8.5_ impurities that normally develop above 1100 °C in Ba_7_Nb_4_MoO_20−δ_ by applying 1350 °C × 5 min microwave sintering (MWS), achieving 98% relative density versus ∼ 83% for 1250 °C × 5 h conventional firing. A subsequent 1050 °C/12 h anneal raised the total conductivity to 1.3 mS cm^–1^ at 500 °C by simultaneously increasing σ_bulk_ and—critically—σ_gb_, whereas the parasitic phases possess negligible σ_gb_ and therefore act as proton blockers. Xu et al.^307^ densified BaCe_0.7_Zr_0.1_Y_0.2_O_3−δ_ at 1200 °C, where the microwave-sintered membrane is pore-free, shows larger grains (∼600 nm) than the conventionally treated counterpart (∼300 nm), and doubles the total conductivity (7.6 × 10^–3^ vs 4.0 × 10^–3^ S cm^–1^ at 600 °C) owing to a pronounced decrease in grain boundary resistance that is attributed to homogeneous cation distribution and suppressed Ba loss.

The ultrafast high-temperature sintering (UHS) protocol combines heating rates of 10^3^–10^4^ °C min^–1^ with isothermal holds of ∼ 10 s, arresting grain coarsening and delivering fine-grained, high-grain-boundary-density microstructures in a single step—without the tooling limitations of microwave, SPS, or flash sintering.^308^ Pan et al.^309^ applied UHS (1170 °C, 1 min) to a BZCY172–Li_6.4_La_3_Zr_1.4_Ta_0.6_O_12_ (LLZTO) composite. Partial melting of low-melting-point LLZTO coats BZCY172 grains, creating a continuous, “soft” grain boundary network that lowers the interfacial migration barrier. The resulting electrolyte is virtually pore-free and attains a proton conductivity of 0.028 S cm^–1^ at 600 °C—4.5 times that of pristine BZCY172. First-principles calculations and EIS confirm that LLZTO itself admits proton adsorption and transportation, so protons preferentially traverse the LLZTO-rich interfacial pathways, effectively turning the grain boundaries into high-speed conduction channels. Collectively, these techniques demonstrate that judicious control of grain boundary chemistry—whether by fast pulsed consolidation, selective microwave heating, or millisecond-scale radiative sintering—can convert the grain boundary from the principal resistive element into an active highway for proton conduction, paving the way for dense, low-temperature electrolytes with σ_gb_ comparable to σ_bulk_.

#### 3.4.4 System-Level Process Engineering

Pulsed-laser deposition has emerged as a versatile tool for grain boundary engineering in proton-conducting oxides, enabling architectures that either exploit or eliminate grain boundaries to boost electrochemical performance. Li et al.^310^ inserted an 8 nm GDC interlayer between a 75 nm BZY20 electrolyte and a porous Pt cathode in a micro-H-SOFC. The columnar PLD growth aligned GDC grains with those in BZY20, and the 0.42 nm vs 0.54 nm lattice mismatch generated a dense network of vertical dislocations and grain boundaries. These defective regions created fast pathways for oxygen incorporation and mixed-ionic (H^+^/O^2–^) conduction: the PPD rose from 206 to 445 mW cm^–2^ at 425 °C, while the midfrequency polarization resistance (oxygen adsorption–diffusion limited) dropped from 7.179 to 2.485 Ω cm^2^. The water-formation zone expanded from the Pt/BZY20 interface into the entire GDC layer, confirming that the purposely introduced grain boundaries served as an extended TPB. Pergolesi et al.^290^ grew 1 μm-thick, cube-on-cube epitaxial BZY20 films on (100)-MgO. The films were grain-boundary-free single crystals; XRD rocking curves (with a full-width at half-maximum of ∼ 0.3–0.35°) and reflection high-energy electron diffraction confirmed high crystalline quality. Under 5% H_2_/H_2_O, the proton conductivity reached 0.11 S cm^–1^ at 500 °C, 2 orders of magnitude higher than dense sintered pellets, with an activation energy of 0.63 eV. This unequivocally demonstrated that the sluggish macroscopic transport in conventional BZY20 originates from resistive grain boundaries, not from intrinsically low bulk mobility. Fluri et al.^311^ deposited 45–60 nm Ba_2_In_2_O_5_ films epitaxially on differently oriented MgO substrates. In the hydrated perovskite phase, proton conduction along the [100]/[001] directions (within the InO_6_ layers) exceeded that perpendicular to the layers, whereas in dry atmospheres oxygen-ion conduction was highest parallel to the one-dimensional vacancy channels of the brownmillerite phase. The anisotropy confirmed that engineered grain boundary orientation and density can tailor both H^+^ and O^2–^ pathways, offering an additional design degree of freedom beyond composition.

Recent work demonstrates two complementary, industry-friendly strategies for suppressing R_gb_ in protonic ceramic electrolytes. Choi et al.^312^ fabricated a columnar-structured thin electrolyte (∼2.5 μm) of BaCe_0.55_Zr_0.3_Y_0.15_O_3−δ_, in which no perpendicular grain boundaries exist against the current direction. A single screen-printing/cofiring step produced grains averaging 3.2 μm, so the charge path crosses virtually no high-resistance grain boundaries. The dense film delivered 350 mW cm^–2^ at 500 °C while retaining the scalability and low cost intrinsic to screen printing. Tang et al.^291^ solved the BZY20 densification conundrum with a BCZY + NiO support|BZY20 + NiO buffer|BZY20 membrane trilayer. The highly sinterable support transmits compressive stress to both the laminated BZY20 + NiO buffer layer and the BZY20 electrolyte, enabling full densification of Ce-free BZY20 to >95% relative density at 1450 °C—nearly 150 °C below the temperature needed for standalone BZY20 and without NiO/ZnO/CuO sintering aids that often form insulating proton traps at grain boundaries. The cell with a 15 μm-thick BZY20 (BZY–15) achieved 0.60 W cm^–2^ in fuel cell mode and −1.14 A cm^–2^ at 1.3 V in electrolysis mode at 600 °C. Thinning the electrolyte to 5 μm lifted performance to 1.24 W cm^–2^ and −2.33 A cm^–2^, respectively. Under 0.7 atm steam, −2 A cm^–2^, 600 °C, the BZY–15 cell operated over 800 h with negligible voltage rise and stable FE, whereas a BZCYYb1711 analog showed pronounced degradation.

Notably, microstructural engineering extends far beyond the single objective of reducing the interfacial resistance. Rather, it emphasizes the rational and targeted regulation of grain boundary regions to achieve a balance between electronic conduction suppression and ionic transport enhancement. Looking forward, advances in microstructural engineering can be significantly accelerated through the synergistic integration of *in situ*/*operando* characterization techniques, computational interface modeling, and machine-learning-assisted material exploration. These emerging methodologies are expected to provide deeper insights into intrinsic interfacial mechanisms and enable the predictive modulation of grain-boundary electrochemical behaviors. Despite these promising developments, the scalable translation of grain boundary modification strategies into industrially viable manufacturing techniques remains a major bottleneck, significantly hindering the commercialization of proton-ceramic fuel cells.

## 4 Characterization Methods

Proton uptake and proton transport constitute the two fundamental pillars governing the functionality of proton-conducting oxides. Proton uptake determines the concentration of protons accommodated by the lattice and their preferred crystallographic or defect-associated sites, whereas proton transport dictates how efficiently these species migrate in response to electrochemical driving forces. Because both processes span a wide range of length and time scales—from local O–H bonding configurations and defect equilibria to long-range diffusion and macroscopic conduction—no single experimental method can capture their full complexity. Quantitative understanding, therefore, emerges only through an integrated, multitechnique approach.

To chart this multiscale landscape, researchers employ gravimetric methods, vibrational and electronic spectroscopies, neutron-based structural and dynamical probes, and a suite of electrochemical and isotope-tracing techniques. Each provides a distinct window: TGA^313^ and Karl Fischer titration (KFT)^314^ quantify bulk hydration; XPS,^315^ IR,^315^ Raman,^316^ and temperature-programmed desorption (TPD)^92^ elucidate surface chemistry and hydroxyl environments; neutron powder diffraction (NPD),^317^ INS,^318^ quasi-elastic neutron scattering (QENS),^63^ and neutron pair-distribution-function (PDF)^173^ analysis uniquely resolve proton positions, local disorder, and jump dynamics; NMR^319^ identifies site-specific proton populations; and electrochemical methods such as EIS,^320^ EMF,^321^ and Hebb–Wagner polarization^322^ probe macroscopic transport pathways and transference numbers. Their complementary capabilities—and equally important limitations—are summarized in Table 4, which provides a consolidated overview of the experimental landscape, together with the typical temperature windows over which each technique is routinely applied, to underpin the discussions in Sections 4.1 and 4.2.

**Table 4:** Key Features, Capabilities, and Typical Operating Temperature Windows of the Selected Electrolyte Characterization Methods for P-RSOCs

Technique	Principle	Scale/Depth	Typical temperature window	Information obtained	Strengths	Limitations	Ref.
QENS	Proton jump diffusion broadening	Å–nm	200–800 °C	★Jump lengths	Direct probing of proton dynamics	Model-dependent data interpretation	^63^
				★Residence times			
				★Activation energies			
H_2_O–TPD	*m*/*z* = 18 desorption during heating	Surface and subsurface	20–800 °C	★Water binding energies	High signal-to-noise sensitivity	Surface-dominated information	^92^
				★Lattice OH stability			
XPS	Core-level photoemission	Surface (∼5 nm)	20–600 °C	★O 1s species	Direct chemical-state specificity	Surface-limited probing depth	^170^
				★Hydroxyl vs lattice oxygen			
Neutron PDF	Fourier transform of total scattering	1–10 Å	20–800 °C	★Local disorder	Sensitive to local structural disorder	High-energy neutron requirement	^173^
				★Defect networks			
				★Trapping motifs			
TGA	Monitors mass change during hydration/dehydration	Bulk	20–1000 °C (hydration isobars typically 100–900 °C)	★Hydration isotherms	★Quantitative mass sensitivity	Lack of species specificity	^313^
				★Diffusion fronts	★Defect-coupled kinetics		
KFT	I_2_ redox titrates evolved H_2_O	Bulk	Sample release at 600–1000 °C; titration at 20 °C	★Absolute OH^–^ content	Absolute quantitative accuracy	★Destructive analysis	^314^
				★Hydration stoichiometry		★Carbonate interference	
FTIR/DRIFTS	IR absorption	Surface and shallow bulk	20–700 °C (in situ cell)	★OH bonding motifs	High sensitivity to hydroxyl bonding	Complex spectral assignment	^315^
				★Hydration kinetics			
Raman/operando Raman	Vibrational spectroscopy	Surface–bulk	20–800 °C	★Hydroxyl intermediates	Operando vibrational accessibility	Weak hydroxyl scattering cross-section	^316^
				★Reaction pathways			
NPD	Neutron nuclear scattering	Long range	20–800 °C	★Oxygen/vacancy order	High sensitivity to hydrogen positions	Large sample requirement	^317^
				★Proton site occupancy			
INS	Neutron vibrational scattering	Local	–269–20 °C	★Proton configurations	Direct vibrational fingerprinting of OH species	Limited facility accessibility	^318^
				★OH bending/stretching			
NMR (^1^H MAS, relaxometry)	Nuclear spin transitions	Local	20–400 °C	★Local OH environment	High site-specific resolution	Limited high-temperature applicability	^319^
				★Dynamics			
EIS	Frequency-dependent impedance	Bulk and grain boundary	200–900 °C	★Bulk and grain boundary conductivity	Comprehensive electrochemical sensitivity	Electrode-dependent artifacts	^320^
				★ASR			
EMF	Voltage under a chemical potential gradient	Bulk	400–900 °C	Proton/oxide ion/electron transference number (t_e_)	Direct thermodynamic transference measurement	Strict gas-control requirement	^321^
Hebb–Wagner Polarization	Blocking electrode partial polarization	Bulk	400–900 °C	Minority carrier conductivity	Selective minority-carrier sensitivity	Ideal-blocking-electrode requirement	^322^
PALS	Positron lifetime sensitive to open volumes	Bulk (defect-selective)	20–600 °C	★Vacancy clusters	Unique sensitivity to vacancy topology	Indirect defect interpretation	^369^
				★Cation/oxygen vacancy size and concentration			
H/D Isotope Exchange (macroscopic and spectroscopic)	Replace H with D and monitor the rate	Surface and bulk	200–900 °C	★Proton exchange rates	Simple indicator of proton mobility	Experimental-condition-sensitive quantification	^372^
				★Site acidity			
				★Mechanism			
TOF-SIMS	Mass-resolved ion sputtering	nm−μm	Exchange anneal 200–700 °C; SIMS analysis at 20 °C	★D/H profiles	High depth-resolved chemical sensitivity	Sputtering-induced artifacts	^379^
				★Grain boundary diffusion			
				★Surface segregation			
DC Four-Probe	Steady-state I–V	Bulk	200–900 °C	★Total conductivity	Simple conductivity determination	Limited phase separation capability	^390^
				★Protonic fraction			
Isotope Tracer Diffusion (^18^O, D SIMS, TOF-SIMS)	Depth profiling of isotope penetration	μm–mm	Exchange 200–800 °C; SIMS at 20 °C	★Self-diffusion	Gold-standard diffusion quantification	High experimental cost	^409^
				★Surface exchange			

Together, these techniques establish a coherent mechanistic picture of proton behavior in oxides: how protons are incorporated into the lattice, how they interact with defects and local bonding environments, how their configurations evolve under hydration or dehydration, and ultimately, how they traverse microscopic pathways to give rise to macroscopic conductivity. The following subsections, therefore, detail the operating principles of each technique, the information that it yields, and the specific insights that it provides into proton uptake (Section 4.1) and proton transport (Section 4.2).

### 4.1 Proton Uptake

#### 4.1.1 TGA

TGA has become an indispensable tool for probing the defect chemistry of proton-conducting oxide electrolytes, particularly for RSOCs. By continuously monitoring sample weight under controlled temperature and atmosphere, TGA directly measures oxygen loss/gain and water uptake/release in these materials. This allows researchers to quantify fundamental equilibrium constants for key defect reactions—notably the oxidation of oxygen vacancies and the hydration reaction—and to observe how protonic defects form and migrate.^313^ Beyond equilibrium thermodynamics, TGA also provides insight into hydration/dehydration kinetics and the spatial distribution of defects during transient processes, shedding light on how these electrolytes behave across temperatures and gas conditions relevant to fuel cell or electrolyzer operation.

Efforts to extract equilibrium constants focus primarily on oxidation and hydration equilibria. For acceptor-doped perovskite electrolytes, the oxidation equilibrium constant K_O_ is typically defined by eq 12.^313^ This expression describes the tendency of the material to form electron–holes by oxidizing oxygen vacancies in an oxygen-containing atmosphere. By measuring the sample mass as a function of oxygen partial pressure under dry conditions, researchers use TGA to quantify K_O_ as a function of temperature.

Similarly, the hydration equilibrium constant, K_W_, defined by eq 5, describes water incorporation into the oxide lattice. This hydration is captured by TGA as a mass increase when the material is exposed to humidified gas. By equilibrating the sample at a fixed temperature under various pH_2_O, one can determine K_W_ from the dependence of proton concentration on water vapor pressure (Figure 25a). Both K_O_ and K_W_ are temperature- and material-dependent; Van’t Hoff analysis of TGA data yields the enthalpies and entropies associated with redox and hydration processes (Figure 25b).^323^

12 $$K_{O}=\frac{{\left[h^{\cdot }\right]}^{2}\cdot \left[O_{O}^{\times }\right]}{p_{O_{2}}^{1/2}\cdot \left[V_{O}^{\cdot \cdot }\right]}$$

**Figure 25:**
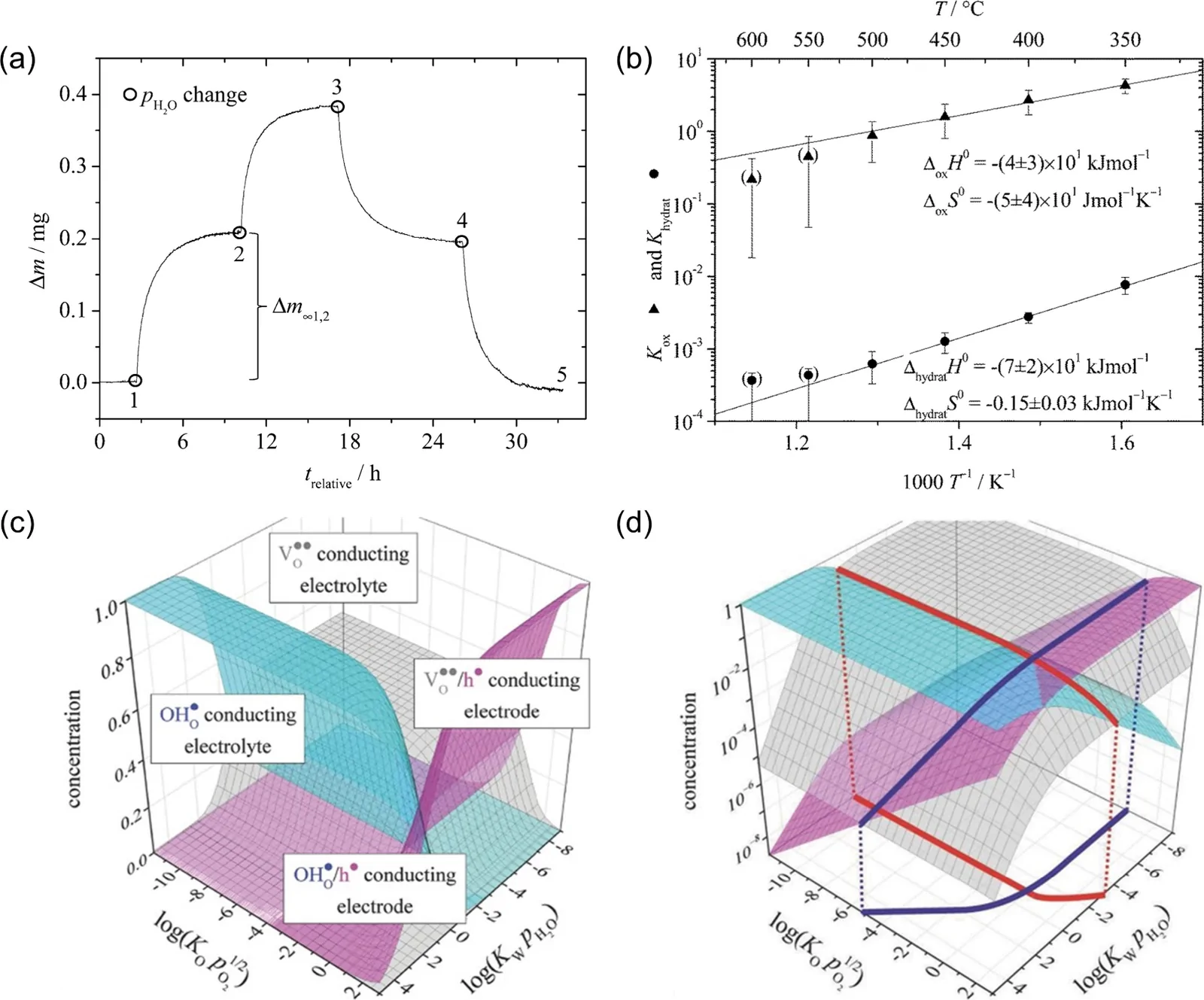
(a) Exemplary mass relaxation upon changing pH_2_ O at *T* = 400 °C and pO_2_ = 10 mbar (pellet mass = 3.88 g). pH_2_O was changed from 5.70 (point 1) to 9.83 (2) to 14.5 (3) to 9.83 (4) and to 5.70 (5) mbar. (b) Mass action constants of reaction 1 (hydration, numerical solution) and reaction 2 (oxidation, calculated from thermogravimetric isobars in a dry O_2_ atmosphere). Owing to the high error bars resulting from an error of ± 0.02 for the absolute δ and K values at 550 and 600 °C are excluded from the linear regression. Reproduced with permission from ref 323. Copyright 2014 RSC. Concentration (molar fraction) of oxygen vacancies V_O_^••^ (gray), protons OH_O_^•^(blue), and holes h^•^ (pink) in an acceptor-doped oxide (A’] = 1) as functions of $K_{O}p_{O_{2}}^{1/2}$ and K_W_p_H_2_O_. (c) linear scale, (d) logarithmic concentration scale, the red and blue lines mark the intersections with [V_O_^••^] = [OH_O_^•^] and [V_O_^••^] = [h^•^]; also, their projections to the base plane are shown. Reproduced with permission from ref 313. Copyright 2015 Wiley.

Crucially, TGA enables detailed insight into the coupling between oxygen exchange and hydration. In humid atmospheres, proton incorporation can occur not only via the “pure” hydration mechanism (eq 1)—where water fills pre-existing oxygen vacancies—but also through a redox-hydration pathway (eq 13), often referred to as hydrogenation.^313^ In this case, protons are incorporated by consuming electron holes and lattice oxygen, forming hydroxide defects while releasing oxygen. This coupled mechanism allows proton uptake even when oxygen vacancies are scarce and becomes particularly important under p-type conditions.

13 $$H_{2}O+2\left[O_{O}^{\times }\right]+2h^{\cdot }\to 2\left[{\mathrm{OH}}_{O}^{\cdot }\right]+\frac{1}{2}O_{2}$$

14 $$K_{W/O}=\frac{p_{O_{2}}^{1/2}\cdot {\left[{\mathrm{OH}}_{O}^{\cdot }\right]}^{2}}{p_{H_{2}O}\cdot {\left[O_{O}^{\times }\right]}^{2}\cdot {\left[h^{\cdot }\right]}^{2}}$$

The equilibrium constant for hydrogenation K_W/O_ is effectively the quotient of K_W_ and K_O_ (eq 14) and can be determined by TGA under controlled pO_2_ and pH_2_O.^313^ By monitoring weight changes from both water incorporation and oxygen release or uptake, TGA quantifies the relative contributions of the two pathways and reveals which defect mechanism dominates under specific conditions.

Y-doped BaCeO_3−δ_ (e.g., BaCe_0.9_Y_0.1_O_3−δ_) is a prime example.^324^ TGA measurements show it absorbs 0.012–0.11 wt % water between 300–600 °C in humid environments, reflecting a large K_W_ and a strongly exothermic hydration enthalpy (−120 to −150 kJ mol^–1^ H_2_O). Even at 600–700 °C in wet air, the material retains a substantial proton population. The hydration entropy (−140 to −150 J mol^–1^ K^–1^) reflects the ordering associated with incorporating gaseous H_2_O into the solid lattice. Increasing pH_2_O leads to saturation behavior, consistent with full occupancy of available oxygen vacancies.

TGA also captures BaCeO_3−δ_’s moderate redox activity. Under low pO_2_, Ce^4+^ is partially reduced to Ce^3+^, generating additional vacancies and causing measurable oxygen loss. This redox behavior supports the hydrogenation mechanism when hole concentrations are high. In such cases, proton uptake is accompanied by oxygen evolution, and TGA provides a direct measure of the extent of coupling.^324^

These insights inform the rational engineering of RSOC. In real RSOC operation, BaCeO_3−δ_-based electrolytes are usually well hydrated on the anode side (humid H_2_) but can begin to dehydrate on the cathode side if temperature rises or humidity drops.^321^ This can reduce proton conductivity and introduce minor p-type conduction. Fortunately, TGA data show that unless extreme conditions are applied, the hole concentration remains low due to BaCeO_3−δ_’s relatively small K_O_, minimizing electronic leakage and preserving electrolyte integrity.^324^

Interpreting TGA data with defect models provides a self-consistent picture of the proton conductor’s thermodynamics. Measured weight changes can be translated into defect concentrations using mass balance and site-conservation relations. For example, in ABO_3_–type perovskites, charge neutrality implies the validity of eq 15, and oxygen site conservation gives eq 16.^313^

15 $$\left[A_{B}^{’}\right]=2\left[V_{O}^{\cdot \cdot }\right]+\left[{\mathrm{OH}}_{O}^{\cdot }\right]+\left[h^{\cdot }\right]$$

16 $$3-\delta =\left[O_{O}^{\times }\right]+\left[V_{O}^{\cdot \cdot }\right]+\left[{\mathrm{OH}}_{O}^{\cdot }\right]$$

By inserting equilibrium expressions for K_O_ and K_W_, one can predict how defect populations evolve with pO_2_, pH_2_O, and T. Conversely, the model can be fitted to TGA data to extract these equilibrium constants. Maier et al.^313^ demonstrated this approach by constructing defect phase diagrams for Ba­(Ce, Zr, Y)­O_3−δ_, showing transitions between oxide-ion, proton, and hole conduction regimes as functions of pO_2_ and pH_2_O (Figure 25c,d), where TGA provides the experimental basis for such maps.

Importantly, even in nominally protonic electrolytes like Y-doped BaZrO_3−δ_, high temperature or low humidity reduces [OH_O_^•^], while [h^•^] rises, producing p-type conductivity.^325^ TGA records this transition as a dehydration-induced weight loss, which defect models attribute to proton departure and increasing hole content. Introducing redox-active dopants like Fe can exacerbate this: e.g., in La_0.6_Sr_0.4_ScO_3−δ_, just 5% Fe doping reduces hydration due to increased p-type character. This trade-off, revealed by TGA, highlights the antagonism between K_O_ and K_W_—a key constraint in electrolyte design.^326^

TGA-based kinetic studies are not limited to perovskites. Lanthanum tungstate (La_6–x_WO_12−δ_), a mixed proton/oxide-ion conductor with minimal electronic conductivity, shows single-step hydration kinetics dominated by proton diffusion.^327^ In La/W = 5.6 samples, TGA weight transients under wet/dry switching exhibit smooth relaxation curves, allowing extraction of bulk proton diffusion coefficients. Unlike cerates or zirconates, La_6_WO_12−δ_ avoids multivalent redox: its hydration is insensitive to pO_2_, enabling clean determination of K_W_ and hydration thermodynamics (e.g., ΔH ≈ −90 kJ mol^–1^, ΔS ≈ −115 J mol^–1^ K^–1^). The absence of electronic defects decouples K_O_ and K_W_, enabling high proton uptake without electronic leakage.

Notably, TGA on La_6_WO_12−δ_ also shows isotope effects: switching from H_2_O to D_2_O reduces the mass gain and reveals reversible steps from H/D substitution, confirming protonic species are responsible.^327^ Similar insights apply to aliovalent-doped La perovskites (e.g., LaScO_3_, LaNbO_4_): these materials hydrate readily unless redox-active dopants like Fe or Co are introduced, which suppress hydration by lowering K_W_ and raising K_O_. TGA weight gain correlates directly with protonic conductivity, guiding composition tuning for optimal hydration.

Beyond equilibrium measurements, TGA is equally powerful for probing the kinetics of hydration and dehydration—processes that are critical for RSOC electrolytes subjected to dynamic operating conditions such as rapid gas switching or thermal cycling during start-up and shut-down. By abruptly changing the gas environment (e.g., switching from a dry to a humid atmosphere or stepping pO_2_) and recording the time-resolved mass response, TGA yields relaxation curves that reflect the material’s ability to incorporate or release water in real time. The shape and time scale of these transients encode valuable information about the mobility of ionic and electronic defects, as well as the pathways through which equilibrium is re-established.^313^

In classical diffusion-limited systems with a single mobile species, relaxation follows an exponential decay governed by a chemical diffusion coefficient (D_chem_). However, proton-conducting oxides are inherently multidefect systems involving at least protons and oxygen vacancies and frequently also electronic carriers. Under such conditions, TGA reveals that the hydration kinetics often deviate from single-exponential behavior, exhibiting two-stage (or “2-fold”) dynamics.^313^ This has been corroborated by electrical conductivity relaxation (ECR) studies, which report an initial fast response, followed by a slower secondary process upon exposure to water vapor. TGA confirms these observations with direct mass transients, enabling a more refined kinetic modeling.

In p-type Y-doped BaCeO_3−δ_, for example, Lim et al.^324^ observed a nonmonotonic hydration curve upon switching to humid gas: an initial rapid mass gain, a transient plateau, or even a slight reversal, followed by a slower, secondary weight increase. This behavior is consistent with a two-step diffusion mechanism: a fast redistribution of protons and holes near the surface, followed by slower equilibration involving oxide ion or vacancy diffusion in the bulk. Defect-chemically, these stages correspond to distinct ambipolar diffusion processes—one involving codiffusion of protonic defects and holes, and another involving oxygen vacancies and holes. Each process is characterized by its own time constant and spatial scale.

Importantly, the initial proton–hole diffusion front may hydrate the near-surface region quickly, while the bulk lags due to slower oxide ion transport. In this scenario, proton concentrations can overshoot equilibrium near the surface, producing a temporary reversal in mass change—a phenomenon known as “overshoot” or uphill diffusion. This striking kinetic feature, captured only through high-sensitivity TGA, reflects the complex interplay between multiple defect species and their cross-coupled transport.

By fitting these time-resolved mass curves to multispecies diffusion models, researchers can extract effective diffusion coefficients for both protons and oxygen vacancies, as well as interspecies coupling terms. These analyses consistently show that hydration proceeds through strongly concentration-dependent transport: proton mobility in the already hydrated near-surface region is reduced, resulting in a sharp reaction front that propagates into the bulk.^323^ The presence of such a moving hydration front has been directly confirmed experimentally—for example, spatially resolved optical-absorption imaging in perovskite oxides has visualized a well-defined internal interface separating hydrated and dry regions.^328^ These observations demonstrate that water incorporation is inherently heterogeneous and governed by localized defect gradients rather than uniform bulk diffusion. These insights highlight the importance of considering spatially varying defect populations and cross-coupled transport when evaluating electrolyte behavior under practical RSOC operating conditions.

In summary, TGA provides a quantitative window into the complex defect chemistry of proton-conducting electrolytes. By determining equilibrium constants like K_O_ and K_W_, TGA pins down the thermodynamic landscape that governs how protons, vacancies, and holes populate the material under various temperatures and gas atmospheres. By monitoring mass relaxations and multistep kinetics, TGA illuminates the transport processes and defect interactions that occur when those conditions change. The technique bridges the gap between macroscopic behavior (weight change) and microscopic defect mechanisms, allowing researchers to derive meaningful models that predict performance in real RSOC operations. Whether for a classic perovskite like BaCeO_3−δ_, a robust BaZrO_3−δ_, or a tailored La-based compound, the story is similar: TGA measures the quantity and efficiency with which oxygen and hydrogen are absorbed or released. These measurements, interpreted through defect chemistry, are key to optimizing electrolyte materials so that they maintain high proton conductivity, minimal electronic leakage, and structural integrity across the wide-ranging conditions of reversible fuel cells and electrolyzers. The logic flow from TGA data to defect modeling thus enables a deep understanding of electrolyte functionality—linking raw weight changes to equilibrium constants, these constants to defect distributions, and finally defect behavior to the macroscopic performance and stability of protonic solid oxide cells.

#### 4.1.2 XPS

XPS probes the outermost 5–10 nm of proton-conducting oxides, precisely the region where hydration and proton transfer first occur. When a monochromatic Al Kα beam (hν ≈ 1486.7 eV) ejects core-level electrons, the analyzer records their kinetic energy and converts it to binding energy through eq 17, where E_kin_ is kinetic energy, E_B_ is binding energy, and Φ is the work function of electrons.^329^ Because binding energies are element-specific and sensitive to chemical bonding, XPS spectra reveal both which elements are present and their oxidation or functional state. The technique is inherently surface-focused due to the short inelastic mean free path of electrons in solids (1–3 nm path length corresponding to 5–10 nm sampling depth), meaning it probes the near-surface region where proton incorporation often first manifests.^330^

17 $$E_{\mathrm{kin}}=h\nu -E_{B}-\Phi$$

Importantly, while XPS can detect virtually all heavier elements (atomic number Z) and provide quantitative atomic ratios, it cannot directly detect hydrogen. Hydrogen’s 1s electron has no core level accessible in the typical XPS energy range, and any photoemitted electrons from H are extremely low in energy and absorbed before reaching the detector. As a result, protons in a material are not observed as a distinct XPS peak. Instead, proton uptake is inferred indirectly—primarily through the presence of hydroxyl species or related chemical shifts in other elements. When a proton-conducting oxide incorporates H^+^ into its lattice (usually as hydroxide O–H bonds at oxygen sites), the O 1s core-level spectrum typically shows an additional component at a higher binding energy compared to lattice oxide ions.^267^ This higher E_B_ O 1s signal (often in the 531–533 eV range, vs 528–530 eV for lattice O^2–^) is attributed to oxygen atoms bonded with hydrogen (hydroxyl species) or adsorbed water on the surface.^267,331^ By deconvoluting the O 1s peak, one can quantify the fraction of oxygen present as hydroxyl, providing a measure of protonation at the surface. Additionally, the incorporation of protons can cause subtle shifts in cation core levels if charge compensation or changes in valence occur. For instance, adding protonic defects may lead to a slight reduction of transition-metal cations (to maintain charge neutrality), which XPS can detect as changes in peak positions or the appearance of mixed valence states.^332^

Early systematic work on doped perovskites such as BaCeO_3−δ_–BaZrO_3−δ_ established this fingerprint. Norby et al.^332^ demonstrated that treating SrZrO_3_ in dry H_2_ splits the O 1s signal into lattice (529 eV) and surface-hydroxyl (531 eV) components, the latter growing during exposure and shrinking when the gas is removed, perfectly mirroring changes in near-surface proton conductivity.^331^ Saito et al.^170^ extended the approach to the high-performance BaSc_0.8_Mo_0.2_O_3−δ_ electrolyte. The XPS core-level spectra showed the expected oxidation states for the cations, and the overall surface composition corresponding to the formula (Ba^2+^)­(Sc^3+^)_0.8_(Mo^6+^)_0.2_(O^2–^)_2.8_(OH^–^)_y_. The inclusion of OH^–^ in the formula directly confirms proton uptake through the clear O–H contribution to the O 1s peak. While the exact proton content y was determined by complementary methods, the XPS data confirmed that the material’s surface was protonated under the tested conditions.

Beyond hydroxyl identification, XPS tracks the cation redox that often accompanies proton incorporation. In a photoassisted protonation study of Al-doped CeO_2_, Xie et al.^332^ observed a growth of Ce^3+^ features in the Ce 3d spectrum as surface hydroxyl and oxygen-vacancy contributions simultaneously rose in the O 1s envelope—quantitative evidence that UV treatment injects protons while reducing Ce to preserve charge neutrality.

Modern near-ambient-pressure XPS (NAP-XPS) now follows these processes under technologically relevant water vapor (Figure 26a,b).^333^ Using NAP-XPS, a recent investigation of BaZr_0.8_Y_0.2_O_3−δ_ revealed that humidification at 550 K increases both surface hydroxyls and under-coordinated oxygen and that a modest 1 V bias further amplifies these species (Figure 26c). Impedance data recorded in parallel showed a 2-fold rise in proton conductivity, linking the spectroscopic signatures to functional performance.

**Figure 26:**
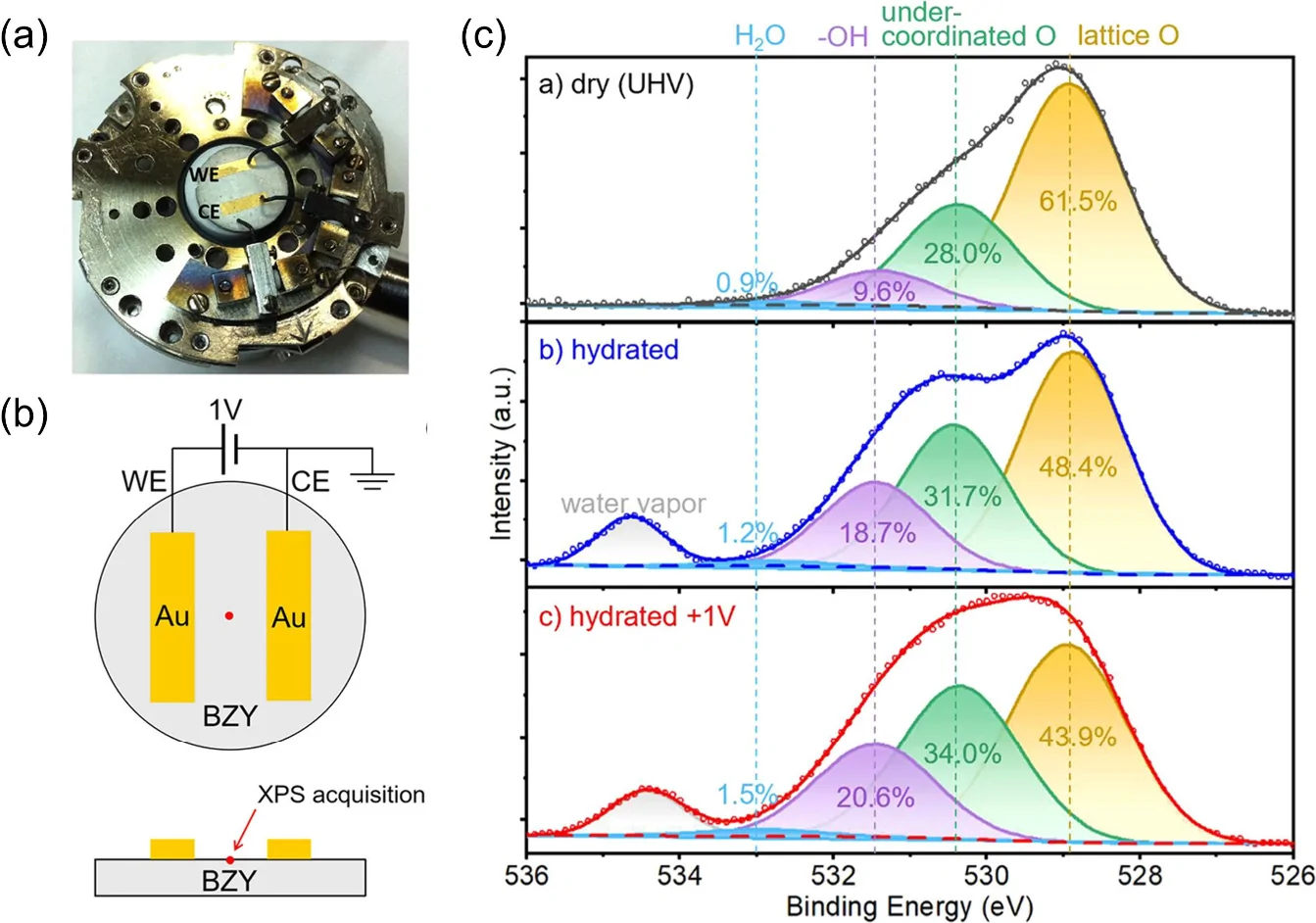
(a) BZY20 mounted on the sample holder, and (b) Schematic drawing of top and sectional view for the BZY20 pellet and electrical connection. WE, working electrode; CE, counter electrode (grounded). (c) O 1s spectra for BZY20 at three conditions, deconvoluted by four peaks with the same width and constrained position: lattice O (yellow, 528.9 eV); undercoordinated O (green, 530.4 eV); hydroxyl group −OH (violet, 531.4 eV); adsorbed intact H_2_O (blue, 533.0 eV). Reproduced from ref 333. Copyright 2022 American Chemical Society.

XPS also exposes side reactions that compete with—or even masquerade as—protonation. In Y-doped BaZrO_3−δ_, Sazinas et al.^74^ detected extensive BaCO_3_ formation after CO_2_ exposure; the carbonate O 1s (531–532 eV) and C 1s (289 eV) signals grew at the expense of Ba^2+^ features, indicating that humid, CO_2_-containing atmospheres can leach Ba as Ba­(OH)_2_ or BaCO_3_ and degrade electrolyte surfaces. Such observations underscore that XPS simultaneously uncovers desirable hydroxylation and deleterious carbonate or hydroxide layers, providing a comprehensive picture of surface chemistry and stability.

Taken together, insights from conventional UHV measurements, near-ambient-pressure experiments, and carefully designed model studies converge to a consistent mechanistic picture. Acceptor doping first creates oxygen vacancies; hydration then fills those vacancies with hydroxyls, a process tracked unambiguously by the high-binding-energy shoulder in the O 1s envelope, while concomitant cation redox restores the charge balance. Because these chemical signatures evolve measurably under operating atmospheres and even under applied bias, XPS can monitor protonation dynamics and degradation pathways in real-time, making it an chemically specific probe for optimizing proton-conducting electrolytes in RSOCs.^332^

The same body of work underscores why XPS has become indispensable: its bond-level resolution separates lattice oxygen from hydroxyl or adsorbed water, identifies accompanying changes in cation valence, quantifies surface stoichiometry, and reveals parasitic phases such as Ba­(OH)_2_ or BaCO_3_ that emerge under humid or CO_2_-containing conditions. These capabilities illuminate how proton uptake modifies interfacial chemistry and, by extension, electrochemical performance. Yet the advantages carry inherent caveats. Because hydrogen itself is invisible, XPS detects only those protons that perturb neighboring atoms. It samples just the outer few nanometers, so bulk hydration remains hidden; vacuum transfer can dehydrate surfaces or rearrange defects; overlapping peaks demand meticulous deconvolution; ion-sputter depth profiling is prone to artifacts; and small analysis areas may overlook heterogeneity. Consequently, XPS functions best as the surface-sensitive “microscope” in a broader toolkit, to be complemented by bulk-sensitive techniques such as TGA, IR, or nuclear magnetic resonance spectroscopy, and electrochemical measurements. When deployed judiciously in this multimodal framework, XPS offers a uniquely detailed chemical map of proton incorporation, degradation, and interfacial evolution—knowledge that is pivotal for the rational design and long-term stability of next-generation RSOCs electrolytes.

#### 4.1.3 H_2_O-TPD

H_2_O-TPD migrated into proton-conducting oxides from catalytic science, where it had long served as a thermometer for the energetics and kinetics of water adsorption–desorption on metal-oxide surfaces. In the porous composite electrodes of RSOCs—typically Ni plus a triple-conducting oxide mixed with the protonic ceramic—the method quickly proved indispensable:^325^ the centimeter-scale flow paths in these networks mean that most water resides either at external sites or in shallow surface vacancies. As a result, when the programmed ramp begins, hydrogen-bond cleavage is immediately followed by a burst of *m*/*z* = 18 (where m is the ion mass in atomic mass units and z is the charge number), which the quadrupole detects with an excellent signal-to-noise ratio. Ding’s study separated a 400–600 °C lattice-hydroxyl band from a shoulder of weakly bound water and correlated the integrated high-temperature area with the electrode’s steam-oxidation activity, thereby confirming a viable proton pathway across the intended operating window.^92^

That clarity is rarely matched in dense electrolytes. After sintering above 1400 °C, their grains reach micron dimensions and the specific surface area falls by 100-fold, so most protons reside deep inside the lattice. To liberate them within a few tens of minutes of a TPD scan, the proton–vacancy pair must diffuse across an entire grain, a process orders of magnitude slower than surface desorption and often buried in baseline drift. The very high temperatures needed to complete dehydration—typically 800–1000 °C under vacuum or inert gas—risk carbonate formation or A-site loss in Ba- and Sr-rich perovskites, so any water detected above about 850 °C can carry an ambiguous fingerprint.^325^ Consequently, electrolyte researchers tend to weigh total uptake with TGA, then probe local hydroxyl environments with *in situ* DRIFTS, near-edge X-ray absorption, or neutron scattering, leaving H_2_O-TPD to electrode specialists.

A handful of recent papers nonetheless demonstrate that the technique can succeed on electrolytes when experimental obstacles are engineered away. Vera et al.^325^ ground high-Sc BaZrO_3−δ_ to submicron powders, limited the sample mass to ∼ 10 mg, slowed the ramp to 4 °C min^–1^, and held the reactor at quasi-steady states; two well-resolved water peaks then emerged, and the larger, high-temperature event revealed that Sc-rich “Zr–OH–Sc” traps immobilize a fraction of protons and throttle conductivity at elevated temperatures. Their protocol underscores both the sensitivity and the labor intensity required when diffusion and secondary reactions conspire against a clean spectrum. Where the technique does deliver, its advantages are decisive: the mass spectrometer assigns the desorbing species unambiguously to H_2_O, eliminating the CO_2_ or O_2_ artifacts that complicate gravimetric curves; the temperature-resolved profile acts as an intrinsic calorimeter, with peak maxima shifting higher as hydroxyl binding strengthens, allowing semiquantitative ranking of materials by defect stability; and the experiment can be run under dry O_2_ or N_2_, recreating cell atmospheres without an applied potential.

Yet the same features expose the limitations. Quantitative proton counts demand careful calibration of the ion-current-to-water-dose relationship and presuppose that every lattice hydroxyl recombines during the ramp; if deep traps remain, the uptake will be underestimated. Residual physisorbed water can swamp the low-temperature region unless the sample is purged at 100 °C in dry gas, as Song did, and overlapping peaks from multiple sites complicate deconvolution unless supported by complementary DRIFTS or isotope exchange.^325^ Instrumentation is another barrier: a high-vacuum or flowing-gas TPD manifold coupled to a sensitive quadrupole is still rarer than a standard TGA furnace. Finally, H_2_O-TPD follows only the dehydration leg of the cycle, so full insight into hydration thermodynamics usually requires pairing with isothermal uptake experiments or conductivity measurements under controlled steam pressures.

Taken together, the technique thrives in porous electrodes, where water desorption mirrors surface proton kinetics and faces intrinsic challenges in the dense, high-temperature electrolytes that dominate protonic ceramics. Even so, when diffusion is accelerated by nanostructuring or trap chemistry is under scrutiny, carefully executed H_2_O-TPD remains uniquely powerful for connecting microscopic proton-defect energetics with the macroscopic performance metrics of RSOCs.

#### 4.1.4 KFT

KFT underpins quantitative hydration studies because it reports only the H_2_O released from a specimen, translating that mass into an absolute proton inventory through the well-defined iodine redox reaction. In coulometric mode, the detection limit reaches the single-ppm range, and replicate measurements on lightly doped BaZrO_3−δ_ routinely show relative standard deviations below 0.3%. Early KFT-based hydration studies on Y-doped BaZrO_3−δ_, such as those by Han et al.,^334^ demonstrated that this method can reliably quantify incorporated protons and correlate water uptake with lattice expansion during hydration-dehydration cycles. After hydration under 0.05 atm H_2_O at 500–700 °C, pellet fragments a few millimeters across are weighed in a dry glovebox, sealed, and transferred to an external furnace; crushing the sintered body accelerates desorption, while a brief purge in CO_2_-free gas removes surface carbonates that would otherwise overestimate water uptake. Once the titrated mass m_H_2_O_ is obtained, the proton concentration per formula unit follows directly from eq 18, putting KFT values on the same scale as conductivity or defect-thermodynamic data, and plotting lattice constants measured by *in situ* HT-XRD against these KFT-derived hydroxyl counts reveals a single linear relation across the Y- and Ce-doped BaZrO_3−δ_ series.^334^

18 $$c_{H^{+}}=\left(m_{H_{2}O}/M_{\mathrm{sample}}\right)/\left(0.5M_{H_{2}O}\right)$$

where c_H^+^_ is the concentration of protons per formula unit, m_H_2_O_ is the mass of desorbed water determined by KFT, M_sample_ is the total mass of the analyzed sample, and M_H_2_O_ is the molar mass of water. The factor 0.5 accounts for the stoichiometry of one H_2_O molecule contributing two protons.

KFT quantifies every mole of water released from a proton-conducting oxide by tracking the iodine consumed in the eq 19, so the charge (in coulometric mode) or volume (in volumetric mode) of I_2_ delivered converts directly into lattice proton content.^335^ For dense perovskites, the solid is first hydrated under a controlled pH_2_O (typically 0.05 atm) at 500–700 °C, then quenched to room temperature to freeze the hydroxyls. The quenched pieces—usually ≤ 2 mm to minimize external condensation—are placed in a predried tube furnace; as the temperature ramps to 600–1000 °C, a dry N_2_ stream sweeps the evolved H_2_O into a coulometric cell where μg-level precision is routine, provided the entire transfer line is sealed from ambient moisture.

19 $$I_{2}+2H_{2}O+{\mathrm{SO}}_{2}↔2\mathrm{HI}+H_{2}{\mathrm{SO}}_{4}$$

Early systematic work on Y-doped BaZrO_3−δ_ used this protocol to map proton concentrations across 0–30 mol % Y. KFT showed that water uptake scales monotonically with vacancy content: after 6 days in Ar + 5% H_2_O, the proton inventory climbed from <10^19^ cm^–3^ in undoped BaZrO_3−δ_ to >10^21^ cm^–3^ in BaZr_0.7_Y_0.3_O_3−δ_ at 300 °C, and similar trends persisted at 600 °C, albeit at lower absolute loadings. Parallel high-temperature XRD revealed that the hydration-induced lattice swelling tracks the KFT-derived proton fraction but plateaus between 10–15 mol % Y, implying a change in the preferred proton site beyond that composition. Hiraiwa et al.^336^ linked the same KFT data to an anomalous 300–450 °C lattice contraction during HT-XRD heating—direct evidence that dehydration, not thermal expansion, drives the shrinkage in this window.

Substituting Ce for Zr in BaZr_0.8–x_Ce_x_Y_0.2_O_3−δ_ further amplifies water uptake.^66^ Han et al.^337^ found that at 700 °C the hydration degree of BZY20 is only ∼ 25%, whereas Ce-rich BCY20 retains ∼ 73% of its vacancies as hydroxyls; at 600 °C the most Ce-rich samples approach 95% saturation. The dehydration temperature extracted from KFT data rises from 375 ± 25 °C for BZY20 to well above 600 °C when x ≥ 0.6, confirming that Ce stabilizes lattice protons thermodynamically. These absolute proton concentrations—up to 8 × 10^21^ cm^–3^ at 500 °C—underpin the superior conductivity and Faradaic efficiencies reported for Ce-rich electrolytes.

KFT is equally diagnostic when hydration is suppressed. In Mn-, Fe- or Co-modified BZY20, introducing only 1.5 at. % Ni during sintering cuts the KFT-measured water content by almost half relative to pristine BZY20 after identical 600 °C humidification, reducing available protons to the mid-10^20^ cm^–3^ range and mirroring a drop in proton conductivity observed electrochemically; Ni diffusion blocking vacancy sites are thereby identified as the culprit.^337^

Because KFT records only an end point, complementary methods are now wired in tandem: Thermal programmed desorption-mass spectroscopy dissects the release profile that KFT integrates, and the absolute KFT number calibrates TGA baselines so gravimetric peaks translate into real water concentrations; Han et al.^337^ used this dual workflow to separate hydroxyl loss from simultaneous volatile reduction in Ni-modified BZY20 and to correlate each event with valence changes observed by *operando* NAP-XPS. Synchrotron XRD/KFT hybrids extend the idea by tracking water evolution, chemical expansion, and vacancy ordering in a single temperature ramp, while automation—carousel furnaces processing 50 ampules overnight, inline FTIR for speciation, and microcoulometric-quartz crystal microbalance stages pushing detection below 2 μg—delivers the speed and sample economy needed for high-throughput electrolyte discovery. These refinements explain why the external-furnace coulometric protocol remains the most widely cited KFT workflow for rugged perovskites, even as headspace extraction and TGA-KFT hybrids gain traction for disentangling surface-adsorbed from true lattice water during compositional optimization in RSOCs research.

#### 4.1.5 IR and Raman Spectroscopy

##### 4.1.5.1 IR Spectroscopy

IR spectroscopy has become the work-horse for confirming and dissecting proton uptake in perovskite electrolytes because every hydration event adds an O–H oscillator whose stretching vibration announces itself, often unmistakably, between 2.5 and 3.6 μm. In the classical case of Sc-doped BaTiO_3_, Perrichon and co-workers followed this vibrational beacon through a polymorphic transition: in the cubic phase the ν­(O–H) maximum lies at 0.42 eV (3380 cm^–1^), but after a modest change in oxygen stacking the band collapses to 0.405 eV (3260 cm^–1^) and sharpens, signaling that protons now experience stronger and more uniform hydrogen bonds in the hexagonal lattice (Figure 27a).^315^ At the flanks of that principal envelope, faint satellites at 270 and 418 meV expose minority proton sites, and, tellingly, the red-shifted, narrowed band accompanies a two-order-of-magnitude fall in σ_bulk_—direct proof that vibrational softening reflects proton trapping and sluggish long-range transport.

**Figure 27:**
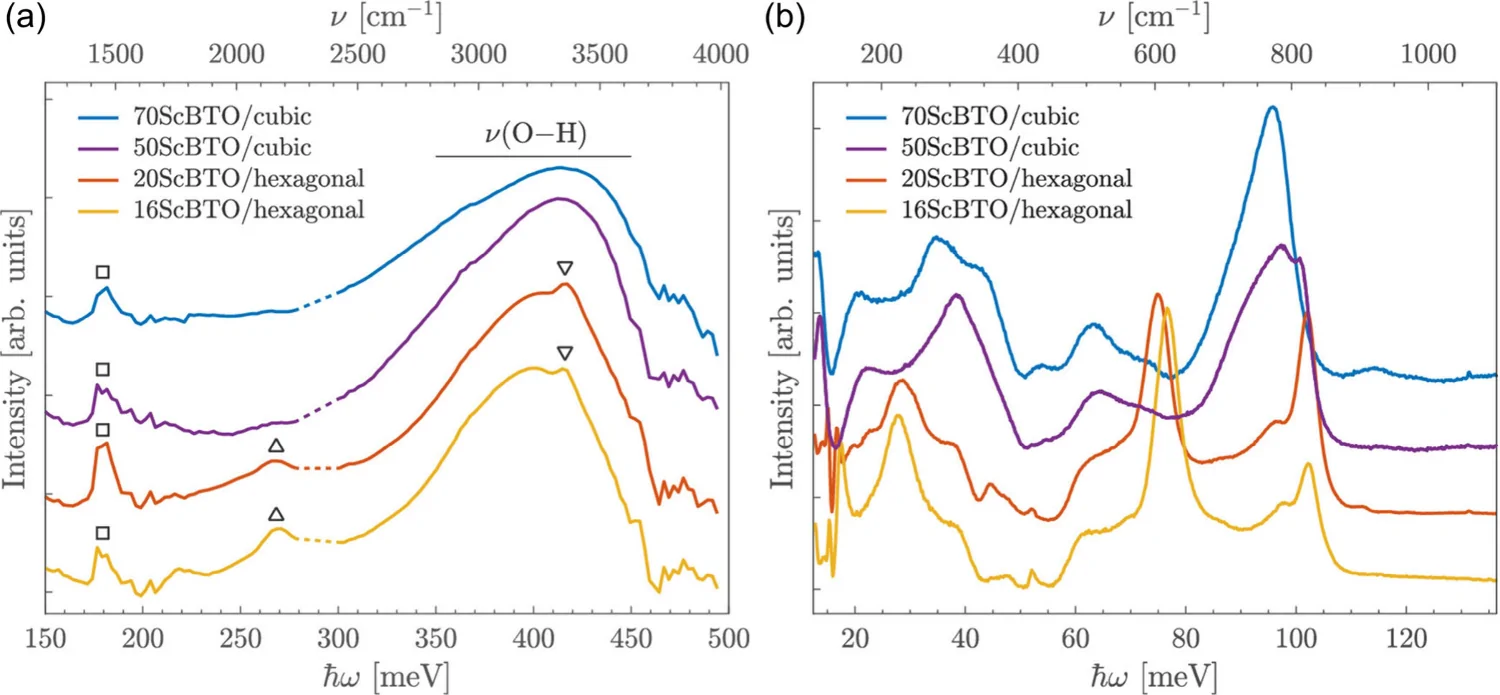
(a) IR spectra of BaTi_0.3_Sc_0.3_O_3_H_x_ (blue), BaTi_0.5_Sc_0.5_O_3_H_x_ (purple), BaTi_0.8_Sc_0.2_O_3_H_x_ (orange), and BaTi_0.84_Sc_0.16_O_3_H_x_ (yellow). The 276–300 meV region of the spectra is omitted due to the strong absorption of the Au reference; interpolated dashed lines are instead shown as a guide for the eyes. The peak at ca. 180 meV (squares) is assigned to a carbonated impurity. The spectra have been corrected by subtraction of a linear background and normalized to the integral of the v­(O–H) band (350–450 meV) and are vertically offset for clarity. (b) Raman spectra of BaTi_0.3_Sc_0.3_O_3_H_x_ (blue), BaTi_0.5_Sc_0.5_O_3_H_x_ (purple), BaTi_0.8_Sc_0.2_O_3_H_x_ (orange), and BaTi_0.84_Sc_0.16_O_3_H_x_ (yellow), over the lattice mode region. The spectra have been corrected by the subtraction of a linear background, have been normalized to unity, and are vertically offset for clarity. Reproduced from ref 315. Copyright 2020 American Chemical Society.

The same logic extends seamlessly to compositionally tuned materials. Naumovska et al.^338^ hydrated magnetron-sputtered BaZr_1–x_Sc_x_O_3–x/2_ films and showed that heavy Sc substitution (x ≥ 0.54) funnels protons into a single, weakly bonded configuration that produces a narrow, intense band at 3400–3500 cm^–1^, whereas lightly doped films (x = 0.45) display only a broad continuum between 2500 and 3700 cm^–1^, characteristic of a heterogeneous site distribution; parallel powder specimens retain that continuum at all Sc levels, underscoring how epitaxial strain can bias site occupancy. Because neutron-reaction analysis fixed the hydration degree, shifts in band intensity could be read quantitatively, revealing that site preference, not water content, governs the vibrational landscape and—by extension—the macroscopic conductivity hierarchy.

Chemical processing modifies the same spectral fingerprints. Mahmud et al.,^339^ for instance, tracked BaCe_0.4_Zr_0.2_Y_0.1_Sm_0.3_O_3−δ_ powders as functionalized activated carbon was introduced into a sol–gel route. After calcination at 1100 °C, both pristine and modified samples show the expected, broad O–H stretch between 2500 and 3600 cm^–1^, but only the pristine powder carries residual nitrate bands at 2031 cm^–1^ and 2158 cm^–1^; their disappearance in the modified version confirms cleaner combustion and hints at a proton sublattice freed from electron-scavenging impurities. This kind of process-interrogation demonstrates how IR can verify not just that hydroxide is present, but that parasitic species—which later poison electrochemical interfaces—have been removed.

Manipulating the dopant spectrum can instead bury the hydroxide signature. In a systematic study of BaZr_0.2_Ce_0.7–x_Sm_x_Y_0.1_O_3−δ_ (x = 0–0.3), Irshad and co-workers found that increasing Sm content broadens the 1420 cm^–1^ O–H bending mode yet leaves the stretching region almost featureless;^340^ complementary TGA shows barely 2% mass loss up to 150 °C, confirming that the residual water is physisorbed, not structurally bound. The absence of a prominent ν­(O–H) peak, therefore, flags inefficient hydration—a strategic warning when these materials are pursued as intermediate-temperature electrolytes.

Where multiple configurations coexist, their relative populations can be extracted by mathematical deconvolution. Arai’s *in situ* FTIR on Ba­(Zn, M)­O_2.9−δ_(OH)_2δ_ (M = Ta, W) resolved the composite 2500–4000 cm^–1^ band into two symmetric and two asymmetric hydroxide motifs.^341^ In the W-rich double perovskite, the asymmetric fraction dominates, and its intensity decays more slowly with temperature, mirroring a higher energetic barrier for proton release and explaining the two-order-of-magnitude conductivity drop relative to the Ta analogue. This level of spectral granularity would be unreachable without targeted curve-fitting combined with controlled thermal ramps.

Broad-band studies push the vibrational story into the terahertz domain. Using THz time-domain and IR reflection spectroscopy, Nagai et al.^342^ monitored Y- and Sc-doped BaZrO_3−δ_ pellets and showed that protons strongly damp the 7 THz Slater and 17 THz Axe phonons, while the sub-THz optical conductivity follows an Arrhenius law with activation energies of 94 meV (Sc-doped) and 72 meV (Y-doped)—values that match macroscopic migration barriers and therefore cement proton–phonon coupling as the ultimate mobility bottleneck. Such dynamic information converts IR from a static probe into a real-time gauge of lattice drag.

Layered onto these mechanistic insights are clear practical advantages. FTIR or DRIFTS instruments are pervasive, require only powders, pellets, or thin films, and leave the sample intact.^343^ Even trace hydration generates a detectable O–H flag long before the microbalance registers weight gain, as evidenced in cubic BaTiO_3_ or in high-entropy perovskites where a nascent 3.3 μm band grows within minutes of exposing the material to 5% H_2_O at 400 °C.^338^ Because the O–H stretch is intrinsically polar, its intensity scales qualitatively with proton content, and isotopic substitution (H_2_O → D_2_O) shifts the peak by the expected $\sqrt{2}$ factor,^338,341^ cleanly separating overlapping bands and confirming assignments. Purpose-built environmental cells, widely adopted since Kreuer’s early *operando* studies,^50^ now permit automatic pH_2_O and temperature sweeps, embedding kinetic hydration curves directly into the vibrational data stream.

The same qualities, however, impose limitations. The essential broadness that makes the O–H envelope conspicuous can cloak multiple species; deconvolution demands high signal-to-noise and model assumptions, as Arai’s four-Gaussian fits^341^ or Naumovska’s hydrated–dehydrated subtraction routines^338^ demonstrate. Many protonic conductors scatter or absorb mid-IR radiation strongly. DRIFTS mitigates this but converts absorbance into a reflectance pseudoabsorbance that is harder to quantify, a complication explicitly acknowledged when comparing BZCYYb1711 pellets to thin films.^344^ Adventitious carbonates introduce bends near 1450 cm^–1^, ambient moisture adds wagging modes at 1630 cm^–1^, and incomplete nitrate burnout leaves artifacts around 2030 cm^–1^—all of which Mahmud et al.^339^ had to suppress by stringent drying and baseline subtraction. At temperatures above 600 °C, thermal radiation from the sample bathes the detector, forcing chopped-beam optics or rapid-scan acquisition, while sintering or phase transformations can shift the overall baseline, complicating kinetic analysis.

A similar reconfiguration of the proton environment under interfacial constraint was revealed by Aparnev et al.,^345^ who investigated composite electrolytes based on CsH_2_PO_4_ modified with highly dispersed ZnSnO_3_ additives. Pure CsH_2_PO_4_ exhibits a sharp and narrow O–H stretching vibration around 2500 cm^–1^, corresponding to its extended hydrogen-bonded network that stabilizes a high conductivity phase above 230 °C. However, upon introducing only 20 vol % ZnSnO_3_, the FTIR spectrum reveals a clear splitting and slight blue shift of this band, now consisting of several distinct, broader components. This vibrational fragmentation directly indicates that the additive disrupts the cooperative hydrogen-bond network, introducing spatial heterogeneity in the local bonding environment. Moreover, the emergence of higher-frequency components signifies that protons experience weaker, more asymmetric hydrogen bonds at the ZnSnO_3_ interface. These changes correlate with a striking 2.5-order-of-magnitude enhancement in proton conductivity at sub-200 °C, suggesting that the additive-induced field effects not only alter bonding topology but create new fast-conduction pathways inaccessible in the bulk phase. This case underscores how vibrational spectroscopy can capture subtle yet functionally critical distortions in the O–H network that enable ionic mobility under otherwise inactive thermal regimes.

Quantifying proton stoichiometry remains the final frontier. Extinction coefficients for lattice hydroxides vary with site symmetry, so absolute concentrations usually require cross-calibration against gravimetric water uptake or ^1^H NMR. Nevertheless, the correlations between ν­(O–H) position, band shape, and proton mobility are remarkably consistent across various systems—such as BaZr_0.36_Ce_0.54_Y_0.1_O_2.95_,^339^ BaCe_0.7–x_Sm_x_Zr_0.2_Y_0.1_O_3−δ_,^340^ BaZr_1–x_Sc_x_O_3−δ_,^338^ and hexagonal BaTiO_3_ ^315^—that the semiquantitative nature of IR spectroscopy rarely constrains its diagnostic utility. Indeed, when such spectral descriptors are plotted against conductivity or activation energy, they fall on universal trends, underlining the method’s ability to bridge local bonding and macroscopic performance.

##### 4.1.5.2 Raman Spectroscopy

Raman spectroscopy enters the vibrational-spectroscopy toolbox at the point where the dipole-selection rules that govern IR absorption fall silent. Because Raman scattering responds to changes in the electronic polarizability of a vibrating bond, centrosymmetric or highly symmetric modes that are IR-inactive can be bright in the Raman spectrum, while—as every practitioner of protonic IR knows—the strongly polar O–H stretch that dominates IR can appear only as a faint, broad shoulder in Raman. This complementarity is especially welcome in proton-conducting oxides, whose ideal perovskite aristotype (*Pm*3m) carries no first-order Raman modes at all; once protons or dopants arrive and break that symmetry, Raman records both the newly allowed lattice modes and, under favorable conditions, the elusive O–H stretch, thereby completing the picture sketched by IR.

The mechanistic leverage of Raman emerges most clearly in *operando* work. A landmark study on Y-doped barium cerate followed thin films of BaCe_0.9_Y_0.1_O_3−δ_ through a heating and humidification cycle inside a dual X-ray/Raman furnace.^316^ Above 650 °C, the Raman spectrum contained only lattice phonons; when the sample cooled in 2 vol % H_2_O, two broad bands near 3450 cm^–1^ materialized, signaling the uptake of protons into two distinct local environments yet leaving the low-frequency perovskite vibrations essentially unchanged. The juxtaposition of stable lattice peaks and emerging O–H bands confirmed that hydration inserts OH^–^ on pre-existing vacancy sites without restructuring the framework—knowledge that dovetailed with conductivity and TGA data collected in parallel.

Where those BaZrO_3−δ_ films preserved long-range order, Sc-doped BaTiO_3_ moves in the opposite direction: local disorder activates Raman modes that would be forbidden in a perfect cubic cell (Figure 27b).^315^ Naumovska et al.^338^ resolved three proton sites in hexagonal BaZr_1–x_Sc_x_O_3–x/2_ and only one in the cubic analog. The stronger, lower-symmetry hydrogen bonds of the hexagonal phase drove its conductivity 2 orders of magnitude lower. Crucially, Raman peak energies and intensities mirrored the calculated hydrogen-bond geometries, whereas IR could track only the broad O–H band. The study established a logical bridge: proton site multiplicity → hydrogen-bond strength → phonon signatures → macroscopic transport.

The same structure–defect–transport chain underpins the Raman analysis of Y-doped SrZrO_3_. Shkerin et al.^346^ varied both Y content and A-site Sr deficiency; Raman shifts in Ag modes tied to Sr displacements reported how part of the dopant migrated from the intended B site to the A site when Sr fell below stoichiometry. Because A-site Y eliminates oxygen vacancies that would otherwise hydrate, the spectral changes correlated quantitatively with the downturn of proton conductivity at large Sr deficiency. Raman therefore exposed cation partitioning that X-ray Rietveld refinement could not resolve (Sr, Zr, and Y have similar scattering factors), linking subtle peak shifts to practical electrolyte design rules.

Moving from bulk ceramics to engineered heterostructures, Du et al.^347^ designed a TiO_2_–SrTiO_3_@TiO_2_ yolk–shell particle whose internal percolating interface network carries protons at 0.084 S cm^–1^ and 550 °C. Raman mapping picked out strong HO–Sr vibrations exactly along those interfaces, visually confirming that the heterointerface—not the TiO_2_ or SrTiO_3_ core alone—hosts the dominant proton pathway.^338^ Such spatially resolved spectra exploit the micron-scale focus of a confocal Raman microscope, an advantage that neither IR transmission nor neutron scattering can match.

Thin films impose their challenges: weak scattering volume, fluorescence from substrates, and the necessity to deconvolute film and support signals. Yang et al.^316^ tackled these with surface-enhanced Raman spectroscopy (SERS) and non-negative matrix factorization (NMF) on epitaxial Ba­(Zr, Ce, Y)­O_3−δ_ films. After NMF separation, the intensity ratio between BO_6_-octahedra and A-site motions served as a symmetry gauge; lower symmetry in the film, preserved across Ce content, coincided with higher activation energy than in bulk pellets, quantitatively demonstrating the symmetry–*E*_a_ link suggested by Perrichon’s BaTiO_3_ work.^315^ The SERS/NMF workflow also illustrates how chemometric tools offset the intrinsically low Raman cross-section of cubic perovskite thin films.^346^

Chemical durability—a prerequisite for reversible solid-oxide cells—is another arena where Raman excels. In tridoped BaZr_0.2_Ce_0.5_Y_0.1_Gd_0.1_Pr_0.1_O_3−δ_, Rajendran et al.^348^ compared XRD and Raman patterns before and after 200 h in 50 vol % steam at 600 °C. The absence of new hydroxide or oxide peaks in Raman confirmed outstanding moisture stability, while the persistence of disorder-related modes signaled that the lattice vacancies essential for hydration survived the test.^316,347^ Conductivity remained 15 mS cm^–1^ under the same conditions,^348^ again tying vibrational fingerprints to functional metrics.

Across these case studies, a coherent theme emerges: Raman detects protons directly through weak high-wavenumber O–H bands under conditions of sufficient proton concentration (e.g., BaZr_0.8–x_Ce_x_Y_0.2_O_3−δ_ films^316^) and indirectly through lattice-mode shifts and the activation of forbidden modes when structural disorder is the primary consequence of hydration (e.g., Sc-doped BaTiO_3_,^315^ SrZrO_3_,^346^ nanoheterointerfaces,^347^ and BaCe_0.5_Zr_0.2_Y_0.1_Gd_0.1_Pr_0.1_O_3−δ_ films^348^). That dual sensitivity underpins several practical strengths:

Raman’s host-lattice acuity unmasks symmetry breaking from dopants, vacancies, or interfacial strain that would leave IR silent; the Sc-doped BaTiO_3_ and Y-doped SrZrO_3_ examples translate those spectral fingerprints into conductivity trends. Its different selection rules complement IR, allowing mutual assignment of complex spectra—Perrichon’s combined IR/Raman fitting of proton sites is exemplary. Thanks to fused silica or sapphire windows and the negligible Raman cross-section of gaseous H_2_O, *in situ* cells comfortably reach 700 °C under humid atmospheres; the BaCeO_3−δ_ chamber experiment relied on exactly this configuration.^348,349^ Optical microscopy affords micron-scale mapping, crucial for the yolk–shell electrolyte. Finally, Raman adapts to bulk pellets, thin films, and even operating devices, needing only a laser path.

These virtues are balanced by limitations that the same literature makes explicit. The BaCeO_3−δ_ study needed long acquisition times and carefully chosen laser power because the OH band is weak and the sample is prone to laser-induced dehydration. Fluorescence from trace transition-metal impurities can swamp the already faint proton signal;^316^ switching to red or near-IR excitation is often mandatory, though at the cost of reduced Raman efficiency. Spectra featuring overlapping modes require chemometric or computational assistance before drawing structural conclusions.^348^ Quantitative proton counting remains elusive, as peak intensities scale with polarizability rather than stoichiometry. Rajendran et al.^348^ therefore combined Raman with impedance and XRD to bracket hydration levels. Finally, sophisticated high-temperature cells, SERS substrates, or NMF software raise the bar for experimental setup and data interpretation.

Yet, as the examples above illustrate, when those challenges are met, Raman spectroscopy weaves a logically continuous narrative from the atomic motion of protons through lattice distortions and defect chemistry to the macroscopic performance targets of reversible solid oxide cells.

#### 4.1.6 Neutron-Related Techniques

##### 4.1.6.1 Neutron Diffraction

NPD derives its unrivaled sensitivity to proton chemistry from the fact that scattering amplitudes are determined by nuclear rather than electronic structure: the coherent length of ^1^H is −3.74 fm, large and negative, that of ^2^H is +6.67 fm, comparable to O, Zr, or Ce. Swapping H for D therefore inverts the sign of the density and boosts contrast, letting even medium-resolution powder patterns resolve O–H/D bonds that vanish in X-ray maps. Basbus and co-workers exploited this contrast in a flowing-D_2_O experiment on rhombohedral BaZr_0.4_Ce_0.4_Y_0.2_O_3−δ_, showing full deuteron occupancy at 150 °C, a smooth fall beginning at 400 °C, and continuing through the rhombohedral-to-cubic transition at 520 °C;^317^ the unit cell contracted by 0.13% with dehydration, quantifying the “chemical contraction” that mirrors proton loss and clarifying that the conductivity crossover stems from deprotonation rather than symmetry change. Time-resolved collections gave a first-order hydration rate constant of 6.7 × 10^–4^ s^–1^ at 400 °C, proving that bulk exchange kinetics can be extracted directly from Bragg intensities.

Because hydrogen’s spin-incoherent cross-section is almost an order of magnitude larger than its coherent term, polarized-NPD was developed to disentangle the two. xyz-polarization analysis of BaZr_1–x_In_x_O_3–x/2_ (x ≤ 0.275) separated a purely spin-flip signal proportional to total hydrogen and revealed a monotonic rise in H with increasing In^3+^, accompanied by systematic lattice expansion and (110) peak broadening that betrays cation-vacancy disorder;^350^ the absolute H inventory agreed with thermogravimetric water uptake to within 0.03 H per formula, validating polarized-NPD as a calibration-free method that leaves the sealed can unmodified for subsequent quasielastic or inelastic neutron studies.

High-flux time-of-flight (TOF) instruments then extend the structural reach to complex, multicomponent perovskites where laboratory X-rays insinuate cubic symmetry. For BaZr_0.3_Ce_0.5_Y_0.1_Yb_0.05_Zn_0.05_O_3−δ_, TOF-NPD detected orthorhombic Pbnm octahedral tilts and located 2.715 oxygens per formula—close to the 2.75 ideal—while correlating a 1.06% mass loss between 200 and 1000 °C with a contraction of the *b* axis on dehydration;^351^ the structural map clarifies why the same ceramic delivers 649 mW cm^–2^ at 700 °C in an all-protonic cell.

Beyond simple ABO_3_ perovskites, neutron contrast uncovers vacancy-ordered superstructures that underpin outstanding proton transport. A 17.18 Å *Fd*3̅*m* polytype of Ba_3_WO_6_, whose crystal structure was solved by joint TOF-NPD and synchrotron XRD, exhibits a three-dimensionally connected network of Ba and O vacancies (Figure 28a,b). This defect lattice is sufficient to sustain long-range proton diffusion in the absence of aliovalent dopants. Variable-temperature synchrotron XRD (rather than NPD) shows a reversible softening and partial decomposition of the oxygen-deficient layers between 600 and 1000 °C, thereby defining a thermodynamic window in which the cubic phase is stable and delivers a proton conductivity of 1.75 × 10^–2^ S cm^–1^ at 500 °C.^352^

**Figure 28:**
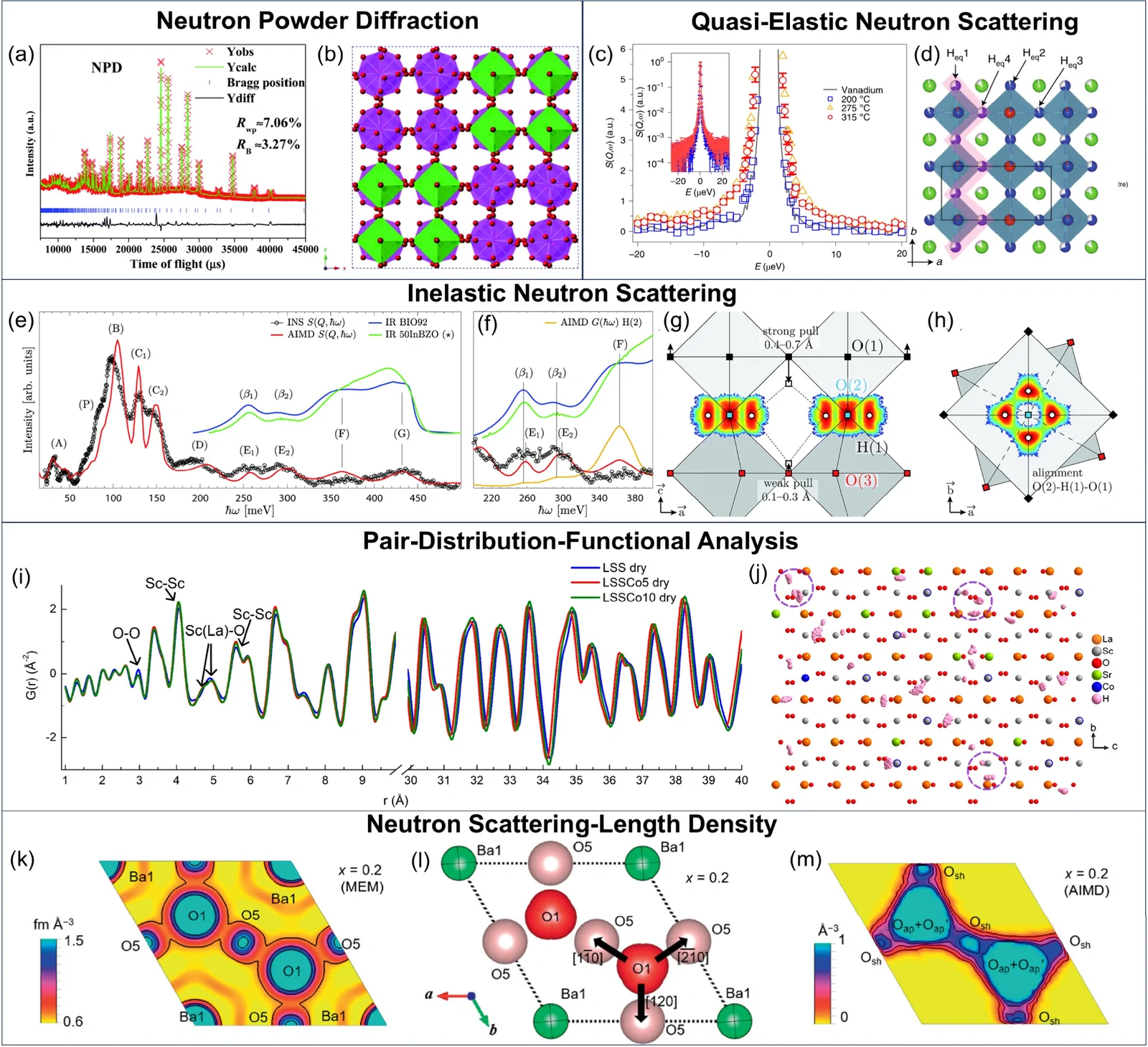
Neutron-scattering techniques for probing local structure and ion dynamics: (a) Rietveld refinement of NPD data for cubic Ba_3_WO_6_. (b) The refined crystal structure of α-Ba_3_WO_6_ (Ba omitted for clarity). Reproduced with permission from ref 352. Copyright 2022 RSC. (c) Q-averaged QENS spectra measured at 200 °C (blue), 275 °C (orange), and 315 °C (red), compared with that from a vanadium cell (gray line). (d) Crystal structures for β-phase Ba_1.75_LiH_2.7_O_0.9_. The green, blue, and red balls represent Ba, H, and O atoms, respectively. The highlighted regions are localized H vacancies. Reproduced with permission from ref 357. Copyright 2022 MDPI. (e) Experimental INS and IR spectra of hydrated Ba_2_In_2_O_5_(H_2_O)_0.92_ compared with calculated spectra. (f) An enlarged view of characteristic vibrational bands. (g–h) Schematic illustrations of the hydrated brownmillerite structure, (g) showing the amplitude of displacement of the acceptor oxygen atoms [O(1) and O(3)] under the pull of the hydrogen bonds of paired H(1) protons, (h) showing the alignment of the O(2)–H(1) covalent bond with the edges of the O(1)_4_O­(2)_2_ octahedra. Reproduced from ref 180. Available under a CC-BY-NC 3.0 license. Copyright 2019 RSC Perrichon et al. (i) X-ray PDFs of dried La_0.9_Sr_0.1_ScO_3−δ_, La_0.9_Sr_0.1_Sc_0.95_Co_0.05_O_3−δ_, and La_0.9_Sr_0.1_Sc_0.9_Co_0.1_O_3−δ_ in the range of 0–40 Å. (j) Illustration of trajectories of protons within a 5 × 5 × 5 supercell, obtained by MD at 298 K for 0.5 ns. Each pink circle represents a place in the proton’s trajectory, in a 0.5 ns simulation. Dashed rings highlight protons with two or more oscillation points. Reproduced from ref 359. Copyright 2022 American Chemical Society. (k) NSLDs derived via the maximum-entropy method on the ab plane at z = 0. (l) Refined crystal structure on the ab plane at z = 0 using neutron diffraction data. Arrows denote the directions of oxide-ion O1-to-O5 migration. (m) Corresponding spatial probability-density distribution of oxide ions on the ab plane calculated by AIMD simulations, where the probability density distribution in the 3 × 3 × 1 supercell is averaged to 1 × 1 × 1. Reproduced from ref 318. Copyright 2023 American Chemical Society.

Fluorite-derived electrolytes profit equally. TOF-NPD of Ce_1–x_Ln_x_O_2–x/2_ (Ln = La, Nd, Sm, Gd, Yb; 0.2 ≤ x ≤ 0.6) captured the onset of an *Ia*3̅ C-type superstructure once x > 0.4;^353^ for La_0.6_, the room-temperature pattern required a biphasic—fluorite + C-type—model that evolved to 97 wt % C-type at 800 °C. Impedance revealed that Gd^3+^ and Sm^3+^ maximize oxide-ion conductivity near x ≈ 0.2, whereas large La^3+^ promotes proton transport when C-type order dominates; thermogravimetric H_2_O uptake followed the same compositional trend, tying structure, hydration, and mixed H^+^/O^2–^ conduction into a single narrative.

When neutron and X-ray contrasts for B-site cations are nearly degenerate, resonance techniques join the toolkit. In Nb/Mo layered perovskite Ba_7_Nb_4_MoO_20−δ_·0.15 H_2_O,^318^ Nb K-edge resonant powder diffraction fixed Mo occupancy at the M2 site, ^95^ Mo MAS NMR confirmed the assignment, and TOF-NPD provided the complete oxygen framework plus two interstitial H sites that flank the c′ layers, revealing how ordered Mo centers and interstitial oxygens cooperate to form low-barrier ion pathways.

TOF-NPD combined with maximum-entropy density mapping of hexagonal Ba_7_Nb_3.9_Mo_1.1_O_20.05_ located an interstitial O5 site that hosts 0.54 O eq^354^ at 800 °C; bond-valence analysis gave a 0.21 eV barrier for O1–O5 interstitially, and isotope-exchange depth profiling measured D* = 7.25 × 10^–9^ cm^2^ s^–1^ at 800 °C—three orders higher than YSZ—validating the neutron-derived pathway.

In mixed-anion oxyhalides, heavy halogens blur X-ray maps, yet neutrons register oxygen and hydrogen sharply. Density reconstructions of Bi_1.9_Te_0.1_LuO_4.05_Cl identified overstoichiometric oxygens trapped inside triple-fluorite blocks; bond-valence landscapes and ab initio MD concur that oxide ions migrate interstitially with a 0.577 eV barrier, consistent with 1.5 × 10^–3^ S cm^–1^ at 310 °C and nearly pO_2_-independent conductivity.^355^

Proton conductors based on donor-stabilized vacancies also owe their mechanistic insight into neutrons. Cubic BaSc_0.8_Mo_0.2_O_3–δ_ shows disordered vacancies over all three oxygen sites from 25 to 800 °C, no vacancy ordering, and 0.22 H per formula after hydration at 300 °C spread across the vacant sites;^170^ ab initio MD yields high proton mobility free from dopant trapping, and pO_2_-independent σ reaches 0.01 S cm^–1^ at 320 °C, again in the “Norby gap,” all rationalized by the vacancy topology captured with NPD.

More recent neutron stories probe exotic structural motifs. Yasui’s study employed TOF-NPD, pair-distribution analysis, and AIMD to reveal that BaZr_0.6_Y_0.2_Yb_0.2_O_3−δ_ hydrates not by filling classical O vacancies but by stabilizing transient O_4_H_10_ clusters whose geometry was extracted from the real-space PDF; the clusters dissolve above 350 °C, matching the onset of long-range proton diffusion measured by impedance.^356^ Ueno et al.^355^ pushed neutron reach to oxyhalide triple-fluorite layers, combining high-resolution NPD with maximum-entropy density to map oxide-ion interstitials, then followed the same specimen by neutron spin–echo measurement to quantify the 70 ps jump time of the ions along the *b* axis, cementing the link between static site occupancy and dynamic diffusion. García and co-workers returned to the fluorite motif, showing by variable-temperature NPD that rare-earth substitution in CeO_2_ favors a nanoscopic C-type framework whose partial ordering, rather than average symmetry, sets the proton diffusion barrier; defect-specific conductivity extracted from ^2^H tracer diffusion corroborated the neutron-derived mechanism.^353^

These case studies illustrate why neutron diffraction remains indispensable even where complementary techniques excel. It reveals the exact lattice positions of statistically sparse H/D atoms, quantifies their occupancies or totals via polarization analysis, and tracks subtle octahedral tilts, vacancy order, or interstitial oxygen content that modulate migration barriers. Neutrons penetrate centimeters of dense ceramics, so they probe the same pellets that ultimately power reversible solid-oxide cells, and the *in situ* capability under humid, reducing, or oxidizing atmospheres lets researchers synchronize structure, hydration, and electrochemical performance in real time. Limitations persist: the need for gram-scale samples and access to high-flux reactors or spallation sources, the background from ^1^H spin-incoherence that often mandates deuteration or p-analysis, and the fact that diffraction averages over mobile protons whose instantaneous positions smear into elongated densities. Yet the continuing advance of brighter sources, large-area position-sensitive detectors, resonant contrast, and combined diffraction–spectroscopy experiments keeps expanding the scope, ensuring that NPD retains a central role in the structural science of proton- and oxide-ion conductors for RSOCs applications.

##### 4.1.6.2 Neutron Scattering

Neutron scattering is unique among spectroscopic probes in that it records, in the same experiment, both the momentum transfer Q and the energy transfer ℏω. Because hydrogen possesses an incoherent scattering cross-section of ∼ 80 barn that dwarfs those of most cations, a proton-conducting solid effectively “draws” its dynamic map under the beam: elastic diffraction spots report where protons prefer to sit, while inelastic and quasi-elastic wings reveal how fast—as a function of temperature, local bonding, and crystal chemistry—those same protons (or hydride anions) can move. Recent neutron studies, therefore, form an unbroken thread that runs from the earliest hydration of brownmillerites to the discovery of cooperative, dimer-mediated transport in complex hexagonal frameworks, and even to emerging biobased electrolytes.

QENS remains the premier method for quantifying picosecond proton dynamics (Figure 28c,d). In hydrated chitosan, Hirota et al.^357^ resolved two jump-diffusion processes: a fast water-like hop (D ≈ 10^–5^ cm^2^ s^–1^ at 283 K) and a slower backbone hydrogen motion; both share an activation energy of 0.30 eV, mirroring the Arrhenius slope of the σ_bulk_ and demonstrating that even soft polymer matrices can host Grotthuss-type transport when sufficient water is retained. Translating the same methodology to crystalline oxides, Basbus et al.^317^ followed deuterium occupancy in BaCe_0.4_Zr_0.4_Y_0.2_O_3−δ_ while recording *in situ* NPD. They observed that proton content drops sharply above ∼ 520 °C—exactly the temperature where a rhombohedral-to-cubic transition is triggered—yet QENS shows increased mobility of individual protons that remain, explaining why proton conduction dominates below 600 °C even though carrier density is falling.

Where QENS supplies jump frequencies, INS pinpoints the vibrational fingerprint of each proton site. In Ba_2_In_2_O_5_(H_2_O)_x_, Perrichon et al.^180^ combined INS and IR spectroscopy to demonstrate two well-defined proton sublattices: H(1) species bonded intraoctahedrally and H(2) species involved in linear interoctahedral O–H···O geometries. An unusually red-shifted O–H stretch at 255 meV revealed a minority of H(2) protons trapped in ultrashort hydrogen bonds, a local distortion later traced by AIMD to transient brownmillerite-like layers persisting even in the “pseudocubic” phase (Figure 28e–h). A similar spectroscopic logic now underpins hydride conductors: in the K_2_NiF_4_-type oxyhydride Ba_2_LiH_3_O_3−δ_, INS registers a pronounced softening of the high-energy H- stretch as temperature approaches 315 °C, the point where long-range hydride diffusion and a σ ≈ 10^–2^ S cm^–1^ plateau appear—experimental proof that lattice-wide disorder, rather than simple thermal expansion, unlocks ultrafast anion motion.^358^

Total scattering and PDF analysis extend the reach of neutrons into real space, capturing correlated disorder that diffraction alone averages out. Plekhanov et al.^359^ combined neutron PDF with reverse-Monte Carlo modeling and nonconstant-force-field MD to show that introducing 5–10 mol % Co into La_0.9_Sr_0.1_ScO_3−δ_ increases the number of proton-binding sites while simultaneously flattening the potential-energy landscape (Figure 28i,j). PDF peaks broaden and split, indicating a distribution of Sc/Co–O bond lengths that suppresses deep traps and renders proton diffusion markedly anisotropic under bias. The same PDF strategy underlies Murakami’s discovery that, in Ba_7_Nb_3.8_Mo_1.2_O_20.1_, excess oxygen condenses into corner-sharing (Nb/Mo)_2_O_9_ dimers whose cooperative break-reform motion carries both O^2–^ and H^+^ along two-dimensional highways;^318^ AIMD confirms migration barriers <0.35 eV and experiment measures a remarkable 11 mS cm^–1^ dual-ion conductivity at 537 °C. Hidden chemical order in the parent Ba_7_Nb_4_MoO_20−δ_ phase, invisible to conventional diffraction, was subsequently quantified by combining resonant X-ray scattering with neutron data, revealing Mo preference for sites bordering the oxygen-deficient layers and providing a crystallographic basis for the hydration selectivity of the later Mo-rich analogue.^356^

Beyond time-averaged diffraction and atom-pair statistics, maximum-entropy reconstruction of the neutron scattering-length density (NSLD) now provides real-space probability maps that pinpoint interstitial species and visualize percolation corridors. In hexagonal Ba_7_Nb_3.8_Mo_1.2_O_20.1_, Murakami et al.^318^ converted TOF-NPD data acquired at 800 °C into iso-surfaces of NSLD derived via the maximum-entropy method, and identified contiguous density distributions between the lattice O1 and interstitial O5 sites (Figure 28k–m). This connected “tube” mirrors the AIMD probability distribution and confirms the two-dimensional O^2–^/H^+^ bucket-relay pathway that underlies the 11 mS cm^–1^ conductivity at 537 °C. Suzuki et al.^354^ applied the same analysis to W-doped Ba_7_Nb_4_MoO_20−δ_, showing that aliovalent W^6+^ fills the O5 pocket, truncates the NSLD corridor, and simultaneously suppresses proton transport while enhancing oxide-ion conduction. The power of NSLD mapping is not confined to perovskites: in triple-fluorite-layer oxyhalide Bi_1.9_Te_0.1_LuO_4.05_Cl, Ueno et al.^355^ visualized interstitial O2 density that links fluorite blocks into a three-dimensional superhighway—information inaccessible to QENS or conventional PDF alone. Because NSLD treats hydrogen and heavier cations on equal footing, it bridges the gap between dynamic QENS/INS parameters and the static defect chemistry captured by PDF, delivering an atom-resolved blueprint for engineering vacancy concentration, interstitial site energy, and long-range connectivity in next-generation protonic electrolytes.

Finally, the full power of the neutron toolbox is realized when dynamic and static experiments are stitched together in a single mechanistic tapestry. In hydrated Ba_7_Nb_3.8_Mo_1.2_O_20.1_, temperature-resolved NPD tracks the gradual dehydration of the oxygen-deficient c′ layer, INS fingerprints the two inequivalent proton environments that evolve during that loss, and QENS—once vacancy concentration is fixed—extracts the Arrhenius prefactor for the surviving hydrogens. Such multimodal workflows now allow researchers to pass jump lengths and residence times from QENS directly into AIMD force-fields; feed site occupancies from INS into cluster-expansion models of hydration isotherms; and transfer PDF-derived defect networks into continuum simulations of tortuosity in triple-conducting oxides. Case studies already demonstrate practical benefits: Co-doping of La_0.9_Sr_0.1_ScO_3−δ_, identified by PDF/QENS as trap-free chemistry, is now being scaled into thin-film oxides,^360,361^ while the dimer-mediated mechanism observed in Ba_7_Nb_3.8_Mo_1.2_O_20.1_ inspires targeted searches for other cations capable of forming flexibly coordinated M_2_O_9_ species.^318^ Even the polymer chitosan example impacts oxide design by highlighting the kinetic symmetry between sluggish hydration water and backbone protons, reinforcing the view that soft or dynamically disordered sublattices can accelerate long-range hopping without sacrificing thermodynamic stability.

In short, neutron scattering has matured from a descriptive method into a predictive engine for proton-conductor discovery: QENS dissects motion, INS fingerprints binding, PDF exposes hidden order, and NPD quantifies both phase evolution and site occupancy. Together, these techniques continue to refine our mechanistic understanding of proton uptake—from brownmillerites and perovskites to hexagonal and mixed-anion frameworks—while simultaneously providing the quantitative parameters needed to design the next generation of reversible solid-oxide-cell electrolytes.

##### 4.1.6.3 Prompt-Gamma Activation Analysis

Neutron-based probes capable of “seeing” hydrogen inside dense ceramics fall into three broad families. Neutron diffraction reconstructs nuclear density maps from Bragg intensities and therefore localizes protons crystallographically; inelastic or quasi-elastic neutron scattering tracks their dynamics through energy- or momentum-resolved spectra; prompt-gamma activation analysis (PGAA) forgoes momentum information and instead counts the γ-quanta released when a nucleus captures a neutron. Because those γ lines are isotope-specific and their integrated intensity is directly proportional to the number of emitting nuclei, PGAA converts the otherwise elusive hydrogen sublattice into an absolute, bulk concentration without modeling, Rietveld refinement, or calibration curves. The following discussion keeps that quantitative focus while briefly sign-posting the structural (diffraction) and dynamical (scattering) complements.

PGAA irradiates the sample with a cold- or thermal-neutron beam; ^1^H, with its enormous capture cross-section in the 1/v regime, absorbs a neutron to form deuterium in an excited state and instantaneously emits a 2.223 MeV γ-photon. High-purity germanium detectors register that line with keV resolution, while additional peaks from O, Ba, Zr, dopants, or adventitious B and Cl appear at their characteristic energies. Every count, therefore, carries two pieces of information: identity and quantity. Cold neutron guides at dedicated instruments such as the Budapest Research Reactor’s PGAA station maximize flux in the low-energy tail, boosting the H signal and pushing detection limits into the tens-of-μg range—ppm in many oxides—without sacrificing matrix penetration.^362^ The National Institute of Standards and Technology has taken the same philosophy further: the next-generation CN-PGAA spectrometer on guide NG-D couples a 10-fold flux increase with hydrogen-free construction and active Compton suppression, achieving <5 μg H g^–1^ for silicon, steels, or ceramic standards and allowing kilogram-scale samples or entire electrochemical cells to be mounted in evacuated chambers.^363^

Quantitative power was first demonstrated for protonic perovskites by Jones et al.,^364^ who compared undoped BaPrO_3_, BaPr_1–x_Y_x_O_3−δ_, and benchmark BaCe_0.9_Y_0.1_O_3−δ_. Cold-neutron PGAA showed that humidified BaCe_0.9_Y_0.1_O_3−δ_ incorporated 0.87 ± 0.16 H per Y—within experimental error of the ideal vacancy-filling model—whereas BaPr_1–x_Y_x_O_3−δ_ took up three to five times more protons than dopant cations. Concomitant XPS revealed a redox shift from Pr^4+^ to Pr^3+^, leading the authors to propose “self-doping” via eq 20,^364^ an insight only accessible because PGAA returned an unequivocal hydrogen inventory uncontaminated by the oxygen loss that would plague thermogravimetry.^350^

20 $${\mathrm{Pr}}^{4+}+\frac{1}{2}H_{2}O+O_{O}^{\times }\to {\mathrm{Pr}}^{3+}+{\mathrm{OH}}_{O}^{\cdot }+\frac{1}{4}O_{2}$$

The method’s authority in absolute hydrogen counting makes it the yardstick against which diffraction refinements are gauged. Karlsson et al.^350^ explored a BaZr_1–x_In_x_O_3–x/2_ series (x = 0–0.275) and recorded hydrated powder patterns with neutron diffraction plus full polarization analysis. Occupancies extracted from Rietveld fits agreed within 5% with the totals delivered independently by PGAA, validating both the structural model and the instrumental corrections applied to the polarized data. Such cross-checks are invaluable whenever the weak, incoherent background of hydrogen clouds affects the diffraction profile.

Because every captured neutron produces a γ cascade, the complete elemental makeup can be read out simultaneously. Routine PGAA spectra therefore verify bulk stoichiometry—Ba:Zr, Zr:In, Ce:Y—and expose low-level contaminants whose capture lines are intense (B, Cl, Sm, Gd). In protonic ceramics that are prone to BaO volatilization, this internal mass balance reveals subtle cation loss invisible to weight-change methods; during doping campaigns, it confirms whether nominal compositions survive sintering. The same shot can report hydrogen and a chlorine-rich impurity that might poison electrocatalytic interfaces, a diagnostic efficiency unmatched by any other single technique.

PGAA may also be performed quasi-*in situ*. A dense electrolyte pellet can be humidified at 500 °C, quenched in sealed Teflon, and transferred under dry N_2_ directly into the beam within minutes, freezing in the equilibrium proton content. Remeasurement after isothermal annealing or after DC polarization then tracks bulk hydration/dehydration loops without polishing surfaces or exposing the specimen to ambient moisture, scenarios difficult for vibrational spectroscopy and impossible for TGA under reducing atmospheres. When coupled to neutron imaging or to high-flux mapping stages now contemplated at NIST, the same nuclear principle could yield spatially resolved hydrogen tomograms, although the current centimeter-scale beam footprint still averages over gradients.

Several practical constraints temper those strengths. Access is limited to reactor or spallation facilities equipped with cold guides and low-background γ spectrometers; beam-time allocation inevitably slows iterative synthesis–characterization cycles. Spatial resolution is coarse, and the 2.223 MeV line does not distinguish chemical state—hydroxyls, molecular H_2_O, and trapped protons all contribute equally—so complementary diffraction, IR, or quasielastic scattering experiments remain essential for mechanistic assignments. Interference from elements with prodigious capture cross sections (Sm, Gd, Cd) can raise background, yet in the Ba–Zr–Ce systems typical of RSOCs electrolytes, those overlaps are modest, and modern deconvolution software resolves them routinely.^350^

Despite these caveats, PGAA’s unique ability to produce trace-level detection limits, direct proportionality between peak area and atom number, and matrix-independent calibration elevate it to the role of quantitative anchor for the entire neutron toolkit. Diffraction localizes where the protons sit, and scattering reveals how fast they hop, but PGAA tells exactly how many are there—and therefore how many charge carriers an electrolyte can mobilize—without touching the sample. When the objective is to benchmark hydration thermodynamics, validate defect models, or certify reference materials for impedance spectroscopy, no other technique currently matches its combination of penetration depth, universality, and precision.

#### 4.1.7 Nuclear Magnetic Resonance

Nuclear magnetic resonance spectroscopy—especially high-resolution proton (^1^H) solid-state NMR—has emerged as an indispensable tool for characterizing proton uptake, site distribution, and dynamics in proton-conducting oxide electrolytes. By directly observing ^1^H nuclei, NMR delivers three complementary dimensions of insight: chemical-shift-resolved identification of distinct proton environments, quantitative determination of proton concentrations via signal integrals, and dynamical information on local hopping and reorientation through relaxation and exchange experiments.

The sensitivity of ^1^H chemical shifts to local electronic and hydrogen-bonding environments allows unambiguous fingerprinting of proton species. In doped perovskites such as SrZr_0.8_Y_0.2_O_3−δ_ and BaZrO_3−δ_-based electrolytes, high-field ^1^H magic-angle-spinning (MAS) spectra resolve resonances at ∼ 4.4 ppm (physisorbed water), ∼ 3.5 ppm (surface- or vacancy-associated hydroxyls), and ∼ 0.7 ppm (lattice-bound protons) in the nominally dry state, assignments confirmed across multiple studies.^365^ Upon hydration, overall ^1^H signal intensity increases—directly reporting proton uptake—while chemical exchange and hydrogen-bond interactions broaden and shift peaks, coalescing the dry-state resonances into a broad feature near ∼ 2.3 ppm with a 4.6 ppm shoulder from physisorbed water. Variable-temperature MAS further reveals that above ∼ 125 °C, exchange slows or water desorbs, allowing the merged band to resplit into distinct hydroxyl and lattice signals, thereby tracing hydration thermodynamics and hydrogen-bond dynamics *in situ*.

Achieving such chemical-shift-resolved spectra in ceramic oxides requires overcoming strong homonuclear ^1^H–^1^H dipolar couplings and chemical-shift anisotropies. Ultrafast MAS at ≥ 60 kHz with small (1–2 mm) rotors averages orientation-dependent interactions, yielding subppm resolution without complex multiple-pulse schemes. While static ^2^H NMR can still probe second-moment line broadening and the onset of motional averaging, modern analyses of proton sites nearly always rely on MAS. Because *in situ* high-temperature operation under reversible solid oxide cell conditions remains technically prohibitive, most ^1^H MAS experiments are performed *ex situ* on powders hydrated under controlled pH_2_O and then quenched, providing a bulk-averaged snapshot of local proton environments as functions of hydration and temperature.

Beyond static assignments, ^1^H NMR relaxometry and two-dimensional exchange spectroscopy (EXSY) directly probe proton dynamics on the kHz–MHz time scale. Spin–lattice relaxation times measured from room temperature to ∼ 600 °C in Y-doped BaZrO_3−δ_ yield a local hopping activation energy of ∼ 13 kJ mol^–1^—substantially lower than the macroscopic activation barriers (∼16–45 kJ mol^–1^) obtained by impedance spectroscopy—highlighting the distinction between fast, localized Grotthuss-type steps sensed by NMR and long-range diffusion hindered by dopant trapping.^63^ Two-dimensional ^1^H–^1^H exchange nuclear Overhauser effect spectroscopy experiments detect cross-peaks between water and hydroxyl resonances, furnishing direct evidence of intersite proton transfer, while rotating-frame relaxation and ^2^H substitution experiments bracket jump rates and motional regimes.^356^

These experimental dynamic fingerprints dovetail with atomistic simulations. Torayev et al.^319^ combined static DFT and AIMD to link local inward/outward M–O–M bending motifs to proton transfer pathways in Y-doped BaZrO_3−δ_. Static DFT identified inward-bent configurations as low-energy, favoring reorientations, while DFT-AIMD revealed that outward bending leads to shorter hydrogen-bond lengths and enables intraoctahedral jumps only when O–O distances fall below a threshold—predictions consistent with NMR-observed exchange broadening and activation energies.^319^ Despite the computational intensity limiting long-range sampling, these simulations capture rare trapping/detrapping events and local motion, enriching the multiscale picture of proton diffusion.

Solid-state NMR of quadrupolar framework nuclei has further extended this mechanistic clarity by decoding cation order that dictates proton sites. Yasui et al.^356^ combined ^93^Nb triple-quantum MAS (3QMAS) and ^95^Mo MAS NMR with resonant XRD and gauge-including projector augmented wave (GIPAW)-DFT calculations to conclusively assign Mo exclusively to the tetrahedral M2 sites in hexagonal perovskite Ba_7_Nb_4_MoO_20−δ_·0.15 H_2_O. Two-dimensional ^93^Nb 3QMAS resolved three Nb environments corresponding to four crystallographic sites, while the singular ^95^Mo resonance at −47 ppm with a quadrupolar coupling constant lower than 2 MHz matched DFT-predicted shifts (−29 to −37 ppm) and couplings (0.36–0.90 MHz). Resonant XRD refinements quantified Mo occupancy as 0.50 at M2 and zero elsewhere, revealing hidden chemical order and directly linking framework distribution to both hydration enthalpy (ΔH_hyd_) and oxide ion migration barriers. The obtained ΔH_hyd_ is approximately −22.7 kJ mol^–1^, in contrast to +1.7 kJ mol^–1^ for disordered structural models, while the corresponding oxide ion migration barriers are 1.93 and 1.60 eV, respectively.

Integrating ^1^H MAS NMR, quadrupolar 2*D*/3QMAS, heteronuclear correlation, relaxometry, and GIPAW-DFT underpins a comprehensive NMR crystallography paradigm.^366^ ^1^H site populations and dynamics illuminate proton-dopant associations—“trapped” protons adjacent to Y^3+^ or Sc^3+^ dopants versus “free” lattice protons—and quantify association energies (∼29 kJ mol^–1^) consistent with Yamazaki et al.’s^63^ thermogravimetric-impedance–NMR study on Y-doped BaZrO_3−δ_. These trapped sites manifest as downward curvature in Arrhenius plots of macroscopic diffusivity, while NMR-derived spin–lattice relaxation time minima and relaxations capture local motions unaffected by traps.

Such mechanistic clarity delivers profound advantages. NMR directly detects protons—unlike diffraction methods that struggle with light atoms—and distinguishes OH– groups, H_2_O, and defect sites via chemical shifts. It quantitatively measures hydration levels through signal integrals, correlating with thermogravimetric uptake. Relaxometry and exchange techniques uniquely probe dynamics across the relevant frequency window. Multinuclear NMR (e.g., ^1^H→^89^Y or ^1^H→^45^Sc cross-polarization) further maps protons’ proximity to dopant cations, revealing trap fractions versus free populations. All these insights are obtained nondestructively, on the same sample, and can be combined with first-principles calculations for robust site assignments and dynamic interpretations.

Nevertheless, solid-state ^1^H NMR brings challenges. Magnetic impurities—e.g., unintended paramagnetic phases—can broaden or obliterate ^1^H signals, making sample purity essential. Ultrafast MAS and high-field magnets (≥9.4 T) are required for sufficient resolution and sensitivity, but such hardware is not yet ubiquitous. Ex situ powder preparation, while enabling controlled hydration states, cannot replicate true *operando* bias, gas gradients, and spatial proton distributions within working cells. Spectral complexity in disordered or heavily hydrated materials often demands complementary DFT-computed NMR parameters and low-temperature measurements to deconvolute overlapping signals. Even with ultrafast MAS, broad distributions of hydrogen-bond geometries can yield unresolved features, sometimes necessitating isotopic dilution (^2^H substitution) or dynamic nuclear polarization for enhanced resolution.

Despite these limitations, the synergy of high-resolution ^1^H MAS NMR, advanced multidimensional experiments, quadrupolar host-cation NMR, and computational crystallography has propelled our understanding of proton uptake and mobility in oxide electrolytes. By coupling direct observation of proton species and dynamics with mechanistic modeling, researchers can rationally tailor dopant chemistries, lattice distortions, and microstructures to minimize trapping, optimize hydration thermodynamics, and engineer continuous proton-conduction pathways. As NMR instrumentation advances toward higher fields, faster MAS, and *operando* designs—and as computation and machine learning frameworks grow ever more predictive—NMR will remain at the forefront of characterizing and guiding the next generation of proton-conducting electrolytes for RSOCs.^367^

#### 4.1.8 Positron Annihilation Lifetime Spectroscopy

Positron annihilation lifetime spectroscopy (PALS) offers a fundamentally different window into defect chemistry than neutron-based probes such as neutron scattering or diffraction, or nuclear techniques like PGAA. Whereas neutron methods interrogate atomic positions and dynamic correlations via scattering cross sections, and PGAA quantifies elemental composition through neutron-induced γ-emission, PALS detects open-volume defects by timing the annihilation of implanted positrons with electrons in the bulk lattice. In a PALS experiment, positrons from a radioactive source (commonly ^22^Na) rapidly thermalize (∼10^–12^ s), diffuse over tens to hundreds of nanometers, and then annihilate in regions of variable electron density. The measured lifetime distribution, deconvoluted into components τ_i_ with intensities I_i_, directly reflects vacancy-type defects, voids, and defect complexes that perturb local electron density—information that is largely “invisible” to diffraction or spectroscopic techniques.^368^

At its core, PALS leverages the sensitivity of positrons to charged defects. Positrons are repelled by positively charged vacancies (such as bare V_O_^••^) but become trapped at negatively charged centers—dopant–vacancy complexes, cation vacancies, or space-charge regions at grain boundaries. In acceptor-doped perovskites like Y- or Sc-doped BaZrO_3−δ_, a short “bulk” lifetime component (τ_1_ ≈ 150–180 ps) represents delocalized annihilation, while an intermediate component (τ_2_ ≈ 235–260 ps) arises from positron trapping at defect sites. A minor long-lived component (τ_3_ > 1 ns, I_3_ < 1%) corresponds to positronium formation in any residual voids. By fitting these components using a two-state trapping model, one extracts both the trap lifetime (characteristic of the open-volume size) and the trap rate (proportional to defect concentration), enabling quantitative comparisons across doping levels and treatment conditions.^325^

In proton-conducting perovskite electrolytes for RSOCs, hydration not only fills oxygen vacancies with hydroxyl groups but also alters the defect landscape in ways that profoundly influence proton mobility. Conventional probes (TGA, IR, Raman) confirm water uptake and O–H bonding, yet cannot directly reveal how many vacancy traps remain after hydration or how proton–dopant associations evolve. PALS, by contrast, sensitively reports the disappearance of vacancy-type traps: upon hydration, the intensity I_2_ of the defect-related lifetime τ_2_ systematically decreases, indicating that many positron traps (oxygen vacancies) have been filled by OH– and thus no longer support prolonged positron lifetimes. Simultaneously, the remaining τ_2_ may lengthen slightly, suggesting that deeper or more diffuse traps—dopant–OH clusters or residual grain-boundary free volumes—now dominate the positron annihilation spectrum.^325^

Recent studies illustrate these effects vividly. Urban-Klaehn et al.^369^ applied PALS to Y- and Sc-doped BaZrO_3−δ_ across doping levels from 0 to 60 mol % and under dry, low-temperature (300 °C), and high-temperature (600 °C) hydration conditions. They observed that initial Y-doping introduced a higher positron trapping rate and a longer τ_2_ than Sc-doping at equivalent levels, reflecting the larger ionic radius of Y^3+^ and its stronger association with oxygen vacancies. After low-temperature hydration, I_2_ in undoped and highly doped specimens dropped markedly, confirming vacancy passivation by OH^–^, while the slight τ_2_ increase pointed to the persistence of larger vacancy clusters or lattice relaxations around dopant–OH complexes. Such PALS-derived insights correlate with electrochemical observations of reduced proton mobility in Y-doped BaZrO_3−δ_ compared to its Sc-doped counterpart.^369^

Regalado Vera et al.^325^ further extended PALS applications by comparing dry and hydrated states in high-Sc-doped BaZrO_3−δ_, demonstrating that Sc-doping yields fewer or shallower positron traps and a more complete vacancy hydration at intermediate temperatures. Their data showed that proton trapping energies differ markedly: −0.296 eV for Sc vs −0.196 eV for Y, so that high Sc content, despite introducing more vacancies, ultimately promotes proton mobility by minimizing deep trap formation. DFT supported these experiments, indicating that higher hydration in Sc-doped samples lowers migration barriers despite increased trap density, whereas Y-doping’s stronger trap depths hinder conductivity gains.

PALS thus serves as an indispensable complement to chemical and structural probes. Its unmatched sensitivity—down to 10^14^–10^15^ cm^–3^ vacancy concentrations—and its bulk-penetrating nature (implantation depths of tens to hundreds of microns) allow nondestructive, *operando* studies of dense electrolyte pellets without elaborate preparation. Temperature- and atmosphere-controlled PALS can even monitor defect evolution *in situ*, revealing kinetics of hydration, dehydration, and thermally activated defect annealing.^325^

Yet PALS carries inherent limitations. It lacks chemical specificity—different defect types may yield similar lifetimes—so assignments often rely on complementary data or theoretical lifetime calculations. Multicomponent spectra fitting can be ill-conditioned when lifetimes are close or signal-to-noise is low, and trapping models assume simplified defect landscapes that may not fully capture the complexity of dopant–proton–vacancy interactions.^369^ Instrument timing resolution (∼150 ps) can mask subtle lifetime differences, and high-density samples are required to suppress positronium signals from pores or surface roughness. Moreover, specialized detectors and radioactive sources (or positron beams) limit accessibility compared to more ubiquitous techniques.^369^

Despite these challenges, the application of PALS to proton-conducting oxides has grown substantially since the first explorations in hydrated doped-BaZrO_3−δ_. By quantifying dopant-induced traps and their passivation upon hydration, PALS clarifies why certain dopants or processing routes yield superior conductivity, and guides strategies to reduce proton trapping—such as favoring Sc doping or optimizing sintering to minimize vacancy clusters. As PALS instrumentation advances (e.g., higher-resolution detectors, coincidence Doppler broadening) and as researchers integrate PALS with theoretical modeling, its role in unveiling the atomic-scale defect chemistry that governs macroscopic performance will only strengthen. In the development of next-generation protonic ceramic electrolytes for RSOCs, PALS stands as a definitive method for mapping the subtle interplay of vacancies, dopants, and protons that underlies true material optimization.

#### 4.1.9 H/D Exchange

Hydrogen–Deuterium isotope exchange has become a cornerstone technique for directly probing the uptake and movement of protonic defects in perovskite-type oxides used as electrolytes in RSOCs. When a proton-conducting oxide is exposed to a deuterium-containing atmosphere, lattice hydroxyl groups (O–H) can be replaced by O–D, and by monitoring how and where that replacement occurs, one gains quantitative insight into both how many protons the material can accommodate and how rapidly they can move. Early elastic-recoil-detection (ERD) experiments by Iizuka and co-workers on SrCe_0.95_Yb_0.05_O_3−δ_ demonstrated the power of this approach: after implanting D^+^ into the ceramic and then exposing it to H_2_O vapor at room temperature, deuterons were essentially completely replaced by protium, whereas the reverse H-for-D exchange under D_2_O vapor was negligible. This dramatic asymmetry—D→H exchange proceeding about 2 orders of magnitude faster than H→D—could not be reconciled with a simple round-trip diffusion model. Instead, Iizuka et al.^370^ proposed a one-way diffusion mechanism in which dissociative adsorption of H_2_O at the surface, rapid bulk diffusion of H, mixed-molecule recombination (HD formation), and surface release, followed by trapping of fresh H in vacant sites, conspire to favor the D→H direction so strongly at room temperature.

Building on this foundation, Ananyev and colleagues systematically explored H–D exchange over a broad temperature range (300–800 °C) in the lanthanum–strontium scandates La_1–x_Sr_x_ScO_3−δ_ (x = 0 and 0.04). By exposing protonated samples to D_2_ gas and tracking the temporal evolution of HD in the gas phase via mass spectrometry, they were able to extract heterogeneous surface-exchange rate constants for both protium and deuterium incorporation. Their kinetic model accounted explicitly for the thermodynamic isotope effect—deuterium’s lower zero-point vibrational energy makes O–D bonds slightly more stable than O–H bonds, and the kinetic isotope effect arising from the stronger O–D bond requiring higher activation energy to form and break. Quantitatively, they found that deuterium saturation levels exceeded those of protium, consistent with eq 21,^371^ and that the deuterium surface-exchange coefficient was about 30–50% lower than that for H at the same temperature, reflecting the kinetic isotope penalty.^372^ Their results not only validated isotope exchange as a tool for measuring equilibrium proton uptake but also furnished activation energies and pre-exponential factors for the surface reactions themselves, allowing for direct comparison across different perovskite compositions.

21 $$K_{\mathrm{therm}}=\exp\left[-\left(E_{\mathrm{ZP}}\left(\mathrm{OD}\right)-E_{\mathrm{ZP}}\left(\mathrm{OH}\right)\right)/\mathrm{RT}\right]$$

where K_therm_ is the thermodynamic isotope exchange constant between OD and OH species, E_ZP_(OD) and E_ZP_(OH) are the zero-point vibrational energies of the O–D and O–H bonds, respectively, R is the universal gas constant, and T is the absolute temperature in Kelvin.

Parallel efforts have demonstrated that isotopic tracing does not need to be confined to mass spectrometry or ERD. Jun Gao and co-workers leveraged *in situ* Raman spectroscopy to observe H–D exchange directly in nanostructured TiO_2_ membranes at low temperatures (<150 °C). By alternately flowing Ar–3% H_2_O and Ar–3% D_2_O over porous TiO_2_ pellets while monitoring the O–H (∼3600 cm^–1^) and O–D (∼2600 cm^–1^) vibrational bands, they recorded real-time spectra revealing how the intensity of each peak evolved with time and temperature.^373^ This enabled a mechanistic kinetic description: the isotope exchange followed a Grotthuss-type hopping mechanism along surface-adsorbed water networks, with an activation energy of only ∼ 9 kJ mol^–1^ and exchange rates at 175 °C more than 100 times faster than at 25 °C. Crucially, this spectroscopic approach provided spatially averaged, noninvasive monitoring of proton uptake dynamics at interfaces, complementing bulk tracer studies and extending H–D exchange into the low-temperature regime relevant for next-generation protonic ceramic devices. Similarly, Matsui and co-workers applied H/D isotopic exchange to a Li^+^/H^+^ ion-exchanged Li_3.13_H_0.37_Zn_0.25_GeO_4_ solid at 200–400 °C, confirming that the predominant charge carrier is a proton by observing ionic-transference changes upon isotopic substitution.^374^

While surface exchange and near-surface dynamics are essential, a complete picture of proton transport must also encompass bulk diffusion. In a recent preprint, Shin et al.^375^ applied tracer-exchange depth profiling via time-of-flight secondary-ion mass spectrometry (TOF-SIMS) to the triple-conducting perovskite BaCo_0.4_Fe_0.4_Zr_0.1_Y_0.1_O_3−δ_. After equilibrating samples in D_2_O at 400 °C, they quenched and sectioned the material, mapping the D concentration as a function of depth. Fitting these profiles yielded proton chemical-diffusion coefficients on the order of 10^–10^ cm^2^ s^–1^, even though protons comprised less than 0.2 mol % of the total charge carriers in BCFZY. This allowed unambiguous isolation of proton transport parameters from concurrent oxide-ion and electronic conductivity processes, revealing that—at typical reversible SOFC operating temperatures—the proton incorporation step is so facile as to be nonrate-limiting for the overall electrochemical reaction, which instead is controlled by slower oxygen-ion exchange kinetics.^375^

Beyond classical electrolyte materials, H–D exchange can probe protonation on mixed-conducting oxides and catalytically active perovskites. Zakharov et al.^376^ used HD-mass-spec monitoring under CH_4_/H_2_ environments over Ni–La_0.9_Sr_0.1_ScO_2.95_ cermet, deriving a heterogeneous H/D exchange rate of ∼ 2 × 10^11^ atoms cm^–2^ s^–1^ at 300 °C and attributing the rate-determining step to CH_x_–H adatom exchange on the catalyst layer.^377^ Similarly, catalytic-methane activation studies on La_1–x_Sr_x_ScO_3−δ_ surfaces employed H/D isotopic exchange and ^1^H NMR to identify parallel CH_x_ formation channels and correlate activity to surface oxygen-vacancy concentration.^376^

These landmark studies collectively map out the full thermodynamic and kinetic landscape of proton uptake via H–D exchange. At equilibrium, deuterium saturation levels provide a direct measure of how many protonic defects (OD^–^ or OH^–^) the lattice can accommodate; the difference between D and H uptake quantifies the zero-point energy shift and defines an isotopic equilibrium constant. Surface-exchange kinetics, derived from HD formation rates in gas-phase experiments or from the time-dependent growth and decay of O–H and O–D bands in vibrational spectroscopy, yield activation energies and surface-reaction rate constants that diagnose how readily external gases can supply or remove protons. Bulk transport rates, extracted via isotopic depth profiling (ERD or SIMS), yield chemical diffusion coefficients that quantify how quickly protons—or deuterons—penetrate the interior. By comparing H and D data, one can disentangle kinetic isotope effects from intrinsic material properties to translate deuterium-derived rates back into true protonic rates.

Despite its power, the H–D exchange carries several practical challenges. The small mass difference between H and D means that weight changes in gravimetry or mass spectrometric signals of HD can be subtle, demanding high-precision instrumentation. Techniques such as ERD or TOF-SIMS require ultrahigh-vacuum environments and specialized detectors capable of resolving isotopic ratios at trace levels. Vibrational spectroscopy demands careful baseline correction and spectral deconvolution to distinguish overlapping O–H and O–D peaks, particularly in multidoped or spectrally congested systems. Isotopic purity of the gas phase must be rigorously controlled to avoid inadvertent H contamination, and kinetic modeling must account for both thermodynamic and kinetic isotope effects to avoid misinterpretation of exchange rates. Moreover, transient isotope-switching experiments must be designed to decouple simultaneous hydration/dehydration, surface adsorption/desorption, and bulk diffusion processes—often requiring stepwise or pulse methods and complementary electrochemical or structural probes.

Nevertheless, when applied judiciously, H/D isotope exchange uniquely illuminates the fundamental processes of proton uptake and transport in proton-conducting electrolytes. From the anomalous room-temperature ERD measurements in SrCe_0.95_Yb_0.05_O_3−δ_ to the high-temperature gas-phase tracer studies in La_1–x_Sr_x_ScO_3−δ_, from *in situ* Raman monitoring of surface exchange on TiO_2_ to TOF-SIMS profiling in triple-conducting perovskites, isotopic tracing has revealed how many protons can be hosted, how fast they attach at the surface, how deeply they diffuse, and how these processes vary with composition, temperature, and microstructure. This body of work not only provides rigorous benchmarks for validating new proton-conducting materials but also offers mechanistic blueprints for optimizing surface and bulk properties—advancing the rational design of next-generation reversible solid oxide cell electrolytes through mechanistically interpretable, isotopically grounded insights.

#### 4.1.10 TOF-SIMS

TOF-SIMS has become an indispensable tool for probing proton uptake in proton-conducting electrolytes, providing the unique ability to detect and map hydrogen and its isotopes at the nanoscale. By bombarding the sample surface with a pulsed primary ion beam—commonly Bi^+^, Cs^+^, or O_2_^+^—TOF-SIMS sputters secondary ions from the outermost layers of the material, which are then separated by their mass-to-charge ratio in a TOF analyzer (Figure 29a,b). This configuration affords simultaneous detection of all ejected species with exceptional mass resolution and lateral sensitivity, making TOF-SIMS particularly well suited for identifying light elements such as H and D, even in complex oxide matrices.^378^

**Figure 29:**
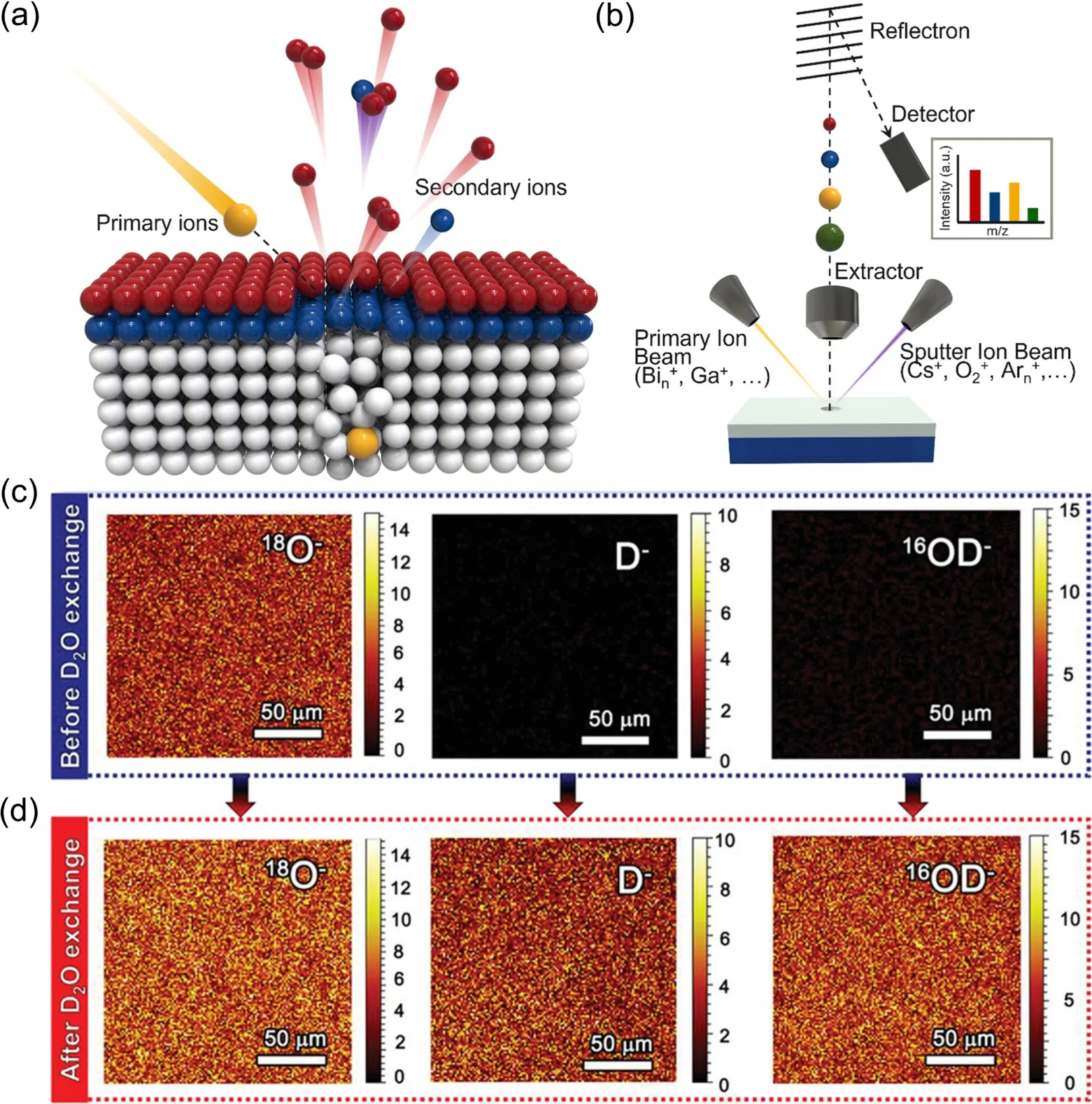
A schematic illustration of the TOF-SIMS principle and typical data sets of TOF-SIMS analysis. (a) Primary ions impact the solid sample surface to eject secondary ions. Secondary ions from only the top 1–2 atomic layer species can escape as SIMS is surface sensitive. (b) Time-of-flight principle. An electric field is added between the extractor and substrate to accelerate secondary ions for mass analysis; normally, only one polarity, either positive or negative ions, can be analyzed in a measurement. Reproduced with permission from ref 378. Copyright 2024 Elsevier. One exemplary case of secondary ion mapping images of ^18^O^–^, D^–^, and ^16^OD^–^ for the surface of the oxide pellet (c) before and (d) after D_2_O exchange. Reproduced from ref 382. Available under a CC-BY 4.0 license. Copyright 2021 Wiley Arim Seong et al.

Because protons in oxide lattices reside predominantly as hydroxyl groups (O–H), TOF-SIMS spectra typically feature characteristic secondary ions—H^+^, OH^–^, and molecular clusters like H_3_O^+^—that unambiguously signal proton incorporation. Yet, the ubiquitous presence of adventitious hydrogen in vacuum environments necessitates isotopic labeling (H/D exchange) to distinguish sample-derived signals from the background. By equilibrating a hydrated sample in D_2_O and then tracking D^+^ (*m*/*z* = 2) or OD^–^ (*m*/*z* = 18) in the TOF-SIMS spectra, one can isolate the deuterium signal and thus directly profile proton uptake with minimal interference.^378^

Earlier pioneering work demonstrated the technique’s broader applicability. Hancke et al.^379^ used *ex situ* TOF-SIMS to measure both hydrogen and ^18^O tracer diffusion in La_28–x_W_4+x_O_54+3x/2_ (La_5.6_W_0.4_O_12_) across 220–620 °C, obtaining activation energies of 63 kJ mol^–1^ for proton diffusion and confirming the ambipolar nature of proton and oxide-ion transport in this high-temperature conductor. More recently, Harvey et al.^380^ applied TOF-SIMS imaging to electroless-plated copper electrodes on BaZr_0.7_Ce_0.2_Y_0.1_O_3−δ_ membranes, correlating nanoscale Cu–Pd alloy formation and noncoking carbon phases with enhanced electrochemical stability during methane oxidative coupling. These studies underscore TOF-SIMS’s versatility in both pure electrolyte systems and composite electrode architectures.

A powerful extension of TOF-SIMS is depth-resolved isotope exchange diffusion profiling (IEDP). In this approach, a proton-conducting oxide is first exposed to H_2_O to saturate the surface and near-surface sites, then switched to D_2_O for a controlled exchange interval (Figure 29c,d). Following rapid quenching to “lock in” the isotope distribution, the sample is transferred under an inert atmosphere to the TOF-SIMS instrument. Continuous sputtering of the surface by the ion beam yields sequential depth layers while monitoring the D-containing secondary ions. Fitting the resulting deuterium concentration versus depth profile to solutions of Fick’s second law extracts both the bulk proton tracer diffusion coefficient (D_H_) and the surface proton exchange rate (k_H_). Zhang et al.^161^ applied IEDP-TOF-SIMS to a BaHf_0.8_Y_0.2_O_3−δ_-based proton conductor, extracting D_H_ and k_H_ at 450–600 °C. They observed that while D_H_ increased with temperature, k_H_ decreased due to the exothermic hydration reaction at the surface, directly linking anion substitution to optimized hydration kinetics and overall performance. In a complementary study, Li et al.^381^ investigated Nd_5.4_Mo_0.3_W_0.7_O_12−δ_, demonstrating through TOF-SIMS depth profiling that hydrogen permeation, water splitting, and hydration kinetics are tightly coupled: hydration rate constants k_H_ of 1.2 × 10^–6^ cm s^–1^ and proton tracer diffusivities D_H_ of 5 × 10^–7^ cm^2^ s^–1^ at 600 °C were reported, correlating subsurface deuterium profiles with measured hydrogen fluxes and revealing the material’s potential for integrated membrane-electrolyzer applications. In the context of RSOCs, TOF-SIMS has been instrumental in revealing how subtle compositional modifications influence proton uptake. For example, dynamic SIMS profiling combined with high-resolution TOF-SIMS imaging confirmed uniform deuterium penetration into the bulk and grain interiors of PBSCF oxides, directly visualizing three-dimensional distributions that matched impedance-derived σ_bulk_ trends.^382^

Several key advantages make TOF-SIMS uniquely powerful for proton uptake studies. First, its exceptional sensitivity—down to ∼ 10^7^ hydrogen atoms cm^–2^—and submicron lateral resolution allow the detection of trace proton concentrations and steep gradients near the electrolyte surface. Second, the mass resolution readily distinguishes ^2^H from ^1^H, enabling unambiguous isotope tracing even in complex, multielement matrices. Third, the combination of sputter depth profiling and high-resolution imaging delivers both quantitative concentration–depth profiles and spatial maps in two or three dimensions. Finally, the ability to collect all secondary ions in parallel permits correlative analyses—for instance, simultaneously monitoring O–H fragments alongside dopant and impurity ions to elucidate how microstructural features or aliovalent substitutions modulate proton uptake.^380,382,383^

Nevertheless, TOF-SIMS has important limitations that must be managed. The requirement for ultrahigh vacuum and the inherently destructive sputtering process can lead to partial loss or redistribution of labile protons during transfer or analysis, potentially underestimating absolute concentrations. Pervasive hydrogen background from chamber components and residual adsorbates imposes a practical detection limit of around 10^16^ atoms cm^–2^ in many instruments, complicating quantification without rigorous calibration against standards or by using H/D ratios. Matrix effects—variations in secondary-ion yield caused by local composition or structure—can skew depth profiles, while charging artifacts in poorly conductive ceramics may distort ion trajectories despite charge-compensation measures. Perhaps most significantly, TOF-SIMS yields limited direct information on the chemical bonding state of detected hydrogen: all lattice-bound protons typically appear as similar secondary ions, making it difficult to distinguish hydroxyl species from molecular water without complementary vibrational or electronic spectroscopy.^378,379,383^

By carefully designing isotope-exchange protocols, minimizing air exposure, and combining TOF-SIMS with *in situ* transfer systems or correlative techniques (e.g., IR spectroscopy, neutron scattering), researchers can mitigate these challenges. The resulting deuterium depth profiles, 3D isotope maps, and correlative multi-ion data sets provide unparalleled insight into proton self-diffusion, surface exchange kinetics, and the interplay between composition, microstructure, and hydration thermodynamics. These insights have guided the rational design of next-generation proton-conducting electrolytes—ranging from aliovalently doped perovskites to hybrid triple-conducting oxides—by revealing which modifications most effectively enhance bulk proton mobility and surface exchange, ultimately advancing the performance and durability of RSOCs and related protonic ceramic electrochemical devices.^379,382^

### 4.2 Proton Conductivity

#### 4.2.1 EIS

EIS has become the cornerstone for the quantitative characterization of proton transport in ceramic electrolytes, owing to its unique ability to disentangle distinct resistive and capacitive processes that occur on widely varying time scales. At its heart, EIS applies a small-amplitude sinusoidal perturbation—either in voltage or current—across the electrolyte-electrode assembly and records the resulting current or voltage response over a broad frequency range (typically 10^–2^ to 10^6^ Hz). By representing this response as a complex impedance shown in eq 22 and plotting -Z″ versus Z′ in a Nyquist diagram, each transport or interfacial process ideally manifests as a semicircular arc whose position and diameter correspond to the process’s characteristic time constant and resistance, respectively.^384^

22 $$Z\left(\omega \right)=Z^{\prime }\left(\omega \right)+{\mathrm{jZ}}^{\prime \prime }\left(\omega \right)$$

Where Z­(ω) is the complex impedance as a function of an angular frequency ω, Z′(ω) is the real part of the impedance representing resistive contributions, Z″(ω) is the imaginary part corresponding to capacitive or inductive elements, and j is the imaginary unit.

In polycrystalline proton conductors, the high-frequency arc is universally assigned to intragranular (bulk) proton conduction, while the intermediate-frequency feature reflects grain-boundary impedances arising from space-charge layers and chemical segregation at interfaces. At still lower frequencies, additional arcs or Warburg-type tails capture electrode polarization phenomena—charge-transfer reactions, adsorption/desorption kinetics, and mass transport in porous electrodes. Equivalent-circuit modeling, employing combinations of resistors (R), constant-phase elements (CPE), and Warburg impedances (W), is then fitted to the measured spectrum, yielding quantitative values for R_b_, R_gb_, and interfacial R_p_ after rigorous validation via Kramers–Kronig consistency checks.^385^

While classical R-CPE circuits suffice for a first-order decomposition, modern techniques such as DRT analysis go a step further by deconvoluting overlapping processes without assuming a fixed circuit topology. Applied to PCFCs, DRT has successfully separated anode and cathode R_p_, revealing, for instance, distinct peaks at ∼ 5 × 10^2^–1 × 10^4^ Hz (anode hydrogen oxidation) and ∼ 1–2 × 10^2^ Hz (cathode oxygen reduction).^320^ This spectral resolution proves essential when grain-boundary and electrode processes overlap, or when the CPE behavior of porous electrodes introduces nonideal capacitive responses.

The power of EIS to pinpoint the rate-limiting step in proton conduction is exemplified by recent studies across diverse material systems. In the BaSc_0.8_Mo_0.2_O_3–δ_ perovskite, which achieves high proton conductivity within the so-called “Norby gap” (0.01 S cm^–1^ at 320 °C), temperature-dependent EIS revealed a single, well-defined bulk-conducting arc down to 250 °C, indicating suppressed proton trapping near Mo-doped intrinsic vacancies and enabling three-dimensional proton migration in a cubic lattice.^170^ Similarly, in stoichiometric BaZr_0.4_Ce_0.4_Y_0.1_Yb_0.1_O_3−δ_, anode-supported PCFC exhibited an exceptionally low R_o_ of 0.060 Ω cm^2^ at 650 °C, directly extracted from the high-frequency intercept of the Nyquist plot, translating to conductivities as high as 0.1–0.2 S cm^–1^ under humidified H_2_ conditions.^80^ These results underscore how precise EIS modeling isolates the intrinsic protonic transport in the electrolyte from extrinsic contributions.

Beyond absolute conductivities, EIS also informs on microstructural design. Xing et al.^386^ demonstrated that engineering a semiconductor–ionic heterostructure by interfacing CeO_2−δ_ with BZY20 yields an interfacial proton conductivity of 0.23 S cm^–1^ at 520 °C—over an order of magnitude higher than bulk BZY20—evidenced by a pronounced increase in the midfrequency arc assigned to grain-boundary (interface) resistance upon sintering. Conversely, when grain-boundary resistances become comparable to bulk values, the Nyquist arcs merge, necessitating DRT or broadband (MHz) EIS to disentangle them. Ebert and colleagues exploited a custom EIS setup capable of 10 MHz measurements to push the deconvolution of bulk versus grain-boundary conductivities down to 350 °C in BZY20 and BZCY721, showing that bulk conduction dominates above a crossover temperature (∼200 °C for BZY20 and ∼ 150 °C for BZCY721), thus refocusing materials research on intrinsic lattice conductivity improvements.^387^

In all cases, the translation of EIS spectra to meaningful physical parameters hinges on meticulous experimental design. To confirm that the measured resistance indeed arises from protons—and not oxide ions or electronic leakage—researchers perform isotope exchange experiments, comparing spectra under H_2_O and D_2_O atmospheres; the expected kinetic isotope effect (a decrease in conductivity by $\sqrt{2}$ ) validates protonic conduction pathways. Simultaneously, comparing EIS in dry versus humidified atmospheres separates the contributions of oxygen-vacancy-driven oxide-ion conduction from water-mediated proton hopping.^386^

The advantages of EIS in this context are manifold: it is nondestructive, *operando* (capturing the electrolyte in actual device environments), and sensitive to minute interfacial resistances—grain-boundary contributions as small as 10^–5^ Ω cm^2^ produce distinct midfrequency arcs. Accessibility of commercial frequency response analyzers and fitting software (e.g., ZView, EC-Lab) has democratized EIS, making detailed impedance analysis routine even in multiphase composite electrolytes. Moreover, tutorial treatments such as those by Lazanas and Prodromidis provide clear guidelines on sinusoidal perturbation theory, complex-plane representations, Kramers–Kronig data validation, and equivalent-circuit simulation, empowering graduate students and senior researchers alike to deploy EIS across applications from corrosion to biosensing.^384,385^

Nevertheless, practitioners must remain vigilant of the technique’s limitations. Equivalent circuits are never unique; different topologies can yield statistically similar fits, so physical insights from microstructural analysis or complementary techniques (e.g., electron microscopy, secondary ion mass spectrometry) must guide circuit selection. Overlapping arcs—common when R_b_ and R_gb_ are of similar magnitude—or CPE behavior from porous electrodes and Warburg diffusion often requires broadband EIS or advanced fitting to Distributed Element models. In poorly sintered or composite ceramics, high grain-boundary impedances can obscure true bulk conductivities; in such cases, symmetric blocking electrodes (porous Pt) or focused-ion-beam-fabricated microelectrodes are employed to isolate intragranular transport. Finally, EIS quantifies only the net ionic resistance; distinguishing protonic from other ionic species demands isotope exchange or tracer diffusion studies.

Despite these challenges, EIS remains indispensable for research on electrolytes for RSOCs. It delivers nuanced insights into how defect chemistry, microstructure, and interface engineering converge to enable record-high proton conductivities and low activation energies. As demonstrated across low-temperature polymer composites, nanostructured ceramics, heterostructured interfaces, and bulk-optimized perovskites, EIS transforms raw impedance spectra into a detailed map of ionic landscapes—revealing where to focus synthesis, processing, and device architecture efforts to push the performance frontier of protonic ceramic electrochemical cells.

#### 4.2.2 Direct Current Four-Probe Method

In contrast to the richness of information obtainable by EIS, the DC four-probe method offers a straightforward, contact-resistance-free route to the σ_bulk_ of protonic ceramics, albeit with inherent limitations that tailor its role to a brief, confirmatory survey rather than exhaustive impedance analysis. In its simplest form, a constant direct current is driven through the outer pair of electrodes clamped to a polished ceramic bar or pellet, while the inner pair, connected to a high-impedance voltmeter, records the corresponding voltage drop across a known interprobe spacing. This spatial separation of current and voltage paths effectively eliminates contact resistance from the measurement, isolating the sample’s intrinsic resistivity, which—via Ohm’s law and knowledge of the specimen geometry—yields the total ionic conductivity (σ_tot_) at each temperature and atmospheric condition.

Typical sample preparation mirrors employed across the field: dense ceramic pellets or bars are produced via solid-state or combustion synthesis, followed by uniaxial pressing and high-temperature sintering to achieve >95% theoretical density. Surfaces are polished or lapped to promote intimate, reproducible contact, and four electrodes—commonly Pt paste or sputtered Au/Pt films—are patterned at equal spacing along the length of the sample bar. The outer current electrodes bear heavier Pt wires or meshes to carry DC currents (often μA to mA, chosen to minimize Joule self-heating), while the inner voltage taps connect to a nanovolt-sensitive, high-impedance meter. Once mounted in a tubular furnace with gas-flow control, the specimen is equilibrated at each temperature step (typically from 200 up to 800 °C) under precisely regulated pO_2_ and pH_2_O, and linear V–I sweeps are recorded in both polarities to cancel thermoelectric offsets. From the slope of V versus I, the bulk resistivity ρ follows directly via eq 23, where A is the cross-sectional area, and l is the voltage probe separation. The σ_tot_ obtained from eq 24 is then plotted in Arrhenius form (lnσ vs 1/T) to extract activation energies. Under humidified or isotopically substituted (H_2_O vs D_2_O) atmospheres, comparisons of σ_DC(H_2_O)_ to σ_DC(D_2_O)_ yield isotope-effect ratios that confirm the protonic nature of transport: for example, BaSc_0.8_Mo_0.2_O_3–δ_ exhibited σ_DC(H_2_O)_/σ_DC(D_2_O)_ ≈ 1.34, close to the theoretical $\sqrt{2}$ value expected for a single-ion proton regime.^170^

23 $$\rho =\frac{V}{I}\times \frac{A}{l}$$

24 $$\sigma_{\mathrm{tot}}=\frac{1}{\rho }$$

where ρ is the bulk resistivity of the sample, V and I are the measured voltage and applied current, respectively, A is the cross-sectional area of the sample perpendicular to current flow, and l is the distance between the inner voltage probes, σ_tot_ is the total ionic conductivity, obtained as the inverse of bulk resistivity ρ.

Although the DC four-probe technique cannot distinguish grain-boundary from bulk conduction without supplementary modeling, nor separate ionic from electronic pathways on its own, careful experimental design—such as varying pO_2_/pH_2_O or comparing humid vs dry atmospheres—permits deconvolution of protonic and oxide-ion contributions. In BaZr_0.1_Ce_0.7_Y_0.1_Yb_0.1_O_3−δ_, for instance, four-probe measurements under mixed pO_2_/pH_2_O conditions showed that proton transport accounted for ∼ 98% of the σ_tot_ at 600 °C, underscoring its near-pure protonic conduction.^69^

Beyond mixed perovskites, CaZr_1–x_Sc_x_O_3−δ_ (x = 0.03–0.20) measured by DC four-probe in H_2_ + H_2_O/N_2_ atmospheres similarly demonstrated unipolar proton conductivity below 700 °C, with peak σ_tot_ emerging at x from 0.05 to 0.10, directly correlating to optimal defect concentrations for hydration and proton mobility. Here, proton transport numbers approached unity until higher temperatures introduced a growing oxide-ion component.^388^

In CaSc and BaSc perovskites, the effect of humidity on AZr_0.95_Sc_0.05_O_3−δ_ (A = Ca, Sr, Ba) was probed by DC four-probe up to 900 °C: total, bulk, and grain-boundary conductivities all rose markedly with increasing pH_2_O at lower temperatures, highlighting the key role of hydronium-mediated transport across grain interfaces—an effect invisible to DC alone, yet readily apparent when combined with EIS.^389^

Several recent reports underscore the practicality of DC four-probe measurements under realistic RSOC conditions. Saito et al.^170^ stabilized BaSc_0.8_Mo_0.2_O_3–δ_ and employed four Pt-tip probes in O_2_/N_2_–5% H_2_ flows (pH_2_O = 0.02 atm), confirming high proton conductivity across 200–800 °C and verifying isotope effects close to theory. Kim et al.^69^ adopted the DC four-probe method to differentiate the partial conductivities of protons and oxide ions for BZCYYb1711 under an air atmosphere with 3% water vapor within the temperature range of 600–750 °C. The results revealed a proton conductivity of 1.3 × 10^–2^ S cm^–1^ at 600 °C, and a distinct onset of mixed conduction was observed at higher temperatures. More recently, Han et al.^390^ combined DC four-probe with EXAFS-guided dopant selection in La_2_(Nb_1–x_Y_x_)_2_O_7−δ_ pyrochlores: after identifying La-adjacent oxygen sites as the most hydration-active, they introduced Ca^2+^, Sr^2+^ and Ba^2+^ at those sites, and observed conductivities up to 1.16 × 10^–3^ S cm^–1^ at 700 °C—nearly four times that of the undoped oxides—highlighting how site-specific doping can dramatically boost proton transport. Similarly, Gorelov et al.^391^ performed direct-current four-probe measurements on CaZr_1–x_Sc_x_O_3−δ_ bars (x = 0.03–0.20) between 600 and 900 °C in humid hydrogen–nitrogen mixtures, revealing pure proton conduction below 700 °C, a conductivity maximum at x ≈ 0.05 (σ_tot_ ≈ 2 × 10^–4^ S cm^–1^ at 600 °C), and an increasing oxygen-ion contribution (16–21% at 900 °C), thereby highlighting the critical influence of Sc content on proton transport efficiency.

Despite its simplicity and speed—measurements can be completed in quasi-real time during temperature or atmosphere ramps—the DC four-probe method has two principal drawbacks compared to EIS. First, DC currents, especially under high humidity and long dwell times, can induce electrode polarization via ion accumulation at contacts, necessitating reduced currents and brief measurement windows to avoid concentration gradients. Second, the DC four-probe yields only net σ_tot_; without complementary impedance or spectroscopic data, it cannot resolve grain-boundary vs bulk, nor ionic vs electronic, components without invoking defect-equilibrium models or isotopic substitution. As a result, the DC four-probe is best reserved for initial conductivity surveys, cross-checks of EIS-derived values, or isotope-effect confirmation under process-relevant gas compositions.

In practice, the DC four-probe method remains invaluable when rapid, contact-resistance-free σ_bulk_ values are needed—such as during material screening, optimizing sintering conditions, or validating protonic dominance via isotope studies—before committing to more time-consuming impedance fits. When implemented with rigorous control of current magnitude, contact quality, and atmosphere composition, it yields high-confidence benchmarks for σ_tot_ and activation energies that guide deeper EIS and microstructural analyses in the development of next-generation proton-conducting electrolytes.

#### 4.2.3 Hebb–Wagner Direct Current Polarization Method

In mixed protonic–electronic conductors, the Hebb–Wagner DC polarization technique remains the benchmark for decoupling and quantifying partial ionic and electronic transport under well-controlled electrochemical conditions. Originally introduced in the 1950s, this method exploits the fact that a DC bias applied between a pair of electrodes—one selectively blocking a given mobile species while the other maintains electrochemical reversibility—drives the system into a concentration-polarized steady state in which the flux of the blocked carrier is suppressed to zero. Under such conditions, the residual current arises solely from the unblocked species, allowing extraction of its conductivity directly from the steady-state current–voltage relationship.^392^

At its core, the Hebb–Wagner approach considers a dense polycrystalline pellet (or thin film) of the mixed conductor sandwiched between an ion-blocking electrode (IBE) and a reversible electrode (RE). For protonic conductors, an ideal IBE must conduct electrons readily while effectively preventing proton exchange; dense noble-metal films or proton-impermeable ceramics such as yttria-stabilized zirconia serve admirably in this role. Conversely, the RE must be hydrogen-permeable—typically a porous nickel or platinum contact in a humidified hydrogen atmosphere—so that the proton chemical potential at that interface remains fixed by the gas phase. When a small galvanostatic current is imposed, protons accumulate on the IBE side until their forward flux vanishes; beyond this “polarization time,” the cell voltage reaches a plateau reflecting pure electronic conduction. Reversing the electrode roles yields the complementary measurement of protonic transport.^393^

Mathematically, the steady-state I–V relation follows the generalized Hebb–Wagner equation, which links the measured voltage V to the partial conductivities σ_H^+^_ and σ_e_, the sample thickness L, and the applied current density j as shown in eq 25, where t_e_ and t_H^+^_ are the electronic and proton transference numbers (summing to unity).^394^ In the IBE/RE configuration above, one transference number becomes effectively zero at the IBE interface, so that the plateau voltage V_ss_ satisfies eq 26 from which σ_unblocked_ (either σ_e_ or σ_H^+^_) is obtained directly.^395^

25 $$V=\frac{\mathrm{jL}}{\sigma_{e}}t_{e}+\frac{\mathrm{jL}}{\sigma_{H^{+}}}t_{H^{+}}$$

26 $$V_{\mathrm{ss}}=\frac{\mathrm{jL}}{\sigma_{\mathrm{unblocked}}}$$

where V is the measured steady-state voltage across the sample, j is the applied current density, and L is the sample thickness, σ_e_ and σ_H^+^_ are the partial conductivities of electrons and protons, respectively, t_e_ and t_H^+^_ are the corresponding transference numbers, V_ss_ is the steady-state plateau voltage after polarization, and σ_unblocked_ is the conductivity of the charge carrier that remains mobile across both electrodes (either electrons or protons, depending on which species is blocked at the IBE).

In practice, modern implementations refine the original two-electrode geometry by adopting a four-probe design that separates current injection and voltage sensing. Such cells feature two outer electrodes for galvanostatic control and two inner, high-impedance reference probes to record the “true” sample voltage drop, thereby eliminating artifacts from contact resistance and electrode overpotentials. Miruszewski and colleagues demonstrated this approach on a mixed-conducting BaCe_0.6_Zr_0.2_Y_0.2_O_3_ pellet, using BaZr_0.7_Ce_0.2_Y_0.1_O_3_ as both proton- and oxide-ion-blocking contacts; by monitoring the voltage evolution until a steady plateau was reached, they extracted the oxygen-ion conductivity at 800 °C with excellent agreement to literature values (∼2.3 × 10^–3^ S cm^–1^).^322^ More recently, the same four-wire Hebb–Wagner cell was adapted to mixed protonic–electronic oxides, with La_0.98_Ca_0.02_NbO_4_ serving as a double-blocking electrode that prohibits both electrons and oxide ions, thus isolating the protonic current; in this configuration, the partial proton conductivity of BaGd_0.3_La_0.7_Co_2_O_6−δ_ was measured to be 4.4 × 10^–5^ S cm^–1^ at 600 °C, corresponding to a transference number of order 10^–8^.^396,397^

Beyond perovskite-based systems, the Hebb–Wagner DC polarization method has proved invaluable in probing proton transport in diverse materials. In Ruddlesden–Popper-type La_2_NiO_4+δ_ phases, where interstitial oxide ions rather than vacancies dominate defect chemistry, Chen et al.^396^ employed a La_0.99_Ca_0.01_NbO_4_ blocking electrode to quantify high-temperature protonic conductivity in Cu- and Co-substituted analogues; the results confirmed that proton incorporation and mobility correlate strongly with interstitial oxide concentration, attaining the highest values in Co-doped samples at 400–575 °C. Similarly, in fluorite-related La_27_(W_1–x_Nb_x_)_5_O_55.5‑δ_ lanthanum tungstate, systematic variation of Nb-induced oxygen vacancies tuned the t_H^+^_ above 0.98 below 800 °C, while above that temperature mixed conduction with both n- and p-type electronic contributions emerged; here again, Hebb–Wagner polarization provided a direct, model-free measure of ionic versus electronic currents over a wide temperature and atmosphere window.^398^

The virtues of Hebb–Wagner DC polarization lie chiefly in its straightforward interpretation and sensitivity. By physically blocking one species, the method avoids the complexity of equivalent-circuit fitting often required in AC impedance spectroscopy and yields transference numbers and partial conductivities inherently. Even trace protonic currents (transference <10^–7^) can be detected in highly electronic matrices by careful choice of a hydrogen-impermeable IBE and a prolonged polarization period.^322,397^ Moreover, four-probe sensing diminishes uncertainty from contact resistance, and environmental control of pH_2_O and pO_2_ allows mapping of conductivity contributions as functions of chemical potentials.

Nevertheless, accurate application demands attention to several potential pitfalls. First, a true steady state must be reached—relaxation times scale with sample thickness, ionic diffusivities, surface exchange coefficients, and the magnitude of the applied current. In systems where surface exchange kinetics are sluggish, equilibration may require minutes to hours, and partial steady-state amplitudes can be masked by slow drift or electronic injection effects at high bias.^396,398^ Second, the classical Hebb–Wagner theory presumes a single dominant mobile ion; in materials with concurrent protonic, oxide-ionic, and electronic conduction, the I–V response reflects the convolution of multiple species unless double-blocking contacts are engineered to isolate each runner. Third, high DC bias can induce nonideal phenomena—such as space-charge layer formation, electrode overpotential nonlinearity, or even chemical decomposition under extreme atmospheres—compromising the plateau interpretation unless current densities remain low and voltages modest.^392,397^ Finally, in proton conductors, stable hydration throughout the cell’s bulk is critical; water loss at elevated temperatures or under dry atmospheres can lead to underestimation of σ_H^+^_ unless pH_2_O is carefully controlled.

Despite these challenges, Hebb–Wagner DC polarization continues to be the definitive route for decoupling partial conductivities in mixed protonic–electronic ceramics. Its direct linkage of steady-state I–V plateaus to single-species transport obviates fitting ambiguities, while four-probe and double-blocking electrodes extend their reach to triple-conducting and complex oxide families. By combining rigorous steady-state criteria with tailored electrode architectures and environmental control, researchers can extract σ_H^+^_, σ_O^2–^_, and σ_e_ with high fidelity, thereby illuminating the mechanistic underpinnings of proton mobility, defect interactions, and chemical-potential-driven transport in next-generation protonic ceramic electrolytes.^392^

#### 4.2.4 EMF Method

The EMF method for characterizing proton-conducting electrolytes in RSOCs leverages a concentration-cell configuration to impose a defined chemical-potential gradient across a dense ceramic membrane and, under open-circuit conditions, to read out the resulting equilibrium voltage. In its simplest form, a highly dense protonic-ceramic disc (typically >95% of theoretical density) is sandwiched between two gas chambers, each fitted with reversible hydrogen electrodes—most commonly porous platinum films sintered onto the electrolyte surfaces. By exposing the two sides to different H_2_/H_2_O atmospheres, a steady-state chemical-potential difference for protons is established; protons migrate through the bulk electrolyte to balance this gradient, while the interfacial hydrogen-water redox reactions at the electrodes maintain local equilibrium. Because no net current flows under open-circuit conditions, the measured cell voltage, E_meas_ corresponds directly to the protonic component of the chemical-potential drop and, in a pure proton conductor, equals the Nernst potential for the H_2_/H_2_O couple as shown in eq 27, where R and F are the universal gas and Faraday constants, respectively, and the superscripts “high” and “low” denote the partial pressures on the two sides.^399^ In the ideal case of a purely protonic electrolyte, the measured open-circuit voltage reproduces this Nernst potential exactly (i.e., transport number t_H^+^_ = 1). However, real ceramics often exhibit some degree of coconduction by oxide ions or electronic carriers; in such mixed conductors, the observed EMF is suppressed relative to the ideal Nernst voltage, and the proton transference number is readily obtained by the simple ratio as shown in eq 28.^400^ Because only the fraction t_H^+^_ of the ionic current is carried by protons. In practice, researchers calculate E_Nernst_ from the known gas ratios, measure E_meas_ under open-circuit conditions, and thus extract t_H^+^_, which, when combined with total conductivity data from impedance or DC measurements, yields the absolute proton conductivity σ_H^+^_ from eq 29.

27 $$E_{\mathrm{Nernst}}=\frac{\mathrm{RT}}{2F}\ln\frac{p_{H_{2}}^{\mathrm{high}}p_{H_{2}O}^{\mathrm{low}}}{p_{H_{2}}^{\mathrm{low}}p_{H_{2}O}^{\mathrm{high}}}$$

28 $$t_{H^{+}}=\frac{E_{\mathrm{meas}}}{E_{\mathrm{Nemst}}}$$

29 $$\sigma_{H^{+}}=\sigma_{\mathrm{tot}}t_{H^{+}}$$

More generally, in a mixed-conducting electrolyte where multiple mobile species such as protons, oxide ions, and electrons contribute to charge transport, the OCV arises from the collective response of all electrochemical potential gradients. Under steady-state, zero-current conditions, the total EMF can be expressed as a sum over all charge carriers, as shown in eq 30.^400^

30 $$E_{\mathrm{meas}}=\underset{i}{\sum }\frac{t_{i}\mathrm{RT}}{z_{i}F}\ln\left(\frac{c_{i}^{\mathrm{high}}}{c_{i}^{\mathrm{low}}}\right)$$

where t_i_ is the transference number of species i, z_i_ is the charge number, c_i_^high^ and c_i_^low^ are effective concentrations on the two sides of the membrane, R is the universal gas constant, T is the absolute temperature, and F is the Faraday constant.

Experimentally, the EMF setup requires meticulous attention to cell assembly and gas-handling.^321,401,402^ The electrolyte pellet—often 1–2 mm thick and sintered to >95% density—is coated on both faces with porous Pt (or, less commonly, Au) electrodes. Silver is avoided because it can electromigrate under hydrogen atmospheres and degrade electrode performance. The cell is mounted within a tube furnace equipped with dual gas inlets and robust seals that isolate the two chambers. Initially, both sides are purged with a reference mixture (e.g., 45% H_2_/50% Ar/5% H_2_O) to verify a zero baseline EMF; subsequently, one side is switched to a dry H_2_ stream or a higher H_2_O content gas to impose the desired H_2_/H_2_O gradient. Precise gas ratios are maintained using calibrated mass-flow controllers and steam generators, ensuring accurate partial pressures. After equilibrium—typically tens of minutes at each temperature—the open-circuit voltage is measured with a high-impedance voltmeter or potentiostat. Background offsets, such as minor oxygen activity differences or reference junction potentials, are quantified under symmetric gas conditions and subtracted from the gradient-induced voltage to isolate the true EMF.

Data analysis begins by computing the ideal Nernst potential, E_Nernst_, from the imposed gas partial pressures at each temperature. The measured voltage, E_meas_, is then divided by E_Nernst_ to yield the proton transference number, t_H^+^_. In mixed-conducting systems, impedance spectroscopy is often collected in parallel; by fitting Nyquist plots to appropriate equivalent circuits, one obtains high-frequency intercepts that correct for any electrode or polarization resistances, thereby refining the true equilibrium EMF. When multiple charged species coexist, additional experiments—such as reversing the high/low H_2_O side or substituting O_2_ atmospheres—help decouple ion-specific contributions. Averaging across several gas gradients and temperatures enhances reliability, under the assumption that protonic defects equilibrate rapidly and that Pt electrodes remain reversible with negligible overpotential.^403^

Putilov, Tsidilkovski, and Demin develop a rigorous analytical framework for protonic ceramic fuel cells that captures how nonuniform distributions of protons and hole carriers form under both open-circuit and operating conditions.^404^ By deriving closed-form expressions for the local and effective EMF, as well as for proton and hole conductivities and transport numbers as functions of cell voltage and gas compositions, they demonstrate that defect redistribution can cause the “true” OCV to deviate significantly from the ideal Nernst value when measured under load. Their model further predicts how humidity and temperature modulate both the EMF and FE, offering guidance for optimizing PCFC performance under a realistic operating regime.

In recent years, the electromotive-force concentration-cell technique has emerged as the benchmark for quantifying proton transport in advanced ceramic electrolytes. Morishita et al.^405^ applied EMF measurements to Ni, Y codoped BaZrO_3−δ_ ceramics between 500 and 900 °C, demonstrating excellent agreement with Nernstian predictions and confirming nearly ideal protonic conduction with negligible electronic leakage; transference-number analyses further yielded t_H^+^_ ≈ 1 across this entire temperature window, thereby validating proton-dominant transport even in the presence of trace oxide-ion conduction. Conversely, Li-bridged apatite-type Ca_5_(PO_4_)_3_OH exhibited sub-Nernstian EMFs corresponding to t_H^+^_ ≈ 0.70–0.87 between 430 and 550 °C, and revealed that silver electrodes systematically underestimate t_H^+^_ compared with platinum—underscoring the critical role of catalytically active, reversible interfaces for accurate EMF determinations.^401^ This combined EMF and total-conductivity approach has since been extended to layered perovskites and other complex oxides to disentangle mixed ionic–electronic conduction phenomena and directly extract proton conductivities once t_H^+^_ is established.

Building on this framework, Jiang, Norby, and Han performed EMF analyses on Y-doped SrZrO_3_ (SZY10), employing both hydrogen and water-vapor concentration cells at 500–700 °C to decouple proton and oxide-ion transport in bulk and grain-boundary regions. Under reducing conditions (constant pH_2_O), the measured voltages closely followed Nernst predictions (t_H^+^_ ≈ 0.93–0.97), confirming protonic dominance; under oxidizing water-vapor cells (constant pH_2_), the measured EMFs were considerably lower than the theoretical Nernst voltage for hydrogen. This gives an oxide ion transference number (t_O^2–^_) of approximately 0.02–0.16, with t_O^2–^_ increasing with temperature. By integrating these EMF-derived transference numbers with impedance-spectroscopy deconvolution, they extracted partial σ_H^+^_ and σ_O^2–^_ values for both grains and grain boundaries, revealing that grain boundaries exhibit reduced ionic transport due to enhanced electronic hole leakage in oxidizing atmospheres.^406^

More recently, Han, Liu, Bjørheim, and Uda applied hydrogen- and water-vapor EMF measurements to BaZr_0.8‑x_Ce_x_Y_0.2_O_3−δ_ (x = 0–0.8) electrolytes between 500 and 700 °C.^66^ Hydrogen-gradient cells (constant pH_2_O) yielded overall ion transference numbers t_ion_ ≈ 0.97–0.99, while water-vapor cells (constant pH_2_) revealed t_O^2–^_ rising from ∼ 0.02 at 500 °C to ∼ 0.33 at 700 °C. Coupling these EMF-derived transport numbers with total conductivities determined by EIS enabled the deconvolution of σ_H^+^_ and σ_O^2–^_, demonstrating that increased Ce content enhances oxide-ion mobility at elevated temperatures while preserving near-unity proton transference at 500–600 °C.

Nevertheless, practical limitations must be acknowledged. The EMF method assumes negligible electronic or oxide-ionic leakage; if these carriers become significant, the open-circuit voltage is depressed, and transference-number extraction grows error-prone. Only materials with high t_H^+^_ produce measurable voltages; for low-t_H^+^_ ceramics, the EMF may approach zero or fall within noise levels. Electrode polarization—resistance to H_2_/H_2_O exchange—also reduces the apparent EMF; while highly catalytic Pt mitigates this, residual overpotentials must be corrected via impedance. Moreover, stringent cell sealing and gas isolation are vital: any leakage or cross-contamination of chambers invalidates the chemical-potential gradient. Equilibration times of tens of minutes are required for true steady-state, and background potentials from oxygen activity differences or reference junctions must be zeroed out. Finally, the EMF yields transference numbers, not absolute conductivities; it must be complemented by total-conductivity measurements (EIS or DC) to calculate proton conductivities from eq 29.

In sum, the EMF concentration-cell method remains a classical, yet indispensable, tool for probing proton conduction in RSOCs electrolytes. By imposing controlled H_2_/H_2_O chemical potentials and reading the resulting open-circuit voltage, one directly accesses the proton transference number; in combination with total conductivity, this delivers the proton conductivity itself. Modern studies—from doped BaZrO_3−δ_ to complex apatite—demonstrate how ideal Nernstian behavior confirms high t_H^+^_ transport, while subideal voltages reveal mixed conduction and guide materials optimization. Accurate implementation—dense, well-electroded specimens, precise gas control, and careful impedance-based corrections—ensures quantitative insights into protonic behavior, enabling rational development of novel proton-conducting electrolytes for advanced RSOCs.

#### 4.2.5 Hydrogen–Deuterium Isotope Method

The H/D isotope exchange method stands out as a definitive probe of protonic transport in ceramic electrolytes by exploiting the nearly 2-fold mass difference between H^+^ and D^+^. In a typical experiment, a dense electrolyte pellet—often conditioned to >95% theoretical density—is first equilibrated under an H_2_O- or H_2_-containing atmosphere at a fixed temperature, and its ionic conductivity is measured using either DC four-probe or AC impedance techniques. The gas atmosphere is then switched to an isobaric D_2_O/D_2_ environment at identical temperature and total pressure, and the conductivity is remeasured. Because the diffusion coefficient D classically scales inversely with the square root of the ion mass, as shown in eq 31, the corresponding conductivity ratio for a purely protonic transport mechanism is expected to follow eq 32.^407^

31 $$D\propto m^{-1/2}$$

32 $$\frac{\sigma_{H^{+}}}{\sigma_{D^{+}}}\approx \sqrt{\frac{m_{D^{+}}}{m_{H^{+}}}}\approx 1.41.$$

where σ_H^+^_ and σ_D^+^_ are the measured conductivities in H_2_O and D_2_O environments, respectively, m_H^+^_ and m_D^+^_ are the effective masses of H^+^ and D^+^, and the factor $\sqrt{\frac{m_{D^{+}}}{m_{H^{+}}}}$ reflects the kinetic isotope effect for a hopping-type diffusion process. Observation of a conductivity ratio close to 1.41 within experimental uncertainty confirms that proton transport proceeds via a Grotthuss-type hopping mechanism. Conversely, the absence or attenuation of this ratio indicates competing transport pathways involving oxide ions or electronic defects.^408^

At its core, the H/D isotope effect arises from alteration of the zero-point energy and vibrational frequencies of the O–H bond when H is replaced by D. In a hydrated oxide lattice, protons attach to framework oxygens to form O–H groups (or OH^–^ defects), and hop between adjacent oxygens through a sequence of bond-breaking and bond-forming events. The heavier deuteron shifts the O–D vibrational band to the lower frequency, raising the classical activation barrier for hopping and slowing diffusion. In the simplest approximation—neglecting quantum-tunneling corrections—the isotope dependence of mobility μ and conductivity σ follows eq 31 and eq 32 under identical carrier concentrations. More refined treatments incorporate quantum tunneling of protons, which slightly modifies the ratio but preserves the large kinetic isotope signature. Observations of σ_H^+^_/σ_D^+^_ near $\sqrt{2}$ across a range of proton-conducting oxides—such as cubic BaSc_0.8_Mo_0.2_O_3–δ_, hexagonal Ba_5_Er_2_Al_2_SnO_13_, and surface-proton-conducting CeO_2_—attest to the universality and quantitative power of the H/D test.^170,409^

Experimentally, careful control of hydration levels is critical: D_2_O and H_2_O vapor pressures differ, and a thermodynamic isotope effect can lead to slight discrepancies in proton concentration between H- and D-exposures. To mitigate this, researchers match the partial pressures of H_2_O and D_2_O (accounting for their distinct vapor pressures) or cycle the atmosphere until steady-state conductivity is achieved. In practice, DC four-probe measurements are favored for their simplicity and avoidance of capacitive artifacts, while AC impedance spectroscopy can separate bulk, grain-boundary, and surface-proton contributions via equivalent-circuit analysis. For instance, Matsuzaki et al.^409^ alternated O_2_-saturated humid N_2_ and D_2_O/N_2_ atmospheres at 300 °C on Ba_5_Er_2_Al_2_SnO_13_, observing a reversible conductivity jump with σ_H^+^_/σ_D^+^_ ≃ 1.5 (close to $\sqrt{2}$ ) every cycle. Likewise, Matsuda^408^ and co-workers applied in situ impedance on porous CeO_2_ under dry H_2_ and D_2_ atmospheres between 423 and 573 K, finding conductivity ratios σ_H^+^_/σ_D^+^_ ≈ 1.27–1.69 bracketing the classical value and confirming that proton–electron pairs generated by dissociative H_2_ adsorption dominated surface conduction.

Beyond pure proton conductors, the H/D method has proven invaluable in mixed-conducting oxides. Saito et al.^170^ demonstrated that Mo-doped BaScO_2.5_ (BaSc_0.8_Mo_0.2_O_3–δ_) exhibits high proton conductivity within the so-called “Norby gap,” with σ_H^+^_/σ_D^+^_ ≈ 1.34 at 300 °C under humidified air, closely matching $\sqrt{m_{H}/m_{D}}$ , and a proton transference number t_H^+^_ ≃ 1 from complementary EMF data. In high-entropy perovskite BaSn_0.16_­Zr_0.24_­Ce_0.35_­Y_0.1_­Yb_0.1_­Dy_0.05_O_3−δ_, Guo and He verified proton conduction via a pronounced isotope effect and hydration-driven conductivity enhancement, establishing a proton-conductor isotope effect in a multiprincipal-element framework.^410^ In triple-conducting oxides such as PrNi_0.7_Co_0.3_O_3−δ_, temperature-programmed desorption coupled with H/D isotope exchange revealed that increasing Ni occupancy stabilizes proton defects in the lattice and enhances proton mobility, a strategy that underpins improved catalytic rates for water-splitting and oxygen-reduction reactions in protonic ceramic electrochemical cells.^411^ Meanwhile, TOF-SIMS isotope-exchange depth profiling has quantified proton tracer diffusion coefficients up to 10^–6^ cm^2^ s^–1^ at 550 °C in layered triple-conducting oxides, 2 orders of magnitude above their oxygen tracer diffusion, further highlighting the H/D approach’s versatility for both bulk and surface proton kinetic studies.^170^

In the realm of new hexagonal perovskite-related oxides, Ba_5_R_2_Al_2_SnO_13_ (R = Gd, Dy, Ho, Y, Er, Tm, Yb) exhibits high proton conductivity via full hydration in its octahedral layers without extrinsic doping. Matsuda et al.^408^ measured σ_H^+^_/σ_D^+^_ ≈ 1.2–1.6 at 300 °C for Ba_5_Er_2_Al_2_SnO_13_, affirming that proton mobility is confined to two-dimensional planes between intrinsically oxygen-deficient layers and that isotope exchange remains a robust probe of such anisotropic diffusion. These case studies underscore that the H/D method can be applied across a spectrum of structures—from cubic perovskites and hexagonal variants to high-entropy and triple-conducting oxides—providing a unified framework for pinpointing and quantifying proton transport in complex materials.

The primary advantage of the H/D isotope approach lies in its directness: by isolating the mass-dependent mobility term, it separates carrier identity from concentration effects, requiring only a conductivity cell and accessible isotopes (D_2_O or D_2_ gas). Unlike methods that infer proton conduction indirectly—such as EMF cells or transference-number analysis—the H/D test yields a quantitative mobility ratio that can be compared directly to theoretical expectations. It can be implemented *in situ* under operating conditions relevant to device applications (e.g., 300–700 °C, humidified or reductive atmospheres) without specialized labeling or tracer isotopes beyond deuterium and water.^382^

Nonetheless, several caveats accompany the technique. First, ensuring equal proton concentrations under H_2_O and D_2_O is nontrivial: slight differences in adsorption enthalpies or vapor pressures can introduce a thermodynamic isotope effect that skews n_H_ vs n_D_. This is often mitigated by matching vapor pressures or equilibrating until reproducible steady-state conductivities emerge. Second, the conductivity change (30–50%) is modest compared to σ’s absolute value, demanding high-precision instrumentation and careful background subtraction of electrode and grain-boundary resistances. Third, mixed conduction can dilute the observed ratio: even a few percent electron or oxide-ion leakage will reduce σ_H^+^_/σ_D^+^_ below the ideal $\sqrt{2}$ , necessitating complementary EMF or tracer-exchange measurements to validate proton transference. Fourth, if proton transport proceeds via a vehicular mechanism—where OH^–^ complexes migrate intact rather than Grotthuss-style hopping—the simple $\sqrt{m_{D^{+}}/m_{H^{+}}}$ rule no longer applies, and interpretation requires detailed modeling of complex diffusion pathways. Finally, deuterium exchange at low temperatures can be kinetically slow, so equilibration times must be tailored to each material’s adsorption kinetics to achieve complete isotope substitution.

In summary, the H/D isotope exchange method remains the gold standard for unequivocally identifying and quantifying proton conduction in oxide electrolytes. By measuring kinetic isotope effects on conductivity, researchers can directly confirm H^+^ mobility, distinguish Grotthuss vs vehicular mechanisms, and uncover subtle mixed-carrier contributions. From early demonstrations in lanthanum orthoantimonates (LaSbO_4_) to recent applications in high-entropy perovskites, hexagonal close-packed materials, and triple-conducting oxides, the H/D approach has proven both versatile and quantifiable.^412,413^ When combined with complementary techniques—such as EMF cells, tracer-diffusion profiling, and theoretical modeling—it provides a comprehensive lens into protonic transport phenomena critical for the design of next-generation RSOCs.

### 4.3 Multimodal Characterization Strategies

A quantitative mechanistic understanding of proton-conducting oxides almost always emerges from the deliberate combination of multiple complementary methods, because no single probe spans the full hierarchy of length, time, and information scales that govern protonic transport. The relevant length scales extend from the Å-scale O–H bond and its first coordination shell, through the nanometre-scale cooperative networks formed by hydrated dopant clusters, to the micrometre-scale grain interiors and grain boundaries that ultimately set the macroscopic conductivity of a sintered ceramic. The relevant time scales are equally broad, ranging from the ∼ 10^–13^ s period of an O–H stretching vibration, through the ∼ 10^–12^ to 10^–9^ s residence times of an individual proton between successive Grotthuss-type hops, to the ∼ 10^–3^ to 10^2^ s relaxation of macroscopic chemical-potential gradients probed by impedance, EMF, and galvanostatic experiments. Each experimental technique resolves only a narrow window within this multiscale landscape, and the limitations of any given method—surface specificity, model dependence, restricted temperature window, or insensitivity to particular charge carriers—are typically the strengths of another. Multimodal characterization strategies exploit precisely this complementarity, and a small number of recurring technique combinations have proven especially powerful in the proton-conductor literature.

The combination of TGA with neutron-based techniques offers the most established route from bulk hydration thermodynamics to atomistic-scale structural and dynamical resolution. TGA performed under controlled pO_2_ and pH_2_O quantifies the equilibrium constants K_O_ and K_W_, the enthalpies and entropies of hydration and oxidation extracted from Van’t Hoff analysis (typically ΔH_hyd_ ≈ −80 to −170 kJ mol–1 for acceptor-doped cerates and zirconates), and the absolute proton concentration as a continuous function of temperature and atmosphere.^313^ However, TGA is intrinsically blind to where the absorbed protons reside in the lattice, how they distribute over symmetry-distinct oxygen sites, and how rapidly they hop between adjacent positions. Neutron-based probes complete this picture: NPD, supplemented by Rietveld refinement or maximum-entropy density mapping and routinely carried out on D-substituted samples to enhance the scattering contrast against the host lattice, locates protons relative to the host framework and refines their site occupancies;^317^ PGAA exploits the high (n, γ) cross-section of ^1^H to return an absolute, model-independent count of incorporated hydrogen that calibrates the diffraction-derived occupancies;^350^ INS supplies the vibrational fingerprints that distinguish hydroxyl environments through their O–H stretching, bending, and librational modes;^180,318^ and QENS measures the quasi-elastic broadening of the elastic line, from which proton jump distances, residence times, and activation energies for diffusion are extracted directly without recourse to a transport model.^63^ The BaZr_1–x_In_x_O_3−δ_ series exemplifies this synergy: TGA-derived hydration stoichiometry agreed quantitatively with NPD-refined proton occupancies and was independently validated within ∼ 5% by PGAA, producing a self-consistent thermodynamic–structural description that none of the three techniques could have delivered alone.^350^ Analogous TGA + QENS analyses on BaZrO_3−δ_- and BaCeO_3−δ_-based perovskites have explicitly reconstructed the macroscopic activation barrier for long-range proton transport from the elementary hopping statistics, providing one of the cleanest experimental tests of the underlying Grotthuss mechanism.

A second class of integration links local-scale dynamics to macroscopic transport. ^1^H MAS NMR identifies distinct hydroxyl environments through chemical-shift fingerprints, typically resolving structural OH groups in the 5–9 ppm range from physisorbed water near 5 ppm, while spin–lattice relaxation time, spin–spin relaxation time, and motional line-narrowing analyses extract local jump rates and dwell times of individual protons over a broad temperature range; EXSY further distinguishes proton populations that exchange on the millisecond-to-second time scale.^319^ These molecular-scale observables become mechanistically powerful only when correlated with the bulk and grain-boundary conductivities resolved by EIS:^320^ a discrepancy between the activation energy obtained from NMR—which reports intrinsic hopping in the absence of long-range obstacles—and that from EIS—which reports the effective transport energy that includes all blocking processes—directly reveals the contribution of dopant trapping, defect association, or grain-boundary space-charge resistance. This strategy provided the first unambiguous demonstration of dopant-cation proton trapping in Y-doped BaZrO_3−δ_, in which ^1^H–^89^Y correlation NMR localized protons preferentially in the first coordination shell of Y^3+^ acceptors, with the resulting trap depth (∼0.2 eV) accounting quantitatively for the gap between the intrinsic hopping barrier resolved by NMR and the macroscopic activation energy measured by EIS (∼0.4–0.5 eV).^63,366^ The same logic now underpins multinuclear NMR investigations of Sc-, In-, and Ga-substituted BaZrO_3−δ_, in which the magnitude of the trap depth correlates with the dopant ionic radius and chemical hardness—a predictive design rule that no single technique could have supplied.

Vibrational spectroscopy combined with H/D isotope exchange constitutes a third natural pairing. Replacement of H by D shifts every O–H mode toward a lower frequency by approximately the harmonic isotope ratio ν_O–H_/ν_O–D_ ≈ 1.36–1.41, providing an unambiguous spectroscopic signature of protonic incorporation that cannot be confused with surface-adsorbed water, residual organic contamination, or instrument background.^315,316^ When several hydroxyl environments coexist, each population exhibits a characteristic shift magnitude, intensity, and exchange rate; tracking these jointly during isotopic substitution therefore deconvolutes the proton speciation and quantifies the relative population of each site. INS combined with operando IR on Ba_2_In_2_O_5−δ_ hydrates resolved two crystallographically distinct proton sublattices, including a minority population trapped in ultrashort hydrogen bonds that produced an anomalously red-shifted O–H stretch near 255 meV.^180^ In the double-perovskite Ba­(Zn, M)­O_2.9−δ_(OH)_2δ_ (M = Ta, W), in situ FTIR mathematically deconvoluted the broad 2500–4000 cm^–1^ envelope into two symmetric and two asymmetric hydroxide motifs, with their relative thermal stabilities mirroring the macroscopic two-order-of-magnitude conductivity contrast observed between Ta- and W-rich variants.^341^ Extending the same logic to operando geometries, in situ Raman has been used to follow H/D substitution in real time at temperatures as low as 150 °C, capturing not only the static end-state proton populations but also the kinetics of exchange itself—an information channel that is invisible to ex situ measurements and increasingly relevant to assessing electrolyte response under realistic P-RSOC transient operation.

On a more macroscopic scale, isotope-tracer (D and ^18^O) depth profiling by TOF-SIMS combined with EMF or impedance has emerged as the definitive way to disentangle proton mobility from proton concentration in mixed protonic–electronic conductors. SIMS yields the D_H_ and k_H_ directly from concentration-depth profiles after isotopic equilibration with D_2_O- or ^18^O2-labeled atmospheres, with submicrometre depth resolution that enables grain-boundary and bulk contributions to be resolved independently.^379,409^ EMF and impedance, in contrast, return the D_chem_ together with partial conductivities, t_H^+^_,t_O^2–^_ and t_e_.^320,321^ The thermodynamic enhancement factor corresponds to the ratio of the D_chem_ to the D_H_, which is entirely governed by the concentration dependence of the proton chemical potential. Combining these two measurements enables the determination of absolute proton concentration without the need for independent hydration tests, and further clarifies whether conductivity variations across the doping series arise from changes in carrier density or carrier mobility.^414^ In acceptor-doped barium zirconates and cerates, this analysis has shown that mobility variations contribute substantially to the observed conductivity trends—an insight masked by impedance measurements alone and one that has informed contemporary dopant-selection strategies for high-conductivity electrolytes.

Underpinning all of the experimental pairings above, the integration of experimental observables with DFT and AIMD has become an essential component of modern proton-conductor characterization. DFT at the generalized gradient approximation or hybrid functional level predicts the most stable proton sites, OH orientations, and crystallographic-site-resolved O–H vibrational frequencies, while NEB calculations and AIMD trajectories capture proton motion over picosecond windows, providing direct access to migration barriers, the relative weights of Grotthuss reorientation versus translational hopping, and the temperature dependence of jump-distance distributions. Comparison of these predictions with INS spectra, NMR chemical shifts, neutron-derived site occupancies, QENS-derived jump frequencies, and conductivity activation energies provides the cross-validation needed to elevate a structural model from plausible to rigorously demonstrated. The classical AIMD studies of BaZrO_3−δ_ and BaCeO_3−δ_ first established that proton transport in cubic perovskites proceeds dominantly via a Grotthuss mechanism with rotational reorientation as the rate-limiting elementary step, a conclusion subsequently reinforced by QENS- and NMR-derived activation energies and now extended to more complex hexagonal and disordered hosts.^415^ Recent studies on BaSc_0.8_Mo_0.2_O_3–δ_ ^170^ and hexagonal Ba_7_Nb_3.9_Mo_1.1_O_20.05_ ^354^ illustrate the mature form of this integration: AIMD-predicted vacancy topologies and migration barriers were cross-validated against neutron-determined site occupancies, isotope-resolved conductivities, and pO_2_-independent transport behavior, producing a self-consistent atomistic picture of high-mobility proton transport in vacancy-disordered hosts that no individual measurement could have established. Collectively, these multimodal strategies transform a catalogue of individual measurements into a self-consistent mechanistic narrative that translates atomistic chemistry into device-relevant performance metrics; their complementary capabilities, characteristic temperature windows, and limitations are summarized in Table 4, which serves as a practical reference for matching characterization tools to the specific mechanistic questions at hand in the design of next-generation proton-conducting electrolytes for P-RSOCs.

## 5 Fabrication Methods

The microstructure of proton-conducting electrolytes critically influences the overall electrochemical performance of proton-conducting RSOCs, significantly determined by electrolyte density, thickness, and interfacial quality. The electrochemical contribution of the electrolyte typically manifests as the R_o_ of the system, expressed theoretically as eq 33, where the theoretical ohmic resistance (R_o,th_) depends on electrolyte thickness (L), electrolyte conductivity (σ), and the cross-sectional area (A).^416^ Thus, beyond selecting electrolyte materials with inherently high proton conductivity, minimizing electrolyte thickness is critical to reducing the overall R_o_, thereby enhancing RSOC performance. Furthermore, electrolyte microstructure is equally crucial, as proton transport in bulk materials is significantly higher than grain-boundary transport.^417^ Consequently, an ideal electrolyte layer from a fabrication perspective would be extremely thin and essentially free from grain boundaries.

33 $$R_{o,\mathrm{th}}=\frac{L}{\sigma A}$$

To produce single cells featuring thin electrolyte layers, researchers typically employ three structures: (1) fuel electrode-supported SOCs, utilizing a thick fuel electrode (500 μm–1 mm) as structural support beneath a thin electrolyte layer; (2) metal–supported cells, leveraging robust, cost-effective, and easily manufacturable porous metallic supports coated with thin layers of fuel electrode and electrolyte; and (3) anodic aluminum oxide (AAO)-supported cells, employing nanoporous anodized Al_2_O_3_ supports to anchor thin electrolyte and fuel electrode layers.

Given the importance of microstructural control in optimizing electrolyte performance, numerous fabrication methodologies have been explored to achieve thin, dense electrolytes characterized by tailored grain structures. Effective fabrication methods must precisely manage grain growth, minimize R_gb_ by encouraging columnar grain structures, ensure nanocrystalline microstructure uniformity, and provide substantial interfacial areas, thereby maximizing the effective surface interaction.

In this chapter, we systematically review the major fabrication techniques for proton-conducting electrolyte layers, together with the specialized sintering approaches developed to optimize their microstructure. The discussion covers the underlying principles, typical preparation processes, associated advantages and limitations, and representative examples drawn from recent studies. To facilitate direct comparison, key electrochemical performance metrics (e.g., OCV, R_o_, etc.) of state-of-the-art single cells incorporating BaCe­(Zr)­O_3−δ_-based electrolytes fabricated using different techniques are summarized in Table 5. To further assist readers in selecting an appropriate fabrication route, Table 6 provides a concise comparison of the typical electrolyte thickness, throughput and cost, major advantages, principal limitations, and most suitable application scenarios (e.g., fundamental pellet studies, laboratory-scale thin-film cells, or scalable manufacturing) associated with each fabrication and sintering method discussed in this chapter.

**Table 5:** Performance of Recent P-RSOCs Fabricated Using Different Processing Methods^a^

Fabrication method	Electrolyte material and Sintering temperature	Electrolyte thickness	Ohm resistance @ temperature	Fuel electrode and Air electrode material	PPD @ temperature	OCV @ temperature	Atmosphere
Dry pressing	BZY20 ^418^ and 1500 °C	25 μm	1.70 Ω cm^2^ @ 600 °C	NiO–BZY20 and LSCF–BZY20	70 mW cm^–2^ @ 600 °C	0.98 V @ 600 °C	wet H_2_–dry air
	BZCY442 ^419^ and 1400 °C	20 μm	0.90 Ω cm^2^ @ 600 °C	NiO–BZCY and BSCFT–BZCY	194 mW cm^–2^ @ 600 °C	1.08 V @ 600 °C	wet H_2_–dry air
	BZCYYb1711–0.95 ^493^ and 1450 °C	12 μm	0.38 Ω cm^2^ @ 600 °C	NiO–BZCYYb1711and BCFZY	562 mW cm^–2^ @ 600 °C	1.06 V @ 600 °C	H_2_–dry air
Tape casting	BZY20 ^291^ and 1450 °C	5 μm	0.10 Ω cm^2^ @ 600 °C	NiO–BZCY/NiO–BZY20 and PNC	1240 mW cm^–2^ @ 600 °C	1.06 V @ 600 °C	wet H_2_–dry air
	BSCYb172 ^421^ and 1400 °C	7 μm	0.07 Ω cm^2^ @ 600 °C	NiO–BSCYb172 and PBCHf10	1490 mW cm^–2^ @ 600 °C	1.08 V @ 600 °C	H_2_–dry air
	BZCYYb1711 ^494^ and 1450 °C	7 μm	0.15 Ω cm^2^ @ 600 °C	NiO–BZCYYb1711and PBCFN	723 mW cm^–2^ @ 600 °C	1.05 V @ 600 °C	wet H_2_–wet air
Screen printing	BCS^428^ and 1400 °C	16 μm	0.70 Ω cm^2^ @ 600 °C	NiO–BCS and LSF	125 mW cm^–2^ @ 600 °C	1.04 V @ 600 °C	wet H_2_–dry air
	BZCY172 ^429^ and 1400 °C	25 μm	0.88 Ω cm^2^ @ 600 °C	Ni–BZCY and SBCO	154 mW cm^–2^ @ 600 °C	1.04 V @ 600 °C	H_2_–dry air
Spin coating	BZCYYb2611 ^434^ and 1350 °C	8 μm	0.20 Ω cm^2^ @ 600 °C	NiO–BZCYYb2611 and PBSCF	648 mW cm^–2^ @ 600 °C	1.10 V @ 600 °C	wet H_2_–dry air
Dip coating	BZCY721 ^433^ and 1500 °C	12 μm	0.87 Ω cm^2^ @ 450 °C	NiO–BZCY172 and NiO–BZCY172	-	-	wet H_2_–wet Helium
	BZCYZ (Tubular)^436^ and 1450 °C	25 μm	1.58 Ω cm^2^ @ 600 °C	NiO–BZCYZ and LSCF–BZCYZ	152 mW cm^–2^ @ 600 °C	1.01 V @ 600 °C	wet H_2_–dry air
	BZCY172 ^495^ and 1400 °C	35 μm	0.25 Ω cm^2^ @ 600 °C	NiO–BZCY172 and BCFZY–BZCY361	510 mW cm^–2^ @ 600 °C	1.07 V@ 600 °C	H_2_–dry air
	BZY20 ^496^ and 1450 °C	20 μm	0.50 Ω cm^2^ @ 600 °C	NiO–BZY20 and LSM	-	0.98 V @ 600 °C	wet H_2_–air/10% water
Spray coating	BZCYYb4411 ^160^ and 1450 °C	4 μm	0.11 Ω cm^2^ @ 600 °C	NiO–BZCYYb4411 and BSC–PBSCF	1640 mW cm^–2^ @ 600 °C	1.08 V @ 600 °C	H_2_–dry air
	BZCYYb1711 ^440^ and 1300 °C	4 μm	0.30 Ω cm^2^ @ 600 °C	NiO–BZCYYb1711 and BSCF–BZCYYb1711	418 mW cm^–2^ @ 600 °C	1.10 V @ 600 °C	H_2_–dry air
	BZCY172 ^442^ and 1400 °C	20 μm	0.62 Ω cm^2^ @ 650 °C	NiO–BZCY172 and LNCO–BZCY172	362 mW cm^–2^ @ 600 °C	1.03 V@ 650 °C	H_2_–dry air
Magnetron sputtering	BCY20 ^448^ and 700 °C	1 μm	0.25 Ω cm^2^ @ 600 °C	Pd and LSCF	1050 mW cm^–2^ @ 600 °C	1.08 V @ 600 °C	H_2_–wet air
	BZY20 ^453^ and 1000 °C	4 μm	7.02 Ω cm^2^ @ 525 °C	NiO–BZY20 and BSCF–BZY20	-	0.78 V @ 525 °C	H_2_–dry air
PLD	BCZY503515 ^457^ and 700 °C	2 μm	0.11 cm^2^ @ 600 °C	NiO–BCZY50.35.15 and LSC	635 mW cm^–2^ @ 600 °C	1.00 V@ 600 °C	wet H_2_–dry air
	BZY15 ^458^ and 1200 °C	3 μm	0.10 Ω cm^2^ @ 600 °C	NiO–BZY15 and LSC	740 mW cm^–2^ @ 600 °C	1.00 V @ 600 °C	wet H_2_–dry air
	BZY20 ^497^ and 600 °C	1 μm	0.50 Ω cm^2^ @ 450 °C	Pt and Pt (AAO support)	44 mW cm^–2^ @ 450 °C	1.04 V @ 450 °C	wet H_2_–dry air
	BZY20 ^498^ and 1000 °C	4 μm	1.90 Ω cm^2^ @ 600 °C	NiO–BZY20 and LSCF/BCYb10	110 mW cm^–2^ @ 600 °C	0.99 V @ 600 °C	H_2_–dry air
	BZY20 ^499^ and 600 °C	1 μm	0.22 cm^2^ @ 450 °C	Pt and Pt (AAO support)	21 mW cm^–2^ @ 450 °C	1.10 V @ 450 °C	H_2_–dry air
ALD	BZY20 ^471^ and 800 °C	100 nm	-	Pt and Pt	136 mW cm^–2^ @ 400 °C	1.09 V @ 400 °C	H_2_–dry air

a Because processing parameters, operating conditions, and cell configurations are not fully standardized across studies, the reported electrochemical metrics, including R_o_, OCV, and PPD, should be interpreted and compared with caution. Note: BZCY442 = BaZr_0.4_Ce_0.4_Y_0.2_O_3−δ_; BZCYYb1711–0.95 = Ba­(Zr_0.1_Ce_0.7_Y_0.1_Yb_0.1_)_0.95_O_3−δ_; BCY20 = BaCe_0.8_Y_0.2_O_3−δ_; BZY20 = BaZr_0.8_Y_0.2_O_3−δ_; BZY15 = BaZr_0.85_Y_0.15_O_3−δ_; BZCY172 = BaZr_0.1_Ce_0.7_Y_0.2_O_3−δ_; BZCY721 = BaZr_0.7_Ce_0.2_Y_0.1_O_3−δ_; BZCYZ = BaZr_0.4_Ce_0.4_Y_0.15_Zn_0.05_O_3−δ_; BZCYYb4411 = BaZr_0.4_Ce_0.4_Y_0.1_Yb_0.1_O_3−δ_; BZCYYb1711 = BaZr_0.1_Ce_0.7_Y_0.1_Yb_0.1_O_3−δ_; BZCYYb2611 = BaZr_0.2_Ce_0.6_Y_0.1_Yb_0.1_O_3−δ_; BCZY503515 = BaCe_0.5_Zr_0.35_Y_0.15_O_3−δ_; BCYb10 = BaCe_0.9_Yb_0.1_O_3−δ_; BCS = BaCe_0.8_Sm_0.2_O_3−δ_; BSCYb172 = BaSn_0.1_Ce_0.7_Yb_0.2_O_3−δ_; BZCY361 = Ba Zr_0.3_Ce_0.6_Y_0.1_O_3−δ_; BCFZY = BaCo_0.4_Fe_0.4_Zr_0.1_Y_0.1_O_3−δ_; PNC = PrNi_0.7_Co_0.3_O_3−δ_; BSC + PBSCF = Ba_0.62_Sr_0.38_CoO_3−δ_–Pr_1.44_Ba_0.11_Sr_0.45_Co_1.32_Fe_0.68_O_6−δ_; BSCFT = Ba_0.5_Sr_0.5_(Co_0.8_Fe_0.2_)_0.9_Ti_0.1_O_3−δ_; LSCF = La_0.6_Sr_0.4_Co_0.2_Fe_0.8_O_3−δ_; BSCF = Ba_0.5_Sr_0.5_Co_0.8_Fe_0.2_O_3−δ_; LSC = La_0.6_Sr_0.4_CoO_3−δ_; LNCO = LiNi_0.8_Co_0.2_O_2_; SBCO = SmBaCo_2_O_5+x_; LSM = (La_0.8_Sr_0.2_)_0.98_MnO_3−δ_; LSF = La_0.7_Sr_0.3_FeO_3−δ_; PBSCF = PrBa_0.5_Sr_0.5_Co_1.5_Fe_0.5_O_5+δ_; PBCFN = PrBaCo_1.6_Fe_0.2_Nb_0.2_O_5+δ_.

**Table 6:** Comparative Overview of Fabrication and Sintering Methods for Proton-Conducting Electrolyte Layers in P-RSOCs^a^

Method (Section)	Typical electrolyte thickness	Throughput/cost	Major advantages	Key limitations	Most suitable applications
Fabrication methods					
Dry pressing (5.1)	10–800 μm	Low/very low	★Simple, rapid, and reproducible	★Limited geometries	★Fundamental pellet studies
			★Mechanically robust pellets	★Imperfect electrode/electrolyte interfaces	★Early stage materials screening
			★Useful for bulk-property measurements	★Not suitable for thin-film cells	
Tape casting (5.2)	5–20 μm	High/low to moderate	★Scalable to large areas	★Requires shrinkage matching	★Laboratory-scale single cells
			★Compatible with multilayer integration	★Defect risk increases with cell area	★Large-area planar cells
			★Uses mature SOFC infrastructure	★Requires careful binder burnout	★Cell stacks
					★Commercial-scale cells
Screen printing (5.3.1)	10–30 μm	High/low	★Simple and scalable	★Sensitive to slurry rheology	★Laboratory-scale single cells
			★Flexible patterning geometry	★Pinholes and microcracks are possible	★Large-area planar cells
			★Compatible with standard cell fabrication	★Requires shrinkage-mismatch control	
Spin coating (5.3.2)	1–10 μm	Low/low (high material waste)	★Excellent thickness control	★Restricted to planar substrates	★Laboratory-scale single cells
			★Uniform planar coatings	★Multiple cycles required	★Planar thin-film research
			★Lower densification temperature	★Poor fit for large-area cells	
Dip coating (5.3.3)	10–30 μm	Moderate/low	★Versatile for planar and tubular geometries	★Limited coating uniformity	★Laboratory-scale single cells
			★Minimal equipment required	★Multiple cycles required	★Planar and tubular cells
			★Good adhesion with low pinhole density	★Drying-induced cracking possible	★Precommercial microtubular P-RSOCs
Spray coating (5.3.4)	3–20 μm	High/moderate	★Noncontact deposition	★Requires slurry optimization	★Laboratory-scale single cells
			★Suitable for fragile or curved substrates	★Drying-induced cracking possible	★Planar and tubular cells
			★Fine control of sub-10 μm films	★Higher system complexity	★Commercial-scale cells
3D printing (5.7)	10–20 μm	Moderate/moderate to high	★Single-step multilayer fabrication	★Specialized equipment required	★Laboratory-scale single cells
			★High geometric flexibility	Requires sintering-shrinkage matching	★Early pilot-scale tubular cells
			★Digital pattern control		
Drop coating (5.7)	5–20 μm	High/very low	★Extremely simple process	★Uneven films possible	★Laboratory-scale single cells
			★Minimal equipment required	★Multiple cycles required	★Early stage materials screening
				★Moderate film quality	
Inkjet printing (5.7)	1–3 μm	Moderate/moderate	★Digitally controlled patterning	★Susceptible to coffee-ring effects	★Laboratory-scale single cells
			★Minimal material waste	★Drying-induced cracking possible	★Thin and patterned electrolyte studies
			★High resolution	★Requires complex ink formulation	
Magnetron sputtering (5.4.1)	0.1–4 μm	Low/high	★Dense columnar microstructure	★Possible Ba deficiency	★Laboratory-scale thin-film cells
			★Low-temperature densification	★Limited deposition rate	★Thin protective layers
			★Clean, smooth interfaces	★Limited deposition area	★Thin-film fundamental studies
PLD (5.4.2)	0.1–4 μm	Very low/very high	★Accurate stoichiometry	★Low deposition rate	★Fundamental thin-film studies
			★Epitaxial, grain-boundary-free films	★Limited area	★Mechanistic studies
			★High system complexity	★High conductivity in model films	
				★Poor industrial scalability	
CVD (5.5.1)	0.1–5 μm	Low to moderate/high	★Conformal coating on complex geometries	★Limited precursor library	★Exploratory laboratory studies
			★Difficult cell integration	★Potential scalable processing route	★Relatively high deposition rate
ALD (5.5.2)	25–300 nm	Very low/high	★Precise stoichiometry control	★Limited Ba precursor options	★Laboratory-scale thin-film cells
			★Pinhole-free conformal coatings	★Very low deposition rate	★Micro-RSOCs
			★Lowest sintering requirement	★Underdeveloped for P-RSOCs	★Protective layers and interfaces
Sintering methods					
Conventional sintering (5.6.1)	-	High/low	•Well-established standard process	•Long dwell time	Default densification route across fabrication methods and scales
			• Compatible with most geometries	•High temperature	
				•Ba volatilization	
				•Cation interdiffusion	
RLRS (5.6.2)	-	Very high/moderate to high	•Densification within seconds	•Requires precise parameter control	Laboratory-to-pilot-scale processing
			•Reduced chemical loss during sintering	•Limited working area per scan	
			•Local thermal processing	•Scale-up remains difficult	
MWS (5.6.3)	-	High/moderate	•Volumetric heating	•Nonuniform energy absorption	Laboratory-to-pilot-scale processing
			•Rapid heating rates (up to ∼ 500 °C min^–1^)	•Localized overheating possible	
			•Requires careful equipment design	•Reduced sintering time and energy use	
Spark plasma sintering (5.6.4)	-	Low per cycle/high	•Densification at 400–500 °C	•Limited by graphite-die size	•Fundamental studies
			•Minute-scale densification	•Potential carbon contamination	• Laboratory-scale dense samples
			•Nanostructured microstructure	•Not yet scalable	
Flash sintering (5.6.4)	-	High locally/moderate	•Densification at much lower temperatures	•Risk of thermal runaway	Laboratory-scale studies
			• Energy-efficient processing	•Structural inhomogeneity	
				•Difficult equipment setup	
				•Scale-up still under development	
Hot-press sintering (5.6.4)	-	Moderate/moderate to high	•Lower-temperature densification	•Die-geometry constraints	Laboratory-to-pilot-scale processing
			• Possible die contamination	•Precise microstructure control	
				•Nonuniform cell density	

a Note: “section” refers to the corresponding subsection in Chapter 5. The reported thickness, throughput, and cost values are order-of-magnitude estimates derived from the references cited in the relevant subsections; actual values may vary depending on material composition, support architecture, and processing parameters. Sintering methods represent postshaping densification processes and, therefore, are not associated with a specific electrolyte thickness.

### 5.1 Dry Pressing

Dry pressing, also known as uniaxial or isostatic pressing, is one of the most established methods for ceramic shaping and is particularly well suited for fabricating dense, flat electrolyte pellets used in fundamental studies. As illustrated in Figure 30a, this method involves compacting synthesized electrolyte powders into a desired shape using a rigid die under applied mechanical pressure, typically in the range of 100–1000 MPa. The compacted “green body” is then subjected to high-temperature sintering to achieve densification and mechanical integrity. Before pressing, the powder must be phase-pure and well-prepared, with synthesis methods such as SSR, sol–gel processing, hydrothermal synthesis, or coprecipitation commonly employed.^417^ These precursors are typically blended with a binder (e.g., poly­(vinyl alcohol), PVA), a sintering aid (∼1 wt % NiO in the case of state-of-the-art BZCYYb1711 systems), and occasionally lubricants, to ensure homogeneity and facilitate compaction.

**Figure 30:**
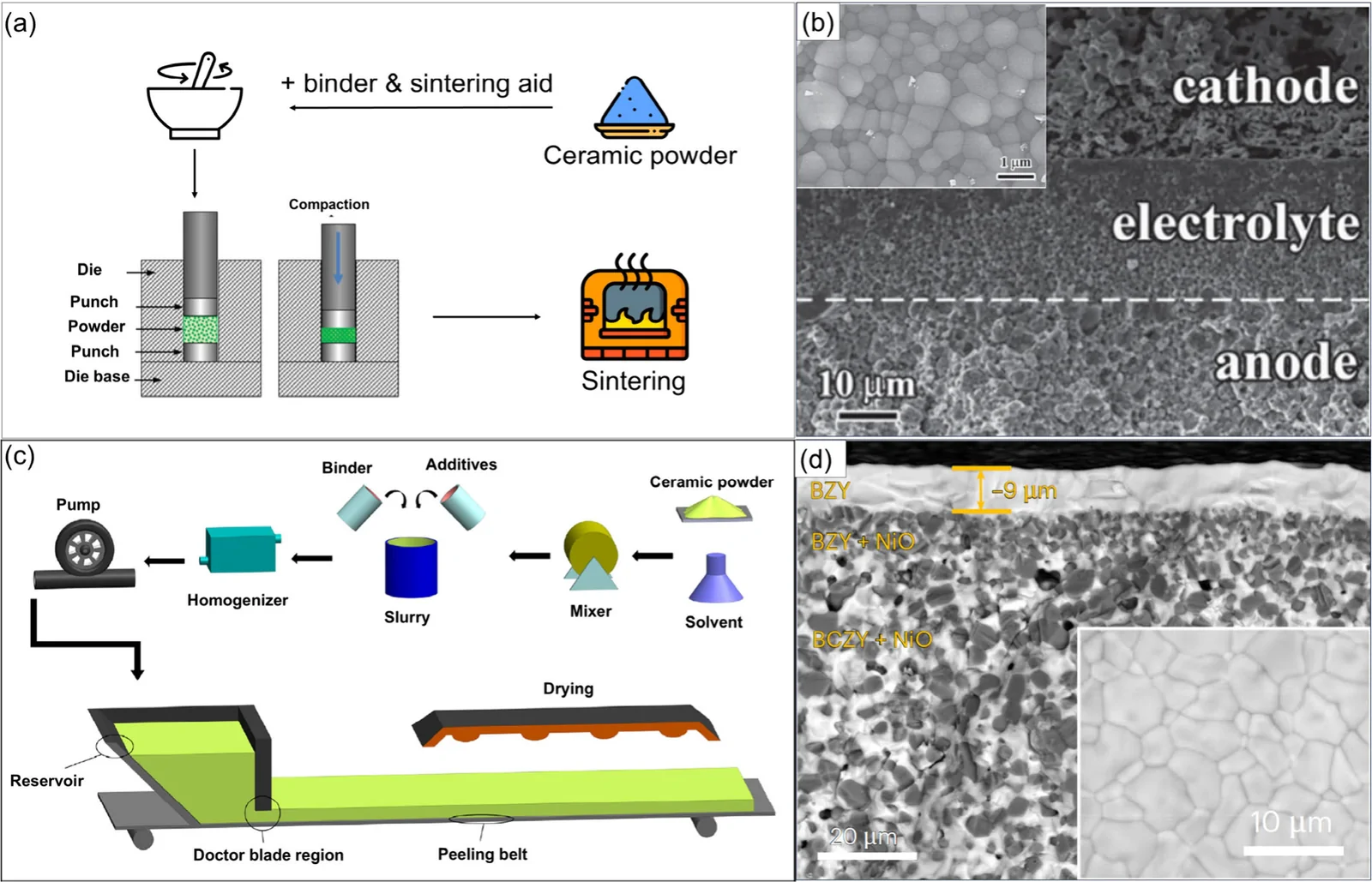
(a) Die pressing method for the preparation of pellet samples. Reproduced with permission from ref 423. Copyright 2022 Springer Nature. (b) Cross-section of the microstructure of the as-prepared copressing cell with BZY20 as the electrolyte material. Reproduced with permission from ref 418. Copyright 2011 Wiley. (c) Typical tape casting process. Reproduced with permission from ref 422. Copyright 2016 Elsevier. (d) Cross-section of the microstructure of the as-prepared tape-casting cell with BZY20 as the electrolyte material. Reproduced with permission from ref 291. Copyright 2025 Springer Nature. All inset: the microstructure of the corresponding electrolyte surface.

The sintering process is typically conducted in air or controlled atmospheres at 1300–1500 °C to achieve dense ceramics with >95% theoretical density. This approach reliably produces electrolyte specimens with thicknesses in the range of 100–800 μm, which are mechanically robust and ideal for characterizing intrinsic transport properties such as σ_bulk_ and σ_gb_. Owing to its simplicity, low cost, and compatibility with routine lab equipment, dry pressing remains a preferred method for rapid screening of new electrolyte compositions or for preparing bulk samples for fundamental electrochemical analysis.

In many studies, dry-pressed pellets serve as the foundational platform for evaluating the protonic conductivity, chemical stability, and electrochemical durability of novel electrolyte materials. For instance, in our previous work, we systematically assessed the transport properties of a series of Ba-based perovskite electrolytes with varying dopant chemistries under different atmospheres and temperatures.^65,135,264^ The most promising candidate was subsequently used in single-cell fabrication with more advanced techniques such as tape casting to achieve thinner electrolyte layers and reduced R_o_.

In addition to single-material pellets, copressing of multilayer structures is also feasible. In this approach, multiple layers (e.g., functional electrode and electrolyte) are physically stacked and compacted simultaneously, followed by cosintering. While this allows for the construction of more complex structures, challenges remain. The physical interface formed between layers in copressed assemblies, even with an added binder, often lacks the intimate bonding achieved in cosintered tape-cast layers, leading to inferior mechanical or electrochemical integration. Furthermore, due to gas-tightness constraints, the minimum achievable electrolyte thickness in copressed bilayers remains relatively high—typically ≥ 20 μm.

Nevertheless, several studies have demonstrated the viability of copressing in producing dense proton-conducting electrolytes with reasonable thickness and performance. For example, as shown in Figure 30b, Sun et al.^418^ fabricated fuel-electrode supported cells using BZY20 as the electrolyte, achieving a thickness of ∼ 25 μm and a R_o_ of 0.95 Ω cm^2^ at 700 °C. Similarly, Bi et al.^419^ obtained a 20 μm-thick BaZr_0.4_Ce_0.4_Y_0.2_O_3−δ_ electrolyte layer with a reduced R_o_ of 0.63 Ω cm^2^ at the same temperature. These results indicate that while copressing may not yield the thinnest or most refined films, it remains a viable route for small-scale cell prototyping and property evaluation.

Dry pressing remains a foundational method in the processing of proton-conducting ceramics, offering a straightforward, rapid, and reproducible route to fabricate dense electrolyte pellets for fundamental characterization. It is particularly advantageous in early stage material screening due to its low equipment requirements and ability to generate mechanically stable specimens with consistent microstructure. However, limitations inherent to dry pressing restrict its utility for advanced cell architectures. The method is generally constrained to simple geometries and relatively thick layers (typically >100 μm), making it unsuitable for low-resistance thin-film electrolytes required in high-performance cells.

### 5.2 Tape Casting

Tape-casting is a well-established wet ceramic processing technique in which a slurry is cast into thin, flexible green sheets. It is particularly attractive for the fabrication of proton-conducting electrolytes due to its scalability, compatibility with multilayer structures, and ability to produce uniform and large-area films with a controlled thickness. As depicted in Figure 30c, a typical tape-casting process involves preparing a stable slurry composed of electrolyte powder, polymeric binders, plasticizers, dispersants, and solvents (either aqueous or organic), which is then spread onto a carrier film using a doctor blade. Upon drying, a flexible green tape is formed. The film thickness can be tailored by tuning key parameters, such as solids loading, blade gap, and slurry rheology. For dense and uniform electrolyte tapes, solids loading exceeding 25 wt % and sufficiently low viscosity are considered essential to achieve desirable microstructural homogeneity and sintered density.^420^

In proton-conducting RSOCs architectures, multilayer integration of electrolyte, electrode, and support structures is often required. One effective strategy to achieve this integration is sequential cotape casting, where layers are cast in sequence onto a single carrier or release film before drying and cofiring. Leonard et al.^420^ demonstrated a trilayer structure consisting of a BaZr_0.44_Ce_0.36_Y_0.2_O_3−δ_ electrolyte (cast with a doctor blade gap of 65 μm, yielding a sintered thickness of ∼ 16.5 μm), followed by casting a NiO–BaZr_0.5_Ce_0.4_Y_0.1_O_3−δ_ support and a NiO–BaZr_0.44_Ce_0.36_Y_0.2_O_3−δ_ functional layer. Liu’s group^421^ adopted a similar cocasting process to fabricate single cells using BSCYb172 as the electrolyte, with NiO–BSCYb172 used in both functional and support layers. In this configuration, electrolyte thicknesses as low as 7 μm were achieved while maintaining excellent electrochemical performance, evidenced by an OCV of 1.08 V and a record-low R_o_ of 0.069 Ω cm^2^ at 600 °C under H_2_-air conditions.

An alternative multilayer assembly approach is lamination, in which each green tape is cast and dried separately before being stacked and pressed together under mild heat and pressure. Leonard et al.^420^ demonstrated successful lamination of a BaZr_0.44_Ce_0.36_Y_0.2_O_3−δ_ electrolyte tape with a NiO-based fuel electrode tape using a warm press at ∼ 80 °C, enabling cofiring into a mechanically robust cell. Once assembled, laminated green bodies undergo binder burnout and sintering in a single thermal cycle. Typical burnout schedules include slow ramp rates and hold between 300–600 °C to avoid thermal stress and delamination.^422^ Final sintering at 1350–1500 °C is required to achieve gastight electrolyte layers. Leonard et al.^420^ reported successful densification of a BaZr_0.44_Ce_0.36_Y_0.2_O_3−δ_-based half-cell by cosintering at 1350 °C for 5 h, whereas our group employed a 1450 °C, 5-h sintering cycle to produce fully dense BSCYb172 electrolytes.^421^

One of the critical considerations in cosintering is the compatibility of the shrinkage behavior among different layers. Mismatched shrinkage between the electrolyte and the electrode layers can lead to warping, delamination, or mechanical failure. To mitigate this issue, identical or closely matched electrolyte compositions are often employed across both layers (e.g., NiO–BZY20 for the fuel electrode and BZY20 in the electrolyte). Recent work by Tang et al.^291^ leveraged the constrained sintering effect of a mechanically rigid NiO–BZY20 support to assist densification of a BZY20 electrolyte at 1450 °C, which is significantly lower than the conventional sintering temperature of >1700 °C required for free-standing films. This approach enabled the fabrication of a dense 5 × 5 cm^2^ BZY20 electrolyte layer with a thickness of 7 μm (Figure 30d), highlighting an innovative route to circumvent the limitations of high-temperature sintering and the use of chemical sintering aids.

Overall, tape casting offers a compelling combination of process control, scalability, and material versatility, positioning it as a leading technique for fabricating high-performance thin-film electrolytes in P-RSOCs. Continued research into shrinkage-matched composites, controlled sintering atmospheres, and defect suppression strategies will be crucial to fully harnessing its potential in both laboratory and industrial settings.

### 5.3 Solution-Based Deposition Methods

Solution-based deposition techniques, including screen printing, spin coating, dip coating, and wet powder spray coating (WPSC), have been widely investigated for depositing proton-conducting electrolyte films onto porous electrode substrates (Figure 31a), typically with film thicknesses ranging between 5 and 30 μm.^424^ By carefully tailoring slurry composition, rheological properties, and deposition parameters, these methods can yield dense, gastight electrolytes postsintering, effectively minimizing ohmic losses at operational temperatures while preserving structural integrity. This section provides a brief introduction to these fabrication methods, discussing their fundamental principles, representative slurry formulations, critical process parameters, and their respective advantages and limitations. We are mainly focused on BaZrO_3−δ_- and BaCeO_3−δ_-based protonic conducting electrolytes, highlighting both physical and electrochemical properties, especially regarding thickness, microstructure, R_o_, and OCV.

**Figure 31:**
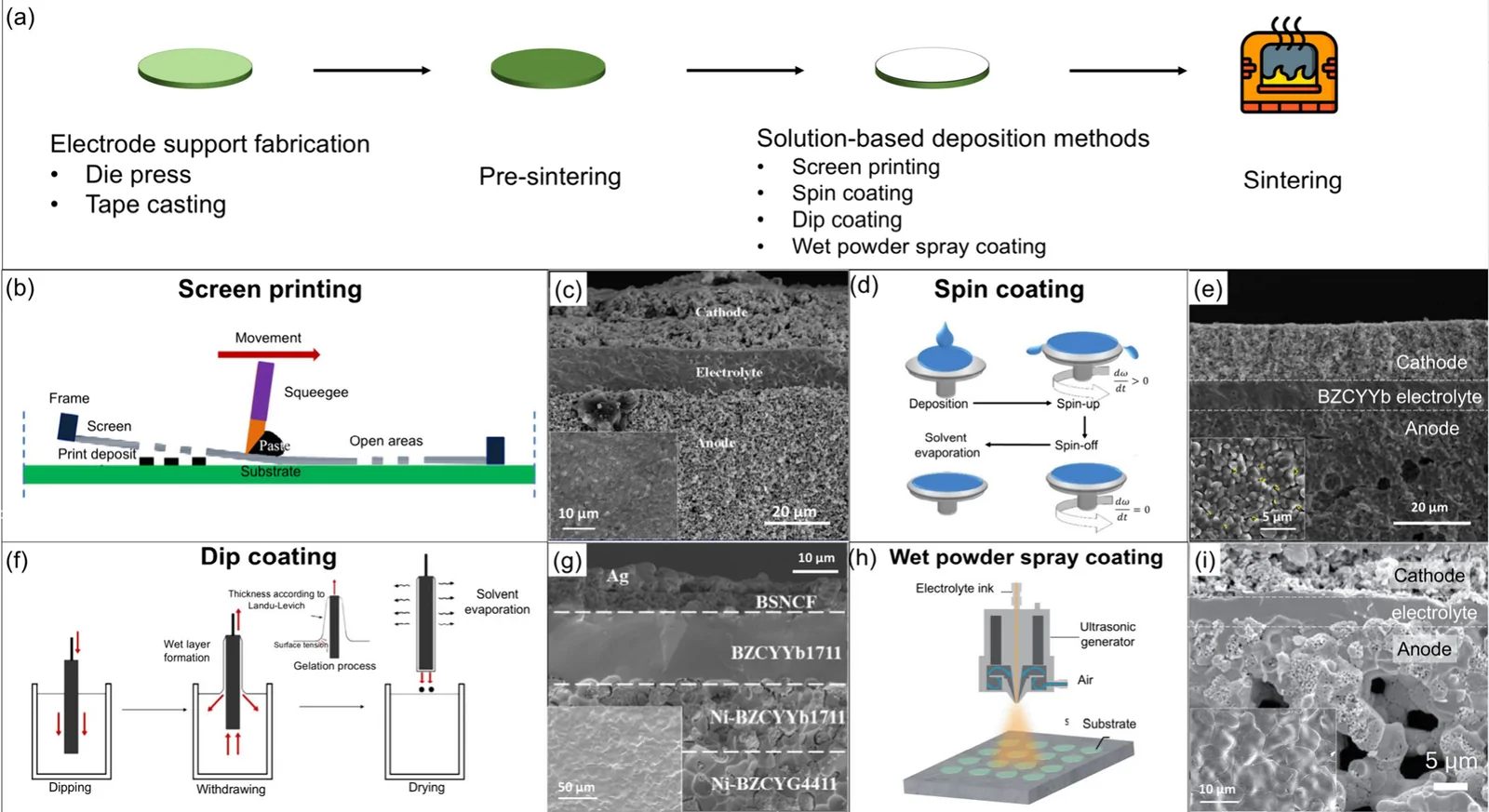
(a) Overall fabrication process using colloidal methods. Schematic diagram of (b) screen printing,^425^ (d) spin coating,^430^ (f) dip coating,^446^ and (h) WPSC methods.^160^ Reproduced with permission from ref 425, 430, 446, 160 . Copyrights from 2017 Elsevier, 2020 Elsevier, 2014 Elsevier, and 2023 Springer Nature. Cross-section microstructure image of an as-prepared cell with (c) BZCY172 electrolyte by screen printing,^429^ (e) BZCYYb2611 electrolyte by spin coating,^434^ (g) tubular BZCYYb1711 electrolyte by dip coating,^439^ and (i) BZCYYb4411 electrolyte by WPSC methods.^160^ All inset: the microstructure of the corresponding electrolyte surface. Reproduced with permission from ref 429, 434, 439, 160 . Copyrights from 2009 Elsevier, 2020 Elsevier, 2025 Energy Materials and 2023 Springer Nature.

#### 5.3.1 Screen Printing

Screen printing is a well-established thick-film fabrication technique that has been adapted for preparing thin-film proton-conducting electrolytes, typically ranging from 5 to 30 μm, on porous fuel electrode substrates. In this process (Figure 31b), a viscous slurry, or “paste,” consisting of electrolyte powders and organic vehicles, is pressed through a patterned mesh screen onto the substrate using a squeegee. The open areas of the mesh precisely define the film geometry, allowing for uniform coatings of controlled thickness over large areas. After printing, the substrates are dried to evaporate solvents and subsequently sintered at high temperatures, removing residual organics and achieving densification through ceramic particle sintering.^425^

The success of this fabrication method hinges critically on the careful formulation of slurry rheology, requiring a balance between sufficient thixotropy, ensuring shape retention after deposition, and adequate fluidity to flow through the screen mesh under shear. Postprinting, the slurry must also fully occupy the channels formed by the warp and weft threads of the screen without spreading undesirably.^426^ Typical electrolyte slurries for screen printing contain a high solid content (40–60 wt %), with fine electrolyte powders dispersed in a solution comprising solvents, dispersants, binders, and plasticizers.^426,427^ Alpha-terpineol is frequently selected as the solvent due to its favorable drying characteristics, while ethyl cellulose commonly serves as a binder, imparting both shear-thinning behavior and film-forming properties. Dispersants, such as fish oil or phosphate esters, and surfactants, notably Triton X-100, stabilize particle dispersions and enhance substrate wetting. Adjustments in particle loading, binder concentration, and solvent composition (occasionally involving alcohols like butanol or isopropanol) help optimize viscosity for smooth printing without slumping or cracking during drying.^426,427^

Screen printing offers considerable advantages due to its simplicity, scalability, and compatibility with established SOFC manufacturing technologies. It enables the uniform coating of extensive surface areas and easily accommodates varying cell geometries through tailored screen designs. Additionally, the relatively low equipment costs and capacity to achieve dense, 10–20 μm-thick electrolyte films in minimal printing passes reduce processing complexity. For instance, Bi et al.^428^ successfully fabricated single cells with ∼ 16 μm-thick BCS electrolyte on NiO–BCS electrode support, achieving gas-tightness upon sintering. These cells demonstrated impressive open-circuit voltages of approximately 1.04 and 1.01 V at operating temperatures of 600 and 700 °C, respectively, closely matching theoretical Nernst potentials.

Moreover, screen printing can be coupled with SSRS methods, where precursor mixtures (e.g., metal oxides and carbonates) are directly screen-printed and subsequently sintered to form dense electrolyte layers. As demonstrated by the preparation of BaZr_0.1_Ce_0.7_Y_0.2_O_3−δ_ electrolytes, precursor slurries containing BaCO_3_, ZrO_2_, CeO_2_, and Y_2_O_3_ printed onto green NiO–BZCY172 substrates achieved dense, defect-free electrolyte films (∼25 μm-thick) upon cofiring at 1400 °C (Figure 31c). Cells fabricated with this method showed superior electrochemical performance, evidenced by high OCV (∼1.01 V) and PPD (382 mW cm^–2^ at 700 °C) when coupled with SmBaCo_2_O_5+δ_ air electrode.^429^ Furthermore, screen printing methods effectively incorporate sintering aids such as NiO or ZnO (1–2 wt %) to improve the densification of electrolyte materials with inherently poor sinterability.

Nevertheless, the fabrication of proton-conducting electrolytes by screen printing faces several challenges, particularly in avoiding defects such as pinholes, microcracks, and delamination that can stem from paste inconsistencies or substrate irregularities. These defects significantly compromise electrolyte gas tightness, resulting in decreased OCV values and heightened local current densities and accelerating performance degradation. While thicker electrolyte films mitigate gas leakage issues, they concomitantly increase R_o_; conversely, thinner films necessitate exceptionally precise slurry formulations and sintering conditions to achieve defect-free layers. Additionally, differential shrinkage rates between electrolyte layers and supporting substrates during sintering can induce interfacial stresses, potentially leading to cracks or delamination.^427^ Mitigating these issues typically involves meticulous sintering profile optimization or the introduction of AFL to ensure uniform shrinkage compatibility.

#### 5.3.2 Spin Coating

Spin coating is a widely adopted solution-based deposition technique for fabricating thin-film proton-conducting electrolyte layers, particularly from colloidal slurries or sol–gel-derived gels. In this method (Figure 31d), a small volume of suspension is dispensed onto a substrate, which is then rapidly rotated at speeds typically ranging from 1000 to 6000 rpm. The centrifugal force spreads the liquid across the substrate, forming a thin, uniform film whose thickness is governed by several parameters including slurry viscosity, solid content, spin speed, and duration. During the process, solvent evaporation and centrifugal thinning synergistically contribute to film uniformity, often yielding dense pinhole-free coatings. Such uniformity is especially beneficial in reducing the R_o_ when the sintered film is fully dense.^430^

There are two predominant approaches to spin-coating protonic electrolytes: (1) sol–gel-based precursors and (2) colloidal powder suspensions. The sol–gel method employs molecular precursors, such as metal nitrates or acetates, dissolved with complexing agents like citric acid and ethylenediaminetetraacetic acid, forming a homogeneous solution that gels upon drying and crystallizes upon sintering. For instance, Xie et al.^431^ synthesized nanocrystalline BaZr_0.8_Y_0.2_O_3_ thin films (∼200 nm thick) at relatively low temperatures (1000 °C) using a sol–gel formulation containing metal–organic precursors and complexing agents, although the work was limited to standalone film characterization and not applied in full-cell configurations. In contrast, the colloidal approach uses presynthesized ceramic powders dispersed in a solvent system along with binders, dispersants, plasticizers, and other rheology modifiers to form a stable suspension. A typical slurry is essentially a diluted screen-printing paste with lower viscosity to facilitate film leveling during spinning. Han et al.^432^ prepared a spin-coating slurry with 50 wt % BZY20 powder, 2 wt % ethyl cellulose, and 48 wt % α-terpineol, which produced thin and smooth green films after coating. Mushtaq et al.^433^ developed a low-viscosity suspension for BaCe_0.2_Zr_0.7_Y_0.1_O_3−δ_ (BCZY271) using 6 wt % solid loading in isopropanol, incorporating polyvinyl butyral (PVB) as binder, dibutyl phthalate as plasticizer, Triton X-100 as surfactant, and ammonium polyacrylate as dispersant. After deposition, the films are dried and cosintered with the substrate, often following thermal profiles similar to those used in screen printing. Due to their thinness, spin-coated films can densify at relatively lower temperatures; however, excessive grain growth or unwanted interdiffusion with the support electrode can occur if sintered at high temperatures.^432^

Spin coating offers distinct advantages in producing high-quality thin electrolyte layers with superior thickness control and surface uniformity. Parameters such as spin speed, acceleration, and solution formulation critically determine the final film quality. Higher spin speeds and longer durations result in thinner films due to increased centrifugal thinning and solvent evaporation. Compared to screen printing, spin coating readily yields sub-10 μm films, and submicron thicknesses are achievable using sol–gel approaches.^431^ Kang et al.^434^ demonstrated this by fabricating a spin-coated BaZr_0.2_Ce_0.6_Y_0.1_Yb_0.1_O_3−δ_ (BZCYYb2611) film with a thickness of 7.6 μm (Figure 31e), achieving an OCV of 1.101 V (very close to the theoretical value) and a low area-specific R_o_ of 0.2 Ω cm^2^ at 600 °C. The ability to create dense, pinhole-free films with minimal equipment requirements makes spin coating an attractive choice for lab-scale development. Moreover, the short diffusion length in such thin layers facilitates sintering, allowing for full densification at temperatures as low as 1600 °C for BZY20 films, which is significantly lower than those required for densifying pellets (>1700 °C).^432^ Spin coating is also adaptable for constructing multilayer architectures by sequential deposition of different functional layers, including barrier or interfacial films.^434^

Despite these merits, the spin coating has several limitations. Achieving film thicknesses in the 5–15 μm range, necessary for minimizing electron leakage, typically requires multiple coatings, which increases processing time and risks introducing interfacial defects. Uniform coating on porous or rough substrates can be challenging unless the slurry is carefully optimized for rheological properties.^433^ Furthermore, spin coating is best suited to planar, symmetrical substrates (e.g., circular disks); applying it to tubular geometries or large-area stack components is nontrivial due to balance and uniformity constraints during spinning. Additionally, significant material wastage occurs as most of the slurry is expelled during spinning, reducing process efficiency. These factors render spin coating a batch process that, while valuable for research and prototyping, is less practical for high-throughput manufacturing compared to scalable, continuous deposition methods such as screen printing or spray coating.

#### 5.3.3 Dip Coating

Dip-coating is a widely utilized and cost-effective technique for fabricating ceramic electrolyte films, particularly for proton-conducting RSOCs. As shown in the schematic diagram in Figure 31f, the process involves immersing a substrate, typically a porous electrode, into a well-dispersed ceramic slurry, dwelling for a specified duration, and withdrawing it at a controlled rate. During withdrawal, a thin liquid film forms on the substrate surface, which subsequently dries and undergoes high-temperature sintering to yield a dense, gastight electrolyte layer.^435^ As one of the oldest and most versatile ceramic coating methods, dip coating offers significant advantages in geometric adaptability, making it especially well-suited for planar, tubular, and other complex or nonplanar configurations.^436^

The typical slurry formulation for dip coating consists of a proton-conducting ceramic powder, such as the state-of-the-art BZCYYb1711, dispersed in a volatile solvent (e.g., ethanol or butanol), combined with organic binders like polyvinylpyrrolidone or PVB, dispersants, and plasticizers to tailor rheology and film-forming behavior.^435,437^ In some cases, minor sintering aids such as NiO or ZnO are introduced to promote densification at lower firing temperatures.^438^ Upon immersion and withdrawal, a liquid meniscus forms around the substrate, and the resulting wet film thickness adheres to the Landau–Levich theory, which relates film thickness to the withdrawal speed, slurry viscosity, surface tension, and solid loading.^435^ By carefully optimizing the withdrawal speed and dwell time, film thicknesses in the range of ∼ 20 μm with uniformity deviations as low as ± 7% can be achieved postsintering.^437^

Dip coating is particularly appealing for fabricating proton-conducting RSOCs due to its geometric versatility and compatibility with both planar and tubular architectures. A prominent enhancement to conventional dip coating is the vacuum-assisted dip coating method, in which a moderate vacuum (∼0.4–0.5 bar) is applied to the backside of the porous support during slurry immersion. This vacuum draws the slurry more uniformly into surface pores, improving contact at the interface and suppressing air bubble entrapment, which significantly enhances adhesion and reduces defect formation.^433^ Electrolyte membranes fabricated via this approach exhibit minimized surface pinholes, cracks, and delamination, resulting in reliable gastight layers that are essential for high open-circuit voltages and low ohmic losses in electrochemical testing

For example, Zhang et al.^438^ demonstrated that vacuum-assisted dip coating of BCZYYb4411 electrolytes yielded defect-free membranes with thicknesses as low as 10 μm and reproducible high OCV (1.08 V at 600 °C), reflecting excellent gas-tightness and structural integrity. Similarly, Mushtaq et al.^433^ applied a vacuum-assisted coating of BCZY271 on NiO-based anodes to fabricate dense and pinhole-free layers, which were used in hydrogen-pumping proton-conducting RSOCs and exhibited current densities up to 525 mA cm^–2^ at 1 V (electrolysis mode) in the range of 350–450 °C.

Dip coating has been effectively employed to fabricate thin and dense electrolyte layers on both planar and tubular anode supports for PCFCs. In a recent study by Zhuang et al.,^439^ a high-performance anode-supported microtubular PCFC was fabricated using a combination of extrusion and dip-coating techniques, demonstrating the viability of this method for producing long and mechanically robust tubular architectures with outer diameters below 5 mm. The fabrication process involved first preparing NiO–BZCYG4411 anode support tubes via extrusion, followed by sequential dip coating of the AFL and the BZCYYb1711 electrolyte layer. To ensure slurry uniformity and prevent intrusion into the tube interior, one end of the tube was sealed with paraffin, and vacuum degassing was applied before coating. The dip-coating procedure, repeated twice for the electrolyte, enabled precise control over film thickness (∼20 μm) and uniformity across the curved surface.

The sintered electrolyte layer exhibited a dense and crack-free microstructure with excellent adhesion to the underlying AFL and anode support. Cross-sectional SEM analysis (Figure 31g) revealed that the dip-coated BZCYYb1711 electrolyte achieved significantly higher densification compared to films prepared by screen printing or spin coating methods. These microstructural advantages translated into superior electrochemical performance, with the cell demonstrating an OCV of 1.085 V at 600 °C in a humidified H_2_–air system, very close to the theoretical value. Furthermore, the cell exhibited a low R_o_ of 0.25 Ω cm^2^ at 600 °C, affirming the effectiveness of the dip-coating process in minimizing electrolyte thickness while maintaining structural integrity.^439^

Despite its utility, dip-coating does present certain challenges. Film thickness can be difficult to precisely control, and capillary and gravitational effects may induce edge riding or an uneven thickness near tube ends or plate edges. Multiple coating cycles are often required to build a dense, defect-free film, and slow, uniform drying is essential to preventing cracking or delamination. Nonetheless, due to its adaptability, low cost, and minimal equipment requirements, dip coating remains a foundational method for depositing proton-conducting electrolyte layers in both laboratory-scale and precommercial P-RSOC devices.

#### 5.3.4 WPSC

WPSC is a versatile, noncontact deposition technique widely applied in the fabrication of proton-conducting RSOCs, particularly for producing thin, dense electrolyte films. In this process, a suspension of ceramic electrolyte powders—often termed “spray ink”—is atomized and deposited onto a substrate to form a green film that is subsequently dried and sintered into a gastight electrolyte layer (Figure 31h).^160^ WPSC is especially attractive due to its compatibility with both planar and tubular geometries, scalability to large-area deposition, and precise control over film morphology and thickness.^160,440^

A typical spray ink formulation includes a proton-conducting ceramic powder such as BZCYYb1711, combined with volatile organic solvents (e.g., ethanol, toluene, or isopropanol), polymeric binders (e.g., PVB or ethyl cellulose), plasticizers (e.g., butyl benzyl phthalate), and dispersants (e.g., fish oil) to tailor the ink’s rheology for effective atomization.^441,442^ Process parameters such as atomization method (ultrasonic or pneumatic), spray flow rate, nozzle-substrate distance, droplet size, and substrate temperature are meticulously optimized to achieve film uniformity and prevent defects such as mud cracking or delamination. Recent advances have demonstrated sub-10 μm electrolyte layers with thickness control enabled by modulating the number of spray passes and solid loading levels.^442^

For instance, Liu et al.^160^ utilized ultrasonic spray coating to deposit a ∼ 3.5 μm-thick BZCYYb4411 electrolyte on a green anode substrate (Figure 31i). The resulting film exhibited a dense, crack-free bamboo-like microstructure with average grain sizes of ∼ 8 μm and achieved an OCV exceeding 1.05 V at 600 °C in H_2_–air conditions. In a similar demonstration, Taillades et al.^440^ applied WPSC with a simple isopropanol-based ink (10 wt % BZCYYb1711 without binders or plasticizers), achieving a dense ∼ 4 μm-thick electrolyte on planar anode support and reporting peak power densities up to 634 mW cm^–2^ at 700 °C.

WPSC’s scalability has also been demonstrated through its integration with roll-to-roll fabrication and tubular geometries using rotatable jigs, supporting its potential for industrial-scale manufacturing.^442^ Furthermore, its noncontact nature allows deposition on fragile or curved substrates without mechanical damage. Recent innovations, such as the incorporation of ultrasonic atomization, have enabled finer control over the droplet size and film homogeneity.^160,443^ Electrostatic spray deposition variants have also been employed to fabricate porosity-graded cathodes or dense electrolyte membranes with improved long-term stability.^444,445^

Despite these advantages, challenges remain. Achieving crack-free and uniformly dense coatings requires careful balancing of the ink solid content, droplet overlap, and drying behavior. Oversaturation can result in film delamination or mud cracking, while a low solids loading may lead to incomplete coverage or pinholes. Moreover, substrates must exhibit sufficient mechanical strength to endure multiple spray and drying cycles, particularly for binder-burnout or cosintered supports. Finally, the need for precision-controlled spray heads, motion stages, and atomization equipment adds to the system complexity and cost.

### 5.4 Physical Vapor Deposition Methods

Physical vapor deposition (PVD) techniques have emerged as powerful methods for fabricating ultrathin, dense proton-conducting electrolyte films, offering precise control over the film thickness, composition, and microstructure. Compared to traditional slurry-based approaches, PVD techniques enable the deposition of submicrometer films with excellent uniformity and reduced sintering requirements. Key PVD methods include magnetron sputtering and PLD, while other approaches, such as electron-beam evaporation and aerosol deposition, have also been investigated, although less frequently. These methods are particularly effective in reducing electrolyte R_o_ by enabling shorter and more optimized ion transport pathways, thereby minimizing R_o_ in single-cell electrolyte films. The influence of these effects is discussed in detail in the context of each specific PVD technique.

#### 5.4.1 Magnetron Sputtering

Magnetron sputtering has been extensively utilized to deposit dense thin films of BaZrO_3−δ_- and BaCeO_3−δ_-based proton-conducting electrolytes. As depicted in Figure 32a, in the sputtering process, a target composed of the desired electrolyte (or its constituents) is bombarded by energetic ions (typically Ar gas in a low-pressure glow discharge), ejecting target atoms which then condense on a substrate to form a film.^447^ For insulating ceramic targets, radio frequency (RF) sputtering is preferred over direct current to sustain plasma. Film deposition typically occurs at substrate temperatures between 200–600 °C to facilitate adatom mobility and promote densification.^28,448^

**Figure 32:**
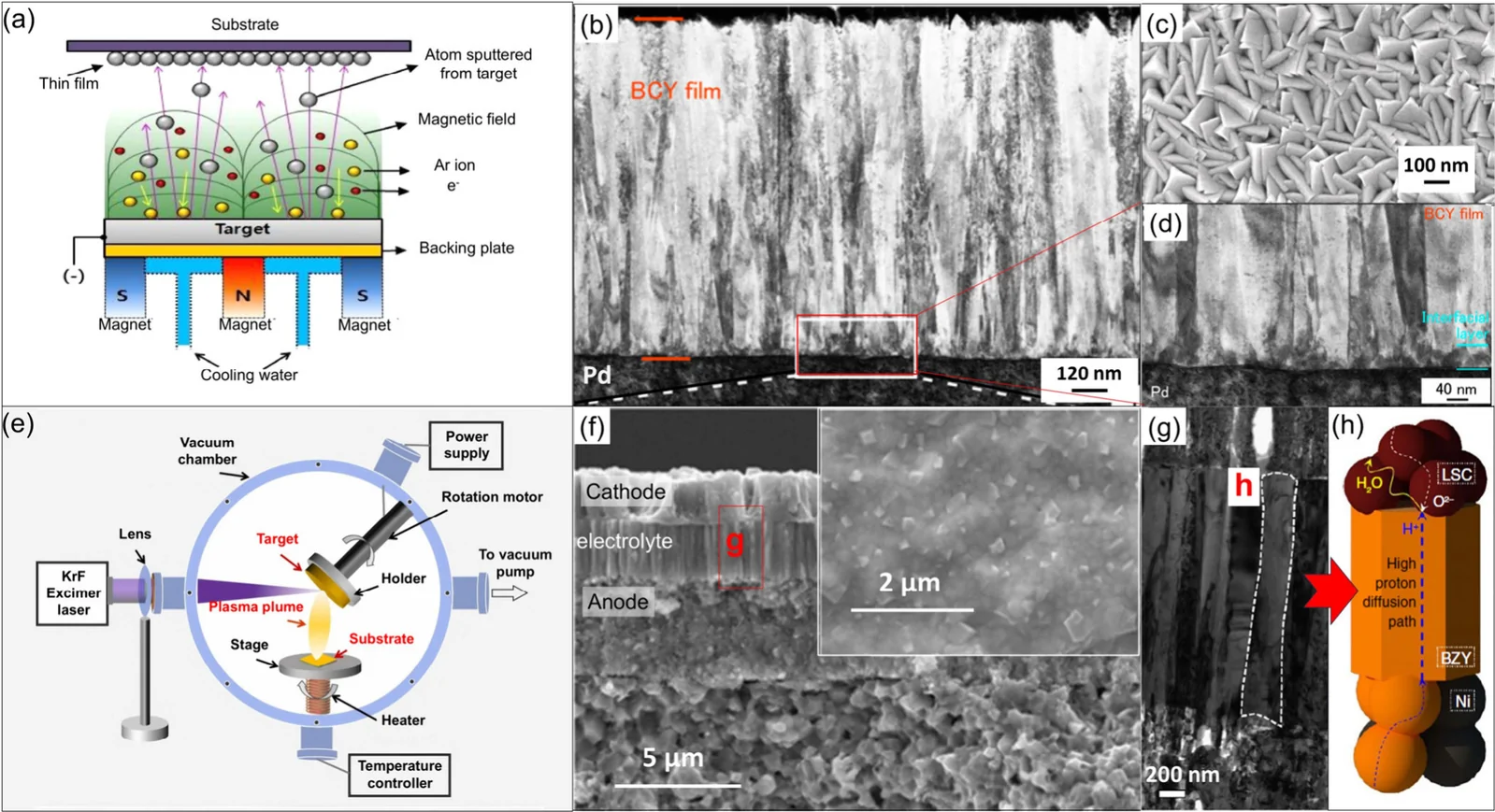
(a) Schematic diagram of a typical sputtering process. Reproduced with permission from ref 461. Copyrights 2021 Elsevier. (b) Cross-sectional TEM image and (c) surface SEM image of BCY20 thin film deposited on a Pd substrate by RF cosputtering. (d) Expansion of the region around the BCY20/Pd interface. Reproduced from ref 448. Copyright 2016 American Chemical Society. (e) Schematic diagram of a typical PLD process. Reproduced with permission from ref 462. Copyrights 2025 Elsevier. (f) Cross-sectional image of BZY20 electrolyte deposited by PLD. Inset: microstructure of the electrolyte surface (g) Bright-field image of dense BZY20 electrolyte in the middle and nanoporous electrodes, showing single-column microstructure. (h) A schematic diagram of a single column in the thin BZY20 electrolyte and the neighboring electrode grains in the fuel cell configuration with a possible charge transport path. Reproduced from ref 458. Available under a CC-BY 4.0 license. Copyright 2017 Springer Nature Kiho Bae et al.

Owaku et al.^449^ reported the deposition of ∼ 210 nm BaCe_0.9_Y_0.1_O_3−δ_ films via RF sputtering on Al_2_O_3_ substrates, yielding nanocrystalline films with grain sizes around 80 nm and smooth surfaces after postannealing at 300–500 °C. Similarly, Pornprasertsu et al.^450^ utilized a two-step RF/DC sputtering process to grow BaZrO_3−δ_-based films, which crystallized into the perovskite phase after annealing at 800 °C for 3 h. However, sputtered films often suffer from stoichiometric deviations, particularly Ba deficiency, due to differences in sputtering yields and oxygen nonequilibrium conditions.^451^ These deviations significantly impact phase stability and proton conductivity.^452^

To address this, researchers have employed cosputtering approaches. Lescure et al.^453^ deposited BZY20 by cosputtering metallic Ba and Zr_0.8_Y_0.2_ targets with a DC power source, achieving dense, columnar films of ∼ 4 μm thickness. Similarly, a 110 nm BaHf_0.8_Yb_0.2_O_3−δ_ protective layer was recently fabricated by cosputtering metallic Ba and BHYb82 ceramic targets on a BZCYYb1711 electrolyte, forming a uniform, adherent barrier layer. Due to the amorphous or poorly crystalline nature of as-deposited films, postdeposition annealing (typically 800–1000 °C) is often required to induce crystallization and activate proton conductivity. To avoid conventional high-temperature sintering (>1400 °C), Wallis et al.^454^ developed an *in situ* laser-assisted annealing method, producing crystalline BZY20 films directly on Ni-BZY20 substrates at only 800 °C.

Aoki et al.^448^ demonstrated the fabrication of a ∼ 1 μm-thick BCY20 thin film electrolyte using RF cosputtering of BaCe_0.8_Y_0.2_O3 and Ce_0.9_Y_0.1_O_2_ targets onto a hydrogen-permeable Pd substrate. Cross-sectional transmission (Figure 32b) and electrolyte top-view (Figure 32c,d) electron microscopy revealed a distinctive “bamboo-like” columnar morphology, with vertically aligned grains several hundred nanometers in width growing perpendicular to the substrate. This microstructure resulted from nucleation on Pd nanoparticles, followed by competitive grain growth, ultimately yielding a dense, crack-free film with tightly packed grain boundaries. The architecture enabled enhanced proton transport through aligned, low-resistance pathways.

The resulting SOC, composed of a Pd/BCY20/LSCF configuration, exhibited an open-circuit voltage of 1.08 V and an exceptional PPD of 1.05 W cm^–2^ at 600 °C in humidified H_2_-air conditions. The R_o_, derived from high-frequency intercepts in impedance spectra, was as low as 0.25 Ω cm^2^. Importantly, impedance analysis revealed that cathodic charge-transfer resistance was 25 times smaller than in conventional PCFCs, underscoring the advantage of the sputtered film’s TPB integration and minimized microstructural heterogeneity (Figure 32d).^448^

Furthermore, Lescure et al.^453^ employed reactive magnetron sputtering to deposit a 4 μm-thick BZY20 layer on a tape-cast NiO–BZY20 anode support, achieving a dense columnar film structure without pinholes or delamination. The electrolyte deposition was performed using cosputtering of metallic Ba and Zr_0.8_Y_0.2_ targets under a controlled Ar–O_2_ atmosphere at 1.2 Pa, with substrate heating at 560 °C. Postdeposition annealing at 1000 °C for 2 h ensured densification and phase stabilization. Electrochemical testing of the resulting single cell (NiO–BZY20/BZY20/BZY20–BSCF) showed a relatively high R_o_ of 7 Ω cm^2^ at 525 °C but remarkably low R_p_ (0.3 Ω cm^2^), attributed to the clean electrolyte–electrode interface and the well-developed microstructure. The performance could be further optimized through adjustments in electrolyte thickness and substrate preparation.

Overall, magnetron sputtering enables the deposition of compositionally controlled, dense, and microstructurally tailored electrolyte films with submicrometer to few-micron thicknesses. Its advantages include (1) decoupling of densification from high-temperature sintering, thus reducing BaO volatility and interfacial interdiffusion, (2) compatibility with chemically sensitive substrates, and (3) potential integration with roll-to-roll or microfabricated platforms. However, challenges such as limited deposition rates, the need for precise stoichiometry control via multitarget setups, and scalability to large-area or three-dimensional substrates must be addressed for broader implementation in commercial P-RSOCs manufacturing.

#### 5.4.2 PLD

PLD has emerged as a powerful PVD technique for the fabrication of high-purity, stoichiometric, and structurally tailored proton-conducting ceramic electrolyte films. In PLD, a high-energy pulsed laser, typically a KrF excimer laser (λ = 248 nm), ablates a dense ceramic target in a vacuum chamber, creating a transient plasma plume that deposits material onto a heated substrate (Figure 32e). Unlike magnetron sputtering, PLD enables congruent transfer of multicomponent oxide targets, thus maintaining the desired cationic stoichiometry even in complex compositions.^455,456^

One of PLD’s greatest strengths lies in its ability to produce highly crystalline, epitaxially oriented films with minimal grain boundaries. Pergolesi et al.^290^ reported a 1 μm-thick epitaxial BZY20 film deposited on (100)-oriented MgO that exhibited grain-boundary-free columnar growth throughout the film thickness. XRD and TEM analyses confirmed a single-crystalline columnar morphology, aligned normally to the substrate, which facilitated direct proton migration along low-resistance pathways. EIS revealed an exceptional proton conductivity of 0.11 S cm^–1^ at 500 °C, nearly 1 order of magnitude higher than that of polycrystalline BZY20 sintered ceramics and exceeding the conductivity of oxygen-ion conductors in the same temperature range. The study demonstrated that eliminating grain boundary blocking, typically associated with charge carrier trapping and reduced conductivity, could unlock the intrinsic transport properties of proton-conducting electrolyte material for intermediate-temperature proton-conducting RSOCs.

Building upon this foundation, Bae et al.^457^ fabricated thin-film electrolyte layers using PLD in combination with optimized anode structures. In 2016, a 2 μm-thick BaCe_0.5_Zr_0.35_Y_0.15_O_3−δ_ electrolyte was deposited onto a graded Ni–BaCe_0.5_Zr_0.35_Y_0.15_O_3−δ_ anode support, yielding a dense, pinhole-free film with excellent interfacial adhesion. The corresponding single cell with a La_0.6_Sr_0.4_CoO_3−δ_ air electrode achieved a PPD of 625 mW cm^–2^ and an OCV of over 1 V at 600 °C.^457^ In a follow-up study, the same group produced a 2.5 μm-thick BaZr_0.85_Y_0.15_O_3−δ_ (BZY15) film with vertically aligned columnar grains tightly integrated onto nanoengineered electrode surfaces (Figure 32f).^458^ This columnar architecture effectively suppressed the formation of high-resistance grain boundaries, producing a high proton diffusion path and maintaining gas tightness under operation (Figure 32g,h). The cell demonstrated an even higher PPD of 740 mW cm^–2^ and a R_o_ of only ∼ 0.1 Ω cm^2^, maintaining an OCV of ∼ 1.0 V at 600 °C, among the lowest R_o_ values for BZY20-based electrolyte to date.

Beyond high performance, PLD also enables fundamental studies of strain engineering in proton-conducting films. Fluri et al.^459^ explored the effects of biaxial strain on epitaxial BZY20 films, revealing that tensile strain (up to +0.7%) significantly lowered the activation energy for proton hopping by ∼ 0.07 eV, thereby doubling the conductivity at 200 °C compared to the compressive-strained counterpart. Complementary first-principles MD simulations attributed this enhancement to increased proton diffusivity in the direction perpendicular to the strained lattice plane.

Microstructural control via PLD is highly sensitive to deposition conditions, such as substrate temperature and ambient oxygen pressure. Chang et al.^460^ reported that crystalline and dense BZY20 films formed only when the substrate temperature exceeded 500 °C and oxygen pressure was kept below 0.013 mbar. In contrast, lower temperatures or elevated oxygen pressures led to columnar or porous morphologies with limited gas impermeability.

While PLD excels in producing high-purity, grain-boundary-free thin films with tailored orientation and microstructure, it remains constrained by relatively slow deposition rates and limited scalability to large-area or commercial substrates. Nevertheless, PLD continues to serve as a benchmark technique for optimizing the electrolyte performance and elucidating fundamental transport mechanisms. Ongoing innovations, including high-power impulse PLD, laser sintering, and hybrid deposition platforms, are actively being explored to translate these insights into scalable manufacturing processes.

### 5.5 CVD

CVD techniques enable the formation of thin, conformal electrolyte films by delivering vapor-phase precursors to a heated substrate, where they chemically react or decompose to form a solid phase. CVD methods are advantageous for coating substrates with complex geometries, including porous or tubular supports due to their excellent step coverage and uniformity. Two principal CVD categories applied to solid-oxide systems are conventional CVD and ALD. Compared with PVD techniques, CVD approaches can yield more conformal coatings and allow for the fabrication of ultrathin films with fewer defects, particularly on 3D-structured substrates.

#### 5.5.1 Conventional CVD

CVD is a vapor-phase film deposition technique in which volatile precursors decompose or react on a heated substrate surface to form a thin solid film. It enables highly conformal, compositionally uniform coatings on both planar and complex surfaces. While extensively used in oxide electronics, its application in proton-conducting electrolytes remains limited because of the difficulty of phase formation at moderate temperatures and Ba volatility.

Pellegrino et al.^154^ recently reported the fabrication of dense BCY20 thin films via metal–organic CVD using β-diketonate precursors (i.e., Ba­(hfa)_2_·tetraglyme, Ce­(hfa)_3_·diglyme, and Y­(hfa)_3_·diglyme), which showed clean thermal volatilization and excellent suitability for vapor-phase transport. Films deposited at 950 °C on YSZ and MgO substrates yielded phase-pure orthorhombic BCY20 with compact nanocrystalline morphology (crystallite size ∼ 42 nm). Lower deposition temperatures led to secondary phases like BaF_2_ and CeO_2_, highlighting the importance of thermal optimization. EDS mapping confirmed chemical uniformity and strong film adhesion, demonstrating the potential of metal–organic CVD for producing high-quality protonic thin films.

To reduce processing temperatures, Sakai et al.^463^ employed laser-assisted CVD (Figure 33a) for BZY20, where precursor decomposition is locally activated by a focused 1064 nm Nd-doped yttrium aluminum garnet laser during deposition at ∼ 500–650 °C. This method achieved high deposition rates, forming dense 16 μm-thick films in just 10 min. XRD and SEM confirmed polycrystalline perovskite formation with strongly oriented and densely packed columnar grains. EDS revealed a homogeneous elemental distribution without secondary phases (Figure 33b). Despite these structural merits, the laser-assisted CVD films showed lower proton conductivity than PLD-grown or bulk-sintered BZY20, likely due to R_gb_, possible Ba deficiency at interfaces, or incomplete proton incorporation.^463^ Nonetheless, the fast deposition, reduced thermal budget, and ability to deposit over large or curved areas make laser-assisted CVD a promising candidate for scalable fabrication.

**Figure 33:**
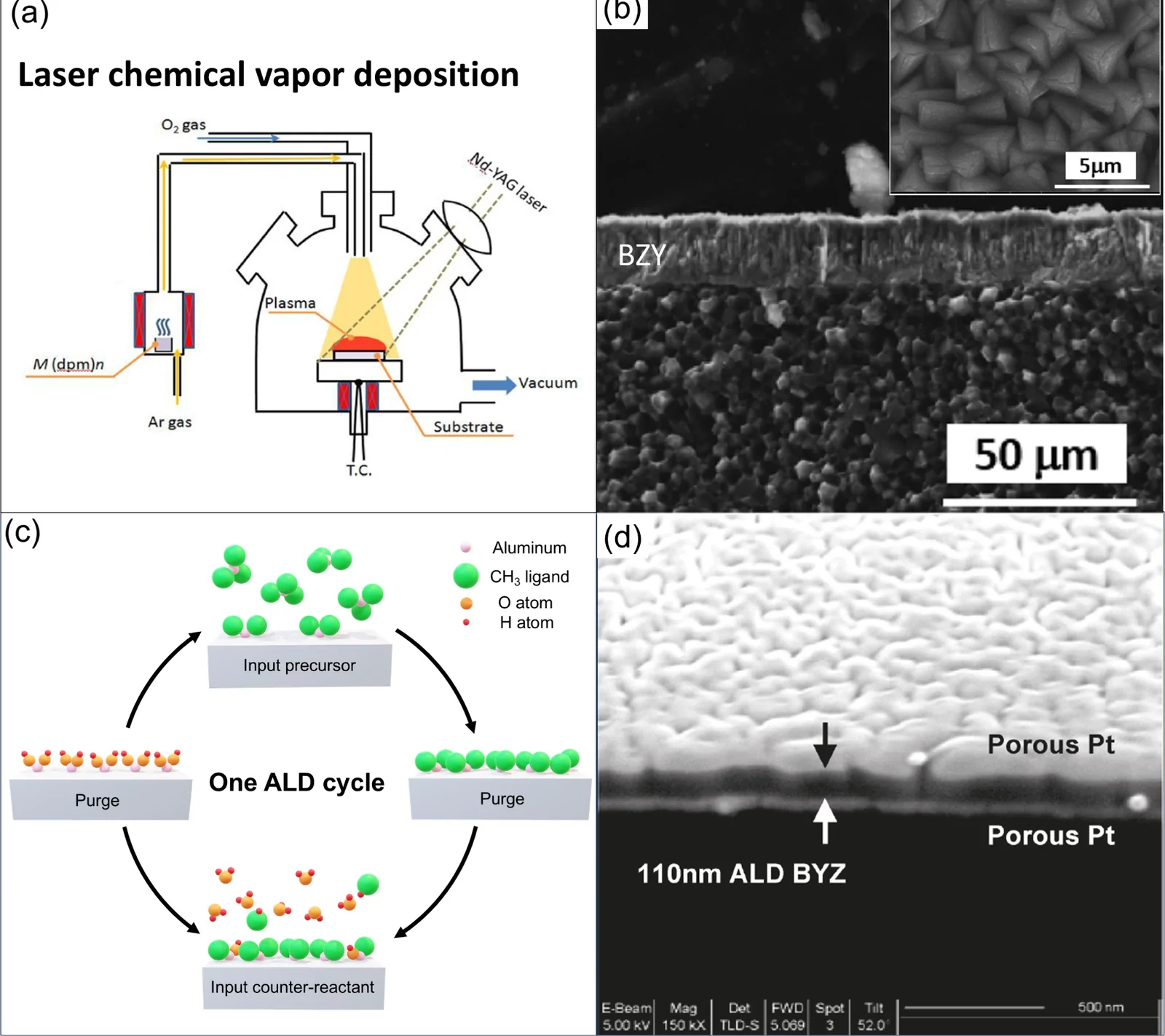
(a) Schematic diagram of the laser-assisted CVD process. (b) Cross-section SEM images of the BZY20 film deposited on the AIN substrate by laser-assisted CVD. Inset: microstructure of the electrolyte surface. (c) Schematic diagram of the ALD process. Reproduced with permission from ref 463. Copyright 2020 Elsevier. (d) Cross-section SEM image of freestanding Pt/BZY20/Pt assembly with BZY20 deposited by ALD. Reproduced from ref 471. Copyright 2009 American Chemical Society.

While CVD has been extensively used in oxygen-ion-conducting systems,^464,465^ its application to P-RSOCs remains limited. To date, no studies have demonstrated the use of CVD for fabricating dense proton-conducting electrolyte films in fully integrated single cells. Nonetheless, the method’s excellent conformality, scalability, and compatibility with both planar and tubular cell geometries make it a promising candidate for future development in RSOC systems.

#### 5.5.2 ALD

ALD is a vapor-phase film deposition technique based on sequential, self-limiting surface reactions between gas-phase precursors, enabling atomic-level control over film thickness and composition. As illustrated in Figure 33c, each ALD cycle consists of two half-reactions: (1) a metal precursor is pulsed into the reaction chamber and chemisorbs onto available surface sites; (2) following a purge with inert gas, a counter-reactant (e.g., H_2_O, O_3_, or O_2_ plasma) is introduced to complete the surface reaction. This cycle deposits a single atomic layer (or less), and the process is repeated to build up films with Ångström-scale precision. ALD typically operates under sub-Torr pressure and within a defined thermal window (∼100–350 °C), which ensures self-limiting growth kinetics.^466–468^

The key advantages of ALD are exceptional conformality, uniformity across high-aspect-ratio structures, and precise stoichiometric control—critical features for electrolyte film deposition in solid oxide cells. ALD has been widely used for depositing ultrathin oxide-ion-conducting layers, especially YSZ, with thicknesses down to 25–100 nm. For instance, Chao et al.^469^ deposited 80 nm-thick YSZ films on nanostructured supports and achieved a PPD of 1.34 W cm^–2^ at 500 °C and a R_o_ of 0.05 Ω cm^2^ at 450 °C. Similar high-performance results have been demonstrated in ALD-based composite electrolytes, where ALD-grown YSZ layers are embedded within sputtered SDC matrices to improve ionic conductivity and mechanical integrity.^470^

Despite its maturity in oxide-ion systems, ALD for proton-conducting electrolytes has remained underexplored due to difficulties in identifying suitable precursors, particularly for BaO. However, a landmark study by Shim et al.^471^ demonstrated the first successful deposition of a proton-conducting BZY20 thin film via ALD, using organometallic precursors and postdeposition crystallization to form the perovskite phase. In their work, a ∼ 100 nm BZY20 film was deposited on micromachined silicon substrates and integrated into a full fuel cell device with sputtered Pt electrodes (Figure 33d). The resulting cell exhibited outstanding performance at intermediate temperatures: a PPD of 136 m W cm^–2^ and an OCV of 1.09 V at 400 °C in H_2_–air operation. This performance surpassed that of a control sample fabricated via PLD, which achieved only 120 mW cm^–2^ at 450 °C. The superior electrochemical response of the ALD-derived cell was attributed to smoother surfaces, better interfacial adhesion, and minimized R_gb_—features inherently enabled by ALD’s conformal and pinhole-free growth.

These results demonstrate that ALD can produce functional, proton-conducting electrolytes at low temperatures and submicron thickness, highlighting its unique advantages for miniaturized or microfabricated RSOCs architectures. Further optimization—such as improved Ba precursors, ternary ALD cycles, and postdeposition annealing—could expand ALD’s utility for a wider range of perovskite compositions and scalable device integration.^466,472,473^ Moreover, as reviewed by Shin et al.,^466^ ALD provides opportunities not only for dense electrolyte deposition but also for interface engineering at the electrode–electrolyte boundary. Tailoring interfacial chemistry and grain boundary character via nanolaminates or dopant gradients could significantly enhance proton conductivity and durability under RSOC cycling.

In summary, while ALD is still in its early stages for protonic systems, the work of Shim et al.^471^ validates its feasibility. As precursor libraries grow and multicomponent ALD techniques mature, this method may become a key enabler for the next generation of ultrathin, low-temperature, and high-efficiency proton-conducting electrolytes.

### 5.6 Sintering Technique

Sintering plays a critical role in fabricating dense, gastight proton-conducting electrolyte layers for RSOCs. The sintering method impacts the microstructure, grain boundary density, and final proton conductivity. Below, we summarize conventional and emerging sintering techniques with attention to their applicability to typical proton-conducting electrolyte materials.

#### 5.6.1 Conventional Sintering

Conventional sintering is the most widely used method to densify proton-conducting ceramic electrolytes. It typically involves forming a green body—such as a pressed pellet, tape-cast layer, or colloidally deposited film on electrode support—followed by thermal treatment in a furnace at elevated temperatures for extended durations, often exceeding 10 h.^474^ For BaZrO_3−δ_-based materials, particularly BZY20, the firing temperature required to achieve full densification is extremely high, typically ranging from 1600 to 1700 °C.^452^ These harsh thermal conditions result in significant barium volatilization, leading to nonstoichiometry, reduced proton conductivity, and structural degradation.

To mitigate BaO evaporation, sacrificial powder beds rich in Ba are commonly employed during sintering to maintain equilibrium vapor pressure and minimize composition loss. Even with such measures, some compositions (e.g., BaZr_0.7_Ce_0.2_Y_0.1_O_3−δ_) still require >10 h at ∼ 1500 °C for sufficient densification. To lower the sintering temperature and time, sintering aids such as NiO, ZnO, CuO, and Fe_2_O_3_ have been introduced. These additives form transient liquid phases or enhance grain-boundary diffusion to promote densification. Chen et al.^292^ demonstrated that 2 mol % NiO enabled dense sintering of BZY20 and BZCY172 at ∼ 1400 °C. Hagy et al.^475^ showed that 1–3 wt % ZnO enabled full densification of BZY20 at 1500 °C, significantly reducing the thermal load compared to ZnO-free systems, which required sintering up to 1700 °C.

For electrolyte materials containing Ba, the Ba loss is always a concern in the high-temperature sintering process. Therefore, an additional sacrificial powder bed is essential to minimize the BaO evaporation during this process.^80^ However, some types of electrolyte materials require sintering under high temperatures for a long time. For example, Y-doped barium-cerate-zirconate pellets may require firing at ∼ 1500 °C for >10 h to reach high density, which is challenging due to BaO loss.^452,474^ An alternative sintering process that requires minimum time exposed to high temperature is required. Recently, cosintering approaches have been adopted, where the shrinkage of a supporting electrode structure during firing assists the densification of a thin electrolyte film. This method aligns thermal expansion and densification rates between layers, helping prevent delamination and reducing overall porosity.^291^

Despite its widespread use, conventional sintering suffers from long processing times, high energy consumption, and difficulties in cofiring multiple layers without interfacial degradation. Ni coarsening, interdiffusion, and mechanical mismatch between the layers are common concerns. These drawbacks motivate the exploration of alternative sintering methods with reduced thermal budgets and an improved microstructural control.

#### 5.6.2 Rapid Laser Reactive Sintering

Rapid laser reactive sintering (RLRS) is an advanced fabrication technique developed to overcome the high-temperature and long-duration limitations of conventional sintering for proton-conducting ceramic electrolytes. RLRS utilizes a high-energy CO_2_ laser (λ = 10.6 μm) to deliver localized heating, enabling the concurrent synthesis and densification of green films within seconds. Unlike traditional furnace sintering, which typically requires temperatures above 1600 °C for more than 10 h, RLRS can achieve comparable microstructural outcomes in under 10 s.^474,476,477^

In this process, the green electrolyte layer is often composed of a stoichiometric mixture of raw oxides or carbonates (e.g., BaCO_3_, ZrO_2_, CeO_2_, Y_2_O_3_, Yb_2_O_3_) that undergo simultaneous phase formation and densification upon laser scanning. Preheating the substrate to moderate temperatures (∼500 °C) is typically employed to prevent thermal shock. Mu et al.^476^ demonstrated that BCZYYb1711 films could be sintered to >95% relative density within 3 s by tuning laser power and scan rate, achieving microstructures comparable to those obtained via SSRS but with drastically reduced Ba volatilization and interdiffusion. Ishii et al. further showed that RLRS-derived BaCe_0.7_Zr_0.1_Y_0.07_Sm_0.13_O_3−δ_ electrolytes exhibited ∼ 97% relative density and robust grain structures, yielding electrochemical properties similar to those from long-term sintering.^478^

Notably, RLRS is compatible with sintering aids such as NiO, which promotes grain growth and enhances proton conductivity. NiO-assisted BZY20 films reached 98.9% relative density and exhibited nearly grain-boundary-free microstructures conducive to high proton conductivity.^474^ Additionally, the microstructures produced via RLRS often show hierarchical features, including dense bulk regions and adjacent porous zones, which can be leveraged for monolithic electrolyte–electrode architectures.^477^

Despite these advantages, ultrafast processing, minimal energy consumption, suppressed Ba loss, and potential for additive manufacturing, RLRS poses challenges such as the need for precisely controlled laser parameters, limited scalability for large-area or batch production, and the necessity to optimize slurry optical absorption properties. Nevertheless, RLRS represents a transformative path for the sustainable and tunable fabrication of high-performance protonic ceramic devices.

#### 5.6.3 MWS

MWS offers an alternative route for densifying proton-conducting ceramics through volumetric heating driven by dielectric losses at 2.45 GHz. This technique facilitates rapid internal heating—achieving rates up to 500 °C min^–1^—resulting in substantial reductions in overall processing time and energy consumption compared to conventional sintering.^475^

In studies using BaZr_0.7_Ce_0.2_Y_0.1_O_3−δ_, the incorporation of NiO or ZnO as sintering aids has enabled dense pellet formation (>97% relative density) at temperatures as low as 1400–1500 °C within 30 min of microwave exposure. The addition of 2–4 mol % ZnO was shown to improve phase purity and mitigate the formation of eutectic liquid phases that degrade conductivity. Notably, samples microwave-sintered with 2 mol % ZnO exhibited uniform microstructures and total conductivity of ∼ 1.2 mS cm^–1^ at 550 °C, rivaling or surpassing those from conventional sintering.^475,479^

Similarly, BZCY721 samples sintered with 2 mol % NiO (BZCYN) at 1500 °C using a microwave heating rate of 500 °C min^–1^ achieved uniform grains and minimized secondary phases. Raman spectroscopy confirmed the retention of a defect-rich perovskite lattice, with pronounced bands at ∼ 630 cm^–1^ indicative of enhanced oxygen vacancy formation, thereby facilitating proton transport.^479^ Furthermore, Wang et al.^480^ demonstrated that microwave-sintered BZCYN cells achieved larger average grain sizes (∼3.8 μm) and significantly lower R_gb_ compared to conventionally sintered counterparts (1.3 μm).

MWS also offers the benefit of suppressing undesirable interfacial diffusion, such as Ni migration from the anode to the electrolyte, due to its shorter dwell times. When coupled with presintering binder burnout at moderate temperatures (∼600 °C), MWS yields dense, crack-free electrolytes while preserving substrate integrity.^475,479^

Nevertheless, technical challenges remain in ensuring uniform energy absorption, managing rapid densification kinetics, and avoiding localized overheating. Advanced cavity designs and dielectric susceptors (e.g., SiC) are often employed to stabilize the thermal field. Despite these hurdles, MWS presents a highly energy-efficient and structurally beneficial approach for fabricating proton-conducting oxide electrolytes, particularly when combined with sintering-aid strategies.

#### 5.6.4 Other Sintering Techniques

In addition to the sintering methods previously discussed, several innovative techniques have emerged over the past decade to address processing challenges inherent to conventional firing, particularly by employing external fields or novel low-temperature strategies.^481^

SPS leverages pulsed direct current combined with uniaxial pressure, rapidly heating electrolyte powders through localized spark discharges between particles, while the applied pressure promotes effective particle rearrangement. Consequently, SPS can achieve full densification at temperatures approximately 400–500 °C below traditional methods, often within minutes.^482^ This rapid heating and cooling process facilitates controlled grain growth, yielding highly dense nanostructured electrolytes with comparable proton conductivities to conventionally sintered counterparts. However, scalability and feasibility challenges remain, primarily due to limitations imposed by graphite die sizes, equipment cost, potential carbon contamination, and the necessity for carefully controlled current distribution to ensure uniform heating throughout the material.^483^

Flash sintering, or field/current-assisted sintering, employs a strong electric field and current through ceramic compacts during heating.^484^ At a critical temperature and field strength, the ceramic undergoes rapid densification within minutes, marked by a notable current surge or “flash.” For instance, the advanced proton-conducting electrolyte BZCYYb1711 achieves full densification at just 850 °C within 1 h using flash sintering, significantly lower than conventional sintering temperatures (∼1500 °C) required for comparable densities.^485^ The primary advantages of this technique include substantially reduced processing times, lower energy consumption, and refined microstructural control. Nonetheless, controlling flash sintering presents challenges, such as potential thermal runaway, cracking, or structural inhomogeneities resulting from rapid current fluctuations. Additionally, scale-up from small, specialized electrode setups remains challenging. Despite these issues, optimized flash sintering protocols offer considerable promise for rapid, energy-efficient manufacturing of protonic ceramics.

Hot-press sintering concurrently applies heat and uniaxial pressure to ceramic powders, achieving dense, uniform electrolyte layers at lower temperatures and shorter durations compared to conventional sintering methods. Advantages include precise control over the microstructure, improved mechanical properties, and enhanced ionic conductivity. Nevertheless, the technique faces practical constraints, such as limitations in size and shape dictated by die geometry, potential contamination from pressing dies, and density gradients in the larger specimens. Recent developments demonstrate the successful integration of hot pressing with multilayer tape casting to fabricate large-area protonic electrolytes for RSOCs.^486^ These optimized structures exhibit excellent electrochemical performance, robust thermal cycling stability, and improved manufacturing efficiency, marking significant progress toward commercial viability.

### 5.7 Other Fabrication Methods

Beyond the techniques discussed previously, several novel electrolyte fabrication methods have gained attention for thin-film proton-conducting electrolytes in the RSOCs. Since this Review primarily focuses on electrolyte materials rather than complete cell fabrication, additional techniques with only brief introductions are highlighted below.

Drop coating, also known as drop casting, is a straightforward solution-based deposition method. It involves dispensing a precursor suspension or solution onto a substrate, followed by drying and sintering to achieve a dense electrolyte film. Sun et al.^487^ successfully applied drop coating to form fully dense BaZr_0.8_Y_0.15_In_0.05_O_3−δ_ electrolyte layers approximately 12 μm thick on NiO–BZY20 electrode substrates. These cells demonstrated excellent electrochemical performance, with a PPD of 379 mW cm^–2^, R_o_ of 0.34 Ω cm^2^, and OCV of 0.93 V at 700 °C. Similarly, Azad et al.^488^ fabricated thin BaCe_0.7_Zr_0.1_Y_0.15_Zn_0.05_O_3−δ_ electrolyte layers via drop coating, achieving notably high-power densities of up to 872 mW cm^–2^ at 700 °C. Despite its simplicity and cost-effectiveness, drop coating typically results in relatively thick (micrometer-scale per coating) and occasionally uneven films, necessitating multiple deposition cycles and careful optimization to achieve fully dense, crack-free electrolytes.

Inkjet printing, another promising solution-based approach, involves the precise deposition of ceramic precursor inks to form uniform electrolyte films without the need for vacuum equipment. Kim et al.^489^ demonstrated the deposition of approximately 1.2 μm-thick BZCYYb2611 films on Ni-based anode substrates via inkjet printing, reporting exceptional performance with a PPD of 577 mW cm^–2^ at 500 °C and a notably low R_o_ of 0.072 Ω cm^2^. Inkjet printing enables precise control over film thickness, minimal material wastage, and maskless patterning. However, optimizing ink composition and addressing challenges such as “coffee-ring” effects and cracking during drying are critical. Utilizing sol–gel-based inks allows crystallization and densification at significantly reduced temperatures (700–1000 °C), as demonstrated by Schneller et al.^490^ for BZY10 electrolyte layers. The successful integration of inkjet printing into proton-conducting electrolytes underscores its suitability for fabricating submicron layers directly onto electrode supports.

Recently, 3D printing has emerged as a versatile approach for fabricating protonic ceramic electrolytes and cell components, often through extrusion of viscous ceramic pastes combined with *in situ* laser-assisted processing. Zou et al.^491^ developed a tubular P-RSOC via microextrusion-based 3D printing, simultaneously forming the porous anode support, a dense ∼ 12 μm-thick BaCe_0.2_Zr_0.7_Y_0.1_O_3−δ_ electrolyte layer, and the cathode. This 3D-printed tubular cell exhibited impressive power generation capabilities, delivering 2.45 W over a 12.5 cm^2^ active area and demonstrating stability over 200 h of operation at 650 °C. Similarly, Mu et al.^492^ employed laser-assisted 3D printing, integrating microextrusion and precise laser sintering/drying, offering geometric flexibility and simplified multilayer fabrication. While 3D printing provides exceptional geometric versatility and the potential for integrated cell architectures, challenges include equipment specialization, precise control of sintering shrinkage, and porosity management. Nonetheless, these successful demonstrations highlight the substantial potential of 3D printing for scalable manufacturing of proton-conducting electrolytes for RSOCs.

### 5.8 Scalability for Large-Scale Manufacturing

Scaling up P-RSOCs from laboratory prototypes to industrial-scale stacks is critical for commercial viability. To date, most advanced P-RSOC technologies have only been demonstrated at laboratory scale, whereas conventional YSZ-based oxygen-ion SOCs are already commercialized.^162^ This maturity gap underscores the unique scalability challenges of P-RSOCs, which must deliver high electrochemical performance consistently across many cells while meeting strict fabrication quality, cost, and durability targets. In practice, achieving these goals requires addressing several engineering trade-offs in fabrication processes, materials selection, and cell/stack design. For example, protonic ceramic electrolytes (e.g., doped BaCeO_3−δ_–BaZrO_3−δ_) often demand extremely high sintering temperatures (>1600 °C) or sintering aids to densify, which complicates manufacturing and can compromise material properties. By contrast, oxide-ion conductors like YSZ have well-established processing routes (tape casting, cosintering, etc.), though even they face challenges making thin, defect-free electrolytes at scale.

Some of the advanced fabrication methods used in research (as discussed in Sections 5.4–5.5) do not readily translate to high-volume production of large-area cells. Even using traditional fabrication methods (Section 5.1–5.3), as cell size increases, maintaining a thin, dense, crack-free electrolyte over a large area becomes challenging. Additionally, high-performance lab cells typically use small active areas and simple geometries, often involving many sequential steps (e.g., tape casting a support, slurry coating, screen-printing layers, cofiring, sealing, stacking). Scaling such processes uniformly is difficult, where any slight heterogeneity in tape thickness, binder distribution, or sintering profile can cause warping or cracking during firing, leading to yield loss.^500^ Thus, manufacturing technology has become a crucial performance factor: thinner electrolytes lower the R_o_ but demand exceptionally uniform fabrication to avoid flaws.

#### 5.8.1 Reproducibility and Quality Control

Large-scale cell fabrication demands strict control over material uniformity, microstructure, and interfacial bonding than is required for laboratory-scale button cells. Subtle defects (e.g., pinholes, local thickness variations, or incipient delamination) that may be tolerable in a 1–2 cm^2^ button cell become unacceptable when replicated on tens of 100–200 cm^2^ industry-level cells. During scale-up, small deviations in slurry rheology, tape-casting gap and speed, powder particle-size distribution, screen-printing ink formulation, or furnace temperature uniformity can be amplified into systematic defects and significant cell-to-cell performance scatter. Stack assembly imposes further constraints, in which each cell must meet strict specifications in flatness, dimensional tolerances, electrolyte density, and edge integrity to ensure reliable sealing, gas-tightness, and uniform current collection. In a 30–80 cell stack, even a single electrolyte hairline crack or poorly bonded seal can lead to local gas leakage or electrical shorting, dramatically shortening overall stack lifetime.^501^

To manage these risks, industrial production increasingly relies on in-line, nondestructive quality control rather than ex situ sampling inspection. IR thermography, ultrasound, acoustic methods, and electrochemical diagnostics are being developed as rapid screening tools to detect cracks, delamination, and other subsurface defects in individual cells and substacks before final integration.^501^ Montanini et al.^502^ demonstrated real-time IR thermal imaging of SOFC cathodes during operation, resolving local hot spots and early stage delamination that correlate with performance degradation and thus illustrating the potential of thermal imaging as an in situ diagnostic and quality-control tool. El-Amiri et al.^503^ utilized pulsed IR thermography combined with three-dimensional finite-element modeling to planar SOFCs, showing that both crack location and depth can be identified from thermal response maps, enabling quantitative defect characterization at the cell level. On the stack-scale SOCs, Netzelmann et al.^504^ applied high-frequency ultrasound testing coupled with active thermography to detect bonding defects and cracks in full-size SOFC stack elements, demonstrating that multimodal NDT can be integrated into manufacturing workflows for large components.

#### 5.8.2 Cost Considerations

Scalability is tightly coupled to cost at the cell, stack, and system levels. For protonic SOCs, the cost structure differs from that of mature oxygen-ion SOCs because both the electrolyte chemistries and their supply chains are less developed. State-of-the-art proton-conducting perovskites such as BaCeO_3−δ_–BaZrO_3−δ_ solid solutions (e.g., BZCYYb4411) require high-purity Ba/Ce/Zr/Y/Yb precursors, multistep powder synthesis, and typically very high calcination and sintering temperatures to achieve full densification and high protonic conductivity.^505^ Less mature supply chains and smaller production volumes for P-RSOC materials mean they have not yet benefited from economies of scale enjoyed by YSZ (which is mass-produced for fuel cells, oxygen sensors, etc.). Recent techno-economic benchmarking indicates that, under current assumptions of materials cost and manufacturing volume, projected stack manufacturing costs for intermediate-temperature PCFCs remain several-fold higher than those for comparable SOFC stacks, primarily because of immature supply chains and lower manufacturing scale.^15^

At the stack and balance-of-plant levels, protonic SOCs can partially offset these higher materials costs. Their intermediate operating temperature allows the use of relatively low-cost ferritic stainless steels and commercial SOFC-grade alloys (e.g., 441-type or Crofer-type steels) as interconnects, rather than more expensive high-Cr or ceramic interconnects required at ∼ 800–900 °C. Corrosion studies on ferritic stainless steels show that such alloys combine acceptable oxidation behavior, good mechanical properties, and significantly lower raw-material cost than ceramic or high-Ni alternatives, while interconnects constitute a large fraction of total stack manufacturing cost.^506^ Additionally, lower operating temperatures also ease demands for glass-ceramic seals and insulation, potentially reducing sealing failure rates and balance-of-plant costs over the stack lifetime.

Manufacturing technique also heavily influences cost at scale. Many thin-film deposition techniques that deliver record cell performance in the laboratory (e.g., methods mentioned in Sections 5.4–5.5) remain too capital-intensive and low-throughput for mass production of large-area cells. In contrast, tape casting, screen printing, wet spraying, and cosintering are inherently suited to high-volume manufacturing and have already been industrialized for planar SOFCs. Large-area protonic cells based on BZCYYb-type electrolytes have now been fabricated by multilayer aqueous tape casting and cosintering, demonstrating that low-cost SOFC manufacturing platforms can be adapted to protonic chemistries while maintaining reasonable electrochemical performance.^507^ However, proton-conducting perovskites generally densify at higher peak temperatures or longer dwell times than YSZ, increasing firing cost and furnace wear. Advanced sintering techniques discussed in Section 5.6 can mitigate these challenges, but they may introduce additional complexities and reliability concerns when scaled to full-size cells and stacks.

#### 5.8.3 Long-Term Stability and Durability

The commercial viability of P-RSOCs ultimately hinges on achieving stack lifetimes and degradation rates comparable to, or better than, today’s oxide-ion SOC stacks. For stationary applications, target metrics are typically on the order of thousands of hours of continuous operation with area-specific degradation ≤ 0.5% per 1000 h, and total service lifetimes of 40000–50000 h (5–6 years of continuous operation). Long-term field and demonstration tests of oxide-ion SOC stacks have already achieved operating times up to and beyond 10000 h with average stack degradation rates below ∼ 1% per 1000 h, illustrating what is now considered an industrial benchmark for solid oxide technology.^508,509^ In contrast, most P-RSOC reports still focus on single cells or short stacks operated for hundreds to a few thousand hours. One breakthrough from Duan et al.^510^ demonstrated highly durable P-RSOCs operated for >6000 h on a wide range of practical fuels (H_2_, natural gas, hydrocarbons, and ammonia) with degradation rates generally <1.5% per 1000 h, highlighting the promise of protonic technology but also underscoring the remaining gap relative to commercial P-RSOCs targets.

Materials-related degradation, particularly chemical stability in realistic operating atmospheres, remains a primary concern in scaling up P-RSOCs. As discussed in Section 2.1, many BaCeO_3−δ_-rich proton-conducting perovskites are vulnerable to BaCO_3_ formation and decomposition under CO_2_- and H_2_O-containing gases at intermediate temperatures. In laboratory single-cell tests, this is often mitigated by using high-purity fuels and a deionized water source. However, in practical systems using reformate fuels or ambient air, minor contaminants (CO_2_, hydrocarbons, sulfur species, and mineral impurities in water) can progressively decompose the electrolyte or electrode surface, forming resistive secondary phases over large areas. As discussed in chapter 2.1, a clear trade-off persists, where Ce-rich compositions tend to offer higher proton conductivity but are more susceptible to carbonation and hydration, whereas Zr-rich compositions are chemically more stable but more difficult to densify and often exhibit lower conductivity at operation temperature. Rational electrolyte design for durable large-area P-RSOCs must therefore balance conductivity, sinterability, and long-term chemical stability against realistic fuel/air compositions.

Mechanical durability is an equally critical constraint during scale-up. High-performance protonic cells typically employ ultrathin (<10 μm) electrolytes supported on porous electrodes or supports. While this architecture minimizes ohmic losses, it also increases susceptibility to cracking and delamination under handling, thermal cycling, and stack clamping stresses. Larger planar cells with unsupported spans are especially vulnerable to thermomechanical gradients, much as observed in large-area SOFCs, where failure probability increases with cell size and thermal cycling severity. To mitigate these issues, several more robust architectures are being explored. One route is metal–supported protonic cells, in which a porous Ni or ferritic-steel support provides mechanical robustness and tolerance to thermal shock, with a thin proton-conducting ceramic membrane and functional electrodes deposited on top.^511^ Another strategy is using thick anode-supported designs (similar to some SOFCs) where a robust porous electrode (e.g., Ni–BZCYYb cermet) lends strength, at the expense of some proton transport distance. This can reduce the risk of cell fracture during stack assembly and operation.

## 6 Challenges and Prospects

Despite significant progress in the development and optimization of proton-conducting electrolytes for SOCs, several critical challenges remain that hinder their widespread commercialization and practical deployment. Overcoming these barriers requires targeted research focused on enhancing proton conductivity at lower operating temperatures, reducing electronic leakage, and improving the chemical stability under realistic working conditions. This section critically examines the current limitations and outlines the prospective strategies and future research directions.

### 6.1 Challenges

#### 6.1.1 Enhancing Proton Conductivity at Temperatures below 400 °C

One of the most pressing challenges facing proton-conducting solid oxide electrolytes is the sharp decline in ionic conductivity at temperatures below 400 °C—a regime increasingly targeted for practical applications such as distributed energy generation, electrolysis, and portable fuel cell systems. Operating at lower temperatures not only reduces material degradation—thereby extending device lifetimes—but also enables the use of cost-effective sealing, current collectors, and interconnect materials that would otherwise be vulnerable to degradation at conventional high temperatures (>600 °C). Additionally, lower-temperature operation improves thermal cycling durability and mitigates mechanical stresses caused by thermal expansion mismatches among cell components.

Figure 34 summarizes the conductivity of the proton-conducting electrolytes discussed in this paper across different temperature ranges.^37,38,45–47,49,207,216,229,233,512,513^ Notably, ceria-carbonate electrolytes and semiconductor-based electrolytes exhibit significantly higher conductivities at a similar temperature level. Constrained by testing methodologies, conductivity characterizations of such electrolytes in most reported articles are typically conducted using single-cell configurations, wherein the highly conductive electrode materials introduce non-negligible electronic conduction contributions to the measured results. Currently, perovskite-based oxide materials remain the dominant choice for proton-conducting electrolytes. Since the intrinsic proton conduction mechanism in perovskite-based oxides follows Arrhenius-type temperature dependence, both proton mobility and hydration reaction rates decline sharply with falling temperature, leading to an exponential drop in conductivity. This behavior arises because proton migration relies on thermally activated hopping between oxygen lattice sites, while hydration reactions—essential for generating mobile protons—become increasingly sluggish at lower temperatures. For example, even the benchmark electrolyte BZCYYb1711 typically exhibits proton conductivities on the order of 10^–3^ S cm^–1^ at 400 °C—well below the ideal threshold (>10^–2^ S cm^–1^) required for high-efficiency device performance.^514^

**Figure 34:**
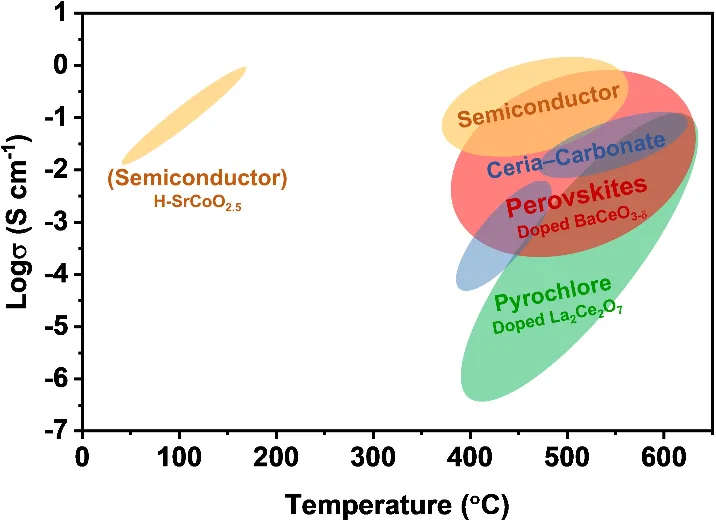
Summary of conductivity for various advanced proton-conducting electrolytes.^37,38,45–47,49,207,216,229,233,512,513^ . Note: only materials demonstrated in functional full cells are included.

To overcome this limitation, several strategies have been explored to boost proton transport at reduced temperatures. One approach involves doping with smaller trivalent cations, such as Yb^3+^ or Sc^3+^, which introduce beneficial lattice distortions and oxygen vacancies while minimizing proton trapping. These smaller dopants effectively “soften” the lattice, lowering the migration energy barriers for proton hopping and improving overall mobility. However, the incremental gains from simple dopant substitution are generally insufficient to meet the stringent conductivity requirements for operation below 400 °C. Moreover, excessive doping can induce defect clustering, cation ordering, and lattice hardening effects that paradoxically impair long-range proton diffusion and hydration, underscoring the need for careful optimization of dopant levels.^515^

Tailoring hydration thermodynamics through defect engineering has emerged as an important strategy. Ideally, defect chemistry should promote the formation of stable oxygen vacancies and strong proton solvation without compromising the electrolyte’s mechanical or chemical stability. Materials with lower hydration enthalpies and higher hydration entropies are especially desirable as they enable efficient proton uptake at reduced temperatures. Achieving this balance requires precise control over dopant selection, oxygen partial pressure during synthesis, and processing temperature to stabilize favorable defect populations.

Beyond bulk approaches, recent advances in thin-film fabrication and nanostructuring have opened new avenues for enhancing low-temperature proton conductivity. Epitaxial strain engineering, for example, has been employed to stabilize high-symmetry cubic or pseudocubic phases at lower temperatures—phases that inherently exhibit more isotropic and lower-barrier proton conduction pathways.^516^

Nanoscale engineering of grain boundaries and interfaces also holds significant promise. Introducing coherent grain boundaries, suppressing secondary phase formation, and creating space-charge regions that preferentially promote proton transport have all been shown to enhance low-temperature performance.^287^ Furthermore, incorporating nanoheterostructures and multilayer architectures with tailored local strain and defect chemistry can amplify hydration kinetics and mitigate defect associations that limit proton mobility in conventional bulk ceramics.

Draber et al.^515^ demonstrated that in Y-doped BaZrO_3−δ_, proton conductivity is governed by nanoscale percolation pathways formed through overlapping dopant-induced trapping zones. At low dopant concentrations, protons are strongly trapped near acceptors, leading to reduced mobility. However, beyond a critical dopant threshold, these trapping zones overlap, creating interconnected low-barrier pathways that facilitate long-range proton transport. Their DFT and kinetic Monte Carlo simulations quantitatively reproduced the experimental conductivity trends and revealed that ordered dopant superstructures—particularly in 1D and 3D configurations—further enhance conductivity by aligning favorable ion jump pathways and suppressing high-barrier transitions. This work establishes percolation and dopant arrangement as key principles for optimizing proton transport in perovskite electrolytes.^515^ In addition, Kasamatsu et al.^517^ used large-scale first-principles thermodynamic simulations to show that at realistic doping levels, many-body interactions lead to dopant clustering and the emergence of extended networks. These networks enable percolative proton transport, while certain configurations increase trapping. Their findings underscore the importance of optimizing dopant topology to balance percolation and trap avoidance for improved conductivity. Experimentally, Ngabonziza et al.^518^ demonstrated that 2D doping of BaZrO_3−δ_ with Y^3+^ in epitaxial superlattices enhances proton conductivity by promoting percolation pathways along dopant planes and reducing proton trapping. Conductivity increased by up to 2 orders of magnitude as the BaZrO_3−δ_ layer thickness decreased, confirming the critical role of dopant arrangement in facilitating ion transport.

Despite these promising advances, significant challenges remain in translating thin-film and nanostructure-enabled improvements to practical, large-area manufacturing. Techniques such as PLD and molecular beam epitaxy offer excellent microstructural control but are inherently costly, slow, and difficult to scale. Scalable alternatives—such as chemical solution deposition and aerosol deposition—are under active investigation but require further optimization to achieve the necessary film quality, phase purity, and mechanical robustness.

Importantly, efforts to improve low-temperature proton conductivity must avoid compromising other critical material properties, including chemical stability against CO_2_- and H_2_O-rich atmospheres, mechanical robustness, and compatibility with other cell components, such as electrodes. As such, the next generation of proton-conducting electrolytes will likely require integrated solutions that combine optimized doping strategies, tailored defect chemistry, nanoscale structural control, and scalable processing methods to simultaneously meet the demands of high conductivity, long-term durability, and manufacturability.^519^

Given the limited performance improvements attainable through existing perovskite-based chemistries, there is an urgent need to discover entirely new classes of proton-conducting materials that intrinsically exhibit high conductivity at temperatures below 400 °C. These materials must offer fast proton transport without relying heavily on thermal activation, and simultaneously demonstrate chemical and mechanical resilience under operational conditions. High-throughput computational screening, machine learning-guided discovery, and rapid experimental validation pipelines will be critical to identifying and developing such next-generation systems.

In summary, while enhancing proton conductivity below 400 °C remains a formidable challenge, it is a crucial enabler for the wider commercialization and application of protonic ceramic devices. Continued interdisciplinary efforts—combining materials design, advanced processing, mechanistic modeling, and device integration—will be essential to achieving the breakthroughs required for the next generation of efficient, durable, and scalable low-temperature proton-conducting technologies.

#### 6.1.2 Blocking Electronic Leakage

Electronic leakage currents represent a major challenge in proton-conducting electrolytes, especially under oxidizing conditions and at elevated operational temperatures (>600 °C). In an ideal proton-conducting material, ionic transport—specifically proton conduction—should dominate overwhelmingly, with minimal electronic conductivity to ensure high energy efficiency. However, under high-temperature oxidizing environments, parasitic electronic conduction becomes increasingly pronounced.^520^

Electronic conductivity in these materials primarily arises from partial oxidation of the oxide, generating electron–hole (p-type) conduction. This mechanism leads to unwanted mixed ionic-electronic conduction, which can significantly reduce device efficiency and cause additional issues such as electrode polarization, interfacial degradation, and accelerated long-term performance loss.^521^

The impact of electronic leakage is particularly severe under electrolysis conditions, where a high t_i_ is essential to maintain high FE. Any appreciable electronic conductivity diminishes the fraction of the applied current contributing to water splitting or CO_2_ reduction, lowering system efficiency and increasing parasitic energy losses. Moreover, electronic carriers can promote deleterious side reactions at the electrolyte–electrode interfaces, causing microstructural instabilities, chemical incompatibilities, and premature device failure.

An alternative and increasingly attractive strategy involves introducing an electron-blocking layer at the electrolyte surface or the electrolyte/electrode interface. As Figure 35a illustrates, the thin, electronically insulating, yet ionically conductive layer effectively suppresses electronic leakage by blocking the transport of free electrons and holes across the electrolyte, thereby maintaining a high t_i_. By acting as a physical barrier to electronic conduction, electron-blocking layers minimize parasitic electronic currents, enhance overall cell efficiency, and help preserve the chemical and electrochemical stabilities of the system under both fuel cell and electrolysis conditions.^88^

**Figure 35:**
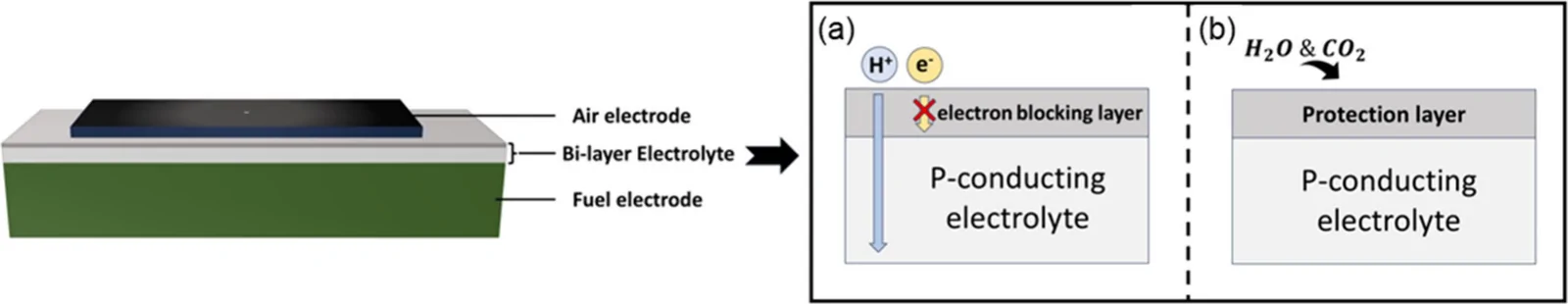
Schematic illustrations of bilayer electrolyte architectures designed for enhanced proton-conducting solid oxide cells: (a) a bilayer featuring an electron-blocking layer to suppress electronic leakage and improve FE; (b) a bilayer incorporating a protective layer to mitigate degradation from contaminants such as CO_2_ and H_2_O, thereby improving chemical stability and operational durability.

Despite these promising advances, several critical challenges remain. Precise control over dopant distribution and concentration is essential, as inhomogeneous dopant segregation can introduce localized electronic pathways or promote secondary phase formation that undermine electrolyte performance. Additionally, the complex interplay between defect chemistry, local lattice distortions, and thermodynamic stability must be carefully balanced to avoid adverse effects, such as increased R_gb_ or reduced hydration capacity.

Beyond material design, operational strategies can also contribute to minimizing electronic leakage. Lowering operational temperatures, optimizing gas compositions (for example, maintaining reducing atmospheres on the fuel side), and tailoring device architectures to minimize oxygen chemical potential gradients across the electrolyte are effective complementary measures. Furthermore, achieving a deeper mechanistic understanding of electronic leakage pathways remains a crucial step toward advancing these materials for practical, high-efficiency electrochemical applications.

In summary, suppressing electronic leakage remains a critical requirement for realizing the full potential of proton-conducting solid oxide electrolytes. Achieving this goal will require an integrated approach combining intelligent dopant design, advanced processing, precise defect engineering, and system-level operational optimization. Continued fundamental studies of defect interactions, redox stability, and electronic transport mechanisms will be essential to guide the development of next-generation electrolytes with minimal electronic conductivity under practical conditions.

#### 6.1.3 Improving Chemical Stability against High Concentrations of H_2_O and/or CO_2_

The chemical stability of proton-conducting electrolytes—particularly under H_2_O- or CO_2_-rich atmospheres —is a fundamental property critical to the long-term durability and practical viability of protonic ceramic devices. Among the widely studied perovskite-based electrolytes, BaCeO_3−δ_-derived materials have garnered significant attention for their excellent proton conductivity. However, these cerate-based systems are thermodynamically unstable in steam- and CO_2_-containing environments at intermediate temperatures, which are typical operating conditions for fuel cells and electrolysis devices.

Under CO_2_-rich conditions, BaCeO_3−δ_ readily decomposes, forming BaCO_3_ and CeO_2_ as secondary phases. These reactions lead to the formation of insulating and nonproton-conducting products that not only degrade the ionic conductivity but also compromise the mechanical integrity and structural cohesion of the electrolyte. Likewise, under humidified atmospheres, barium hydroxide Ba­(OH)_2_ can form. This reaction not only depletes the active electrolyte phase but can also result in swelling, microcracking, and delamination in multilayer cell structures. Both degradation mechanisms preferentially initiate at grain boundaries, surfaces, and interfaces, making them particularly detrimental to long-term stability.^522^

Recent studies have explored the substitution of more chemically robust tetravalent cations—particularly Hf^4+^ and Sn^4+^—into the perovskite B-site lattice. For instance, BHCYYb electrolytes have demonstrated substantial resistance to both CO_2_ and H_2_O degradation. The enhanced performance is primarily attributed to the strong Hf–O bond, which raises the free energy barrier for carbonate and hydroxide formation and thereby stabilizes the perovskite phase under harsh operational conditions. Such modifications not only suppress decomposition reactions but also preserve grain boundary cohesion and inhibit dopant migration that could otherwise result in the formation of reactive secondary phases.^21^

Beyond compositional tuning, materials development has also focused on structural innovations—such as bilayer electrolytes—to address the trade-off between conductivity and chemical stability (Figure 35b). As mentioned in Section 3.3, compositions like BZCYYb1711 offer excellent proton conductivity but remain vulnerable to degradation in CO_2_- and H_2_O-containing environments. In contrast, more chemically robust materials such as BHYb82 offer improved stability but suffer from lower conductivity. A bilayer design featuring an ultrathin (15–110 nm) BHYb82 protective coating applied onto a BZCYYb1711 backbone has been proposed. This architecture preserves the high proton conductivity of the BZCYYb1711 core while effectively protecting it from chemical attack, as the dense BHYb82 layer blocks CO_2_ and H_2_O ingress and suppresses surface reactions. Structural analysis confirmed coherent, low-resistance interfaces, highlighting the potential of bilayer designs to combine the best features of conductivity and stability in proton-conducting devices.^28^

Additionally, intergranular phase engineering—through the introduction of inert secondary phases or dopants that stabilize grain boundary regions—has been proposed to mitigate local phase instability. For instance, rare-earth dopants with low reactivity toward NiO can help maintain stable electrode–electrolyte interfaces, particularly during high-temperature cosintering in cell fabrication.

Despite recent advances, the widespread implementation of chemically stable proton-conducting electrolytes remains hindered by dopant solubility limits, synthesis reproducibility, and the lack of cost-effective, scalable processing methods. Moreover, simultaneously optimizing the ionic conductivity, mechanical integrity, and interfacial compatibility remains a nontrivial challenge.

Looking forward, integrated materials design approaches—combining compositional optimization, microstructure control, and surface/interface engineering—will be essential for developing durable electrolytes that can meet the chemical and electrochemical demands of real-world operation. High-throughput computational screening and *in situ/operando* characterization methods will play pivotal roles in accelerating the discovery of stable new compositions and unraveling degradation mechanisms at the atomic scale. Furthermore, scalable fabrication techniques capable of producing dense, stable, and defect-free bilayer structures will be critical for translating laboratory-scale innovations into commercially viable protonic ceramic devices.

In conclusion, improving the chemical stability against CO_2_ and H_2_O is indispensable for the long-term operation of P-RSOCs. Continued research efforts must focus on both fundamental material understanding and practical processing strategies to enable robust, high-performance, and commercially scalable proton-conducting electrolytes.

### 6.2 Prospects

To overcome the challenges outlined in the previous sections and fully unlock the potential of proton-conducting electrolytes for intermediate to low-temperature SOCs, the field must adopt innovative research strategies and embrace interdisciplinary methodologies. Future progress will hinge on accelerating materials discovery, deepening the fundamental understanding of structure–property relationships, and leveraging successful approaches from adjacent electrolyte systems such as lithium- and sodium-ion conductors.

#### 6.2.1 Accelerating the Discovery of Novel Proton-Conducting Materials

Historically, the discovery and optimization of proton-conducting oxide electrolytes have relied heavily on iterative experimental efforts, a process that is time-consuming, resource intensive, and inherently slow. This traditional approach has constrained the pace at which promising new materials can be identified, refined, and translated to practical applications. In recent years, however, the integration of high-throughput computational screening and machine learning has emerged as a transformative alternative, offering the potential to dramatically accelerate materials discovery while reducing the reliance on costly trial-and-error experimentation.^523^

Advances in computational materials science—particularly DFT and MD simulations—have significantly improved the predictive capabilities for key material properties of proton-conducting electrolytes. Properties such as proton migration barriers, defect formation energies, hydration thermodynamics, and chemical stability under operational conditions can now be estimated with increasing accuracy. As a result, large-scale computational databases populated with these data have enabled the rapid screening of thousands of candidate compositions. By applying selection criteria based on computed descriptors, researchers can efficiently narrow the chemical space to a focused set of highly promising candidates for experimental validation.

Recent studies have showcased the power of this approach. For example, Luo et al.^135^ employed a high-throughput DFT screening framework to investigate multication perovskite oxides, successfully identifying compositions with low proton migration energies and enhanced chemical stability in CO_2_-rich environments. In this study, a systematic, computation-driven approach was employed to accelerate the discovery of next-generation proton-conducting materials. A bulk perovskite crystal structure was used as the reference model to assess the suitability of pristine BaCeO_3−δ_, BaZrO_3−δ_, BaHfO_3−δ_, and BaSnO_3−δ_ as doping hosts. By incorporating 29 distinct elements at varying doping levels, a comprehensive screening library of 932 unique perovskite compositions was generated. For each material, four key descriptors were evaluated using high-throughput DFT calculations: oxygen vacancy formation energy, hydration energy, H_2_O adsorption energy, and CO_2_ adsorption energy.

This computational framework led to the discovery of BSCYb as a highly promising new family of proton-conducting electrolytes, offering potential improvements in hydration, proton transport, and chemical stability compared to the widely studied BZCYYb series. Experimental validation confirmed the computational predictions: BSCYb172 demonstrated superior conductivity at reduced temperatures (400–500 °C) compared to benchmark BZCYYb1711. Overall, this work not only introduces a new class of high-performance proton-conducting electrolytes but also showcases the power of predictive, high-throughput computational screening combined with experimental validation. This integrated methodology represents a significant step forward in the rapid discovery and development of advanced materials for protonic ceramic electrochemical cells, paving the way for future breakthroughs in the field.

Beyond computational screening, the integration of machine learning with computational and experimental data sets has introduced new dimensions of predictive capability. Machine learning models—including neural networks, support vector machines, and random forests—have been applied to reveal complex, nonlinear relationships between material descriptors (such as ionic radii, electronegativity, lattice parameters, and bond strengths) and key performance metrics like proton conductivity, chemical resilience, and mechanical stability. By training on limited but curated data sets, these models can rapidly predict the properties of unexplored compositions, reducing the experimental workload while preserving predictive reliability.

A notable example comes from the work of Hyodo et al.,^524^ who demonstrated the effectiveness of machine learning in accelerating materials discovery by constructing a robust structure–property map for hydration behavior. Their study utilized thermogravimetric data from 65 perovskite compositions to train a model capable of predicting proton concentration across thousands of hypothetical doped oxides. Notably, the model identified SrSn_0.8_Sc_0.2_O_3−δ_ as a new proton conductor with desirable hydration thermodynamics and verified proton conduction, despite this composition not previously being recognized for such functionality. This study emphasizes the importance of incorporating physicochemical fundamentals—such as dopant concentration, hydration enthalpy, and entropy—into the machine learning process to reliably extrapolate properties in unexplored compositional spaces.

In another study, Fujii et al.^523^ introduced a sophisticated computational approach utilizing defect-chemistry-trained, interpretable machine learning to systematically discover novel proton-conducting inorganic solid electrolytes. While conventional proton conductor research has predominantly emphasized perovskite structures due to their adaptable coordination frameworks that facilitate defect manipulation, this study uniquely targets unconventional, nonperovskite inorganic solids. As shown in Figure 36, by explicitly incorporating critical defect chemistry features—specifically dopant dissolution energies, hydration energies, and proton migration barriers—into predictive models, the authors significantly enhance both predictive capability and interpretability. Using this methodology, two previously unexplored proton conductors were experimentally identified: Pb-doped Bi_12_SiO_20_ with a sillenite structure and Sr-doped Bi_4_Ge_3_O_12_ exhibiting an eulytite structure. Notably, Pb-doped Bi_12_SiO_20_ represents the first proton conductor composed exclusively of groups 14 and 15 cations. It features a unique sillenite lattice that enables rapid, three-dimensional proton conduction through a loosely bonded BiO_5_ polyhedral network. The experimentally validated proton conductivity of this novel material is competitive with state-of-the-art perovskite-based electrolytes, showing particularly low activation energies for proton transport. Overall, this study underscores the powerful synergy of high-throughput computational screening, defect chemistry considerations, and interpretable machine learning in accelerating the discovery of unconventional proton conductors, thereby substantially broadening the material landscape available for advanced solid oxide electrochemical applications.

**Figure 36:**
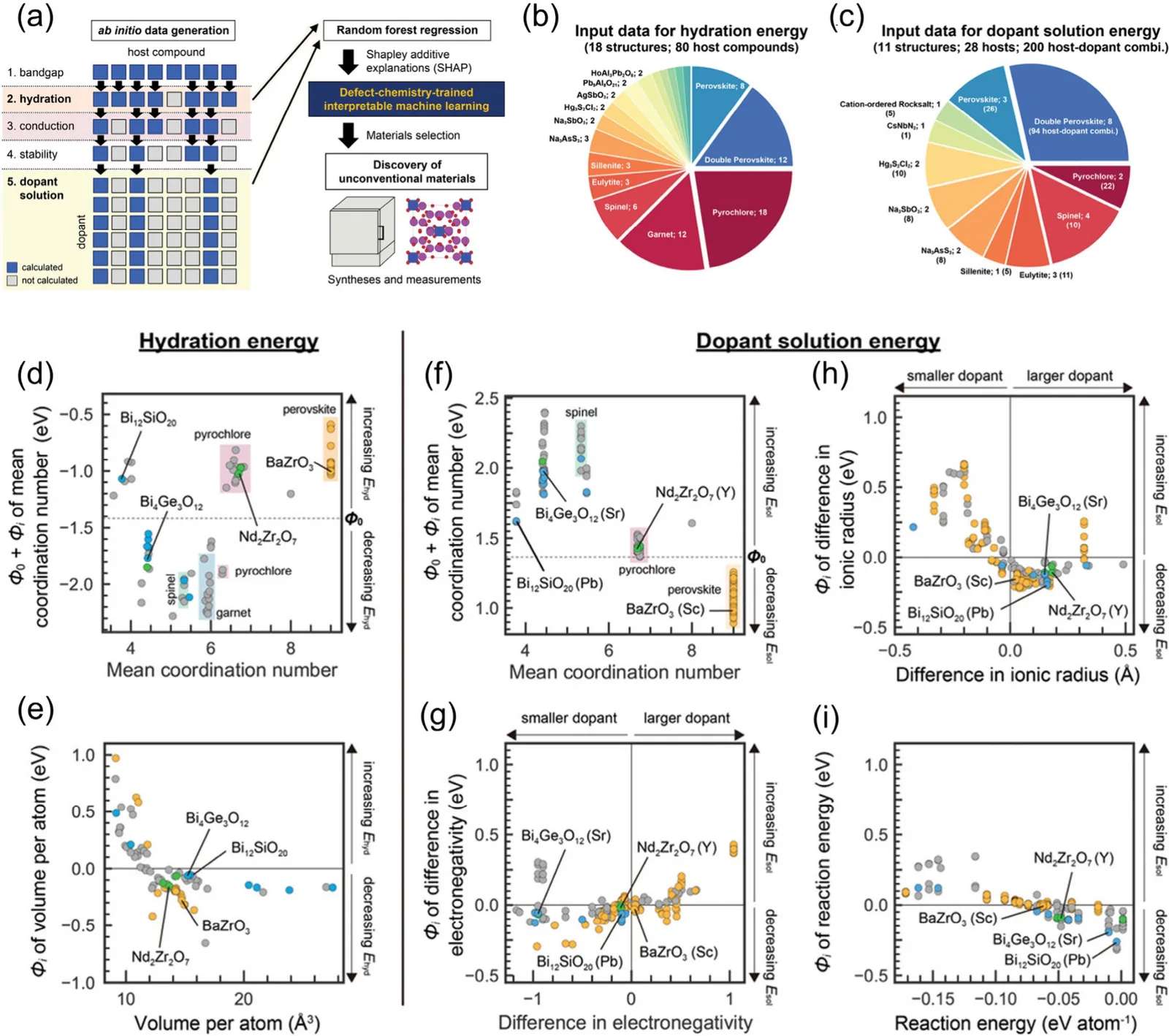
(a) Schematic workflow illustrating the exploration and identification of unconventional proton-conducting oxides. The procedure involves high-throughput ab initio calculations for generating hydration and dopant solution energy data, constructing interpretable defect-chemistry-trained machine learning models, and performing proof-of-concept synthesis and experimental validation. (b,c) Distributions of ab initio-calculated hydration and dopant solution energies used as input data sets for machine learning models. (d–i) Influence of structural and chemical descriptors on predicted hydration and dopant solution energies: (d,f) average coordination number of host cations; (e) volume per atom; (g) difference in ionic radius and (h) difference in electronegativity between dopant and host cations; and (i) reaction energy of the host compound relative to neighboring equilibrium phases (inverse distance to convex hull). Different oxide classes are highlighted using color coding: perovskites (yellow), previously reported proton-conducting nonperovskites (green), newly identified promising nonperovskites (blue), and other nonperovskite compounds (gray). The eight promising, yet-unreported nonperovskite candidates identified include Pb-doped Bi_12_SiO_20_ (sillenite), Ti-doped Li_3_NbO_4_ (ordered rocksalt), Sr-doped Bi_4_Ge_3_O_12_ (eulytite), Pb-doped Cs_3_BiO_3_, Pb-doped Rb_3_BiO_3_, Li-doped K_2_Sn_2_O_3_, Li-doped K_2_Pb_2_O_3_, and Mg-doped ZnGa_2_O_4_ (spinel). Average baseline energies (Φ_0_) for hydration and dopant solution energies are −1.42 and 1.36 eV, respectively. Pyrochlores comprising trivalent and tetravalent cations are clustered toward higher hydration energies (top of panel b), whereas those composed of divalent and pentavalent cations exhibit lower hydration energies (bottom of panel b). Reproduced from ref 523. Available under a CC-BY 4.0 license. Copyright 2023 Wiley Susumu Fujii et al.

Despite this progress, several challenges must be addressed to fully harness the computational and machine learning approaches. The quality, consistency, and completeness of input data strongly influence the ML model performance. Experimental data sets are often sparse, noisy, or inconsistently reported, while computational data are limited by the inherent approximations of first-principles methods. Overcoming these limitations will require robust data curation, uncertainty quantification frameworks, and active learning strategies that prioritize the acquisition of high impact, informative data points.

Another important challenge lies in improving the interpretability of the machine learning models. While complex models such as deep neural networks can deliver impressive predictive power, they often lack transparency, making it difficult to extract mechanistic insights that could inform rational material design. To address this, researchers are increasingly adopting interpretable machine learning approaches, including feature importance analysis, model distillation, and the development of physically informed models that embed known thermodynamic or kinetic relationships.

A crucial yet often underappreciated gap remains between theoretical predictions and experimental realization. Many predicted materials are thermodynamically metastable under typical processing conditions, such as sintering at high temperatures (>1400 °C), which is often required to achieve phase purity and densification. Issues such as dopant volatilization, phase segregation, and secondary phase formation frequently impede the successful synthesis of theoretically promising candidates. To help bridge this gap, fabrication-aware filters—such as thermodynamic stability analyses, convex hull calculations, and phase diagram evaluations under relevant gas environments—are increasingly being integrated into computational screening workflows. These filters help ensure that candidate materials are not only theoretically high-performing but also practically synthesizable.

In parallel, advances in processing techniques—such as ultrafast joule heating sintering, SPS, cold sintering, aerosol deposition, and field-assisted processing methods—offer promising routes to stabilize metastable compositions at lower temperatures, thereby expanding the range of computationally predicted materials that are experimentally accessible.^308,525^

In summary, the integration of high-throughput computational screening, machine learning, and fabrication-aware design holds enormous promise for revolutionizing the discovery of proton-conducting electrolytes. Realizing this potential will require a holistic approach that combines accurate computational modeling, robust machine-learning methodologies, systematic experimental validation, and innovative materials processing techniques. Strengthening the feedback loop between computational prediction and experimental realization will be critical to accelerating the development of next-generation proton-conducting materials that meet the stringent performance, stability, and manufacturability requirements of advanced SOC devices.

#### 6.2.2 Unraveling the Inherent Structure–Conductivity and Structure–Stability Relationships

A deeper fundamental understanding of the intrinsic structure–property relationships in proton-conducting electrolytes is essential for the rational design, optimization, and eventual commercialization of next-generation materials. The performance of proton-conducting oxides is determined by a complex interplay among the crystal structure, defect chemistry, proton transport mechanisms, and chemical and mechanical stability under operational conditions. These interdependent factors require systematic investigation through a combination of advanced experimental characterization and state-of-the-art theoretical and computational modeling.

Recent advances in experimental techniques have opened unprecedented opportunities to probe local structures, defect states, and proton dynamics on atomic and molecular scales. Methods such as neutron scattering, solid-state NMR, *in situ* TEM, synchrotron-enabled scattering, and XAS have been particularly effective at providing mechanistic insights. Neutron scattering, because of the high sensitivity of neutrons to hydrogen atoms, has emerged as a premier tool for studying proton distributions and motions in complex oxide lattices.^317,526,527^

Braun et al.^528^ present the first direct experimental evidence for a proton polaron in hydrated metal oxide proton conductors, specifically BCY20, through the use of advanced characterization techniques. QENS was employed to resolve proton jump dynamics on the picosecond time scale, providing direct measurements of diffusion coefficients and jump times as a function of temperature and applied pressure (Figure 37a–e).^528^ The QENS spectra were analyzed using the Chudley–Elliott model, enabling a quantitative description of proton motion and its evolution under varying thermodynamic conditions. To complement the QENS results and probe long-range ion transport, high-pressure EIS was conducted using a custom-designed cell capable of sustaining up to 1.25 GPa (Figure 37f). This allowed the authors to correlate changes in proton conductivity and activation energy with lattice strain. The combination of QENS and EIS revealed a consistent increase in activation energy and suppression of proton mobility under pressure, directly linking lattice compression to transport limitations. These techniques also enabled the identification of the Ce–O stretching phonon mode as the dominant vibrational driver of proton transport.^528^ By analyzing the pressure dependence of Raman-active phonon modes and jump kinetics, the study demonstrated that proton motion is modulated by lattice dynamics and follows the thermally activated hopping behavior predicted by Holstein’s small polaron theory. The integrated use of QENS and pressure-tunable EIS thus provided an unprecedented view into proton–phonon coupling and confirmed the polaronic nature of proton conduction in ceramic oxides.

**Figure 37:**
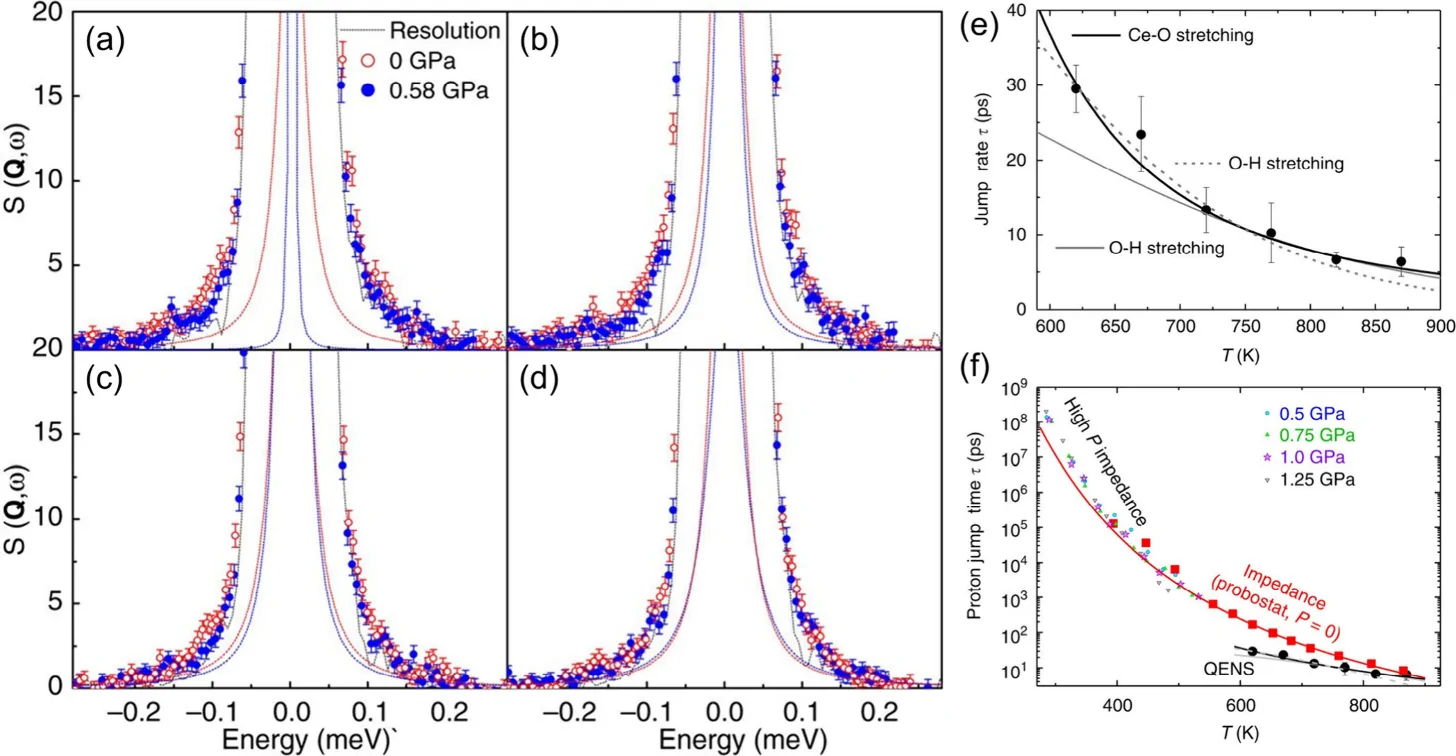
QENS spectra of hydrated BCY20 measured in a high-pressure INCONEL cell at 0 GPa (red open symbols) and 0.58 GPa (blue filled symbols) at a momentum transfer of Q = 0.6 Å^–1^ for (a) 720 K and (b) 770 K, and Q = 0.8 Å^–1^ for (c) 720 K and (d) 770 K. Solid lines represent least-squares Gaussian fits to account for elastic contributions. (e) Proton jump times τ extracted from QENS data as a function of temperature, with fitted curves based on Samgin’s polaron model using the Ce–O stretching mode (355 cm^–1^, solid black) and the O–H stretching mode (3560 cm^–1^, solid and dotted gray). (f) Proton jump times derived from EIS under varying applied pressures, fitted with the polaron model using the Ce–O stretching mode. Reproduced from ref 528. Available under a CC-BY 4.0 license. Copyright 2017 Springer Nature Artur Braun et al.

*In situ* and *operando* TEM studies have further advanced our understanding by enabling direct visualization of structural evolution, phase transformations, and defect interactions under realistic operational conditions. These techniques have been instrumental in uncovering dynamic processes such as grain-boundary reactions, secondary phase formation, and lattice distortions triggered by hydration or CO_2_ exposure—phenomena central to the degradation mechanisms that limit electrolyte durability. Likewise, synchrotron-based techniques such as X-ray absorption near-edge structure and extended X-ray absorption fine structure spectroscopy have proven valuable for probing cation oxidation states, local coordination environments, and defect clustering under working conditions.

On the computational front, first-principles calculations—including DFT and MD simulations—have played a pivotal role in mapping proton migration pathways, elucidating defect interactions, and predicting lattice responses to doping, strain, and environmental changes.^529–531^ These insights are critical for guiding rational dopant selection, defect engineering, and microstructure design.

The synergistic integration of experimental characterization and computational modeling is increasingly recognized as indispensable for unraveling the complex relationships among structure, properties, and performance in proton-conducting materials. Coupling empirical observations with theoretical predictions not only enables the validation of computational models but also refines experimental interpretation, creating an iterative feedback loop that enhances the reliability of both approaches. This integrated framework supports the predictive, mechanism-based design of novel electrolytes with tailored proton-transport properties and improved long-term stability.

Looking ahead, continued progress in *operando* multimodal characterization, multiscale modeling, and machine-learning-guided analysis of structure–property relationships is expected to play a transformative role in advancing the field. These integrated efforts will be key to bridging the gap between fundamental scientific understanding and technological implementation, accelerating the development of durable, high-performance protonic ceramic devices for a wide range of energy-conversion applications.

#### 6.2.3 Drawing on the Development Approach of Lithium (Sodium)-Ion Electrolyte Materials

The tremendous progress made in the discovery and development of lithium-ion and sodium-ion electrolyte materials offers valuable lessons and methodologies that can be directly leveraged to advance proton-conducting solid oxide electrolytes.^532^ Over the past several decades, the systematic exploration of Li-ion and Na-ion conductors—driven by the synergistic combination of high-throughput computational screening, machine learning-aided property prediction, advanced structural characterization, and iterative experimental validation—has led to the identification of electrolytes with exceptionally high ionic conductivity, electrochemical stability, and processability.^533^ This integrated and highly effective research model provides a powerful blueprint for accelerating similar breakthroughs in the field of proton conductors.

In lithium-ion electrolyte research, the strategic integration of computational methodologies with targeted experimentation has enabled the discovery of novel high-performance solid electrolytes, including lithium garnets (e.g., Li_7_La_3_Zr_2_O_12_) and NASICON-type materials (e.g., Li_1+x_Al_x_Ti_2–x_(PO_4_)_3_). Computational screening approaches based on DFT, combined with defect chemistry modeling and thermodynamic stability analyses, have proven invaluable in identifying compositions with low ion migration barriers, wide electrochemical windows, and robust mechanical properties. The iterative cycle of computational prediction, focused experimental synthesis, rapid performance evaluation, and model refinement has created a virtuous feedback loop that has significantly accelerated the materials discovery timeline. This iterative approach, now a cornerstone of lithium-ion electrolyte development, could be effectively applied to the discovery and optimization of proton-conducting oxides, which face parallel challenges in balancing ionic conductivity, chemical durability, and manufacturability.

Similarly, recent advances in sodium-ion electrolytes, particularly with sodium superionic conductors based on NASICON-type frameworks, have demonstrated the importance of systematic compositional tuning and lattice architecture design. By varying cation species, stoichiometry, and structural motifs, researchers have optimized Na-ion mobility while enhancing stability against atmospheric degradation and electrode reactivity.^534^ These strategies underscore the importance of simultaneously addressing ionic size effects, local lattice distortions, defect energetics, and framework flexibility—principles that are directly translatable to the rational design of proton-conducting materials, where optimizing proton mobility must be balanced with chemical and mechanical resilience under realistic operating conditions.

Adopting the proven methodologies established in Li-ion and Na-ion electrolyte research, proton-conducting oxide development can greatly benefit from a systematic, combinatorial approach to experimental synthesis and characterization. Coupled with accelerated testing protocols—including high-throughput impedance spectroscopy, environmental stability screening, and mechanical durability assessments—these workflows allow for the efficient screening and down-selection of promising candidates at an early stage.

In parallel, the development of comprehensive experimental and computational databases—analogous to those developed for lithium- and sodium-based electrolytes—is expected to play a pivotal role in enabling machine learning-driven optimization of proton conductors. Databases containing systematically curated information on proton conductivity, activation energies, chemical stability limits, thermal expansion coefficients, mechanical properties, and phase stability can serve as critical resources for both human-guided and AI-assisted materials design.^535^

Ultimately, the adoption of systematic, iterative, and data-driven workflows—successfully demonstrated in the development of lithium- and sodium-ion electrolytes—holds the potential to substantially streamline the discovery process for proton-conducting oxides. By integrating high-throughput computation, combinatorial synthesis, rapid property characterization, machine learning, and database-driven analysis, the proton-conducting materials community can significantly accelerate the identification, optimization, and deployment of high-performance electrolytes tailored for specific chemical or energy transformation applications—including SOFCs, PCECs, and hydrogen separation membranes.^536,537^

The convergence of these methodologies offers a promising pathway to overcome longstanding bottlenecks in the development of proton-conducting electrolytes, enabling more rapid and efficient translation of fundamental material insights into commercially viable and technologically transformative energy solutions.

#### 6.2.4 A Spiral Development Roadmap for Proton-Conducting Electrolytes in P-RSOCs

A strategic vision for advancing the field, presenting a comprehensive roadmap for future development from five interrelated perspectives, is provided in Figure 38. The framework organizes materials discovery and validation into five interconnected phases arranged as a spiral rather than a linear pipeline. Phase I establishes a mechanistically grounded feature-space foundation through a closed loop of physics–measurement–evaluation in well-understood proton conductors. Phase II navigates vast chemical spaces via active learning, iterating among models, data and decision-making to efficiently identify promising compositions. Phase III serves as the first experimental bottleneck, validating intrinsic material properties through reproducible synthesis–diagnosis–validation. Phase IV tests property transferability at the device level, assessing whether intrinsic electrolyte behavior can be preserved under realistic P-RSOC operation via integration–operation–assessment. Phase V closes the spiral by transforming operando device observations into transferable knowledge through observation–mechanism–principle, feeding mechanistic insights back into the feature space and surrogate models for subsequent iterations. Within each phase, the three elements form a directional microcycle, followed in a clockwise order from the upper segment to the lower-right and then the lower-left segment, emphasizing iterative refinement rather than static categorization. Together, the spiral highlights interpretability, transferability, and predictive power across scales from fundamental physics to operating devices.

**Figure 38:**
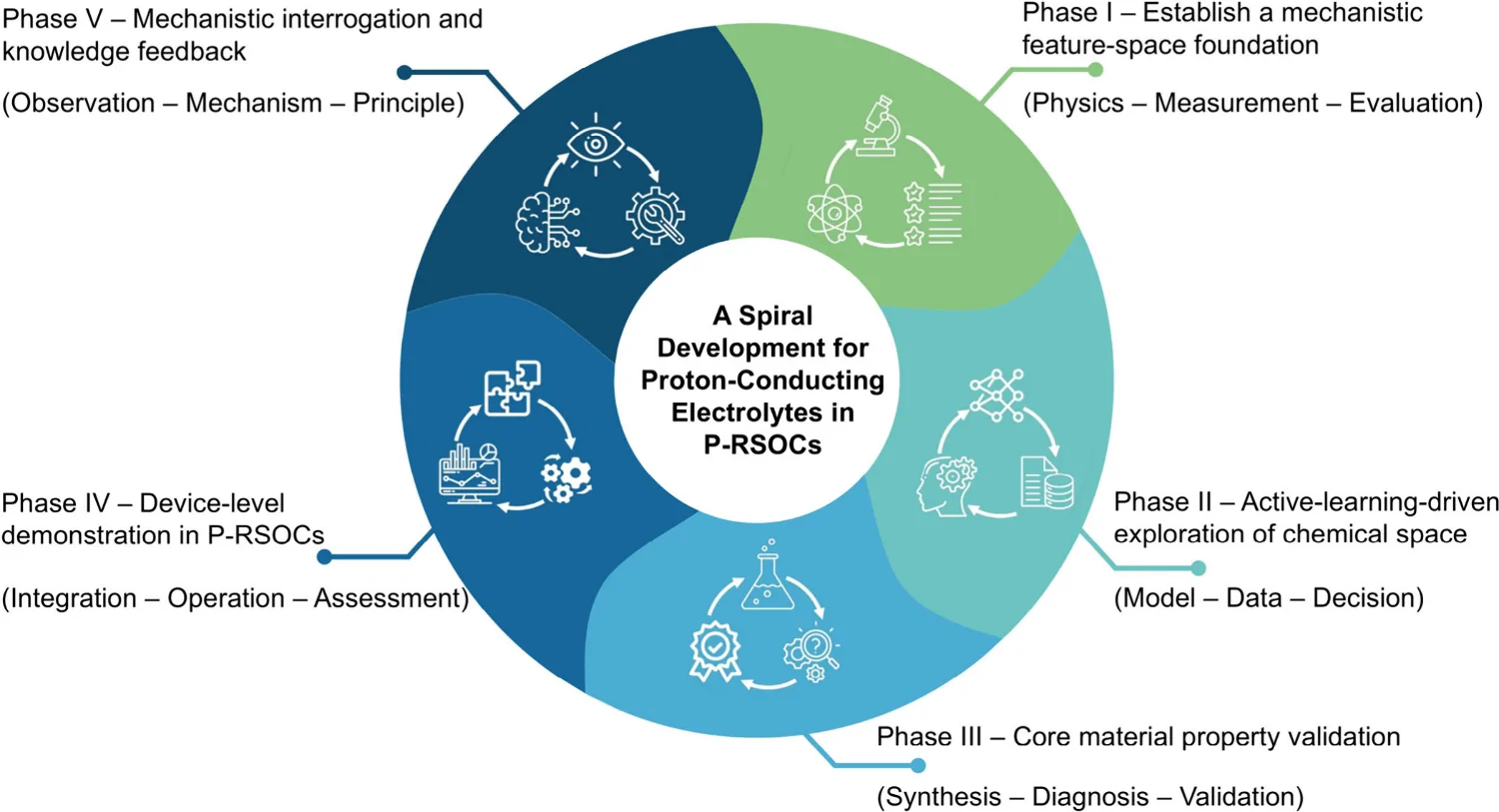
A spiral development roadmap for predictive design of proton-conducting electrolytes in P-RSOCs.

##### 6.2.4.1 Phase I: Establish a Mechanistic Feature-Space Foundation

To initiate a truly predictive design cycle, the first phase must construct a physically grounded model anchored in well-understood proton conductors rather than in silico abstractions. Canonical systems such as BaZrO_3−δ_, BaCeO_3−δ_, and their solid solutions (e.g., BZCY, BZCYYb, BHYb) already benefit from decades of combined electrochemical, structural, and spectroscopic study. Phase I consolidates this dispersed knowledge into a coherent mechanistic foundation by systematically interrogating these materials with the most informative tools summarized in Section 4—QENS, neutron and X-ray diffraction, solid-state NMR, operando Raman and IR spectroscopy, TOF-SIMS/isotope exchange, PALS, advanced EIS/EMF protocols, and complementary first-principles calculations and AIMD simulations. Together, these techniques yield a high-resolution picture of the defect landscape and transport mechanisms across multiple length and time scales.

The central task of this phase is to translate that mechanistic picture into a quantitative feature space that can serve as input for data-driven models. Beyond conventional scalar descriptors such as nominal dopant content or tolerance factor, the feature set should encode: hydration thermodynamics (ΔH_hyd_, hydration entropy, hydration isotherms and kinetic exponents); defect formation and association energies for oxygen vacancies, protonic defects, and dopant–vacancy complexes; migration barriers and preferred pathways for both protons and oxide ions (from DFT/AIMD and QENS-derived jump statistics); proton transference numbers and onsets of electronic leakage; grain-boundary blocking factors, space-charge layer parameters, and intergranular phase fractions; microstructural metrics such as grain size distributions, porosity, tortuosity, and susceptibility to Ba loss or secondary phase formation under realistic sintering schedules; and, where available, measures of proton–phonon coupling, lattice softness, and vibrational density of states. In parallel, chemical stability descriptors—such as decomposition energies in H_2_O/CO_2_-containing atmospheres, redox stability windows as a function of pO_2_, and mechanical compatibility with typical electrode and support materials—are incorporated to ensure that transport performance is always evaluated alongside robustness.

By embedding this rich set of experimentally constrained and theoretically validated properties into a structured, vectorized mechanistic feature space, Phase I provides the training ground for a first-generation surrogate model within an active-learning framework. Because the input chemistries are restricted to systems whose defect chemistry, transport behavior, and degradation modes are already well characterized, the resulting model is naturally biased toward physically meaningful correlations rather than spurious patterns. This mechanistically anchored starting point is essential for avoiding common pitfalls of high-throughput screening—false positives that appear promising on the basis of simple energetics or crystal-structure descriptors but in practice fail due to unrealistic stability assumptions, overlooked sintering barriers, severe grain-boundary blocking, or unphysical defect landscapes. In this way, Phase I does more than assemble data: it distills mechanistic understanding into a navigable feature space, upon which subsequent phases of active exploration and device-level validation can reliably build.

##### 6.2.4.2 Phase II: Active-Learning-Driven Exploration of Chemical Space

Where Phase I builds a mechanistic foundation in a relatively narrow set of well-understood systems, Phase II uses that foundation to navigate the vastly larger, only partially charted chemical space. The central engine here is active learning: instead of passively fitting a model once and then screening millions of compounds in a blind, one-shot manner, active learning treats material selection as an iterative dialogue between model and experiment (or high-level computation). At each iteration, a surrogate model—trained on the Phase I feature space and subsequent observations—predicts both the expected performance of untested compositions and the uncertainty of those predictions. Acquisition strategies (e.g., exploitation of high-predicted performers, exploration of high-uncertainty regions, or multiobjective trade-offs between performance and risk) are then used to choose the next batch of compositions to evaluate. In this way, each new data point is maximally informative for refining the model, rather than just adding more points to an already well-sampled region.

In practice, active learning in proton-conducting oxides must contend with the well-known limitations of high-throughput computation. Standard DFT workflows can efficiently provide formation energies, approximate phase stability, and zero-K defect formation energies across many compositions, but they do not directly deliver finite-temperature enthalpies and entropies of hydration, defect association equilibria, or realistic activation barriers for proton migration in disordered environments. Explicit AIMD or phonon calculations for every candidate are prohibitively expensive. This gap motivates the construction of surrogate models for thermodynamics and kinetics: machine-learned regressors or force fields that map from structural and chemical descriptors to hydration free energies, migration barriers, or effective diffusion coefficients, trained on a smaller but mechanistically rich subset of high-fidelity calculations and experiments. Within Phase II, these surrogates act as a “thermodynamic and kinetic emulator,” allowing the active-learning loop to reason about stability and transport at scale, even when direct calculations would be prohibitive.

The chemical spaces of interest in P-RSOC electrolytes illustrate why such an approach is necessary. Even within the ostensibly simple perovskite family, design variables include: A-site composition (Ba/Sr/Ca mixtures; partial rare-earth substitution), B-site multimetal combinations (Zr, Ce, Hf, Sn, Ti, Nb, Ta, Mo), multiple acceptor dopants (Y, Yb, Sc, In, Gd, Ga) at different concentrations, and the possibility of codoping or charge-compensating strategies. The combinatorial explosion in compositions is further compounded in more complex families such as Ba_7_Nb_4‑x_Mo_1+x_O_20+x/2_ hexagonal perovskite-related oxides, layered Ruddlesden–Popper phases, LaNbO_4_-type scheelites, oxy-apatites, brownmillerites, and disordered fluorite- or pyrochlore-based proton conductors. Many of these spaces remain sparsely explored, not because they are uninteresting, but because the data throughput needed to systematically map their stability, hydration behavior, and sintering characteristics has not yet been achieved. They are precisely the regimes where active learning can have the greatest impact: steering limited high-fidelity calculations and experiments toward the most informative and promising corners of multidimensional composition–structure space.

Finally, Phase II explicitly incorporates practical feasibility filters into the exploration loop. Candidates are not judged solely by theoretical σ_bulk_ or idealized stability; the surrogate model or postprocessing layer also screens for sinterability (e.g., estimated densification temperature, propensity for Ba loss), tolerance-factor and lattice-mismatch constraints, A- and B-site volatility, dopant solubility limits, and robustness in mixed H_2_/H_2_O/CO_2_ atmospheres. In combination with uncertainty-aware acquisition, this ensures that active learning does not simply propose exotic, “paper-only” materials with superb calculated transport but catastrophic processing or stability issues. Instead, Phase II delivers a prioritized, realistically synthesizable short list of compositions whose predicted performance, mechanistic plausibility, and fabrication risk jointly justify the more resource-intensive validation and device-level testing undertaken in subsequent phases.

##### 6.2.4.3 Phase III: Core Material Property Validation

Once Phase II identifies a tractable set of promising compositions, Phase III establishes whether these candidates truly possess the intrinsic proton-conducting properties predicted by the computation and active learning. This phase serves as the first rigorous experimental bottleneck and therefore must be executed with highly standardized, reproducible, and mechanistically transparent synthesis and characterization protocols. Without such standardization, the performance of a candidate becomes inseparable from artifacts of processing, making it impossible to determine whether the material itself is promising or whether poor synthesis conditions mask its true potential.

A critical requirement is the adoption of a stable and fully documented synthesis route capable of reproducibly expressing a material’s intrinsic defect chemistry across multiple batches, laboratories, and fabrication environments. For conventional perovskite electrolytes, this often includes: (i) high-purity oxide or carbonate precursors with controlled carbonate removal; (ii) carefully conditioned ball-milling or planetary milling to ensure homogeneous dopant distribution; (iii) calcination profiles that minimize Ba loss and prevent intermediate-phase trapping; (iv) sintering schedules that target >94–96% density with controlled heating and cooling rates; and (v) crucible and support materials selected to avoid Si, Al, or alkali contamination. Parallel synthesis routes—such as reactive sintering, sol–gel precursors, or polymer-assisted synthesis—may be evaluated for compositions predicted to be difficult to densify. Establishing these reference protocols is essential: without them, even the most promising composition from Phase II cannot be reliably validated, and the entire active-learning loop loses its grounding.

Phase III also acknowledges a recurring observation: many chemically promising, high-scoring compositions fail during synthesis due to intrinsic processing limitations. For example, highly Zr-rich or Hf-rich perovskites predicted to exhibit excellent proton mobility often require sintering temperatures exceeding 1650 °C, leading to substantial Ba volatilization, the formation of insulating grain-boundary phases, and catastrophic suppression of transference number. Similarly, Nb- and Mo-substituted perovskites or complex hexagonal derivatives can segregate into multiple phases unless dopant solubility limits and charge-compensating schemes are respected. Even Ce-containing compositions, which typically sinter well, may introduce unintended electronic leakage or structural instability under reducing conditions. These failure modes do not imply that such high-performance chemistries should be abandoned; rather, they highlight the need for complementary engineering approaches.

Several solution pathways exist and should be systematically deployed within Phase III. For refractory perovskites, strategies such as transient liquid-phase sintering, ZnO-assisted densification, cold sintering, SPS, or the addition of carefully chosen sacrificial sintering aids may drastically reduce densification temperatures while preserving σ_bulk_. For multicomponent or hexagonal systems, precursor route engineering, controlled oxygen partial pressure, or slow-step calcination can suppress phase segregation. Where grain-boundary blocking dominates, targeted use of sintering modifiers, grain-boundary cleaning protocols, or postsintering anneals can restore intrinsic proton mobility. Each candidate is diagnosed with a combination of structural probes (XRD/NPD, PDF, SEM/TEM), spectroscopies (XPS, Raman, FTIR), and electrochemical methods (EIS, EMF) to pinpoint the specific physical or chemical reason underlying its deviation from predicted behavior.

Thus, Phase III constitutes the first decisive test of transferability: can a composition predicted to excel on theoretical and mechanistic grounds actually be synthesized and densified in a manner that preserves its intrinsic properties? Only those candidates that demonstrate reproducible hydration thermodynamics, robust bulk and grain-boundary conductivity, stable transference numbers, and chemically resilient phase behavior advance to the device-level evaluation of Phase IV. In this way, Phase III forms the bridge between prediction and reality, ensuring that only truly viable electrolytes continue through the spiral development cycle.

Before entering Phase IV, it is important to acknowledge that the P-RSOC literature contains substantial discrepancies in reported conductivity, activation energies, hydration enthalpies, stability windows, and even qualitative transport mechanisms for nominally identical chemistries. These inconsistencies do not reflect contradictions in the underlying physics as much as they reveal the profound sensitivity of proton conductors to synthesis history, microstructural evolution, measurement configuration, and device-level architecture. Variations in precursor chemistry, carbonate removal, milling energy, calcination schedules, and sintering temperature produce markedly different grain sizes, Ba loss levels, secondary phases, and grain-boundary films—each capable of changing measured conductivity by an order of magnitude. At the cell level, discrepancies are amplified by differences in electrode porosity, seal chemistry, gas humidity control, and pO_2_/pH_2_O gradients, making cross-study comparisons particularly challenging. Recognizing the origins of these inconsistencies is essential: it clarifies that many “conflicting” results are not true contradictions but consequences of uncontrolled processing degrees of freedom. This realization motivates the need for standardized synthesis, reporting, and cell-testing protocols in Phases III and IV to ensure that mechanistic insights and material properties are genuinely comparable across laboratories.

##### 6.2.4.4 Phase IV: Device-Level Demonstration in P-RSOCs

After a candidate material has demonstrated reproducible intrinsic properties in Phase III, the next challenge is to determine whether those properties can be successfully transplanted into an operating P-RSOC. The purpose of Phase IV is not to showcase a specific cell-fabrication recipe, nor to maximize performance through engineering tricks, but rather to establish whether the core physicochemical attributes of the electrolyte—proton conductivity, transference number, hydration behavior, chemical robustness, and interfacial compatibility—can be expressed under device-relevant, realistic, and standardized operating conditions. In this sense, Phase IV is fundamentally a test of property transferability, not of cell optimization.

Achieving this requires a device environment that faithfully reflects the thermodynamic and kinetic constraints under which P-RSOCs operate. Regardless of the exact fabrication route (cofiring, tape casting, uniaxial pressing, infiltration, reactive sintering, screen printing), the key requirement is that the electrolyte enters the device in a microstructural and chemical state that preserves the intrinsic properties validated in Phase III. Excessive porosity, secondary phases, electrode contamination, or uncontrolled Ba loss can all obscure the intrinsic behavior, making it impossible to evaluate the material itself. Therefore, fabrication methods are treated as controlled interfaces, whose task is not to improve performance artificially but to enable reliable interrogation of the electrolyte’s genuine behavior on scale.

To ensure consistency, the testing environment must be standardized, taking inspiration from battery research where strict protocols for rate testing, cycle life evaluation, temperature control, and capacity retention have been established across the community. Analogously, P-RSOC electrolyte assessment should adopt reference atmospheres (e.g., fixed pH_2_O, controlled pO_2_/pH_2_ gradients, defined fuel utilization windows), standard operating temperatures (e.g., 500–700 °C), and prescribed equilibration procedures before measurement. Key metrics such as ASR, effective t_H^+^_ under load, PPD, internal steam electrolysis efficiency, open-circuit voltage consistency, and gas crossover levels should be defined by community-accepted protocols to enable cross-lab comparison. Without such standardized conditions, device-level performance becomes too dependent on reactor configuration, sealing quality, gas-flow architecture, or electrode microstructure, rendering the evaluation subjective and nontransferable.

Equally critical is a precise definition of device-level stability. In P-RSOCs, degradation is multimodal: hydration imbalance across the electrolyte can induce asymmetric expansion; electrode reactions may alter local defect chemistry; redox front propagation may destabilize cation sublattices; and CO_2_/H_2_O impurities can induce surface dissolution or blocking-layer formation. Therefore, Phase IV evaluates stability as a compound metric: (i) retention of ASR and power output over short-term operation (e.g., tens of hours), (ii) stability of local hydration profiles during fuel cell and electrolysis switching, (iii) resistance to electrode-induced chemical modification, (iv) tolerance against mechanical cycling and thermal shocks, and (v) the absence of emergent electronic leakage under reducing conditions. Beyond these performance-oriented metrics, overall efficiency definitions—including voltage efficiency, FE for steam electrolysis, and system-level energy efficiency—must be explicitly documented to avoid inconsistencies across studies.

Through these practices, Phase IV establishes whether the electrolyte can sustain device-relevant transport, hydration, and stability behavior even in the presence of realistic interfacial, mechanical, and chemical stresses. Many high-performing intrinsic proton conductors fail precisely at this stage because of unexpected interfacial reactions, incompatible microstructural evolution, or adverse gas-phase equilibria. Those candidates that succeed demonstrate that their core properties are genuinely expressible in a P-RSOC environment, earning the right to proceed to phase V, where deeper mechanistic interrogation closes the loop and guides the next iteration of rational electrolyte design.

##### 6.2.4.5 Phase V: Mechanistic Interrogation and Knowledge Feedback

Phase V represents the intellectual core of the spiral development roadmap—the point at which empirical device performance is transformed into explanatory knowledge, enabling a deeper and increasingly predictive understanding of proton-conducting oxides. Whereas Phase IV determines whether an electrolyte’s intrinsic properties can be expressed in a P-RSOC environment, Phase V asks the deeper question: why did a material succeed or fail? What mechanistic motifs supported its performance? Which structural, defect, or microstructural features became active only under operating conditions? And how can these insights be formalized into principles that generalize beyond a single composition?

To address these questions, Phase V relies on a suite of in situ and operando mechanistic probes, including neutron scattering (NPD, QENS, INS), operando Raman and IR spectroscopy, synchrotron X-ray scattering, ambient-pressure XPS, TOF-SIMS depth profiling, and high-temperature environmental TEM. These techniques offer complementary access to different aspects of proton transport: the evolution of hydration fronts, defect association and dissociation kinetics, real-time proton hopping statistics, lattice softening or hardening under redox cycling, grain-boundary phase formation, and sublattice reorganizations that produce emergent transport pathways. By performing these studies directly on operating P-RSOC cells rather than static pellets, Phase V reveals reaction mechanisms as they unfold under coupled electrical, chemical, thermal, and mechanical fields—conditions that cannot be replicated ex situ.

A major outcome of Phase V is the extraction and formalization of reaction mechanisms, including:

- proton-transfer pathways mediated by dynamic H-bond networks;
- defect–phonon coupling motifs that accelerate or hinder transport;
- dimer-mediated or cluster-mediated conduction in complex oxides;
- dynamically changing hydration equilibria across temperature and gas gradients;
- grain-boundary structural transitions that modulate charge carrier blocking;
- local-defect-landscape destabilization driven by Ba migration or A-site deficiency.

These mechanistic insights motivate the construction of new theoretical frameworks that unify multiscale behavior: atomic-scale defect energetics, mesoscopic microstructural evolution, and macroscopic device response. Existing models—classical defect chemistry, dilute-solution thermodynamics, simple polaron hopping, or homogeneous diffusion—are insufficient to describe the spatially heterogeneous, strongly coupled processes observed under P-RSOC operating conditions. Phase V, therefore, enables the development of multispecies transport theories, field-coupled reaction–diffusion models, stochastic or non-Markovian proton hopping frameworks, and microstructure-aware continuum descriptions that capture the complexity of real devices. These frameworks do more than explain individual observations: they provide a language for identifying universally transferable principles.

Crucially, Phase V forces the active-learning model to confront interpretability and generalizability. Interpretability arises when the surrogate model incorporates mechanistic constraints—e.g., defect association energies, doping-dependent hydration enthalpies, or experimentally measured jump frequencies—so that its predictions can be traced back to physical phenomena rather than opaque correlations. Generalizability emerges when these mechanistic constraints prevent the model from overfitting narrow training regions, enabling it to extrapolate meaningfully to new chemistries or structural classes. Rather than treating the model as a black box, Phase V elevates it into a mechanistic reasoning tool, capable of proposing hypotheses, identifying failure modes, and revealing structure–property relationships.

All insights obtained from Phase V—successful motifs, failure mechanisms, operando defect behavior, and emergent transport patterns—are finally collapsed back into the mechanistic feature space established in Phase I. The feature space expands and gains new dimensions reflecting the realities of device operation. The surrogate model is retrained, incorporating operando-derived mechanistic constraints that sharpen its predictions for the next iteration of Phase II exploration.

Thus, Phase V provides knowledge closure within the spiral development cycle. It transforms isolated observations into general principles, transforms empirical correlations into mechanistic laws, and transforms a list of candidate materials into a conceptual roadmap for future designs. Through this phase, the field moves progressively closer to the predictive, generalizable, and interpretable rational design of proton-conducting electrolytes for next-generation P-RSOCs.

## Nomenclature

H^+^: proton
O^2–^: oxide ion
t_i_: ionic transference number
t_H^+^_: proton transference number
t_O^2–^_: oxide ion transference number
t_e_: electron transference number
BZY20: BaZr_0.8_Y_0.2_O_3−δ_
BCY20: BaCe_0.8_Y_0.2_O_3−δ_
BCO: BaCeO_3−δ_
BZO: BaZrO_3−δ_
BHYb82: BaHf_0.8_Yb_0.2_O_3‑δ_
BZCYYb1711: BaZr_0.1_Ce_0.7_Y_0.1_Yb_0.1_O_3‑δ_
BZCYYb: BaZr_x_Ce_0.8–x_Y_0.1_Yb_0.1_O_3‑δ_
BHCYYb: BaHf_x_Ce_0.8–x_Y_0.1_Yb_0.1_O_3‑δ_
BZCY721: BaZr_0.7_Ce_0.2_Y_0.1_O_3‑δ_
BZCYYb4411: BaZr_0.4_Ce_0.4_Y_0.1_Yb_0.1_O_3‑δ_
BCZY62: BaCe_0.6_Zr_0.2_Y_0.2_O_3‑δ_
BSCF: Ba_0.5_Sr_0.5_Co_0.8_Fe_0.2_O_3‑δ_
LSCF: La_0.6_Sr_0.4_Co_0.2_Fe_0.8_O_3‑δ_
BHCYb172: BaHf_0.1_Ce_0.7_Yb_0.2_O_3‑δ_
BZCY172: BaZr_0.1_Ce_0.7_Y_0.2_O_3‑δ_
BSCYb: BaSn_x_Ce_0.8–x_Yb_0.2_O_3‑δ_
BCN18: Ba_3_Ca_1.18_Nb_1.82_O_9‑δ_
BSM20: BaSc_0.8_Mo_0.2_O_3–δ_
LMCO: La_1.85_Mg_0.15_Ce_2_O_7‑δ_
LCCO: La_1.95_Ca_0.05_Ce_2_O_7‑δ_
NCAL: Ni_0.8_Co_0.15_Al_0.05_LiO_2_
SNO: SmNiO_3_
SCO: SrCoO_2.5_
BCFZY: BaCo_0.4_Fe_0.4_Zr_0.1_Y_0.1_O_3‑δ_
R_o_: ohmic resistance
R_o,th_: theoretical ohmic resistance
R_p_: polarization resistance
R_b_: bulk resistance
R_gb_: grain boundary resistance
E_V_: oxygen vacancy formation energy
E_H_: hydration energy
BSCYb172: BaSn_0.1_Ce_0.7_Yb_0.2_O_3‑δ_
BSCYb352: BaSn_0.3_Ce_0.5_Yb_0.2_O_3‑δ_
BCS: BaCe_0.8_Sm_0.2_O_3‑δ_
20Y–BZO: 20% Y-doped BaZrO_3‑δ_
BZCY442: BaZr_0.4_Ce_0.4_Y_0.2_O_3‑δ_
BZCYZn: Five mol % Zn-doped BZCY442
BZCY–ZnO: BZCY442 produced by externally adding 1 wt % ZnO
BZCYN: BZCY721 samples sintered with 2 mol % NiO
BNM: Ba_7_Nb_4_MoO_20−δ_
BZY–15: Fifteen μm-thick BZY20
3QMAS: triple-quantum MAS
k_H_: surface proton exchange rate
D_H_: bulk proton tracer diffusion coefficient
D_chem_: chemical diffusion coefficient
σ_bulk_: bulk conductivity
σ_gb_: grain boundary conductivity
σ_tot_: total ionic conductivity
σ_H^+^_: proton conductivity
σ_O^2–^_: oxide ion conductivity
σ_e_: electron conductivity
R: resistors
W: Warburg impedances
ρ: bulk resistivity
H/D: hydrogen–deuterium
ΔH_hyd_: hydration enthalpy
AAO: anodic aluminum oxide
BCZY271: BaCe_0.2_Zr_0.7_Y_0.1_O_3‑δ_
BZCYYb2611: BaZr_0.2_Ce_0.6_Y_0.1_Yb_0.1_O_3‑δ_
**Acronyms**
P-RSOCs: proton-conducting reversible solid oxide cells
RSOCs: reversible solid oxide cells
SOCs: solid oxide cells
FE: Faradaic efficiency
YSZ: yttria-stabilized zirconia
NMR: nuclear magnetic resonance
PLD: pulsed laser deposition
XRD: X-ray diffraction
OCV: open-circuit voltage
SEM: scanning electron microscopy
GDC: gadolinium-doped ceria
PCFCs: protonic ceramic fuel cells
PCECs: protonic ceramic electrolysis cells
AC: alternating current
DC: direct current
ASR: area-specific resistance
EDS: energy-dispersive X-ray spectroscopy
TEC: thermal expansion coefficients
IT-SOFCs: intermediate-temperature solid oxide fuel cells
ALD: atomic layer deposition
DFT: density functional theory
MD: molecular dynamics
AIMD: ab initio molecular dynamics
XAS: X-ray absorption spectroscopy
TGA: thermogravimetric analysis
KFT: Karl Fischer titration
EMF: electromotive force
INS: inelastic neutron scattering
IR: infrared
FTIR: Fourier transform infrared
SOFC: solid oxide fuel cell
SDC: samarium-doped ceria
LT-SOFCs: low-temperature solid oxide fuel cells
PPD: peak power density
SBFCs: semiconductor-based fuel cells
SM: semiconductor membranes
SIM: semiconductor-ionic membranes
BIEF: built-in electric field
SLFCs: single-layer fuel cells
ILG: ionic liquid gating
DLFC: double-layer fuel cell
SJ: Schottky junction
SOECs: solid oxide electrolysis cells
XPS: X-ray photoelectron spectroscopy
EIS: electrochemical impedance spectroscopy
HAADF-STEM: high-angle annular dark field scanning transmission electron microscopy
APT: atom probe tomography
FE-EPMA: field emission electron probe microanalysis
TPB: triple-phase boundary
AFL: anode functional layer
UHS: ultrafast high-temperature sintering
DRT: distribution-of-relaxation-times
SSR: solid-state reaction
NAP-XPS: near-ambient-pressure XPS
TPD: temperature-programmed desorption
SERS: surface-enhanced Raman spectroscopy
NMF: non-negative matrix factorization
TOF: time-of-flight
TOF-SIMS: time-of-flight secondary ion mass spectrometry
NPD: neutron powder diffraction
QENS: quasi-elastic neutron scattering
PDF: pair-distribution-function
NSLD: neutron scattering-length density
NEB: nudged elastic band
EXSY: two-dimensional exchange spectroscopy
PGAA: prompt-gamma activation analysis
MAS: magic-angle-spinning
PALS: positron annihilation lifetime spectroscopy
ERD: elastic-recoil-detection
IEDP: isotope exchange diffusion profiling
CPE: constant-phase elements
IBE: ion-blocking electrode
RE: reversible electrode
WPSC: wet powder spray coating
PVB: polyvinyl butyral
PVD: physical vapor deposition
RF: radio frequency
CVD: chemical vapor deposition
RLRS: rapid laser reactive sintering
SSRS: solid-state reactive sintering
MWS: microwave sintering
